# Antenatal magnesium sulphate and adverse neonatal outcomes: A systematic review and meta-analysis

**DOI:** 10.1371/journal.pmed.1002988

**Published:** 2019-12-06

**Authors:** Emily Shepherd, Rehana A. Salam, Deepak Manhas, Anne Synnes, Philippa Middleton, Maria Makrides, Caroline A. Crowther

**Affiliations:** 1 Robinson Research Institute, Discipline of Obstetrics and Gynaecology, Adelaide Medical School, University of Adelaide, Adelaide, South Australia, Australia; 2 South Australian Health and Medical Research Institute, Adelaide, South Australia, Australia; 3 University of British Columbia, Vancouver, British Columbia, Canada; 4 Liggins Institute, University of Auckland, Auckland, New Zealand; University of Manchester, UNITED KINGDOM

## Abstract

**Background:**

There is widespread, increasing use of magnesium sulphate in obstetric practice for pre-eclampsia, eclampsia, and preterm fetal neuroprotection; benefit for preventing preterm labour and birth (tocolysis) is unproven. We conducted a systematic review and meta-analysis to assess whether antenatal magnesium sulphate is associated with unintended adverse neonatal outcomes.

**Methods and findings:**

CINAHL, Cochrane Library, LILACS, MEDLINE, Embase, TOXLINE, and Web of Science, were searched (inceptions to 3 September 2019). Randomised, quasi-randomised, and non-randomised trials, cohort and case–control studies, and case reports assessing antenatal magnesium sulphate for pre-eclampsia, eclampsia, fetal neuroprotection, or tocolysis, compared with placebo/no treatment or a different magnesium sulphate regimen, were included. The primary outcome was perinatal death. Secondary outcomes included pre-specified and non-pre-specified adverse neonatal outcomes. Two reviewers screened 5,890 articles, extracted data, and assessed risk of bias following Cochrane Handbook and RTI Item Bank guidance. For randomised trials, pooled risk ratios (RRs) or mean differences, with 95% confidence intervals (CIs), were calculated using fixed- or random-effects meta-analysis. Non-randomised data were tabulated and narratively summarised. We included 197 studies (40 randomised trials, 138 non-randomised studies, and 19 case reports), of mixed quality. The 40 trials (randomising 19,265 women and their babies) were conducted from 1987 to 2018 across high- (16 trials) and low/middle-income countries (23 trials) (1 mixed). Indications included pre-eclampsia/eclampsia (24 trials), fetal neuroprotection (7 trials), and tocolysis (9 trials); 18 trials compared magnesium sulphate with placebo/no treatment, and 22 compared different regimens. For perinatal death, no clear difference in randomised trials was observed between magnesium sulphate and placebo/no treatment (RR 1.01; 95% CI 0.92 to 1.10; 8 trials, 13,654 babies), nor between regimens. Eleven of 138 non-randomised studies reported on perinatal death. Only 1 cohort (127 babies; moderate to high risk of bias) observed an increased risk of perinatal death with >48 versus ≤48 grams magnesium sulphate exposure for tocolysis. No clear secondary adverse neonatal outcomes were observed in randomised trials, and a very limited number of possible adverse outcomes warranting further consideration were identified in non-randomised studies. Where non-randomised studies observed possible harms, often no or few confounders were controlled for (moderate to high risk of bias), samples were small (200 babies or fewer), and/or results were from subgroup analyses. Limitations include missing data for important outcomes across most studies, heterogeneity of included studies, and inclusion of published data only.

**Conclusions:**

Our findings do not support clear associations between antenatal magnesium sulphate for beneficial indications and adverse neonatal outcomes. Further large, high-quality studies (prospective cohorts or individual participant data meta-analyses) assessing specific outcomes, or the impact of regimen, pregnancy, or birth characteristics on these outcomes, would further inform safety recommendations. PROSPERO: CRD42013004451.

## Abstract 2

Shepherd and colleagues investigate whether the use of magnesium sulphate in obstetric practice is associated with unintended adverse outcomes for neonates.

## Author summary

**Why was this study done?:**

- Magnesium sulphate is widely used in pregnancy, considered effective for maternal neuroprotection in pre-eclampsia/eclampsia and for fetal neuroprotection (cerebral palsy prevention) in women at risk of preterm birth, and ineffective for preventing preterm birth or labour (tocolysis).
- It is important to understand whether this treatment, when given to women in pregnancy, is associated with any unintended adverse outcomes for babies.

**What did the researchers do and find?:**

- This systematic review incorporates the findings of 197 studies (including 40 randomised controlled trials) that reported on adverse outcomes for babies whose mothers were treated with magnesium sulphate in pregnancy.
- Meta-analysis of randomised controlled trials showed no clear difference in the risk of perinatal death between babies whose mothers were treated with magnesium sulphate and those whose mothers received placebo/no treatment, or between different magnesium sulphate regimens.
- No clear adverse outcomes for babies were observed in randomised trials, and a very limited number of possible adverse outcomes warranting further consideration were identified in non-randomised studies.

**What do these findings mean?:**

- Magnesium sulphate in pregnancy, when given for the beneficial indications of maternal or fetal neuroprotection, is not associated with an increased risk of perinatal death or other adverse outcomes for babies.
- Further investigation into specific adverse outcomes, and the impact of particular treatment regimen, pregnancy, and/or birth characteristics, would further inform safety recommendations.

## Introduction

Antenatal magnesium sulphate is commonly used in obstetric practice. Systematic reviews and clinical practice guidelines support its use when given for maternal neuroprotection in pre-eclampsia or eclampsia [1–3] and for neuroprotection of the fetus in women at risk of preterm birth (for cerebral palsy prevention) [4–7]. Despite continued use in some countries [8], available evidence does not support its role in preventing preterm birth in women with, or following, threatened preterm labour (for tocolysis) [7,9].

Concerns surrounding possible unintended adverse outcomes for fetuses or neonates following exposure to antenatal magnesium sulphate emerged over 50 years ago [10,11], and uncertainty persists today. While the clinical consequences of hypermagnesemia, related to increased serum concentrations, are well known and documented (such as lethargy, drowsiness, flushing, nausea, vomiting, muscle weakness, loss of deep tendon reflexes, hypotension, apnoea, coma, cardiac arrest, and, ultimately, death [12]), whether neonates are at risk of such adverse outcomes following exposure to antenatal magnesium sulphate is unclear.

We have systematically reviewed the maternal adverse effects of different antenatal magnesium sulphate regimens [13], and a further systematic review has summarised the effects specifically on fetal heart rate [14]. To our knowledge, a broad evaluation of evidence surrounding potential unintended neonatal adverse outcomes, informed by current guidance [15–17], has not previously been conducted. Implementation of this treatment may be strengthened, and its safety improved, if guidelines and recommendations for practice can be based on such knowledge.

The aim of our study, therefore, was to conduct a comprehensive systematic review to assess whether antenatal magnesium sulphate is associated with perinatal death and other unintended adverse neonatal outcomes.

## Methods

We conducted a systematic review following the Preferred Reporting Items for Systematic Reviews and Meta-Analyses (PRISMA) guideline; the relevant checklist is provided in S1 PRISMA Checklist. Prior to conduct, this systematic review was registered with PROSPERO (International Prospective Register of Systematic Reviews; CRD42013004451) [18]. The Australian Cerebral Palsy Alliance Research Foundation–funded review protocol is available in S1 Text. Ethical approval was not required.

### Search strategy

Comprehensive searches of the bibliographic databases CINAHL, Cochrane Library, LILACS, MEDLINE, Embase, TOXLINE, and Web of Science were undertaken from their respective inceptions to 3 September 2019, using combinations of MeSH and free text terms. The search strategies are available in S2 Text. No date or language restrictions were applied; however, because of logistical constraints, for non-English papers, only those with an available English abstract or full-text translation were retrieved. The reference lists of eligible articles were checked for additional reports.

### Inclusion criteria

We included randomised and quasi-randomised controlled trials as well as non-randomised controlled studies (non-randomised trials, cohort studies, and case–control studies), and case reports. We excluded cross-sectional studies and case series. We included studies available as abstracts only, along with full-text publications.

We included neonates who were exposed to antenatal magnesium sulphate, regardless of their gestational age at exposure or birth. We included studies where antenatal magnesium sulphate was given for pre-eclampsia or eclampsia, for neuroprotection of the fetus, or for tocolysis. We excluded studies where magnesium sulphate was given as an adjuvant during obstetric anaesthesia. We included intervention studies in which magnesium sulphate was compared with no treatment, placebo, or a different magnesium sulphate regimen. We included observational studies where magnesium sulphate was assessed as an ‘exposure’. We excluded studies where magnesium sulphate was compared with another therapy (for example, diazepam for pre-eclampsia or eclampsia, or nifedipine for tocolysis).

We included studies that reported on adverse outcomes for neonates, however defined. The primary outcome was perinatal death. Secondary outcomes included pre-specified adverse outcomes (based on non-systematic literature review: stillbirth, neonatal death or death up to hospital discharge, low Apgar scores at 1 and 5 minutes, need for active resuscitation at birth, respiratory depression, spontaneous intestinal perforation, patent ductus arteriosus, hypotension, lethargy, hypotonia or hyporeflexia, osteopenia or bone fractures, neonatal intensive care unit admission, and duration of neonatal care unit admission), along with other non-pre-specified adverse neonatal outcomes.

### Study selection

After screening all titles and abstracts, we obtained full-text articles for studies that appeared to meet the inclusion criteria. All full-text articles were assessed for inclusion. Each stage was carried out by 2 reviewers, and we resolved any discrepancies through discussion, or, if required, we consulted a third reviewer.

### Data extraction and management

For included studies, data were extracted using a standardised form, including information regarding design, participants, the magnesium sulphate regimen(s), the control/comparison if applicable, neonatal adverse outcomes reported, results relevant to the review, and the risk of bias. For all randomised trials, all case reports, and 60% of non-randomised studies, extraction was carried out by 2 reviewers (for 40% of non-randomised studies, extractions were checked by a second reviewer), and we resolved discrepancies through discussion, or, if required, we consulted a third reviewer.

### Assessment of risk of bias

Quality appraisal of intervention studies was undertaken utilising established guidelines provided in the Cochrane Handbook for Systematic Reviews of Interventions [19]. The quality assessment of observational studies was guided by the RTI Item Bank [20].

### Data synthesis and analysis

Data analyses were undertaken by study design. Statistical analyses for randomised trials were performed using Review Manager, version 5.3 [21]. We present quantitative data from individual studies as risk ratios (RRs) for dichotomous outcomes and mean differences (MDs) for continuous outcomes, with 95% confidence intervals (CIs). For all outcomes, we carried out analyses as far as possible on an intention-to-treat basis. Pooled estimates were calculated using fixed-effects meta-analysis (Mantel-Haenszel method) where there was a sufficient quantity of data, with clinical homogeneity. Where there was substantial statistical heterogeneity (where *I*^2^ was greater than 30% and either *T*^2^ was greater than 0 or there was a low *P* value [less than 0.10] in the χ^2^ test), summary estimates were calculated using random-effects meta-analysis. Where there were 10 or more trials in a meta-analysis, we investigated reporting biases (such as publication bias) using funnel plots, which we assessed visually.

Separate comparisons were performed for those studies assessing magnesium sulphate versus no treatment/placebo and those comparing different magnesium sulphate regimens. For our primary review outcome (perinatal death) and other mortality outcomes, we conducted subgroup analyses based on indication for use and characteristics of the magnesium sulphate loading and maintenance dose regimens, as these factors were considered likely to influence outcomes. It was not possible to conduct subgroup analyses based on other pregnancy or birth characteristics (gestational age at magnesium sulphate administration, birthweight, mode of birth, and concomitant maternal treatments) due to paucity of data. We assessed subgroup differences by interaction tests available within Review Manager, and, where applicable, we have quoted the χ^2^ statistic and *P* value, and the interaction test *I*^2^ value.

For observational studies (non-randomised trials, cohort studies, and case–control studies), we present effect estimates where possible as adjusted RRs or odds ratios if reported with 95% CIs, unadjusted RRs or odds ratios with 95% CIs, *P* values only, or percentages (rates), in tabular format; we used narrative synthesis to summarise the studies. Data from case reports were grouped according to common adverse outcomes, tabulated, and summarised narratively.

## Results

### Study selection

**Fig 1:**
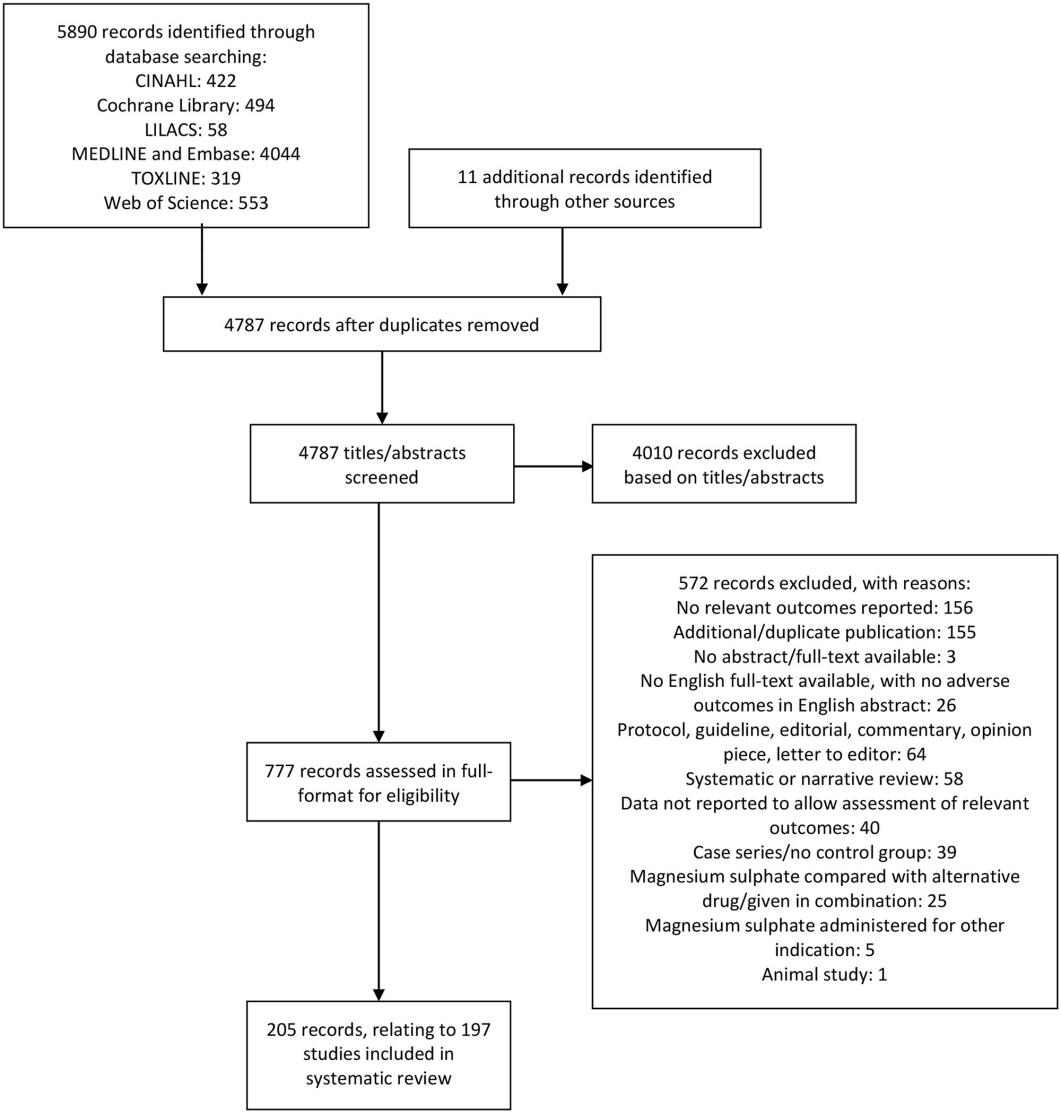
Flow diagram of included studies. Flow diagram showing the flow of records through the different phases of the review, indicating the number of records identified, included and excluded, and the reasons for exclusions.

The results of the search strategy, including the sources of the studies, their assessment, and final inclusion are shown in Fig 1. The database searching identified 5,890 records, and other searching identified a further 11 records. Review of the titles and abstracts and exclusion of irrelevant and duplicate records yielded 777. Of these, we excluded 572 for the documented reasons (see S3 Text for list of records excluded due to absence of an English translation). We included a total of 205 articles, relating to 197 studies. See S4 Text for references for all included studies. In the case of multiple publications from the same study, we included the report with the most relevant data as the primary reference, and only included further publications as secondary references if they provided additional relevant data.

### Evidence from randomised controlled trials

**Fig 2:**
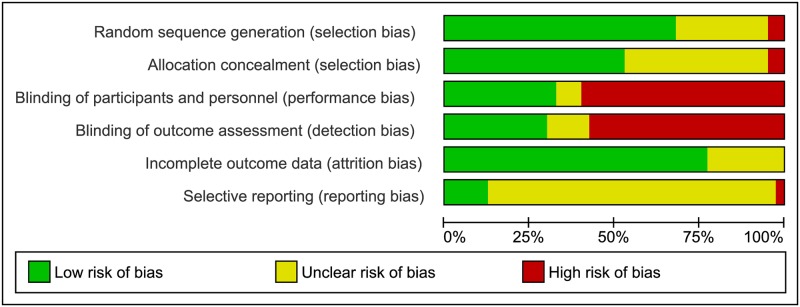
Risk of bias for randomised controlled trials. Risk of bias graph showing judgements about each risk of bias item presented as percentages across the 40 included randomised trials.

Forty randomised trials were included, the characteristics of which are detailed in S1 Table, and the risk of bias assessments are summarised in Fig 2, S1 Fig, and S2 Table [22–61]. The trials assessed a range of different magnesium sulphate regimens with varying control groups, and are therefore assessed under 8 different comparisons:

1. Magnesium sulphate versus placebo or no treatment (18 trials)
2. Lower versus higher dose regimens of magnesium sulphate (8 trials)
3. Intramuscular (IM) versus intravenous (IV) maintenance dose of magnesium sulphate (5 trials)
4. Loading versus loading and maintenance dose of magnesium sulphate (5 trials)
5. Serial IV boluses versus continuous IV infusion of magnesium sulphate (1 trial)
6. Short versus standard maintenance of magnesium sulphate (1 trial)
7. Slower versus standard rate of loading dose infusion of magnesium sulphate (1 trial)
8. Weaning versus no weaning of maintenance of magnesium sulphate (1 trial)

The methodological quality of the 40 trials varied considerably. Considering selection bias, 18 trials were at low risk, reporting adequate methods for sequence generation and allocation concealment. Twelve and 8 trials received an unclear judgement for 1 and 2 of the selection bias domains, respectively. Two trials appeared to be quasi-randomised and thus were at high risk of selection bias. Twelve trials were at low risk of performance and detection bias (with blinding of participants, personnel, and outcome assessors); 21 were at high risk of both performance and detection bias (all trials of different magnesium sulphate regimens, with no reported blinding), and the remaining 7 trials had an unclear judgement for 1 or 2 of the blinding domains. The majority of trials (31) were at low risk of attrition bias for neonatal outcome data, though for 9 trials, this was unclear. Only 5 trials were at low risk of reporting bias, 1 was at high risk of reporting bias, and for the remaining 34 trials, selective reporting was unclear.

### Magnesium sulphate versus placebo or no treatment

This comparison included 18 trials. The indication for use of magnesium sulphate in 6 trials was the prevention of eclampsia [30,32,39,43,48,61]; in 6 trials, fetal neuroprotection [28,36,45,47,51,54]; and in 6 trials, the prevention of preterm birth (tocolysis) [33–35,38,40,46]. Magnesium sulphate regimens assessed varied considerably: 4-gram IV loading dose only (2 trials), 4-gram IV loading dose and 1-gram-per-hour IV maintenance dose (4 trials), 4-gram IV loading dose and 2-gram-per-hour IV maintenance (4 trials), 6-gram IV loading dose and 2-gram-per-hour IV maintenance dose (6 trials), and 4-gram IV and 10-gram IM loading dose and 5-gram IM maintenance dose every 4 hours (2 trials), with duration of treatment generally ranging from 12 to 24 hours. Fourteen trials compared magnesium sulphate with a placebo, while 4 trials had a no-treatment comparison (see Table 1 and S1 Appendix for effect estimates, forest plots, and funnel plots).

**Table 1:** Adverse outcome estimates from randomised controlled trials: Comparison 1—Magnesium sulphate versus placebo or no treatment.

Outcome	Studies	Participants	Method (*I*^2^)	RR (95% CI)
1.1 Perinatal death	8	13,654	F (23%)	1.01 (0.92, 1.10)
1.2 Stillbirth	9	12,340	F (0%)	0.99 (0.87, 1.12)
1.3 Neonatal death	11	12,987	F (21%)	1.00 (0.86, 1.17)
1.4 Death > 28 days, before discharge	5	10,691	F (0%)	0.96 (0.60, 1.53)
1.5 Early neonatal death	1	9,024	F (NA)	1.09 (0.86, 1.37)
1.6 Late neonatal death	1	9,024	F (NA)	1.54 (0.95, 2.49)
1.7 Apgar score < 7 at 1 minute	2	199	F (17%)	**1.67 (1.02, 2.73)**
1.8 Apgar score < 7 at 5 minutes	5	12,729	F (0%)	1.02 (0.92, 1.14)
1.9 Meconium at birth	1	210	F (NA)	1.55 (0.89, 2.72)
1.10 Intubated at birth	3	11,364	F (30%)	0.95 (0.87, 1.04)
1.11 Resuscitation in the delivery room				
1.11.1 Any	1	2,416	F (NA)	0.99 (0.96, 1.03)
1.11.2 Oxygen bag, mask, or both	1	2,416	F (NA)	1.07 (0.98, 1.17)
1.11.3 Chest compressions	1	2,416	F (NA)	1.11 (0.73, 1.71)
1.12 Respiratory distress syndrome	7	3,639	R (46%)	0.95 (0.79, 1.14)
1.13 Transient tachypnoea of the newborn	2	243	F (0%)	0.96 (0.52, 1.77)
1.14 Surfactant	1	87	F (NA)	0.88 (0.62, 1.24)
1.15 Mechanical ventilation	5	12,751	R (63%)	1.01 (0.94, 1.09)
1.16 Non-invasive ventilation	1	688	F (NA)	1.03 (0.93, 1.15)
1.17 Oxygen required	1	153	F (NA)	0.95 (0.67, 1.35)
1.18 Chronic lung disease	5	4,513	F (0%)	1.06 (0.96, 1.17)
1.19 Apnoea and bradycardia	2	841	F (0%)	1.23 (0.98, 1.53)
1.20 Pneumothorax	1	87	F (NA)	2.44 (0.26, 22.52)
1.21 Pulmonary haemorrhage	1	87	F (NA)	2.44 (0.52, 11.41)
1.22 Necrotising enterocolitis	8	4,804	F (0%)	1.21 (0.98, 1.51)
1.23 Sepsis	4	2,694	R (31%)	0.83 (0.54, 1.28)
1.24 Hypoglycaemia on NICU admission	1	34	F (NA)	0.63 (0.06, 6.34)
1.25 Poor feeding	1	90	F (NA)	No events
1.26 Patent ductus arteriosus	3	2,536	F (26%)	0.97 (0.80, 1.17)
1.27 Hypotension	2	3,103	F (22%)	1.03 (0.89, 1.19)
1.28 Volume expansion	1	87	F (NA)	**2.03 (1.01, 4.10)**
1.29 Mean blood pressure < 10th centile in the first 24 hours	1	87	F (NA)	1.30 (0.67, 2.53)
1.30 Superior vena cava flow < 41 ml/kg/min in the first 24 hours	1	87	F (NA)	1.22 (0.62, 2.40)
1.31 Right ventricular output < 120 ml/kg/min in the first 24 hours	1	87	F (NA)	1.08 (0.51, 2.30)
1.32 Dobutamine	1	87	F (NA)	1.73 (0.84, 3.57)
1.33 Dopamine	1	87	F (NA)	2.17 (0.62, 7.62)
1.34 Any inotrope	1	87	F (NA)	1.54 (0.82, 2.92)
1.35 Retinopathy of prematurity	1	2,415	F (NA)	0.99 (0.85, 1.14)
1.36 Generalised hypotonicity	1	2,415	F (NA)	1.03 (0.77, 1.37)
1.37 Seizures	4	11,397	F (0%)	0.78 (0.57, 1.06)
1.38 Hyperbilirubinaemia	1	90	F (NA)	2.00 (0.19, 21.28)
1.39 Intraventricular haemorrhage	10	4,891	F (0%)	0.95 (0.85, 1.06)
1.40 Intraventricular haemorrhage, grade 3 or 4	6	3,769	F (19%)	0.81 (0.60, 1.09)
1.41 Periventricular leucomalacia	4	4,225	F (0%)	0.93 (0.68, 1.28)
1.42 Any white matter injury	1	665	F (NA)	0.87 (0.62, 1.22)
1.43 Severe white matter injury	1	688	F (NA)	0.85 (0.55, 1.32)
1.44 Severe white matter injury or death	1	688	F (NA)	0.92 (0.66, 1.28)
1.45 Persistent parenchymal echogenicity	1	8,260	F (NA)	1.09 (0.66, 1.81)
1.46 Echodensity in children born < 32 weeks	1	1,613	F (NA)	**0.38 (0.19, 0.79)**
1.47 Echolucency				
1.47.1 In all children	1	1,776	F (NA)	0.62 (0.37, 1.03)
1.47.2 In children born < 32 weeks	1	1,613	F (NA)	**0.61 (0.38, 0.97)**
1.48 Ventriculomegaly	2	10,036	F (0%)	0.98 (0.68, 1.42)
1.49 Any of echodensity, echolucency, intraventricular haemorrhage, periventricular haemorrhage, or ventriculomegaly				
1.49.1 In all children	1	1,776	F (NA)	0.85 (0.69, 1.06)
1.49.2 In children born < 32 weeks	1	1,613	F (NA)	0.92 (0.78, 1.09)
1.50 Composite adverse outcome	1	1,776	F (NA)	0.62 (0.37, 1.03)
1.51 NICU admission	3	8,519	F (17%)	1.00 (0.95, 1.06)
1.52 Intensive care unit stay (days)	1	120	MD, F (NA)	0.02 (−0.17, 0.21)
1.53 Hospital stay (days)	2	257	MD, R (99%)	−2.75 (–8.92, 3.43)
1.54 Special care baby unit admission > 7 days or death	1	9,024	F (NA)	1.01 (0.95, 1.08)
1.55 Special care baby unit admission > 7 days	1	8,260	F (NA)	1.02 (0.93, 1.11)
1.56 Still in hospital at 6 weeks	1	9,024	F (NA)	0.99 (0.06, 15.80)

Statistically significant effect estimates in bold. Test for heterogeneity represented by *I*^2^ statistic; where *I*^2^ > 30%, summary estimates were calculated using random-effects meta-analysis.

CI, confidence interval; F, fixed-effects; MD, mean difference; NA, not applicable; NICU, neonatal intensive care unit; R, random-effects; RR, risk ratio.

No clear difference was seen between magnesium sulphate and placebo/no treatment for the primary review outcome perinatal death (RR 1.01; 95% CI 0.92 to 1.10; 8 trials, 13,654 babies; analysis 1.1), nor for stillbirth, neonatal death (no obvious asymmetry observed on visual assessment of funnel plot), death later than 28 days but before discharge, early neonatal death, or late neonatal death (Table 1; S1 Appendix). When considering indication for use, the tocolysis subgroup showed an increase in perinatal death (RR 7.99; 95% CI 1.00 to 63.49; 2 trials, 257 babies; analysis 1.1.1) that was not observed in the pre-eclampsia or fetal neuroprotection subgroups. The subgroup interaction test, however, did not indicate a differential effect according to treatment indication (χ^2^ = 4.07, *P* = 0.13, *I*^2^ = 50.8%). For the remaining mortality outcomes, subgroup interaction tests did not indicate differential treatment effects according to indication for administration (see Tables 2 and 3 for effect estimates for individual subgroups and results from subgroup interaction tests).

**Table 2:** Subgroup analyses based on indication for use from randomised controlled trials: Comparison 1—Magnesium sulphate versus placebo or no treatment.

Outcome and subgroup	Studies	Participants	Method (*I*^2^)	RR (95% CI)	χ^2^, *P* value, *I*^2^
**1.1 Perinatal death**					
1.1.1 Tocolysis	2	257	F (NA)	**7.99 (1.00, 63.49)**	4.07, 0.13, 50.8%
1.1.2 Pre-eclampsia	2	9,259	F (0%)	1.01 (0.91, 1.13)	
1.1.3 Fetal neuroprotection	4	4,138	F (0%)	0.96 (0.80, 1.15)	
**1.2 Stillbirth**					
1.2.1 Tocolysis	2	257	F (NA)	5.70 (0.28, 116.87)	1.45, 0.49, 0%
1.2.2 Pre-eclampsia	3	9,961	F (8%)	0.99 (0.87, 1.12)	
1.2.3 Fetal neuroprotection	4	2,122	F (0%)	0.85 (0.40, 1.80)	
**1.3 Neonatal death**					
1.3.1 Tocolysis	4	445	R (61%)	0.78 (0.11, 5.67)	0.48, 0.79, 0%
1.3.2 Pre-eclampsia	2	9,259	R (35%)	1.03 (0.64, 1.65)	
1.3.3 Fetal neuroprotection	5	3,283	R (0%)	0.86 (0.68, 1.08)	
**1.4 Death > 28 days, before discharge**					
1.4.1 Tocolysis	3	412	F (0%)	0.76 (0.19, 3.09)	0.37, 0.83, 0%
1.4.2 Pre-eclampsia	1	9,024	F (NA)	1.13 (0.55, 2.31)	
1.4.3 Fetal neuroprotection	1	1,255	F (NA)	0.88 (0.44, 1.74)	

Statistically significant effect estimates in bold. Test for heterogeneity represented by *I*^2^ statistic; where *I*^2^ > 30%, summary estimates were calculated using random-effects meta-analysis. Result of test subgroup differences represented by χ^2^ statistic, *P* value, and *I*^2^ statistic.

CI, confidence interval; F, fixed-effects; NA, not applicable; R, random-effects; RR, risk ratio.

**Table 3:** Subgroup analyses based on regimen characteristics from randomised controlled trials: Comparison 1—Magnesium sulphate versus placebo or no treatment.

Outcome or subgroup	Studies	Participants	Method (*I*^2^)	RR (95% CI)	χ^2^, *P* value, *I*^2^
**Subgroups based on LD**					
**1.1 Perinatal death**					
1.1.4 4-g IV LD	5	2,259	R (42%)	0.96 (0.62, 1.49)	0.57, 0.75, 0%
1.1.5 6-g IV LD	1	2,136	R (NA)	1.12 (0.85, 1.47)	
1.1.6 4-g IV and 10-g IM LD	2	9,259	R (0%)	1.01 (0.91, 1.12)	
**1.2 Stillbirth**					
1.2.4 4-g IV LD	6	2,961	F (0%)	1.25 (0.85, 1.84)	1.57, 0.21, 36.1%
1.2.5 6-g IV LD	1	120	F (NA)	No events	
1.2.6 4-g IV and 10-g IM LD	2	9,259	F (0%)	0.96 (0.84, 1.10)	
**1.3 Neonatal death**					
1.3.4 4-g IV LD	6	2,294	R (7%)	0.86 (0.64, 1.16)	0.44, 0.80, 0%
1.3.5 6-g IV LD	3	1,434	R (2%)	0.83 (0.48, 1.44)	
1.3.6 4-g IV and 10-g IM LD	2	9,259	R (35%)	1.03 (0.64, 1.65)	
**1.4 Death > 28 days, before discharge**					
1.4.4 4-g IV LD	3	1,514	F (0%)	0.81 (0.43, 1.53)	0.81, 0.67, 0%
1.4.5 6-g IV LD	1	153	F (NA)	2.47 (0.10, 59.70)	
1.4.6 4-g IV and 10-g IM LD	1	9,024	F (NA)	1.13 (0.55, 2.31)	
**Subgroups based on MD**					
**1.1 Perinatal death**					
1.1.7 LD only	2	747	R (0%)	0.92 (0.59, 1.44)	2.73, 0.44, 0%
1.1.8 1-g/hour IV MD	1	1,255	R (NA)	0.81 (0.60, 1.09)	
1.1.9 2–5-g/hour IV MD	3	2,393	R (71%)	2.27 (0.35, 14.55)	
1.1.10 5-g/4-hour IM MD	2	9,259	R (0%)	1.01 (0.91, 1.12)	
**1.2 Stillbirth**					
1.2.7 LD only	2	747	F (0%)	0.96 (0.22, 4.17)	2.50, 0.48, 0%
1.2.8 1-g/hour IV MD	2	1,957	F (9%)	1.22 (0.81, 1.83)	
1.2.9 2–5-g/hour IV MD	3	377	F (0%)	5.70 (0.28, 116.87)	
1.2.10 5-g/4-hour IM MD	2	9,259	F (0%)	0.96 (0.84, 1.10)	
**1.3 Neonatal death**					
1.3.7 LD only	2	747	R (0%)	0.93 (0.58, 1.47)	0.72, 0.87, 0%
1.3.8 1-g/hour IV MD	1	1,255	R (NA)	0.81 (0.59, 1.11)	
1.3.9 2–5-g/hour IV MD	6	1,726	R (41%)	0.84 (0.30, 2.33)	
1.3.10 5-g/4-hour IM MD	2	9,259	R (35%)	1.03 (0.64, 1.65)	
**1.4 Death > 28 days, before discharge**					
1.4.7 1-g/hour IV MD	1	1,255	F (NA)	0.88 (0.44, 1.74)	0.37, 0.83, 0%
1.4.8 2–5-g/hour IV MD	3	412	F (0%)	0.76 (0.19, 3.09)	
1.4.9 5-g/4-hour IM MD	1	9,024	F (NA)	1.13 (0.55, 2.31)	

Test for heterogeneity represented by *I*^2^ statistic; where *I*^2^ > 30%, summary estimates were calculated using random-effects meta-analysis. Result of test subgroup differences represented by χ^2^ statistic, *P* value, and *I*^2^ statistic.

CI, confidence interval; F, fixed-effects; g, gram; IM, intramuscular; IV, intravenous; LD, loading dose; MD, maintenance dose; NA, not applicable; R, random-effects; RR, risk ratio.

Babies exposed to antenatal magnesium sulphate had a 67% relative increase in the risk of having an Apgar score less than 7 at 1 minute (RR 1.67; 95% CI 1.02 to 2.73; 2 trials, 199 babies; analysis 1.7), and over 2 times the risk of need for volume expansion compared with babies not exposed (RR 2.03; 95% CI 1.01 to 4.10; 1 trial, 87 babies; analysis 1.28). A subgroup of babies born less than 32 weeks gestation exposed to antenatal magnesium sulphate had a 62% relative reduction in the risk of intracerebral echodensity, compared with babies not exposed (RR 0.38; 95% CI 0.19 to 0.79; 1 trial, 1,613 babies; analysis 1.46). While a difference in intracerebral echolucency was not observed in all babies, a 39% relative reduction was seen for babies born less than 32 weeks exposed to antenatal magnesium sulphate (RR 0.61; 95% CI 0.38 to 0.97; 1 trial, 1,613 babies; analysis 1.47.2) (Table 1; S1 Appendix).

There were no clear differences between magnesium sulphate and placebo/no treatment for all remaining secondary outcomes reported (Table 1; S1 Appendix).

### Lower versus higher dose regimens

This comparison included 8 trials, with 6 assessing magnesium sulphate for treatment of eclampsia or severe pre-eclampsia [22,23,44,52,56,59], and 2 for the prevention of preterm birth (tocolysis) [26,60]. Regimens assessed varied: lower dose regimens included a 4- to 10-gram loading dose with a 0.625- to 2-gram-per-hour maintenance dose; higher dose regimens assessed included a 4- to 14-gram loading dose with a 1.25- to 5-gram-per-hour maintenance dose (see Table 4 and S1 Appendix for effect estimates and forest plots).

**Table 4:** Adverse outcome estimates from randomised controlled trials: Comparison 2—Lower versus higher dose regimens of magnesium sulphate.

Outcome	Studies	Participants	Method (*I*^2^)	RR (95% CI)
2.1 Perinatal death	6	543	F (0)	1.01 (0.75, 1.36)
2.2 Stillbirth	5	471	F (0)	0.94 (0.61, 1.45)
2.3 Neonatal death	6	535	F (0)	1.12 (0.57, 2.22)
2.4 Apgar score < 7 at 1 minute	3	302	F (0)	0.96 (0.68, 1.35)
2.5 Apgar score < 7 at 5 minutes	3	302	R (35%)	1.41 (0.54, 3.65)
2.6 Resuscitation	1	64	F (NA)	1.00 (0.22, 4.59)
2.7 Respiratory distress syndrome	2	154	R (53%)	1.97 (0.76, 5.15)
2.8 Respiratory depression	1	50	F (NA)	0.33 (0.04, 2.99)
2.9 Respiratory disorders	1	64	F (NA)	1.08 (0.87, 1.33)
2.10 Mechanical ventilation	1	64	F (NA)	2.00 (0.39, 10.16)
2.11 Bradycardia	1	104	F (NA)	3.85 (0.45, 33.29)
2.12 Jaundice	1	50	F (NA)	1.25 (0.38, 4.12)
2.13 Hypoglycaemia	1	104	F (NA)	0.96 (0.06, 14.98)
2.14 Hypocalcaemia	1	104	F (NA)	2.89 (0.12, 69.32)
2.15 Hypotonia	1	50	F (NA)	0.14 (0.02, 1.08)
2.16 Requirement for calcium gluconate	1	50	F (NA)	0.25 (0.06, 1.06)
2.17 NICU admission	5	409	F (6%)	**1.75 (1.06, 2.88)**
2.18 NICU stay (days)	1	104	MD, F (NA)	**3.10 (0.78, 5.42)**

Statistically significant effect estimates in bold. Test for heterogeneity represented by *I*^2^ statistic; where *I*^2^ > 30%, summary estimates were calculated using random-effects meta-analysis.

CI, confidence interval; F, fixed-effects; MD, mean difference; NA, not applicable; NICU, neonatal intensive care unit; R, random-effects; RR, risk ratio.

No clear differences between the lower and higher dose regimens of magnesium sulphate were seen for the primary review outcome perinatal death (RR 1.01; 95% CI 0.75 to 1.36; 6 trials, 543 babies; analysis 2.1), nor for stillbirth or neonatal death (Table 4; S1 Appendix). For all mortality outcomes, subgroup interaction tests did not indicate differential treatment effects according to indication for administration of antenatal magnesium sulphate (see Table 5 for effect estimates for individual subgroups and results from subgroup interaction tests).

**Table 5:** Subgroup analyses based on indication for use from randomised controlled trials: Comparison 2—Lower versus higher dose regimens of magnesium sulphate.

Outcome and subgroup	Studies	Participants	Method (*I*^2^)	RR (95% CI)	χ^2^, *P* value, *I*^2^
**2.1 Perinatal death**					
2.1.1 Tocolysis	1	104	F (NA)	2.25 (0.61, 8.21)	1.63, 0.20, 38.6%
2.1.2 Pre-eclampsia/eclampsia	5	439	F (0%)	0.94 (0.70, 1.28)	
**2.2 Stillbirth**					
2.2.1 Tocolysis	1	104	F (NA)	0.96 (0.06, 14.98)	0.00, 0.99, 0%
2.2.2 Pre-eclampsia/eclampsia	4	367	F (0%)	0.94 (0.60, 1.46)	
**2.3 Neonatal death**					
2.3.1 Tocolysis	1	104	F (NA)	2.89 (0.61, 13.65)	1.99, 0.16, 49.6%
2.3.2 Pre-eclampsia/eclampsia	5	431	F (0%)	0.82 (0.37, 1.82)	

Test for heterogeneity represented by *I*^2^ statistic; where *I*^2^ > 30%, summary estimates were calculated using random-effects meta-analysis. Result of test subgroup differences represented by χ^2^ statistic, *P* value, and *I*^2^ statistic.

CI, confidence interval; F, fixed-effects; NA, not applicable; RR, risk ratio.

Babies exposed to the lower dose versus higher dose regimens of magnesium sulphate had an increased risk of neonatal intensive care unit admission (RR 1.75; 95% CI 1.06 to 2.88; 5 trials, 409 babies; analysis 2.17). On average, babies exposed to the lower dose regimen had a longer duration of stay in the neonatal intensive care unit compared with those exposed to the higher dose regimen (MD 3.10 days; 95% CI 0.78 to 5.42; 1 trial, 104 babies; analysis 2.18) (Table 4; S1 Appendix).

No clear differences were seen between the lower and higher dose regimens of magnesium sulphate for the remaining secondary outcomes reported (Table 4; S1 Appendix).

### IM versus IV maintenance dose

This comparison included 5 trials, all assessing magnesium sulphate for the prevention or treatment of eclampsia [27,31,49,55,58]. Regimens assessed included Bhattacharjee’s regimen (4-gram IV loading dose; 6-gram IV maintenance dose every 8 hours), Dhaka regimen (4-gram IV and 6-gram IM loading dose; 2.5-gram IM maintenance dose every 4 hours), Pritchard’s regimen (4-gram IV and 10-gram IM loading dose; 5-gram IM maintenance dose every 4 hours), Zuspan’s regimen (4-gram IV loading dose; 1-gram IV maintenance dose every hour), and Sibai’s regimen (6-gram IV loading dose; 2-gram IV maintenance dose every hour) (all maintenance doses were for 24 hours after birth or last seizure) (see Table 6 and S1 Appendix for effect estimates and forest plots).

**Table 6:** Adverse outcome estimates from randomised controlled trials: Comparisons 3–8.

Outcome and subgroup	Studies	Participants	Method (*I*^2^)	RR (95% CI)
**Comparison 3: IM versus IV maintenance dose of magnesium sulphate (pre-eclampsia/eclampsia)**				
3.1 Perinatal death				
3.1.1 Pritchard’s versus Zuspan’s regimen	2	353	F (0%)	0.94 (0.66, 1.32)
3.1.2 Pritchard’s versus Sibai’s regimen	1	115	F (NA)	0.90 (0.53, 1.53)
3.1.3 Pritchard’s versus Bhattacharjee’s regimen	1	107	F (NA)	1.28 (0.61, 2.65)
3.1.4 Dhaka versus Zuspan’s regimen	1	41	F (NA)	0.57 (0.16, 2.08)
3.2 Stillbirth				
3.2.1 Pritchard’s versus Zuspan’s regimen	1	114	F (NA)	0.79 (0.44, 1.40)
3.2.2 Pritchard’s versus Sibai’s regimen	2	133	F (0%)	0.80 (0.46, 1.41)
3.2.3 Pritchard’s versus Bhattacharjee’s regimen	1	107	F (NA)	1.18 (0.38, 3.63)
3.2.4 Dhaka versus Zuspan’s regimen	1	41	F (NA)	0.24 (0.03, 1.95)
3.3 Neonatal death				
3.3.1 Pritchard’s versus Zuspan’s regimen	1	114	F (NA)	2.52 (0.27, 23.47)
3.3.2 Pritchard’s versus Sibai’s regimen	1	115	F (NA)	2.56 (0.27, 23.93)
3.3.3 Pritchard’s versus Bhattacharjee’s regimen	1	107	F (NA)	1.37 (0.47, 4.06)
3.3.4 Dhaka versus Zuspan’s regimen	1	41	F (NA)	1.90 (0.19, 19.40)
3.4 Apgar score < 7 at 1 minute				
3.4.1 Dhaka versus Zuspan’s regimen	1	41	F (NA)	0.32 (0.10, 1.01)
3.5 Apgar score < 7 at 5 minutes				
3.5.1 Dhaka versus Zuspan’s regimen	1	41	F (NA)	0.38 (0.08, 1.74)
3.6 Respiratory distress syndrome				
3.6.1 Dhaka versus Zuspan’s regimen	1	41	F (NA)	0.76 (0.24, 2.44)
3.7 Jaundice				
3.7.1 Dhaka versus Zuspan’s regimen	1	41	F (NA)	0.76 (0.24, 2.44)
3.8 Hypotonia				
3.8.1 Dhaka versus Zuspan’s regimen	1	41	F (NA)	0.48 (0.10, 2.32)
3.9 NICU admission				
3.9.1 Pritchard’s versus Zuspan’s regimen	1	114	F (NA)	0.98 (0.35, 2.73)
3.9.2 Pritchard’s versus Sibai’s regimen	1	115	F (NA)	1.00 (0.36, 2.78)
3.9.3 Dhaka versus Zuspan’s regimen	1	41	F (NA)	0.76 (0.24, 2.44)
**Comparison 4: Loading dose versus loading and maintenance doses of magnesium sulphate (pre-eclampsia/eclampsia)**				
4.1 Perinatal death	3	632	R (63%)	0.94 (0.33, 2.72)
4.2 Stillbirth	3	803	F (26%)	1.10 (0.77, 1.58)
4.3 Neonatal death	2	462	F (0%)	0.78 (0.43, 1.41)
4.4 Neonatal death < 7 days	1	402	F (NA)	0.73 (0.30, 1.77)
4.5 Apgar score < 7 at 0 minutes	1	52	F (NA)	1.07 (0.32, 3.54)
4.6 Apgar score < 7 at 1 minute	1	52	F (NA)	0.86 (0.06, 12.98)
4.7 Apgar score < 7 at 5 minutes	2	406	F (NA)	1.61 (0.72, 3.62)
4.8 NICU admission for respiratory distress	2	397	F (0%)	1.02 (0.63, 1.65)
4.9 NICU admission for early onset sepsis	1	80	F (NA)	1.00 (0.06, 15.44)
4.10 NICU admission for late onset sepsis	1	80	F (NA)	3.00 (0.13, 71.51)
4.11 NICU admission for meconium aspiration syndrome	1	80	F (NA)	1.00 (0.06, 15.44)
4.12 NICU admission for birth asphyxia	1	80	F (NA)	0.33 (0.01, 7.95)
4.13 NICU admission	3	435	F (0%)	0.94 (0.77, 1.15)
**Comparison 5: Serial intravenous boluses versus continuous intravenous maintenance of magnesium sulphate (pre-eclampsia)**				
5.1 Perinatal death	1	197	F (NA)	0.44 (0.08, 2.34)
5.2 Stillbirth	1	197	F (NA)	0.29 (0.01, 7.09)
5.3 Neonatal death	1	197	F (NA)	0.58 (0.10, 3.42)
5.4 Intubated at birth	1	197	F (NA)	0.88 (0.29, 2.62)
5.5 Mechanical ventilation	1	197	F (NA)	0.44 (0.11, 1.70)
5.6 Bradycardia (<110 bpm)	1	197	F (NA)	0.44 (0.08, 2.34)
5.7 Special care baby unit admission	1	197	F (NA)	0.84 (0.53, 1.35)
**Comparison 6: Short versus standard maintenance course of magnesium sulphate (eclampsia)**				
6.1 Stillbirth	1	98	F (NA)	0.87 (0.41, 1.82)
6.2 Birth asphyxia	1	98	F (NA)	0.83 (0.24, 2.92)
**Comparison 7: Slower versus standard rate of loading dose of magnesium sulphate (fetal neuroprotection)**				
7.1 Stillbirth	1	51	F (NA)	0.35 (0.01, 8.12)
**Comparison 8: Weaning versus no weaning of magnesium sulphate (tocolysis)**				
8.1 Apgar score < 7 at 5 minutes	1	141	F (NA)	0.65 (0.22, 1.90)

Test for heterogeneity represented by *I*^2^ statistic; where *I*^2^ > 30%, summary estimates were calculated using random-effects meta-analysis. Bhattacharjee’s regimen: 4-g IV LD; 6-g IV/8 hours MD. Dhaka regimen: 4-g IV and 6-g IM LD; 2.5-g IM/4-hour MD. Pritchard’s regimen: 4-g IV and 10-g IM LD; 5-g IM/4-hour MD. Sibai’s regimen: 6-g IV LD; 2-g IV/hour MD. Zuspan’s regimen: 4-g IV LD; 1-g IV/hour MD. All MDs for 24 hours after birth/last seizure.

bpm, beats per minute; CI, confidence interval; F, fixed-effects; g, gram; IM, intramuscular; IV, intravenous; LD, loading dose; MD, maintenance dose; NA, not applicable; NICU, neonatal intensive care unit; R, random-effects; RR, risk ratio.

No clear differences were observed for

- Pritchard’s versus Zuspan’s regimen for the primary review outcome perinatal death (RR 0.94; 95% CI 0.66 to 1.32; 2 trials, 353 babies; analysis 3.1.1), nor for stillbirth or neonatal death;
- Pritchard’s versus Sibai’s regimen for the primary review outcome perinatal death (RR 0.90; 95% CI 0.53 to 1.53; 1 trial, 115 babies; analysis 3.1.2), nor for stillbirth or neonatal death;
- Pritchard’s versus Bhattacharjee’s regimen for the primary review outcome perinatal death (RR 1.28; 95% CI 0.61 to 2.65; 1 trial, 107 babies; analysis 3.1.3), nor for stillbirth or neonatal death;
- Dhaka versus Zuspan’s regimen for the primary review outcome perinatal death (RR 0.57; 95% CI 0.16 to 2.08; 1 trial, 41 babies; analysis 3.1.4), nor for stillbirth or neonatal death (Table 6; S1 Appendix).

No clear differences were observed for Pritchard’s versus Zuspan’s regimen, Pritchard’s versus Sibai’s regimen, or Dhaka versus Zuspan’s regimen for the remaining secondary outcomes reported (Table 6; S1 Appendix).

### Loading dose versus loading and maintenance doses

This comparison included 5 trials, all assessing magnesium sulphate for the prevention or treatment of eclampsia [25,41,50,53,57]. Trials compared a cumulative 8-, 10-, or 14-gram loading dose (4 grams IV and 4 to 10 grams IM) with Dhaka or Pritchard’s regimen (see descriptions above; and see Table 6 and S1 Appendix for effect estimates and forest plots).

No clear differences for loading dose only versus loading and maintenance dose regimens were seen for the primary review outcome perinatal death (average RR 0.94; 95% CI 0.33 to 2.72; 3 trials, 632 babies; analysis 4.1), nor for stillbirth, neonatal death, or neonatal death at less than 7 days (Table 6; S1 Appendix).

No clear differences for loading dose only versus loading and maintenance dose regimens were seen for the remaining secondary outcomes reported (Table 6; S1 Appendix).

### Serial IV boluses versus continuous IV maintenance dose

This comparison included 1 trial, assessing magnesium sulphate for the treatment of severe pre-eclampsia, and compared a serial intravenous bolus regimen (6-gram IV loading dose, and 2-gram IV bolus over 10 minutes every 2 hours as maintenance) with a continuous infusion (4-gram IV loading dose, and 1-gram-per-hour continuous IV maintenance dose) [37] (see Table 6 and S1 Appendix for effect estimates and forest plots).

No clear differences between the serial bolus and continuous infusion regimens were seen for the primary review outcome perinatal death (RR 0.44; 95% CI 0.08 to 2.34; 1 trial, 197 babies; analysis 5.1), nor for stillbirth or neonatal death (Table 6; S1 Appendix).

No clear differences between the serial bolus and continuous infusion regimens were seen for the remaining secondary outcomes reported (Table 6; S1 Appendix).

### Short versus standard maintenance course

This comparison included 1 trial, assessing magnesium sulphate for the treatment of eclampsia. Both groups received a 4-gram IV and 10-gram IM loading dose, followed by either a short maintenance course (2 doses of 5 grams IM 4 hours apart after birth or last seizure) or a standard maintenance course (5 grams IM every 4 hours for 24 hours after birth or last seizure) [29].

No clear difference between the short and standard maintenance course regimens was seen for stillbirth. No clear difference between the short and standard maintenance course regimens was seen for the only other secondary outcome reported, birth asphyxia (see Table 6 and S1 Appendix for effect estimates and forest plots).

### Slower versus standard rate of loading dose

This comparison included 1 trial, assessing magnesium sulphate for fetal neuroprotection, and compared a slower (over 60 minutes) versus standard (over 20 minutes) rate of administering a 4-gram IV loading dose of magnesium sulphate (all women received a 1-gram-per-hour maintenance dose for 24 hours or until birth) [24].

No clear difference between a slower and standard rate of loading dose administration was seen for stillbirth (see Table 6 and S1 Appendix for effect estimate and forest plot).

### Weaning versus no weaning

This comparison included 1 trial, assessing magnesium sulphate for the prevention of preterm birth (tocolysis), and compared weaning (by 1 gram IV per 4 hours) versus not weaning magnesium sulphate (all women received a 6-gram IV loading dose, and 2- to 3.5-gram IV maintenance dose per hour until tocolysis was achieved) [42].

No clear difference between weaning and no weaning was seen for Apgar score less than 7 at 5 minutes (see Table 6 and S1 Appendix for effect estimate and forest plot).

### Evidence from non-randomised comparative studies

One hundred thirty-eight non-randomised studies were included: 5 non-randomised trials, 35 prospective cohort studies (7 with nested case–control analyses), 82 retrospective cohort studies (16 with nested case–control analyses), 8 non-concurrent cohort studies, and 8 case–control studies [62–199]. The characteristics of the studies and risk of bias assessments are detailed in S1 and S2 Tables.

There was substantial variation in the characteristics of these studies regarding participants, indications for use of magnesium sulphate (prevention or treatment of eclampsia: 30 studies; prevention of preterm birth (tocolysis): 28 studies; fetal neuroprotection: 25 studies; combination of aforementioned indications: 38 studies; unclear: 17 studies), comparison groups, outcomes assessed (and their definitions), and analysis methods employed. Methodological quality (specifically in relation to the risk of bias for reported review outcomes of interest) also differed across the studies, with overall judgements of unclear (specifically when only abstracts were available), high, moderate to high, and moderate risk of bias assigned to 43 studies, 49 studies, 35 studies, and 11 studies, respectively. The most common concerns across studies related to the potential for confounding (with no attempt to balance allocation between groups or match groups, and/or important confounding variables not taken into account in relevant outcome analyses), detection bias (with the consistent implementation of valid and reliable measures being unclear, and/or the absence of blinding of exposure or outcome assessors), and performance bias (with protocols not available to assess important variations).

The primary review outcome, perinatal death, was reported by 11 of the 138 non-randomised studies, 10 of which showed either a possible reduction (or lower rate) or no clear difference (or similar rate) in perinatal death among babies exposed to magnesium sulphate compared with no magnesium sulphate or a different magnesium sulphate regimen. A possible increase in perinatal death, specifically among babies exposed to >48 versus ≤48 grams of magnesium sulphate for tocolysis was shown in in the 11th study (retrospective cohort of 127 babies, moderate to high risk of bias) [183]. See Table 7 and S3 Table for summaries of individual study results.

**Table 7:** Perinatal death from non-randomised studies.

Study; design	Participants	Comparisons	Results summary
Adama-Hondegla 2013; RCS with CCS(N)	178 babies born to women with eclampsia	(1) Babies living at seventh day of life, *N* = 147 babies, versus (2) stillbirths and neonatal deaths in first 7 days, *N* = 31 babies	MgSO_4_ exposure: aOR 1.04, *P* > 0.05
Alexander 2006; PCS	87 babies born to women with eclampsia	(1) No gestational hypertension, no MgSO_4_, *N* = 49 babies, versus (2) gestational hypertension, MgSO_4_, *N* = 11 babies, versus (3) gestational hypertension, no MgSO_4_, *N* = 27 babies	Perinatal death: 6.1% (3/49) versus 0% (0/11) versus 11.1% (3/27)
Cawyer 2019; RCS	2,468 babies born to women with pre-eclampsia >32 weeks GA	(1) MgSO_4_, *N* = 1,353 babies, versus (2) no MgSO_4_, *N* = 1,115 babies	Perinatal or neonatal death: 0.1% (2/1,353) versus 0.2% (2/1,115), *P* = 1.00
Chowdhury 2009; PCS	529 babies born to women with eclampsia	(1) MgSO_4_ Pritchard’s regimen, *N* = 406 babies, versus (2) MgSO_4_ low dose IV regimen, *N* = 123 babies	Perinatal death: OR 1.58, 95% CI 0.93–2.61, *P* = 0.075
Jung 2018; RCS	184 babies born to women with ROM <32 weeks GA	(1) MgSO_4_ for tocolysis, *N* = 143 babies, versus (2) no MgSO_4_, *N* = 41 babies	Perinatal death: Overall: 7.0% (10/143) versus 19.5% (8/41), *P* = 0.0375; ROM at 23 to 27+6 weeks GA: 14.3% (9/63) versus 36.8% (7/19), *P* = 0.0651; ROM at 28 to 31+6 weeks GA: 1.25% (1/80) versus 4.5% (1/22), *P* = 0.9051
Kamilya 2005; CCS(N)	1,205 babies born to women with eclampsia	(1) Birth year 2002–2004 (almost universal MgSO_4_), *N* = 481 babies, versus (2) birth year 1995–1997 (no MgSO_4_), *N* = 724 babies	Perinatal death: 24.3% (117/481) versus 54.8% (397/724)
**Kamyar 2016a**; RCS (secondary analysis RCT)	396 babies born to women with intrapartum clinical chorioamnionitis	(1) MgSO_4_ for fetal neuroprotection, *N* = 192 babies, versus (2) placebo, *N* = 204 babies	Stillbirth or death by 1 year: Overall: aRR 1.68, 95% CI 0.85–3.32; ≤28 weeks GA: aRR 1.34, 95% CI 0.47–2.73
Mitani 2011; RCS	425 babies born between 22 and 31 weeks GA	(1) MgSO_4_ for tocolysis, *N* = 236 babies, versus (2) no MgSO_4_, *N* = 189 babies	Perinatal death: 5.5% (13/236) versus 9.0% (17/189), *P* = 0.185
Okusanya 2012; NRT	103 babies born to women with severe pre-eclampsia or eclampsia	Severe pre-eclampsia: (1) 10-g MgSO_4_ LD, *N* = 25 babies, versus (2) 14-g MgSO_4_ LD, *N* = 30 babies	Perinatal death, unclear reporting: Severe pre-eclampsia: (1) PMR 240 per 1,000 (6 deaths) versus (2) PMR 35 per 1,000 (1 death)
		Eclampsia: (1) 10-g MgSO_4_ LD, *N* = 29 babies, versus (2) 14-g MgSO_4_ LD, *N* = 19 babies	Perinatal death, unclear reporting: Eclampsia: (1) PMR 241 per 1,000 (6 deaths, all IUFD) versus (2) 0 deaths
**Scudiero 2000**; RCS with CCS(N)	127 babies born between 700 and 1,249 g, to women who received MgSO_4_ for tocolysis	(1) Perinatal deaths, *N* = 18 babies, versus (2) survivors, *N* = 109 babies	MgSO_4_ > 48 g: 72.2% (13/18) versus 45.0% (49/109), *P* = 0.03; MgSO_4_ ≤ 48 g versus >48 g (multivariable model): OR 4.72, 95% CI 1.12 to 19.97, *P* = 0.035
		(1) MgSO_4_ ≤ 24 g, *N* = 43 babies, versus (2) MgSO_4_ > 24 to ≤ 48 g, *N* = 25 babies, versus (3) MgSO_4_ > 48 g, *N* = 59 babies	Perinatal death (Cochrane–Armitage trend test, 1 versus 2 versus 3): 7.0% (3/43) versus 8.0% (2/25) versus 22.0% (13/59), *P* = 0.03; perinatal death (1 versus 2): 7.0% (3/43) versus 8.0% (2/25), *P* = 1.0
Young 1977; NRT	144 babies born to women with pre-eclampsia or eclampsia	(1) MgSO_4_ IV bolus MD (2 g over 10 minutes every 1–2 hours), *N* = 97 babies, versus (2) MgSO_4_ continuous IV MD (1 g per hour), *N* = 47 babies	Perinatal death: 2.1% (2/97) versus 2.1% (1/47)

The bold studies were judged to be of higher quality (moderate to high risk of bias) and presented results adjusted for confounders for the relevant outcome; other studies were judged to be at high or unclear risk of bias and/or did not present adjusted results for the relevant outcome.

aOR, adjusted odds ratio; aRR, adjusted risk ratio; CCS(N), nested case–control study; CI, confidence interval; g, gram; GA, gestational age; IUFD, intrauterine fetal demise; IV, intravenous; LD, loading dose; MD, maintenance dose; MgSO_4_, magnesium sulphate; NRT, non-randomised trial; OR, odds ratio; PCS, prospective cohort study; PMR, perinatal mortality ratio; RCS, retrospective cohort study; RCT, randomised controlled trial; ROM, rupture of the membranes.

For the majority of secondary pre-specified and non-pre-specified adverse neonatal outcomes reported, the results from non-randomised studies were consistent with those observed in randomised controlled trials, with no clear differences (and in some cases, possible benefits of magnesium sulphate) observed. The direction of the findings (no clear difference, possible benefit, possible harm, or mixed) from the non-randomised studies are summarised in Table 8, with the detailed individual study results provided in S3 Table.

**Table 8:** Summary of outcomes from non-randomised studies.

Outcome	Direction of effect for magnesium sulphate versus no magnesium sulphate or a different regimen		
	Studies showing no clear difference	Studies showing possible benefit	Studies showing possible harm
Stillbirth	Brazy 1982; Chowdhury 2009*; Jung 2018^	Jung 2018^; Shamsuddin 2005*	Das 2015*
Neonatal death or death before discharge	Alston 2016; Ambadkar 2019; Basu 2012; Bertello Grecco 2019*; Brazy 1982; Canterino 1999; Chowdhury 2009*; De Jesus 2015; del Moral 2007; de Veciana 1995; Drassinower 2015; Elimian 2002*; Elliott 2003; **Farkouh 2001**; Gibbins 2013; Girsen 2015; Gonzalez-Quintero 2001; Hechtman 2002*; Hong 2019; James 2015; Jazayeri 2003; Jung 2018; Kamyar 2015a; Kamyar 2015b; **Kamyar 2016a**; **Kamyar 2016b**^; **Kimberlin 1998**; Lee 2013; Lloreda-Garcia 2016; Mikhael 2019*; Morag 2016; Narasimhulu 2017; Nassar 2006*; Özlü 2019; **Paneth 1997**; Rantonen 2001; **Shalabi 2017**^; Shokry 2010; Suh 2015; **Weisz 2015**^; Whitsel 2004; Yokoyama 2010	**Downey 2017**; Garcia Alonso 2018; **Grether 1998**; **Shalabi 2017**^; **Stockley 2018; Weisz 2015**^	Das 2015*;**Kamyar 2016b**^; Lipsitz 1971*; Rattray 2014; Rauf 2017
Apgar score < 7 at 1 minute (or ≤ 5)	Chun 2014^; Gibbins 2013; Lloreda-Garcia 2016; Mitani 2011; Morag 2016; Narasimhulu 2017		Chun 2014^; Das 2015*; Girsen 2015; Lipsitz 1971*
Apgar score < 7 at 5 minutes (or ≤5 or <6)	Canterino 1999; Chun 2014^; Cuff 2018*; de Veciana 1995; Drassinower 2015; Elimian 2002*; Gibbins 2013; Jung 2018; Lloreda-Garcia 2016; McPherson 2014*; Mitani 2011; Narasimhulu 2017; Nassar 2006*; Nelson 1995; Okusanya 2012*; Rhee 2012; Schanler 1997; Stockley 2018; Weisz 2015*	Jeanneteau 2014; Shalabi 2017	Chun 2014^; Das 2015*; Girsen 2015; Lipsitz 1971*; Morag 2016
Birth asphyxia	McGuiness 1980	Shamsuddin 2005*	
Meconium at birth	Greenberg 2013; Jazayeri 2003		
Intubation	Basu 2012; **De Jesus 2015**; Derks 2016; Morag 2016; Narasimhulu 2017; Weisz 2015*^	**Bajaj 2018;** Drassinower 2015; Weisz 2015^	Das 2015*; Rauf 2017; Weisz 2015*^
Intubation (duration)	de Veciana 1995	O Reilly 2016^	O Reilly 2016*^
Resuscitation	Basu 2012; Brookfield 2015*; De Jesus 2015; **Garcia Alonso 2018**; Gibbins 2013; Lloreda-Garcia 2016; McPherson 2014*; Narasimhulu 2017; Özlü 2019; Weisz 2015*^	Bajaj 2018; **De Silva 2018**	Lipsitz 1971*; Weisz 2015^
Oxygen bag, mask, or both (resuscitation)	**Bajaj 2018**; Drassinower 2015; Riaz 1998; Weisz 2015*^	Weisz 2015*^	
Chest compressions (resuscitation)	Drassinower 2015; Stockley 2018; Weisz 2015*^	**Bajaj 2018**; Weisz 2015^	
Adrenaline (resuscitation)	Stockley 2018; Weisz 2015*^	Jeanneteau 2014; Weisz 2015^	
Score for Neonatal Acute Physiology > 10 or 20 in first 24 hours	Stockley 2018; Weisz 2015^	**Deering 2005**; Shalabi 2017; Weisz 2015*^	
Delayed adaptation	Lai 2017; Riaz 1998		Brazy 1982
Respiratory distress syndrome	Alston 2016; Ambadkar 2019; Bozkurt 2016; Brookfield 2016; Canterino 1999; De Jesus 2015; de Veciana 1995^; Drassinower 2015; Elimian 2002*; Girsen 2015; Gonzalez-Quintero 2001; Gursoy 2015; Imamoglu 2014; Jazayeri 2003; Jung 2018; Kamyar 2015a; Lee 2013; McPherson 2014*; Mitani 2011; Rantonen 2001; Schanler 1997; Shokry 2010; Suh 2015; Yokoyama 2010	de Veciana 1995^; Özlü 2019	
Respiratory depression	Bertello Grecco 2019*		Das 2015*
Surfactant use	delValle 1998; Elimian 2002*; **Garcia Alonso 2018**; Lloreda-Garcia 2016; Rantonen 2001; Shokry 2010; Weisz 2015*		
Ventilation	Brookfield 2016; **De Jesus 2015**^; Drassinower 2015; Garcia Alonso 2018; Girsen 2015; Havranek 2011; James 2015; Lee 2013; Lloreda-Garcia 2016^; McPherson 2014*; Nunes 2018; Özlü 2019; Rantonen 2001; Schanler 1997; Shokry 2010	**De Jesus 2015**^; Lloreda-Garcia 2016^; Rauf 2017^; Shalabi 2017	Lipsitz 1971*; Lloreda-Garcia 2016^; Rauf 2017^
Ventilation (duration)	Black 2006; De Jesus 2015; Kimberlin 1998; Özlü 2019; Suh 2015		
Methylxanthine use or duration	Black 2006; Havranek 2011; Imamoglu 2014; Schanler 1997		
Chronic lung disease or bronchopulmonary dysplasia	Alston 2016; Basu 2012; Bozkurt 2016; De Jesus 2015; **Edwards 2018**; **Garcia Alonso 2018**; James 2015; Jung 2018; Kamyar 2015a; **Kamyar 2016a**; McPherson 2014*; Özlü 2019; **Shalabi 2017**; **Stockley 2018;** Suh 2015; **Weisz 2015**		Narasimhulu 2017; Stetson 2019
Oxygen use (at 28 days, 36 weeks, or discharge)	**Kimberlin 1998**; Morag 2016; Schanler 1997		
Oxygen use (duration)	De Jesus 2015; Özlü 2019; Suh 2015		
Steroid use (dexamethasone or hydrocortisone)	Mikhael 2019*; Rantonen 2001; Rattray 2014; Shalabi 2017		
Apnoea	Bozkurt 2016; Riaz 1998; Wutthigate 2017*		
Pulmonary haemorrhage	De Jesus 2015; James 2015		
Necrotising enterocolitis	Alston 2016; Bozkurt 2016; Brazy 1982; De Jesus 2015; delValle 1998; de Veciana 1995; **Downey 2017**; **Edwards 2018**; Elimian 2002*; Elliott 2003; Garcia Alonso 2018; **Ghidini 2001**; Gursoy 2015; Hong 2019; James 2015; Jazayeri 2003; Jung 2018; Kamyar 2015a; **Kamyar 2016a**; **Kamyar 2016b**; **Kimberlin 1998**; Lee 2013; Lloreda-Garcia 2016; McPherson 2014*; Mikhael 2019*; Morag 2016; Narasimhulu 2017; Özlü 2019; Schanler 1997; **Shalabi 2017**; **Stockley 2018;** Suh 2015; **Weisz 2015**; Yokoyama 2010	Moschos 2001; Wiswell 1996	
Spontaneous intestinal perforation	**Downey 2017**; Mikhael 2019*; **Shalabi 2017**		Rattray 2014
Composite of necrotising enterocolitis/spontaneous intestinal perforation or death	Kamyar 2016b*^; Mikhael 2019*^	Downey 2017; Mikhael 2019*^	Kamyar 2016b*^; Rattray 2014*
Necrotising enterocolitis/spontaneous intestinal perforation–associated death	Hong 2019; **Shalabi 2017**		
Sepsis	Alston 2016; Bozkurt 2016; De Jesus 2015; Elimian 2002*; Girsen 2015; James 2015; Jazayeri 2003; Jung 2018; **Kamyar 2016a**; Lloreda-Garcia 2016; Mikhael 2019*; Morag 2016; Özlü 2019; Rantonen 2001; Riaz 1998; **Stockley 2018;** Teng 2006; **Weisz 2015**		Whitsel 2004
Antibiotic use	Elimian 2002*^; Greenberg 2013		Elimian 2002^
Hypoglycaemia	Bozkurt 2016; Grimbly 2015		
Feeding intolerance	Gursoy 2015; Özlü 2019; Riaz 1998		Belden 2017*
Delayed stooling	Lloreda-Garcia 2016^	Lloreda-Garcia 2016^	Brazy 1982; Das 2015*
Meconium passage delay	Ambadkar 2019; Lloreda-Garcia 2016		
Ileus			Brazy 1982; Nakamura 1991*
Delayed voiding	Sahin 2001		Das 2015*
Patent ductus arteriosus	**Basu 2012**; Bozkurt 2016; delValle 1998; Elimian 2002*; Garcia Alonso 2018; Gursoy 2015; Imamoglu 2014; James 2015; Katayama 2011*^; Lee 2013*; Özlü 2019; Schanler 1997; Yokoyama 2010	Qasim 2017	Brazy 1982; **del Moral 2007**; Gonzalez-Quintero 2001; **Katayama 2011**^; Lee 2013; Narasimhulu 2017; Shokry 2010
Patent ductus arteriosus (treated)	**De Jesus 2015**; del Moral 2007; delValle 1998; Katayama 2011*; Lee 2013; Lloreda-Garcia 2016; Mikhael 2019*; Shalabi 2017; Suh 2015		Bonta 2000*
Hypotension	Brazy 1982; Derks 2016; Drassinower 2015; Gursoy 2015; Morag 2016; **Teng 2006**	**De Jesus 2015**	Narasimhulu 2017
Hypertension	Gursoy 2015	Brown 2019	
Inotrope use	Imamoglu 2014; James 2015; Shokry 2010		
Intravenous fluids and/or nutritional support needed			Greenberg 2013; Rasch 1982
Phototherapy	Greenberg 2013; Havranek 2011; Imamoglu 2014		
Retinopathy of prematurity	**Basu 2012**; Bozkurt 2016; Cuff 2018*; De Jesus 2015; Elliott 2003; **Garcia Alonso 2018**; Jung 2018; Kamyar 2015a; **Kimberlin 1998**; Lee 2013; McPherson 2014*; Narasimhulu 2017; Özlü 2019; **Shalabi 2017**^; **Stockley 2018;** Suh 2015; **Weisz 2015**; Yokoyama 2010	**Shalabi 2017**^	Rauf 2017
Hypotonia	Bertello Grecco 2019*; Drassinower 2015; Gibbins 2013; Girsen 2015; Nassar 2006*		Ambadkar 2019; Brazy 1982; Das 2015*; Rauf 2017; Riaz 1998
Seizure	Drassinower 2015; Girsen 2015; **Kimberlin 1998**; McPherson 2014*; Rauf 2017	Shokry 2010	
Encephalopathy	Girsen 2015; Rantonen 2001; Rauf 2017		
Intraventricular haemorrhage	Alston 2016; Black 2006; De Jesus 2015; delValle 1998; de Veciana 1995; Drassinower 2015; **Edwards 2018**; Elliott 2003; Gano 2016; Garcia Alonso 2018; Gasparyan 2017; Gonzalez-Quintero 2001; Gursoy 2015; Hom 2018; Imamoglu 2014; Jazayeri 2003; Jung 2018^; Kamyar 2015a; Lee 2013; **Leviton 1997**; Martin 1998; McPherson 2014*; Mitani 2011*; Nassar 2006*; Nelson 1995; Özlü 2019; **Paneth 1997**; Schanler 1997; Stetson 2019; **Stockley 2018;** Suh 2015; Yokoyama 2010	Jung 2018^; **Kuban 1992**; Perlman 1995; **Petrova 2012**; Rantonen 2001; Rauf 2017; Shokry 2010	**Salafia 1995**
Intraventricular haemorrhage grade 3 or 4	del Moral 2007; **Downey 2017**; Gano 2016; James 2015; Jung 2018; **Kamyar 2016a**; **Kimberlin 1998**; McPherson 2014*; Mikhael 2019*; Narasimhulu 2017; Özlü 2019; Rantonen 2001; **Stockley 2018; Weintraub 2001**	Gasparyan 2017; Perlman 1995; **Sarkar 2009**; Wiswell 1996	Cuff 2018*; Khodapanahandeh 2008
Periventricular leucomalacia	Bozkurt 2016; De Jesus 2015; del Moral 2007; delValle 1998; Garcia Alonso 2018; Jung 2018^; Kamyar 2015a; **Kamyar 2016a**; Lee 2013; Mitani 2011*; Narasimhulu 2017; Rauf 2017; Suh 2015; Wiswell 1996	**FineSmith 1997**; Jung 2018^; **Murata 2005**	
Intraventricular haemorrhage or periventricular leucomalacia	Basu 2012; Canterino 1999*; Elimian 2002*		
Intraventricular haemorrhage grade 3 or 4 and/or periventricular leucomalacia	Bozkurt 2016; Canterino 1999*; Morag 2016; **Shalabi 2017**^; **Weisz 2015**	Koksal 2002; **Shalabi 2017**^; Wiswell 1996	
Hypocalcaemia	Cho 2014; Lee 2015; McGuiness 1980		Narasimhulu 2017
Bone abnormalities	Yokoyama 2010		Holcomb 1991*; Matsuda 1997*
Hearing impairment or hearing test failure	Jung 2018	**Leung 2016**	
Composite adverse outcomes	Drassinower 2015; Duffy 2012; Kamyar 2015a; Kamyar 2015b; Kamyar 2015c; **Kamyar 2016a**; Mitani 2011*; Narasimhulu 2017; **Palatnik 2019**; Rizzolo 2019; Sakae 2017*^; **Weisz 2015**		Boyle 2018; Sakae 2017*^
NICU admission	Ambadkar 2019*^; Bertello Grecco 2019*; Cawyer 2019; Chun 2014^; Gibbins 2013; Lai 2017; McPherson 2014*; Rantonen 2001; Riaz 1998*		Ambadkar 2019*^; Chun 2014^; Das 2015*; **Girsen 2015**; Greenberg 2011*; Greenberg 2013*; Rhee 2012
NICU duration	Gibbins 2013; **Girsen 2015**; Greenberg 2013; Jazayeri 2003; Jung 2018; Kimberlin 1998; Rauf 2017		Narasimhulu 2017
Hospital stay duration	Alston 2016; **Basu 2012**; De Jesus 2015; de Veciana 1995; Özlü 2019; Riaz 1998; Schanler 1997; Suh 2015		Brazy 1982; Girsen 2015
Other (outcomes reported by single studies)	Black 2006; Blackwell 2002; Brazy 1982; Derks 2016; Gano 2016; Girsen 2015; Greenberg 2013; Havranek 2011; Hong 2019; Imamoglu 2014; Jeanneteau 2014; Jones 2018; Jung 2018; Katayama 2011*; Kelly 1992; **Kimberlin 1998**; Lai 2017; **Leviton 1997**; Lloreda-Garcia 2016; Mittendorf 2005*; Morag 2016; Nassar 2006*; Nunes 2018; Özlü 2019; **Paneth 1997**; Petrov 2013; Rantonen 2001; Riaz 1998; Sahin 2001; Schanler 1997; **Shalabi 2017**^	**Deering 2005**; Derks 2016; **Gano 2016**; Jeanneteau 2014; Kimberlin 1998; Mittendorf 2005*; Petrov 2013	Belden 2017*; Brazy 1982; Das 2015*; **Katayama 2011**; Lai 2017; Lipsitz 1971*; Mittendorf 2009*; **Morag 2015**; Morag 2016; Rasch 1982; **Shalabi 2017**^; Verma 2006*; Weisz 2015; Whitten 2015

The bold studies were judged to be of higher quality (moderate or moderate to high risk of bias) and presented results adjusted for confounders for the relevant outcomes; other studies were judged to be at high or unclear risk of bias and/or or did not present adjusted results for the relevant outcomes.

*Indicates where studies assessed different magnesium sulphate regimens or 1 or more characteristics of the regimen (such as dose, duration, timing, or indication for use).

^Indicates where studies demonstrated mixed findings (such as in different subgroups of the population).

NICU, neonatal intensive care unit.

Seventeen of the 138 non-randomised studies (14 at moderate or moderate to high risk of bias, and 3 at unclear risk of bias [abstracts only]) observed a possible increase in the risk of adverse neonatal outcomes with antenatal magnesium sulphate, with some consideration of important confounding variables in relevant outcome analyses [68,73,84,90,106,108,109,129,132,134,140,152,153,173,180,184,191]. Potential increased risks with antenatal magnesium sulphate of 4 outcomes (discussed below) were observed by more than 1 study. For the remaining outcomes (nosocomial infection [184], enteral feeding intolerance [68], respiratory disease composite [153], pulmonary interstitial emphysema [191], early germinal matrix/intraventricular haemorrhage [180], thalamostriate or mineralising vasculopathy [152], and a composite neonatal adverse outcome [73]), single studies reported possible harms.

Potential increased risk of neonatal death before intensive care unit discharge, and of a composite outcome of neonatal death before intensive care unit discharge and/or necrotising enterocolitis, was shown among a subgroup of babies born less than 26 weeks gestation with the use of antenatal magnesium sulphate for fetal neuroprotection (293 babies, in a retrospective cohort of 697 babies born less than 28 weeks gestation) [129]. A possible increased risk of the composite outcome spontaneous intestinal perforation or neonatal death was also shown among a subgroup of babies born less than 25 weeks gestation with higher cumulative antenatal magnesium sulphate doses for fetal neuroprotection (non-concurrent cohort of 155 babies born less than 1,000 grams) [173].

A possible increased risk of patent ductus arteriosus was observed with magnesium sulphate given for pre-eclampsia (retrospective cohort of 81 ‘very low birthweight’ babies; abstract only) [140] or for pre-eclampsia or tocolysis (retrospective cohort of 941 babies born 500 to 1,000 grams) [90]. Further, a possible increased risk of patent ductus arteriosus in babies born 26 weeks gestation or later was shown with cumulative antenatal magnesium sulphate doses for pre-eclampsia or tocolysis of at least 50 grams (retrospective cohort of 941 babies born 500 to 1,000 grams) [90]. Potential increased risk of symptomatic patent ductus arteriosus, and of failure of early closure of the ductus arteriosus, was also observed with the use of antenatal magnesium sulphate for tocolysis (retrospective cohort of 160 babies born less than 28 weeks gestation, who all received indomethacin prophylaxis) [132].

A potential increased risk of intraventricular haemorrhage grade 3 or 4 was observed with antenatal magnesium sulphate for tocolysis (case–control study of 121 babies born less than 1,500 grams) [134], and with a higher dose regimen of antenatal magnesium sulphate for fetal neuroprotection (6-gram IV loading dose and 2-gram-per-hour IV maintenance dose for 12 hours versus 4-gram IV loading dose only) (retrospective cohort, including 54 babies exposed to magnesium sulphate within 12 hours of birth) [84].

A possible increased risk of neonatal intensive care unit admission with the use of antenatal magnesium sulphate for pre-eclampsia was observed (retrospective cohorts of 264 babies and 2,166 babies born at 37 weeks gestation or later) [106,109]. Further increased risks of neonatal intensive care unit and special care unit admission were observed with higher total hours, higher total doses, more than 12 hours, and more than 30 grams of antenatal magnesium sulphate for pre-eclampsia (retrospective cohort of 242 babies born at 35 weeks gestation or later) [108].

### Evidence from case reports

Nineteen reports describing a total of 134 babies exposed to antenatal magnesium sulphate with adverse outcomes were included [200–218] (see Table 9; the detailed characteristics of cases are presented in S4 Table).

**Table 9:** Summary of main adverse outcomes from case reports.

Outcome	Indication for use: studies
Neonatal death	Tocolysis: Herschel 2001
	Pre-eclampsia/eclampsia: Kurtoglu 2000
Cardiopulmonary arrest after gentamicin exposure following hypermagnesemia at birth	Pre-eclampsia: L’Hommedieu 1983; Rasch 1981
Clinical features of magnesium ‘toxicity’ or ‘intoxication’ at birth (such as apnoea, cyanosis, hypotonia, and/or hyporeflexia)	Pre-eclampsia/eclampsia: Brady 1967; Cruz 2009; Lipsitz 1967; Teng 1989
	Not clear: Jashi 2014
Microcolon or ‘meconium-plug syndrome’	Pre-eclampsia/eclampsia: Amodio 1986; Krasna 1996; Sokal 1972
Nonoliguric hyperkalaemia	Pre-eclampsia: Tanaka 2018
Bone abnormalities with prolonged magnesium sulphate for tocolysis	Tocolysis: Cumming 1989; Kaplan 2006; Kogan 2003; Lamm 1988; Malaeb 2004
	Not clear: Ahmad 2013

Clinical features of neonatal hypermagnesemia, magnesium ‘toxicity’, or magnesium ‘intoxication’ at birth were the focus of 5 reports (35 neonates), in which antenatal magnesium sulphate was given for pre-eclampsia/eclampsia (with exposure durations and doses ranging from 3.5 to 46 hours and 11 to 60.4 grams prior to birth, respectively) [202,205,206,213,218], and were variably described throughout the remaining reports. These included low Apgar scores, apnoea, cyanosis, hypotonia, and/or hyporeflexia, with or without the need for active resuscitation and calcium gluconate administration.

Two reports described neonatal death following antenatal magnesium sulphate exposure. In the first report, death was considered to be related to ‘the toxic effects of magnesium on the myocardium’ when given for tocolysis (4-gram IV loading dose followed by 2.5-gram-per-hour IV maintenance dose for approximately 1 day: 51.4 grams total) [204], and in the second, 1 death (of 7) was attributed to ‘overdose’ (unclear dose/regimen) of magnesium sulphate when given for pre-eclampsia/eclampsia [210].

In the context of hypermagnesemia at birth (following exposure to a total of 24 to 28 grams of magnesium sulphate for pre-eclampsia), neonatal gentamicin administration for suspected sepsis was associated with respiratory arrest and cardiac arrest in 2 reports [211,215]. Other specific adverse outcomes attributed to antenatal magnesium sulphate exposure included

- Microcolon or meconium-plug syndrome (3 reports, 14 neonates, when given for pre-eclampsia/eclampsia: 25 to 41 grams in the day prior to birth in 1 report; regimen not described in 2 reports) [201,209,216];
- Nonoliguric hyperkalaemia (1 report, 1 neonate, when given for pre-eclampsia: 0.1 gram to 2 grams per hour IV for 12 days) [217];
- Bone abnormalities (commonly metaphyseal osteopenia, in some cases leading to fracture) (6 reports, 35 neonates, when given for tocolysis: ranging from 1 to 4 grams per hour for between 8 and 13 weeks) [200,203,207,208,212,214].

## Discussion

Overall, no clear difference in our primary review outcome, perinatal death, was shown in the randomised trials comparing antenatal magnesium sulphate with placebo/no treatment, nor in regimen comparisons in randomised trials. While 11 of the 138 non-randomised studies reported on perinatal death, only 1 cohort study (at moderate to high risk of bias) observed a possible increased risk of perinatal death, with high-dose (more than 48 grams) antenatal magnesium sulphate exposure for tocolysis [183].

Results for secondary adverse neonatal outcomes were reassuring, with very few clear differences observed between antenatal magnesium sulphate and placebo/no treatment or between different magnesium sulphate regimens. Where possible harms of magnesium sulphate were seen, commonly no confounders were taken into account (and studies were judged to be at high risk of bias), study samples were small (less than 200 babies), and/or differences were observed in (non-formal) subgroup analyses only. Non-randomised studies identified a limited number of outcomes justifying further evaluation, such as from large, high-quality studies (prospective cohorts, individual participant data meta-analyses, or randomised trials of regimen comparisons). These included neonatal death and intestinal morbidity in very preterm neonates with exposure for fetal neuroprotection, patent ductus arteriosus in very preterm or very low birthweight neonates with exposure for pre-eclampsia or tocolysis, and intensive care unit admission in term neonates with exposure for pre-eclampsia. Case reports suggested an association between neonatal bone abnormalities and long-term, high-dose exposure to antenatal magnesium sulphate for tocolysis.

We are not aware of any other published systematic reviews with a focus on potential adverse outcomes for neonates following antenatal magnesium sulphate exposure, though we identified 3 review registrations focused specifically on magnesium sulphate for tocolysis and the outcomes neonatal respiratory depression (CRD42017058912), patent ductus arteriosus (CRD42017060049), and bone abnormalities (CRD42017062550) [18]. Three previous systematic reviews have assessed ‘adverse events’ [13], ‘side effects’ [219], and ‘safety’ [220] for women, and a further systematic review has assessed the effects of antenatal magnesium sulphate specifically on fetal heart rate parameters, finding a small negative effect on rate, variability, and accelerative pattern, ‘not sufficient clinically to warrant medical intervention’ [14].

Our review findings are consistent with those from the relevant Cochrane reviews comparing antenatal magnesium sulphate with placebo/no treatment, or different magnesium sulphate regimens [1,2,4,9,221], though our review includes a wider range of outcomes. As was observed in our review’s tocolysis subgroup for perinatal death, the Cochrane review assessing magnesium sulphate for tocolysis demonstrated a borderline increased risk of fetal, neonatal, or infant death with antenatal magnesium sulphate [9].

A recent non-systematic narrative review evaluated ‘whether antenatal MgSO_4_ is beneficial or harmful’ in extremely and very preterm neonates [222]. Relevant systematic reviews, meta-analyses, randomised controlled trials, and observational studies were retrieved, with a broad search strategy focused on neuroprotection and cerebral palsy, necrotising enterocolitis, and spontaneous intestinal perforation. The narrative review suggested that current evidence supports the neuroprotective role of antenatal magnesium sulphate for preterm neonates, and that, while the effects are ‘controversial’ and ‘not well established’, a ‘high index of suspicion of gastrointestinal complications in extremely preterms, particularly < 26 weeks of gestation’ is recommended [222]. While our review similarly identified a possibility of harm, the relevant studies were of questionable methodological quality, and our systematic review included additional reports (of higher quality, and involving much larger cohorts) that indicated no increased risk of intestinal morbidity.

The findings of this review are reassuring and can be considered in conjunction with those from relevant reviews demonstrating a clear benefit of antenatal magnesium sulphate [1,2,4], and current international clinical practice guideline recommendations [3,7]. Our review findings of possible adverse outcomes with long-term, high-dose use for tocolysis have implications for settings with continued use for this indication [8] in spite of the absence of benefit shown in systematic reviews and international guidance [7–9].

### Strengths and limitations

The main limitations of our review relate to missing data for important outcomes across most studies, the inclusion of published data only, and the heterogeneity of included studies.

Of the 40 randomised trials included in this review, our primary outcome (perinatal death) was reported by 22 (55%); it was reported by only 11 (8%) of the 138 included non-randomised studies. Aside from related mortality outcomes (stillbirth and neonatal death), all other adverse outcomes were reported sparsely, by less than a third of trials, with many outcomes reported by single trials only. While a broader range of adverse neonatal outcomes were reported by the non-randomised studies, apart from neonatal death (50 studies), intraventricular haemorrhage (39 studies), and necrotising enterocolitis (36 studies), all other outcomes were reported by less than a fifth of studies, again, with many reported by single studies only.

In addition to missing data for important outcomes across most studies, a further limitation includes the number of studies with relatively small sample sizes comparing different antenatal magnesium sulphate regimens. Moreover, many studies assessing antenatal magnesium sulphate for relevant indications were not included due to lack of reporting of adverse neonatal outcomes.

We searched extensively across multiple databases and reviewed reference lists for additional reports; however, we did not seek unpublished data. Recent evidence has suggested that while much adverse event information remains unpublished, inclusion of such data generally does not change the direction or statistical significance of pooled risk estimates [223]. While we were not able to fully evaluate non-English publications without available translations for inclusion, we have provided a list of these for readers to consider (S3 Text).

As the aim of the review was to provide a comprehensive, general view of potential unintended adverse outcomes, we designed this review with broad scope [15,16]. This presented challenges, including the number of diverse outcomes, inconsistent reporting, and the vast quantities of heterogeneous data. The study characteristics (including designs, settings, participating women and neonates, and antenatal magnesium sulphate regimens) varied greatly, and reporting was commonly incomplete. The ability to conduct subgroup analyses for the randomised trials was limited, and we did not inappropriately pool data from non-randomised studies. We conducted this review in accordance with recommendations for systematic reviews of adverse events [15–17], and of randomised and non-randomised studies more generally [224]. Our evaluation thus provides a firm basis for any further, narrowly focused studies of specific outcomes and characteristics.

### Conclusions

In conclusion, our findings do not support any clear associations between perinatal death or other adverse neonatal outcomes and antenatal magnesium sulphate exposure when given for the beneficial indications of maternal neuroprotection in pre-eclampsia/eclampsia and fetal neuroprotection in cerebral palsy prevention. To further inform safety recommendations of this widely used treatment in pregnancy, future research should be directed towards identified research gaps surrounding specific adverse neonatal outcomes and the impact of particular regimen, pregnancy, and/or birth characteristics.

## Supporting information

S1 Appendix Forest plots and funnel plots for comparisons 1–8. (DOCX)

S1 Fig Risk of bias for randomised controlled trials. Risk of bias summary showing judgements about each risk of bias item for the 40 included randomised trials. Green represents ‘low risk of bias’; yellow, ‘unclear risk of bias’; red, ‘high risk of bias’.
(TIF)

S1 PRISMA Checklist Preferred Reporting Items for Systematic Reviews and Meta-Analyses (PRISMA) checklist. (DOCX)

S1 Table Characteristics of included studies. (DOCX)

S2 Table Risk of bias of included studies. (DOCX)

S3 Table Adverse outcomes from non-randomised studies. (DOCX)

S4 Table Adverse outcomes from case reports. (DOCX)

S1 Text Protocol. (PDF)

S2 Text Search strategies. (DOCX)

S3 Text Articles excluded at full-text screening due to absence of English translation. (DOCX)

S4 Text References for included studies. (DOCX)

## Abbreviations

CI: confidence interval
IM: intramuscular
IV: intravenous
MD: mean difference
RR: risk ratio
