# Supplementary material for: Antenatal magnesium sulphate and adverse neonatal outcomes: A systematic review and meta-analysis
Source: PLoS Med. 2019 Dec 6;16(12):e1002988. doi: 10.1371/journal.pmed.1002988 (PMC6897495; doi:10.1371/journal.pmed.1002988)
Supplement: S1 Appendix — (DOCX) [file pmed.1002988.s001.docx]

**Forest plots from randomised controlled trials**


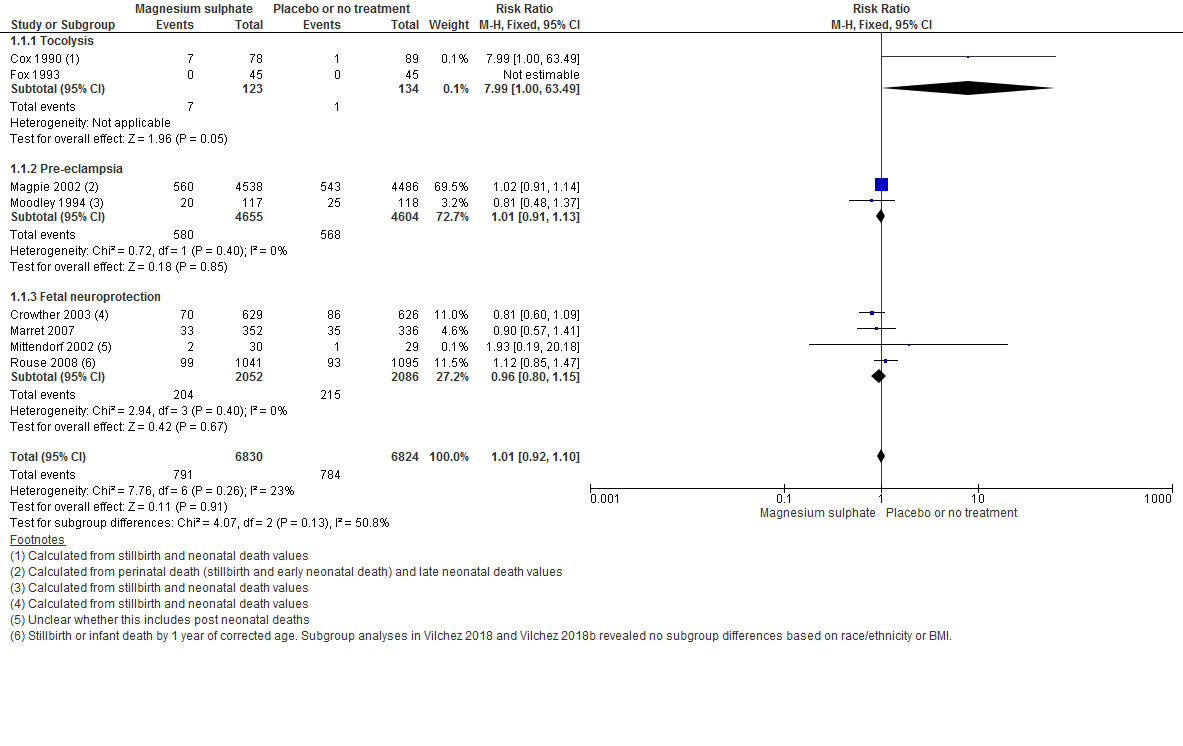


**Figure 1. Forest plot of Comparison: 1 Magnesium sulphate versus placebo or no treatment, outcome: 1.1 Perinatal death**


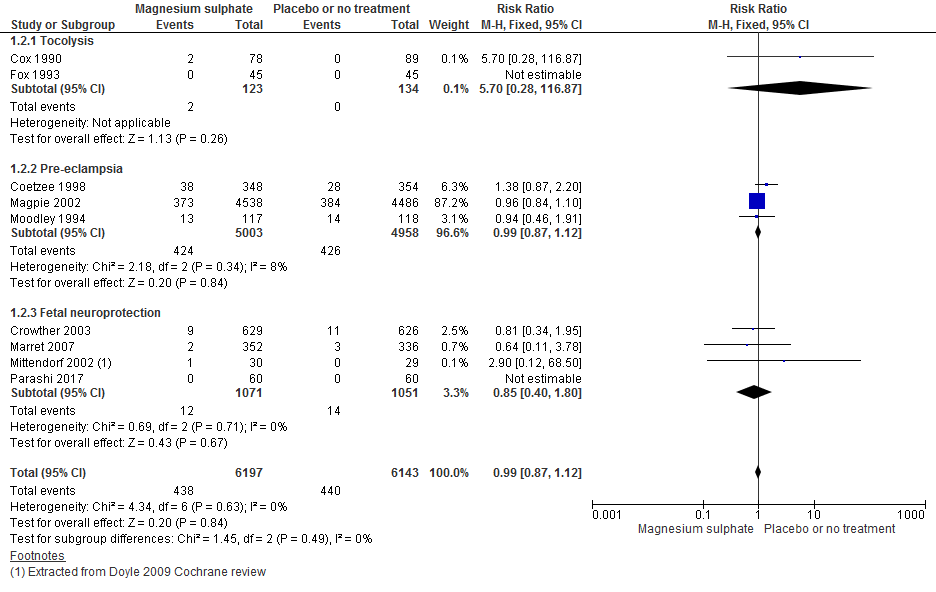


**Figure 2. Forest plot of Comparison: 1 Magnesium sulphate versus placebo or no treatment, outcome: 1.2 Stillbirth**


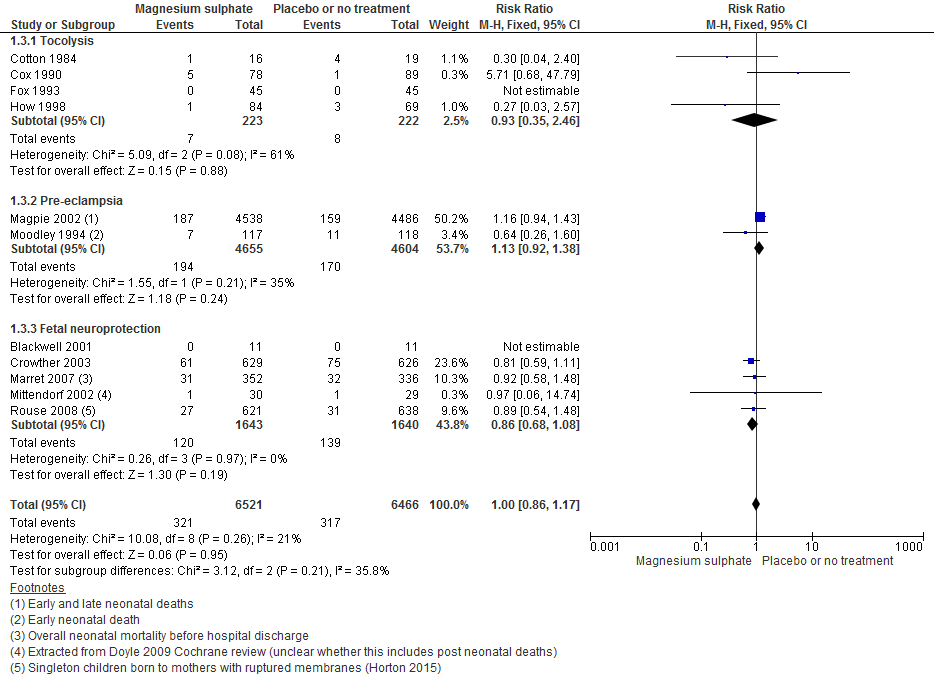


**Figure 3.1. Forest plot of Comparison: 1 Magnesium sulphate versus placebo or no treatment, outcome: 1.3 Neonatal death**

**
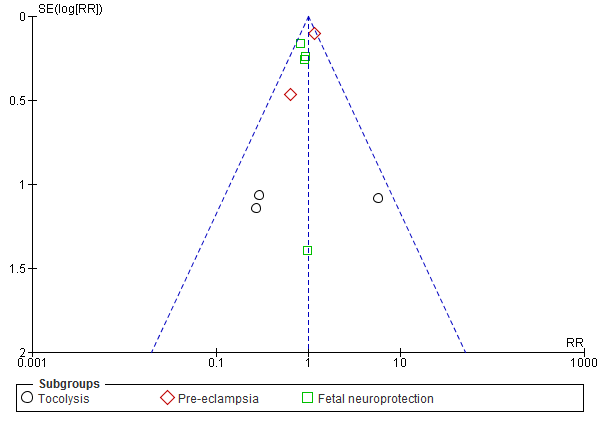
**

**Figure 3.2. Funnel plot of Comparison: 1 Magnesium sulphate versus placebo or no treatment, outcome: 1.3 Neonatal death**

**
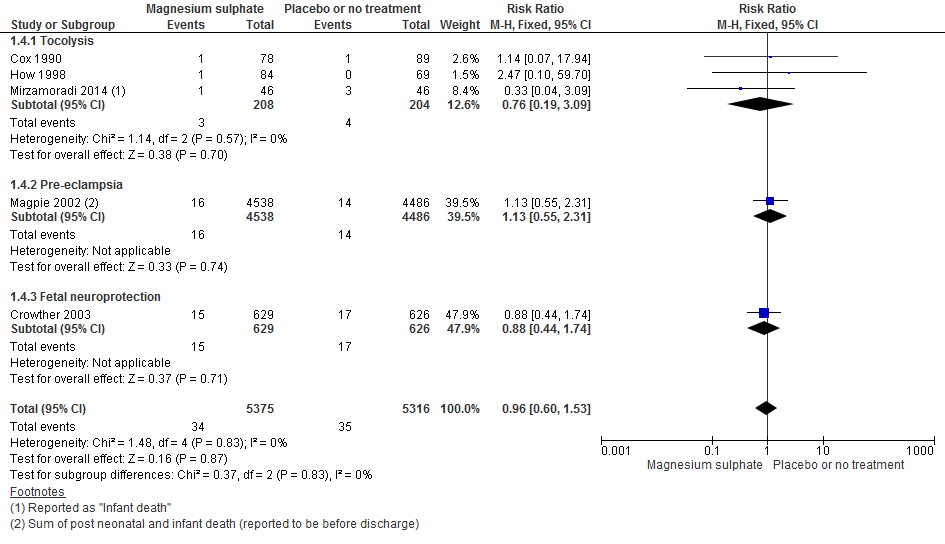
**

**Figure 4. Forest plot of Comparison: 1 Magnesium sulphate versus placebo or no treatment, outcome: 1.4 Death > 28 days, before discharge**

**
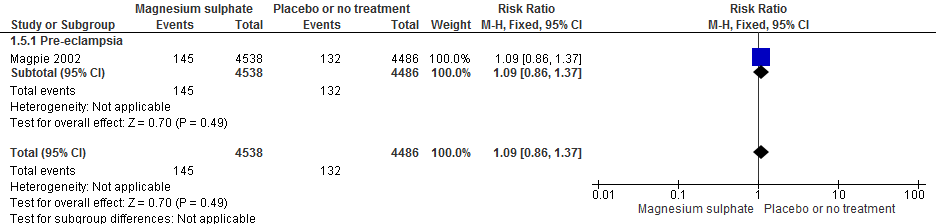
**

**Figure 5. Forest plot of Comparison: 1 Magnesium sulphate versus placebo or no treatment, outcome: 1.5 Early neonatal death**

**
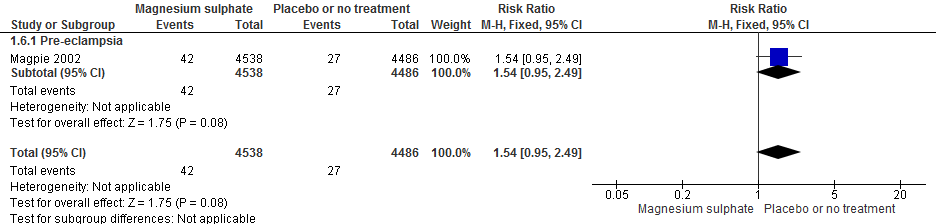
**

**Figure 6. Forest plot of Comparison: 1 Magnesium sulphate versus placebo or no treatment, outcome: 1.6 Late neonatal death**

**
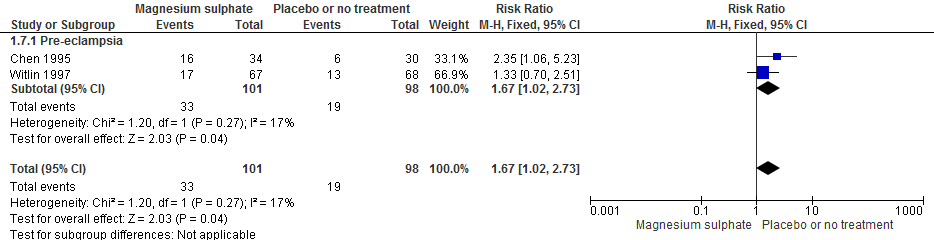
**

**Figure 7. Forest plot of Comparison: 1 Magnesium sulphate versus placebo or no treatment, outcome: 1.7 Apgar score < 7 at 1 minute**

**
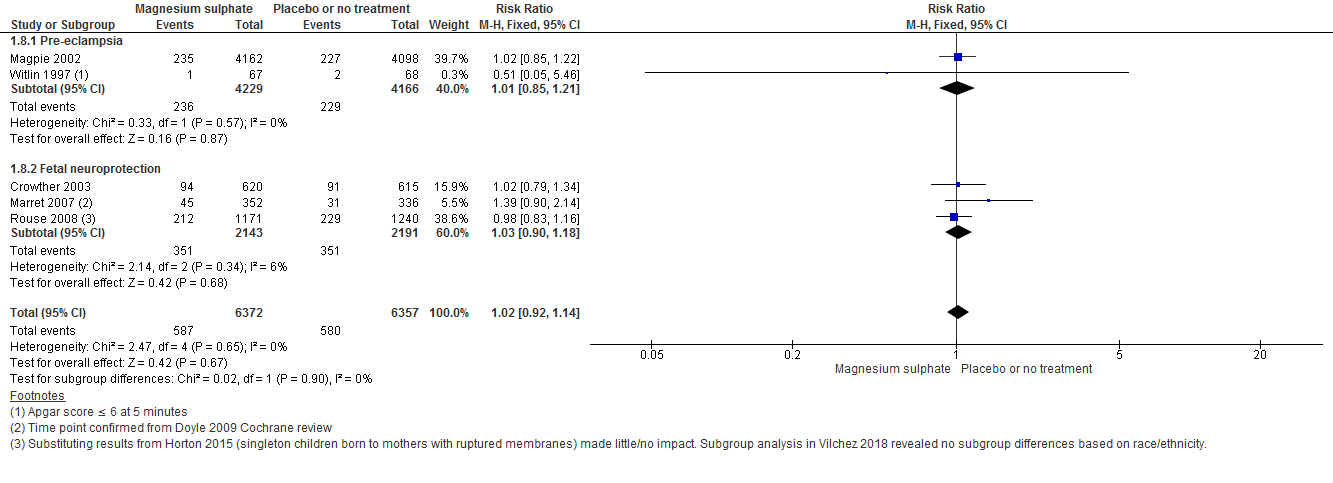
**

**Figure 8. Forest plot of Comparison: 1 Magnesium sulphate versus placebo or no treatment, outcome: 1.8 Apgar score < 7 at 5 minutes**

**
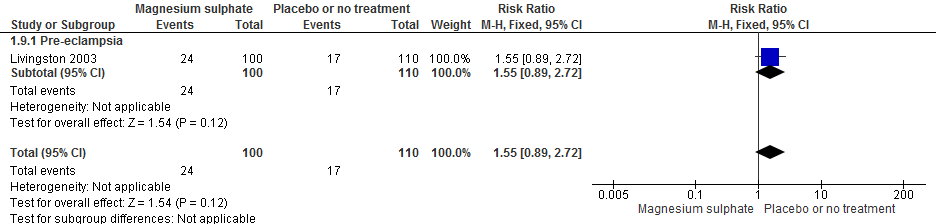
**

**Figure 9. Forest plot of Comparison: 1 Magnesium sulphate versus placebo or no treatment, outcome: 1.9 Meconium at delivery**

**
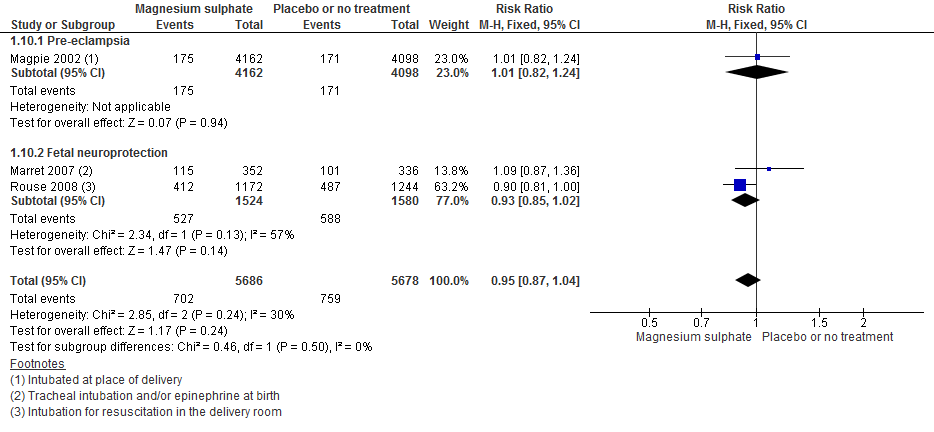
**

**Figure 10. Forest plot of Comparison: 1 Magnesium sulphate versus placebo or no treatment, outcome: 1.10 Intubated at birth**

**
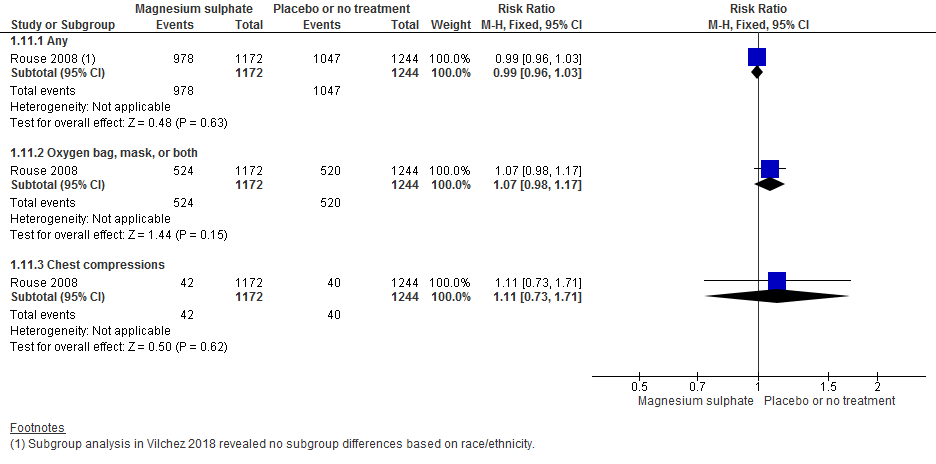
**

**Figure 11. Forest plot of Comparison: 1 Magnesium sulphate versus placebo or no treatment, outcome: 1.11 Resuscitation in the delivery room**

**
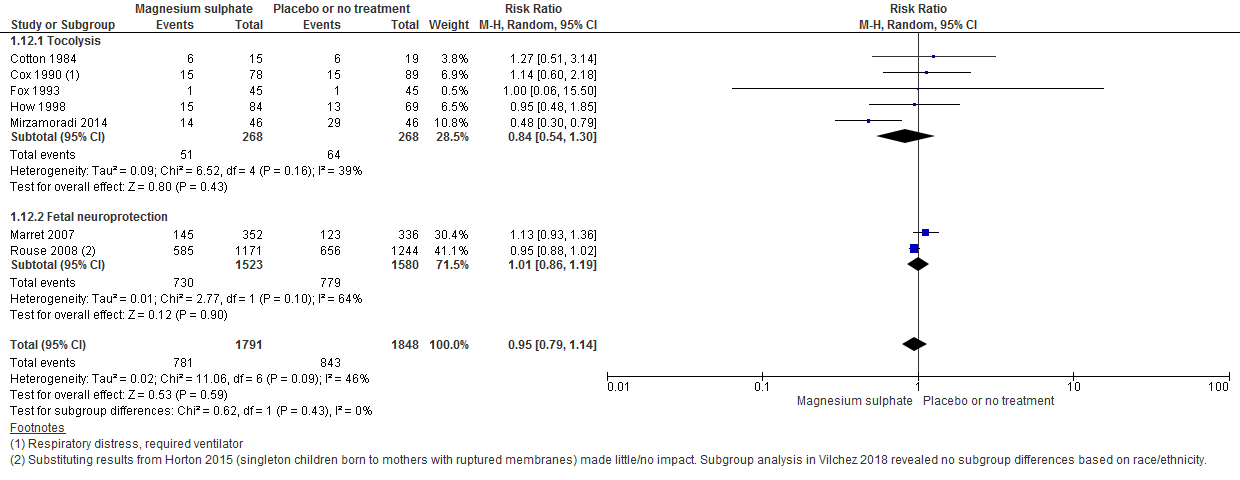
**

**Figure 12. Forest plot of Comparison: 1 Magnesium sulphate versus placebo or no treatment, outcome: 1.12 Respiratory distress syndrome**

**
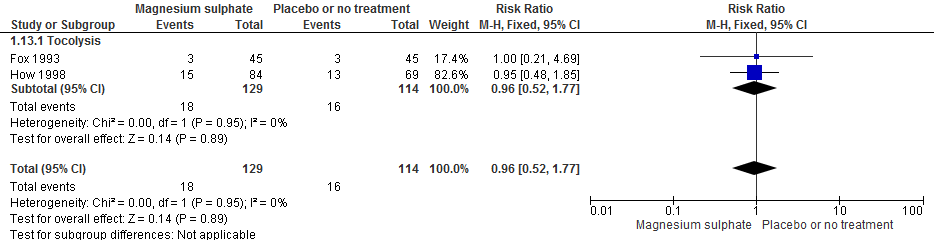
**

**Figure 13. Forest plot of Comparison: 1 Magnesium sulphate versus placebo or no treatment, outcome: 1.13 Transient tachypnoea of the newborn**

**
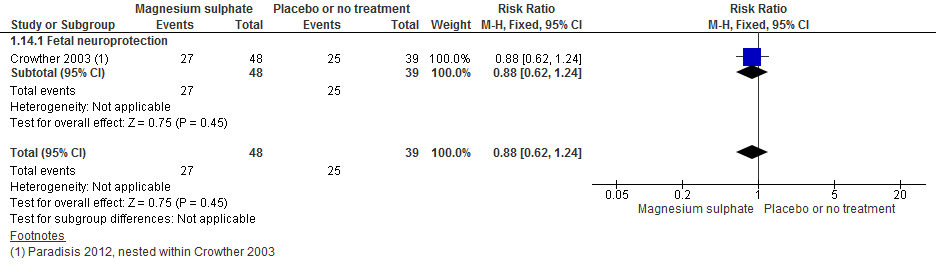
**

**Figure 14. Forest plot of Comparison: 1 Magnesium sulphate versus placebo or no treatment, outcome: 1.14 Surfactant**

**
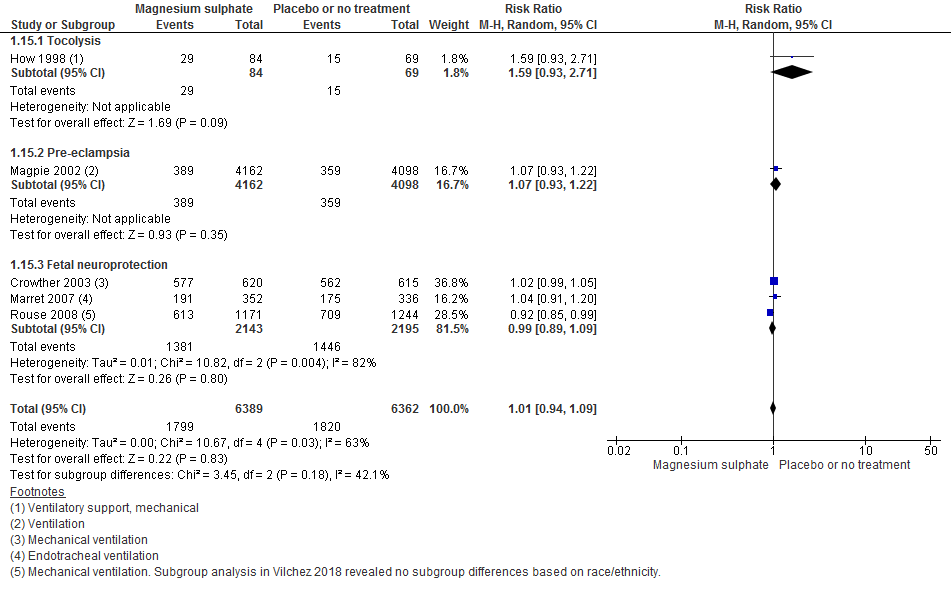
**

**Figure 15. Forest plot of Comparison: 1 Magnesium sulphate versus placebo or no treatment, outcome: 1.15 Mechanical ventilation**

**Additional data reported by How 1998: ventilatory support (magnesium sulphate group (84 babies) median: 2.5 days (interquartile range: 11; range: 0.04 to 81) versus no treatment group (69 babies) median: 5 days (interquartile range: 7; range 0.5 to 383; “P = not significant”). A further trial reported that “The average number of days with the use of the ventilator… were similar in the two groups” (Fox 1993), however did not provide data suitable for inclusion in a meta-analysis.*

**
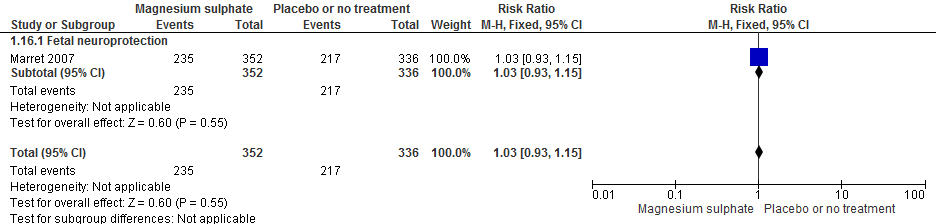
**

**Figure 16. Forest plot of Comparison: 1 Magnesium sulphate versus placebo or no treatment, outcome: 1.16 Non-invasive ventilation**

**
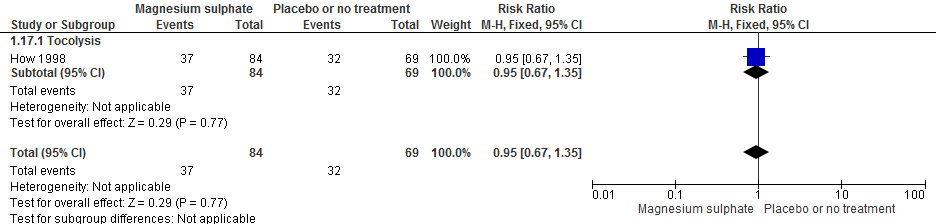
**

**Figure 17. Forest plot of Comparison: 1 Magnesium sulphate versus placebo or no treatment, outcome: 1.17 Oxygen required***

**Additional data reported by How 1998: oxygen required (magnesium sulphate group (84 babies) median: 4 days (interquartile range: 27; range: 0.04 to 95) versus no treatment group (69 babies) median: 5.5 days (interquartile range: 15.8; range 0.2 to 383; “P = not significant”)*

**
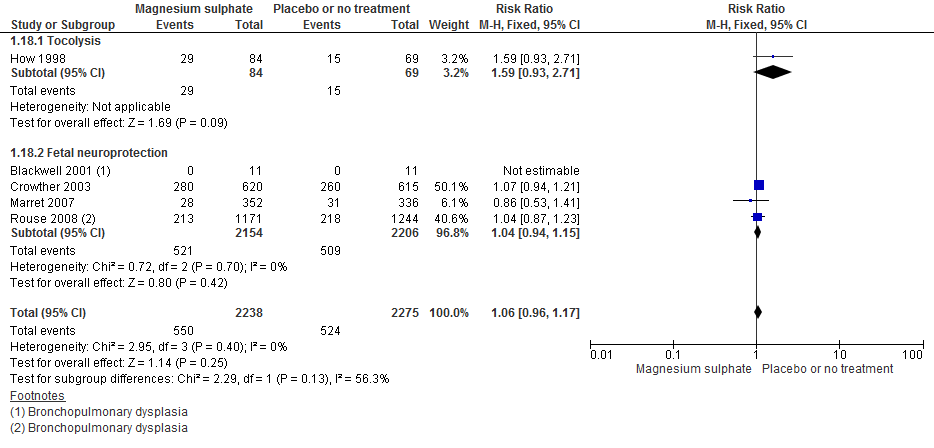
**

**Figure 18. Forest plot of Comparison: 1 Magnesium sulphate versus placebo or no treatment, outcome: 1.18 Chronic lung disease**


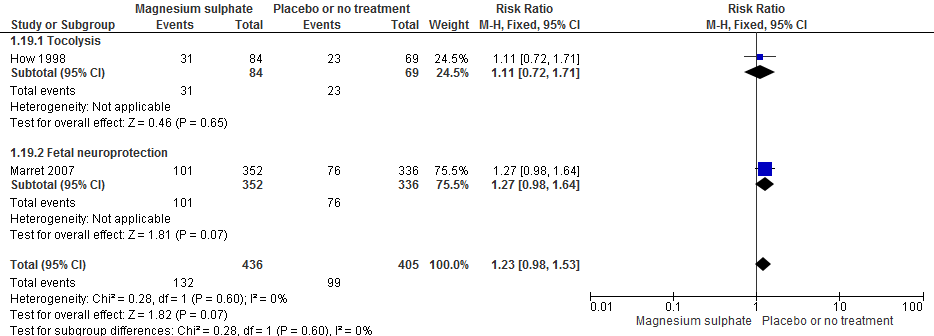


**Figure 19. Forest plot of Comparison: 1 Magnesium sulphate versus placebo or no treatment, outcome: 1.19 Apnoea and bradycardia**

**
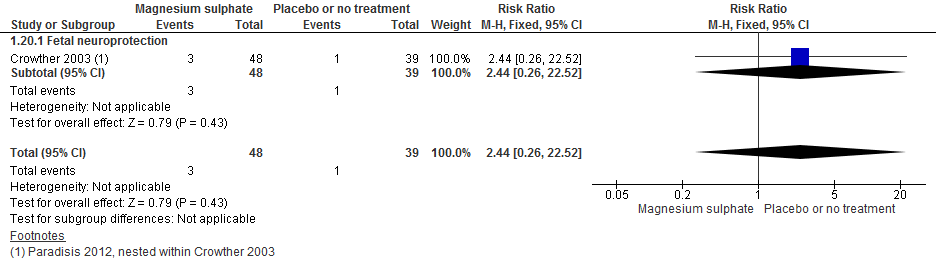
**

**Figure 20. Forest plot of Comparison: 1 Magnesium sulphate versus placebo or no treatment, outcome: 1.20 Pneumothorax**

**
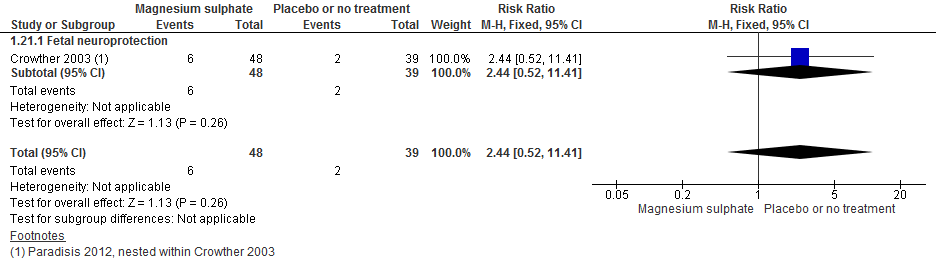
**

**Figure 21. Forest plot of Comparison: 1 Magnesium sulphate versus placebo or no treatment, outcome: 1.21 Pulmonary haemorrhage**

**
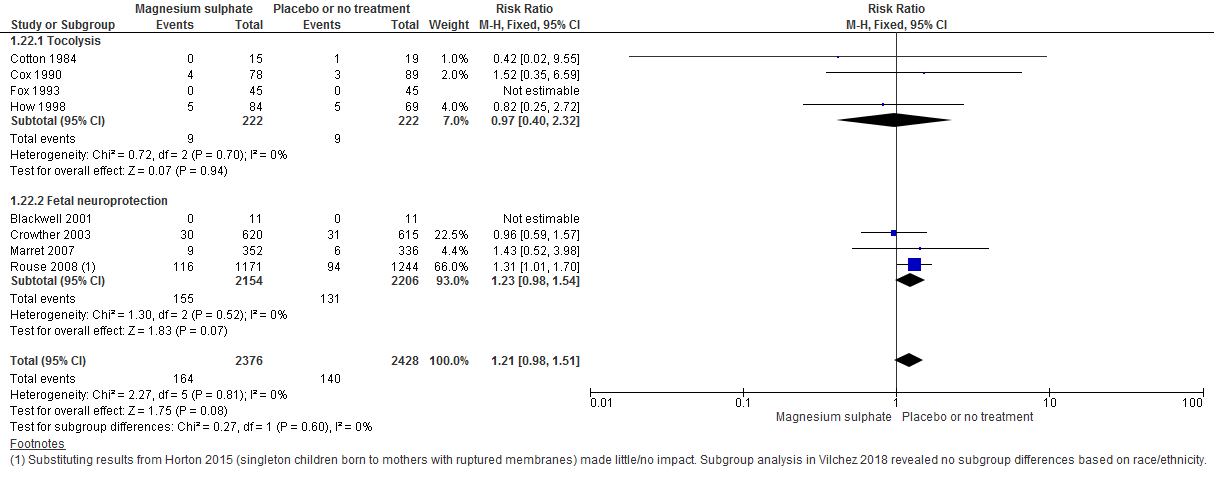
**

**Figure 22. Forest plot of Comparison: 1 Magnesium sulphate versus placebo or no treatment, outcome: 1.22 Necrotising enterocolitis**

**
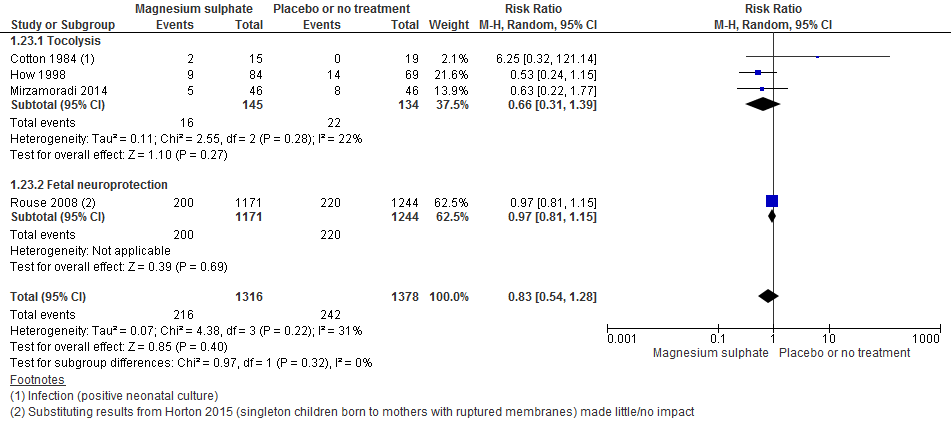
**

**Figure 23. Forest plot of Comparison: 1 Magnesium sulphate versus placebo or no treatment, outcome: 1.23 Sepsis**

**
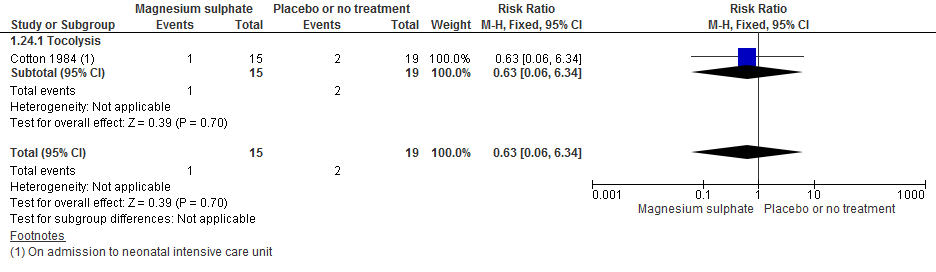
**

**Figure 24. Forest plot of Comparison: 1 Magnesium sulphate versus placebo or no treatment, outcome: 1.24 Hypoglycaemia on neonatal intensive care unit admission**

**
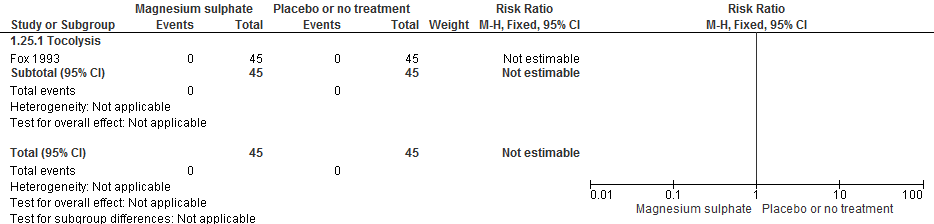
**

**Figure 25. Forest plot of Comparison: 1 Magnesium sulphate versus placebo or no treatment, outcome: 1.25 Poor feeding**

**
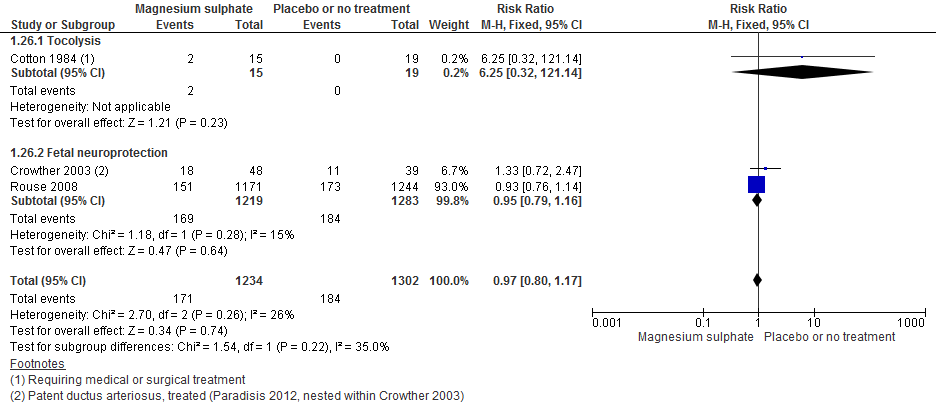
**

**Figure 26. Forest plot of Comparison: 1 Magnesium sulphate versus placebo or no treatment, outcome: 1.26 Patent ductus arteriosus**

**
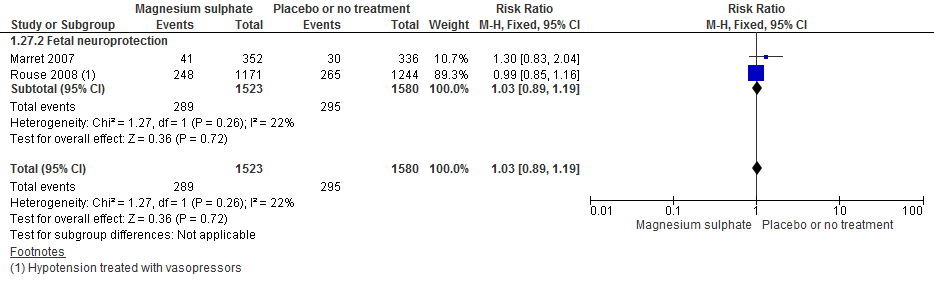
**

**Figure 27. Forest plot of Comparison: 1 Magnesium sulphate versus placebo or no treatment, outcome: 1.27 Hypotension**

**
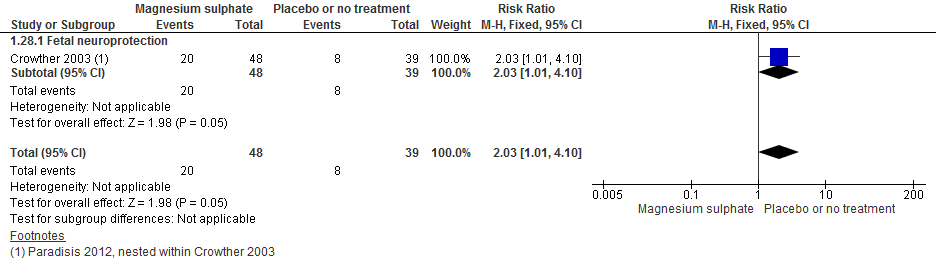
**

**Figure 28. Forest plot of Comparison: 1 Magnesium sulphate versus placebo or no treatment, outcome: 1.28 Volume expansion**

**
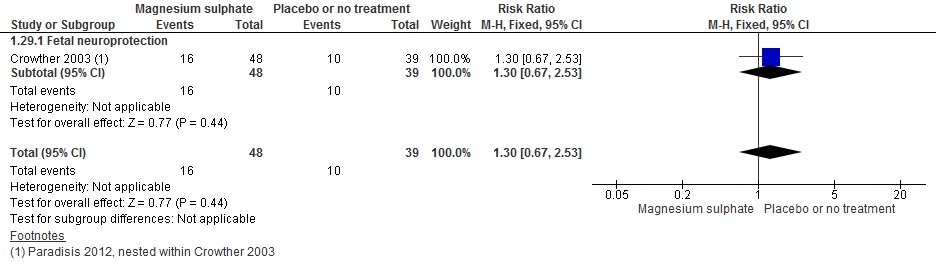
**

**Figure 29. Forest plot of Comparison: 1 Magnesium sulphate versus placebo or no treatment, outcome: 1.29 Mean blood pressure < 10^th^ centile in first 24 hours**

**
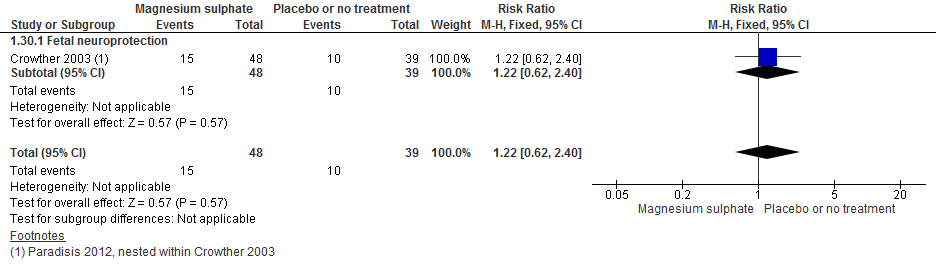
**

**Figure 30. Forest plot of Comparison: 1 Magnesium sulphate versus placebo or no treatment, outcome: 1.30 Superior vena cava flow (< 41 mL/kg/min) in first 24 hours**

**
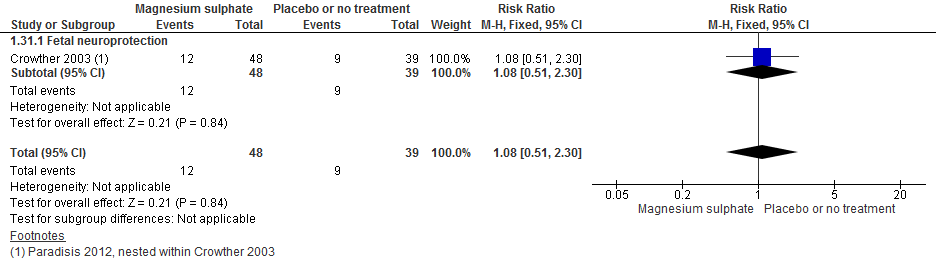
**

**Figure 31 Forest plot of Comparison: 1 Magnesium sulphate versus placebo or no treatment, outcome: 1.31 Right ventricular output (< 120 mL/kg/min) in first 24 hours**

**
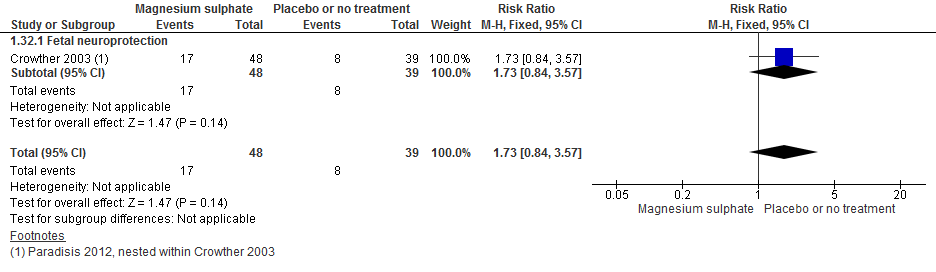
**

**Figure 32 Forest plot of Comparison: 1 Magnesium sulphate versus placebo or no treatment, outcome: 1.32 Dobutamine**

**
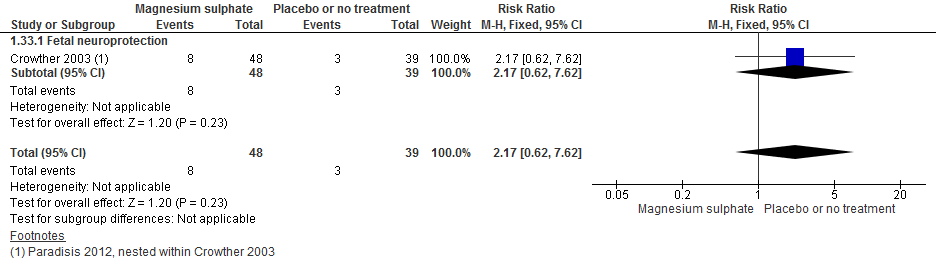
**

**Figure 33 Forest plot of Comparison: 1 Magnesium sulphate versus placebo or no treatment, outcome: 1.33 Dopamine**

**
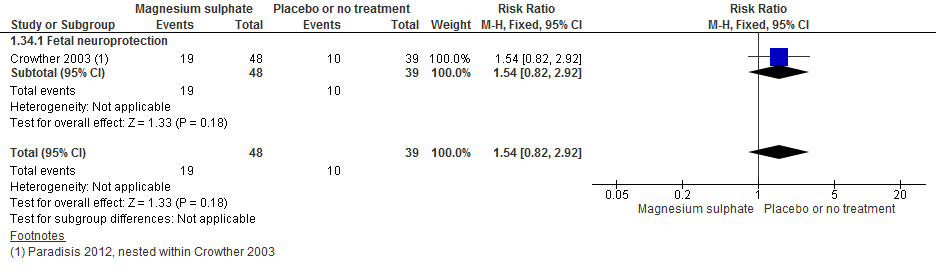
**

**Figure 34 Forest plot of Comparison: 1 Magnesium sulphate versus placebo or no treatment, outcome: 1.34 Any inotrope**

**
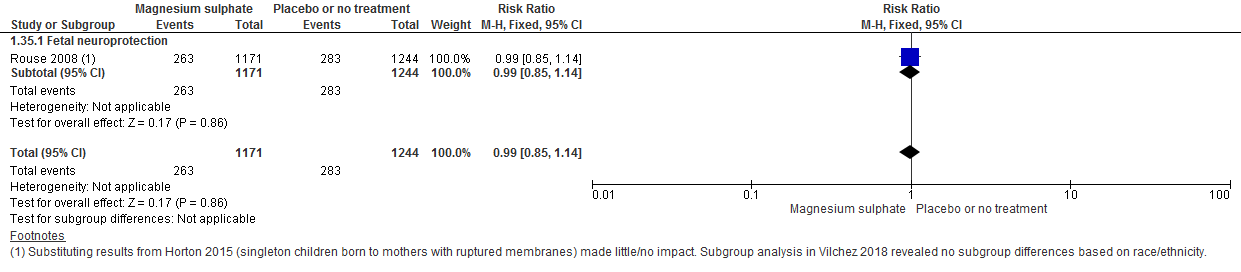
**

**Figure 35 Forest plot of Comparison: 1 Magnesium sulphate versus placebo or no treatment, outcome: 1.35 Retinopathy of prematurity**

**
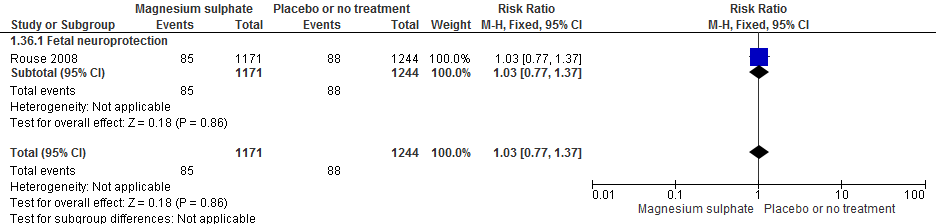
**

**Figure 36 Forest plot of Comparison: 1 Magnesium sulphate versus placebo or no treatment, outcome: 1.36 Generalised hypotonicity**

**
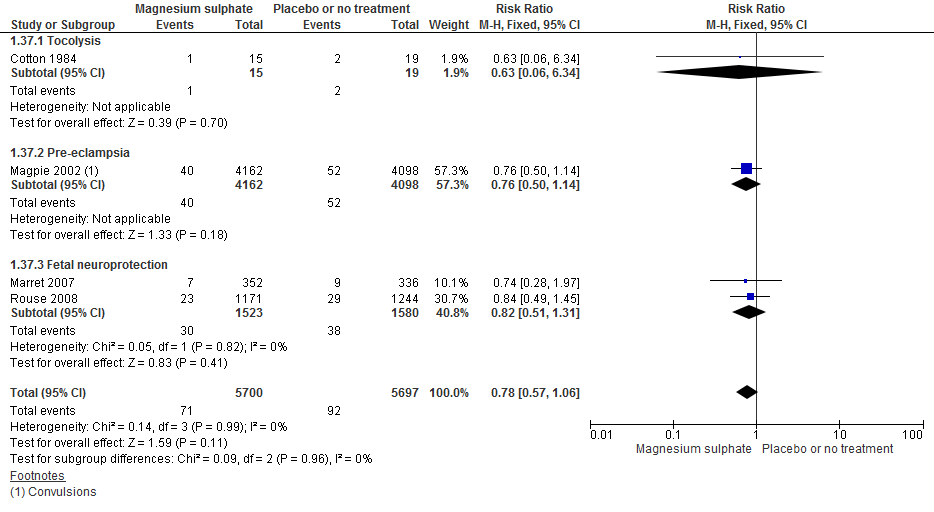
**

**Figure 37. Forest plot of Comparison: 1 Magnesium sulphate versus placebo or no treatment, outcome: 1.37 Seizures**

**
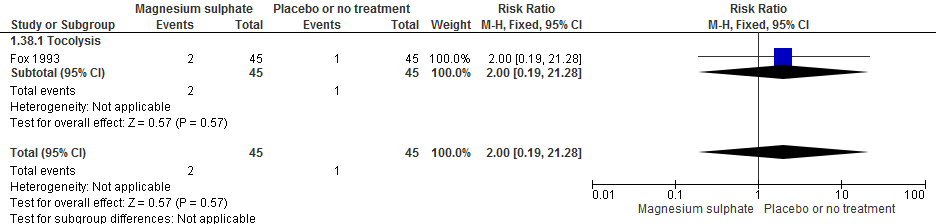
**

**Figure 38. Forest plot of Comparison: 1 Magnesium sulphate versus placebo or no treatment, outcome: 1.38 Hyperbilirubinaemia**

**
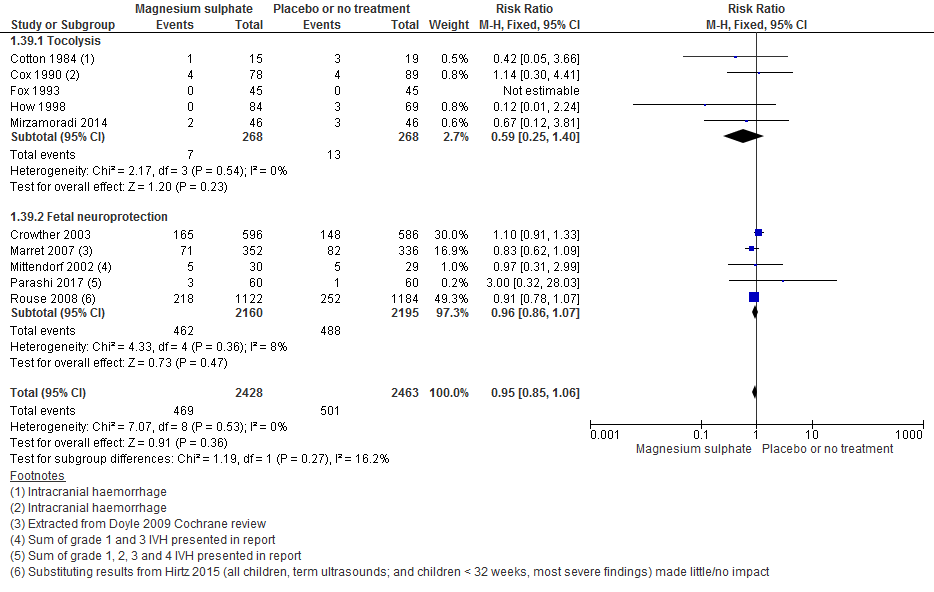
**

**Figure 39.1. Forest plot of Comparison: 1 Magnesium sulphate versus placebo or no treatment, outcome: 1.39 Intraventricular haemorrhage**

**
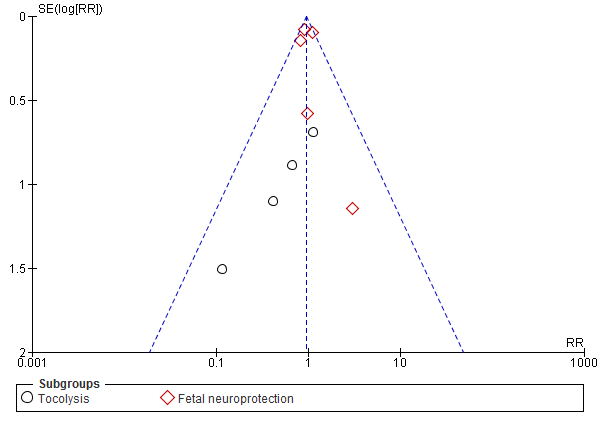
**

**Figure 39.2. Funnel plot of Comparison: 1 Magnesium sulphate versus placebo or no treatment, outcome: 1.39 Intraventricular haemorrhage**

**
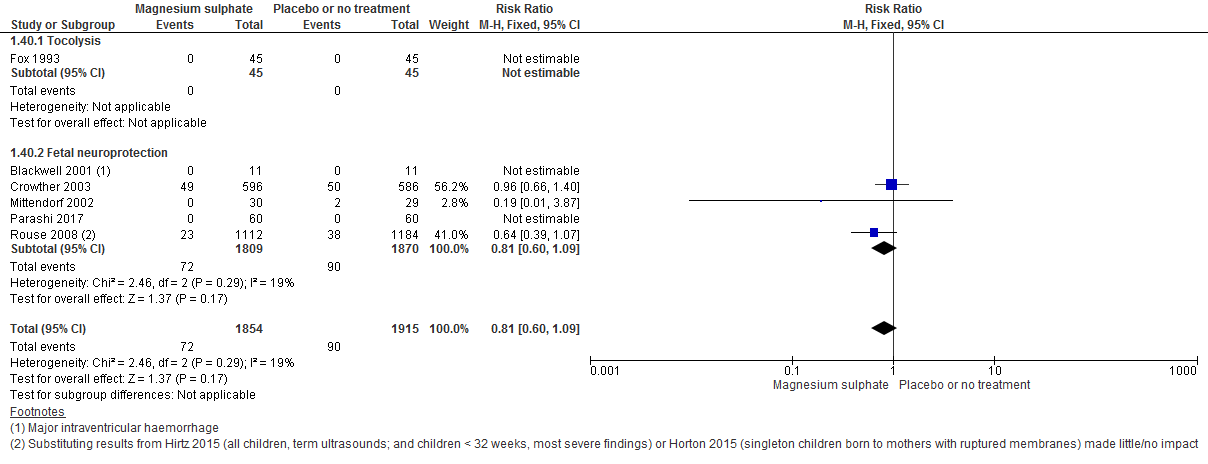
**

**Figure 40. Forest plot of Comparison: 1 Magnesium sulphate versus placebo or no treatment, outcome: 1.40 Intraventricular haemorrhage, grade III/IV**

**
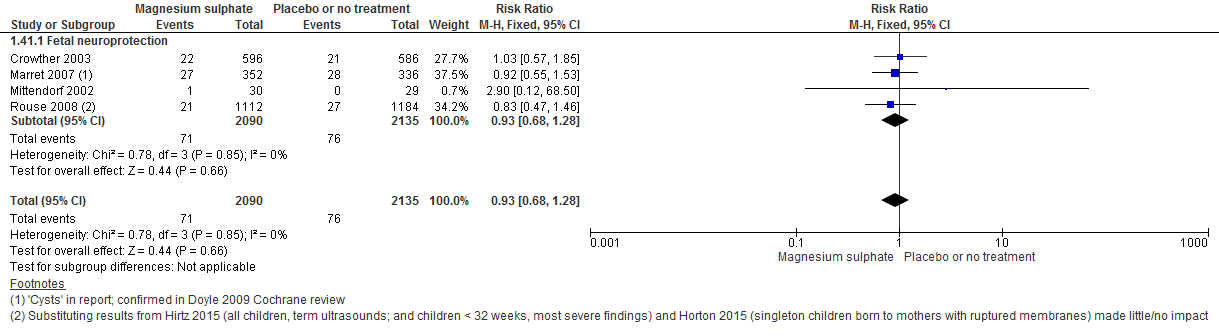
**

**Figure 41. Forest plot of Comparison: 1 Magnesium sulphate versus placebo or no treatment, outcome: 1.41 Periventricular leucomalacia**

**
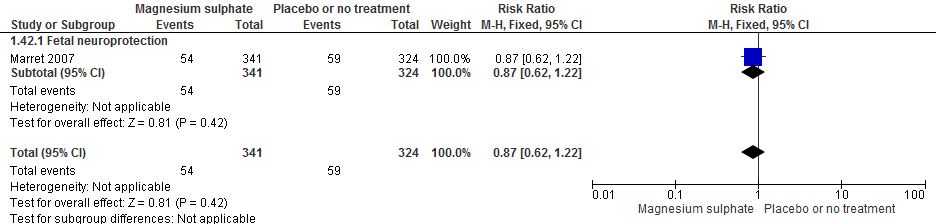
**

**Figure 42. Forest plot of Comparison: 1 Magnesium sulphate versus placebo or no treatment, outcome: 1.42 Any white matter injury**

**
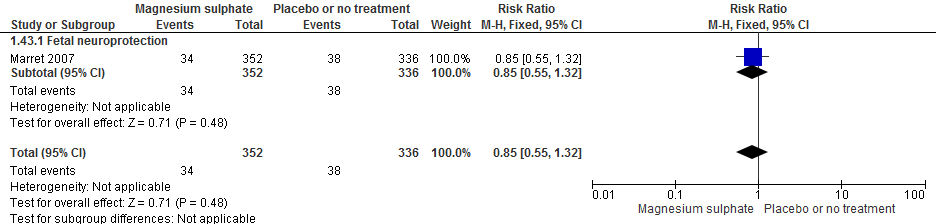
**

**Figure 43. Forest plot of Comparison: 1 Magnesium sulphate versus placebo or no treatment, outcome: 1.43 Severe white matter injury**

**
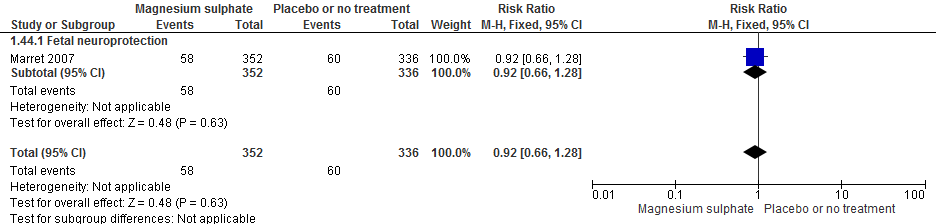
**

**Figure 44. Forest plot of Comparison: 1 Magnesium sulphate versus placebo or no treatment, outcome: 1.44 Severe white matter injury or death**

**
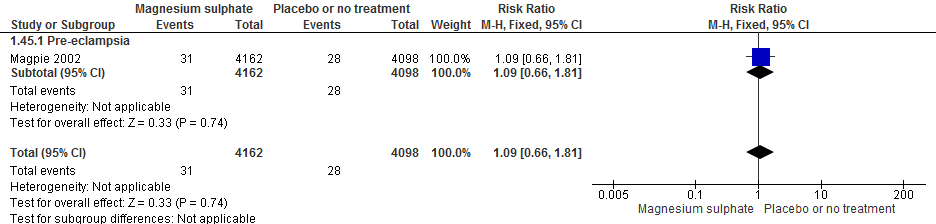
**

**Figure 45. Forest plot of Comparison: 1 Magnesium sulphate versus placebo or no treatment, outcome: 1.45 Persistent parenchymal echogenicity**

**
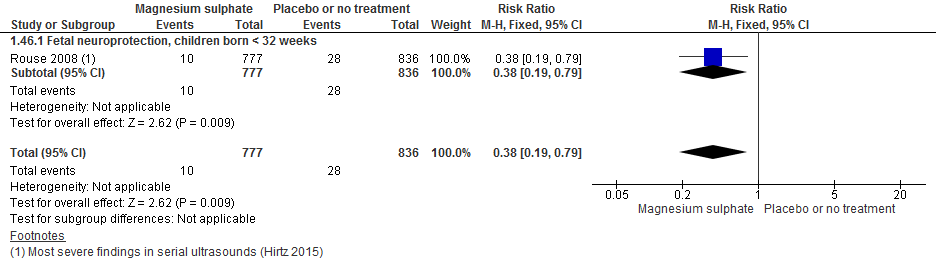
**

**Figure 46. Forest plot of Comparison: 1 Magnesium sulphate versus placebo or no treatment, outcome: 1.46 Echodensity**

**
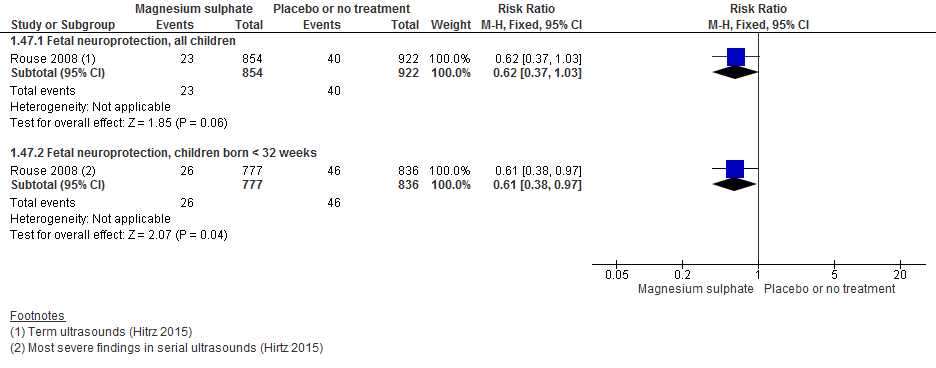
**

**Figure 47. Forest plot of Comparison: 1 Magnesium sulphate versus placebo or no treatment, outcome: 1.47 Echolucency**

**
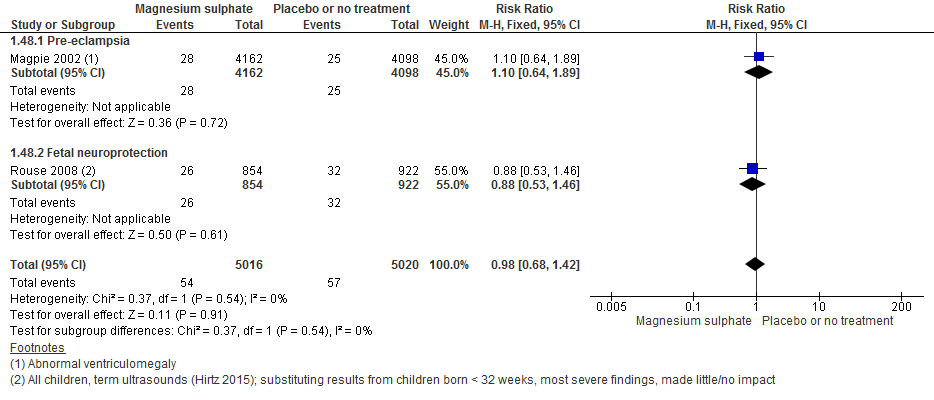
**

**Figure 48. Forest plot of Comparison: 1 Magnesium sulphate versus placebo or no treatment, outcome: 1.48 Ventriculomegaly**

**
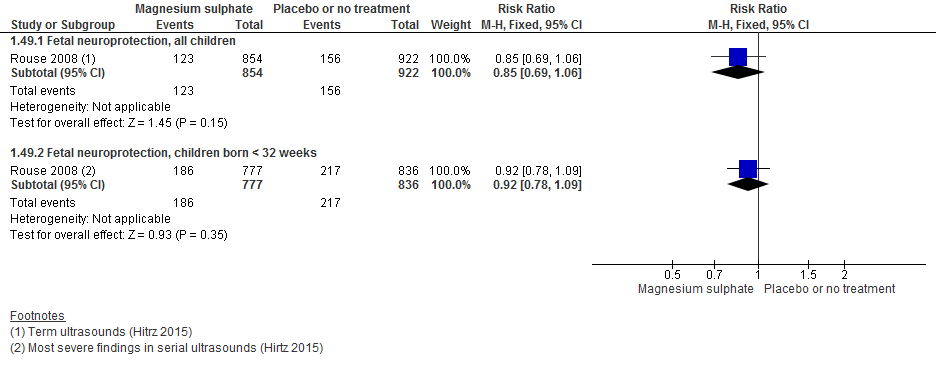
**

**Figure 49. Forest plot of Comparison: 1 Magnesium sulphate versus placebo or no treatment, outcome: 1.49 Any of echodensity, echolucency, intraventricular haemorrhage, periventricular haemorrhage, ventriculomegaly**

**
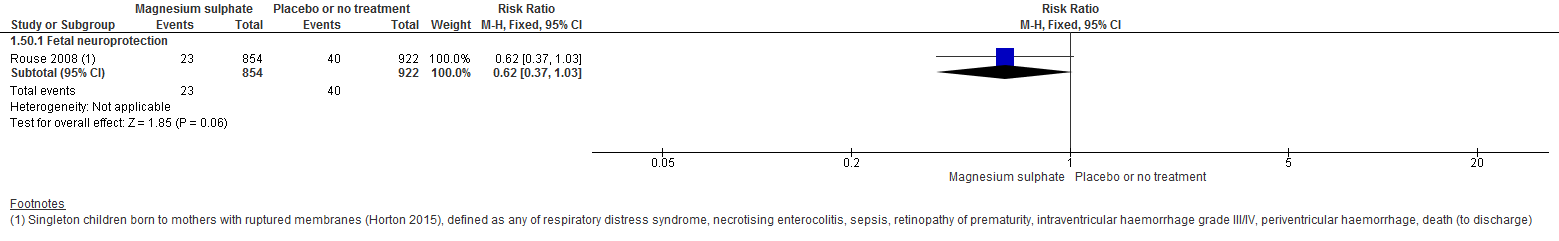
**

**Figure 50. Forest plot of Comparison: 1 Magnesium sulphate versus placebo or no treatment, outcome: 1.50 Composite adverse outcome**

**
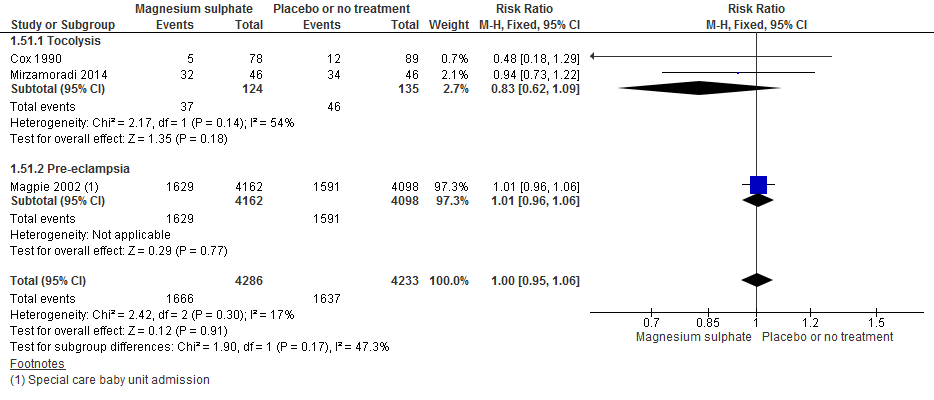
**

**Figure 51. Forest plot of Comparison: 1 Magnesium sulphate versus placebo or no treatment, outcome: 1.51 Neonatal intensive care unit admission***

********Two trials reported that “There were no differences between groups in… admissions to the NICU,” (Colon 2015) and “The average… newborn intensive care time for infants who did have complications were similar in the two groups” (Fox 1993), however did not provide data suitable for inclusion in a meta-analysis.*

**
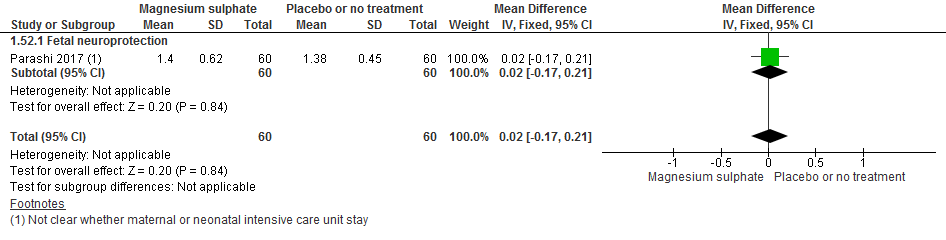
**

**Figure 52. Forest plot of Comparison: 1 Magnesium sulphate versus placebo or no treatment, outcome: 1.52 Intensive care unit stay (days)***

**Two trials reported the data in a format not suitable for meta-analysis, and similarly, did not see a clear difference between groups (magnesium sulphate group (620 babies): median: 76 days (range: 61 to 94) versus placebo group (615 babies) median: 74 days (range: 59 to 95); P = 0.66 (Crowther 2003); (magnesium sulphate group (84 babies) median: 29 days (interquartile range: 28; range: 2 to 204) versus no treatment group (69 babies) median: 28 days (interquartile range: 22; range 2 to 383; “P = not significant”) (How 1998).*

**
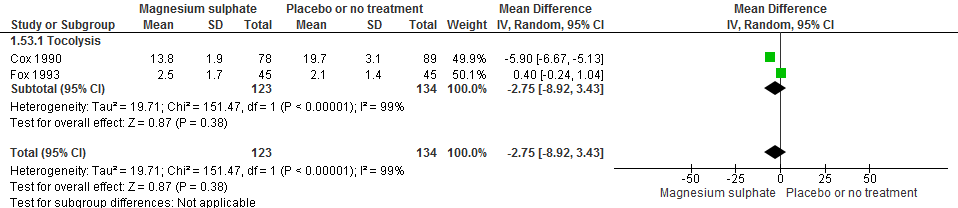
**

**Figure 53. Forest plot of Comparison: 1 Magnesium sulphate versus placebo or no treatment, outcome: 1.53 Hospital stay (days)**

**
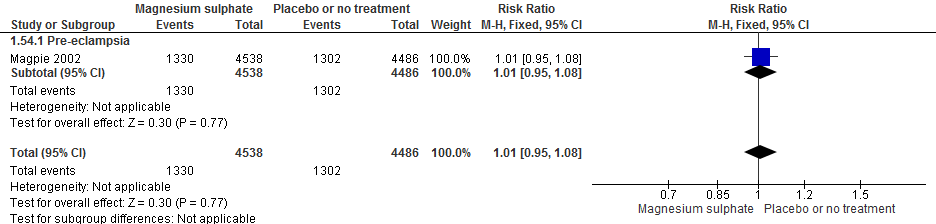
**

**Figure 54. Forest plot of Comparison: 1 Magnesium sulphate versus placebo or no treatment, outcome: 1.54 Special care baby unit admission > 7 days or death**

**
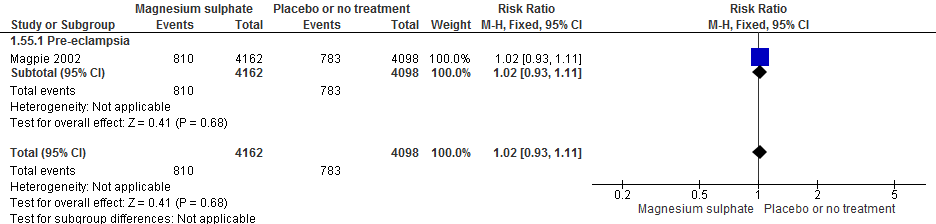
**

**Figure 55. Forest plot of Comparison: 1 Magnesium sulphate versus placebo or no treatment, outcome: 1.55 Special care baby unit admission > 7 days**

**
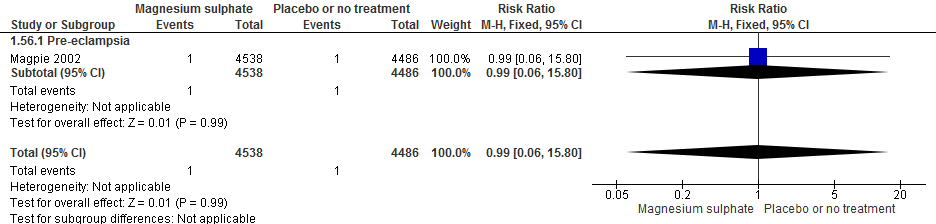
**

**Figure 56. Forest plot of Comparison: 1 Magnesium sulphate versus placebo or no treatment, outcome: 1.56 Still in hospital at 6 weeks**

**
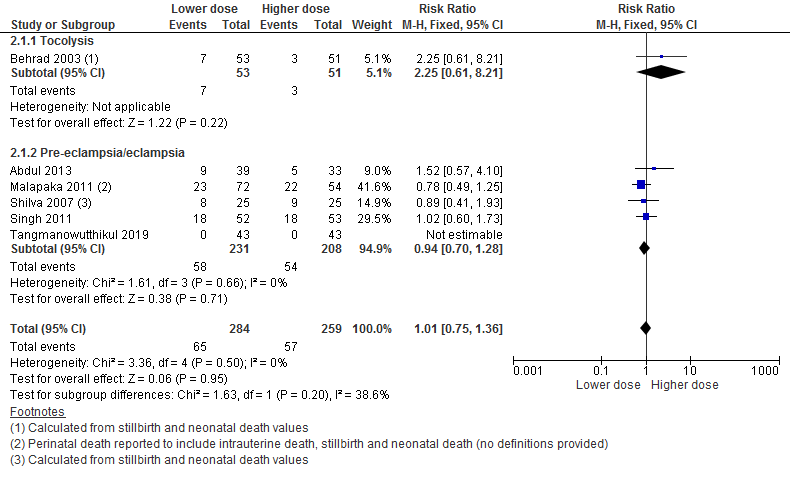
**

**Figure 57. Forest plot of Comparison: 2 Lower versus higher dose regimens of magnesium sulphate, outcome: 2.1 Perinatal death***

******* *One trial reported that “The neonatal outcome was similar in both the groups (p=0.911)” (Agrawal 2015); however did not provide data for inclusion in a meta-analysis.*

**
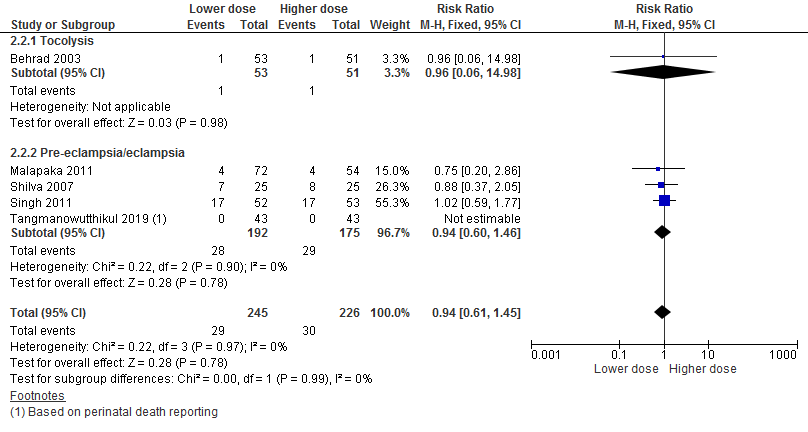
**

**Figure 58. Forest plot of Comparison: 2 Lower versus higher dose regimens of magnesium sulphate, outcome: 2.2 Stillbirth**

**
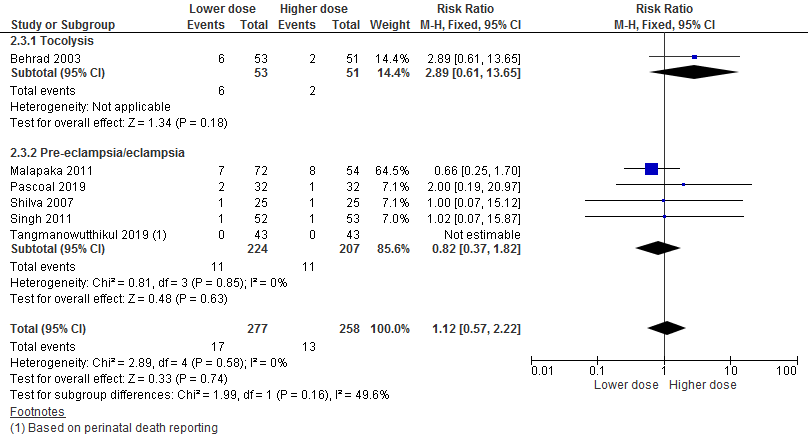
**

**Figure 59. Forest plot of Comparison: 2 Lower versus higher dose regimens of magnesium sulphate, outcome: 2.3 Neonatal death**

**
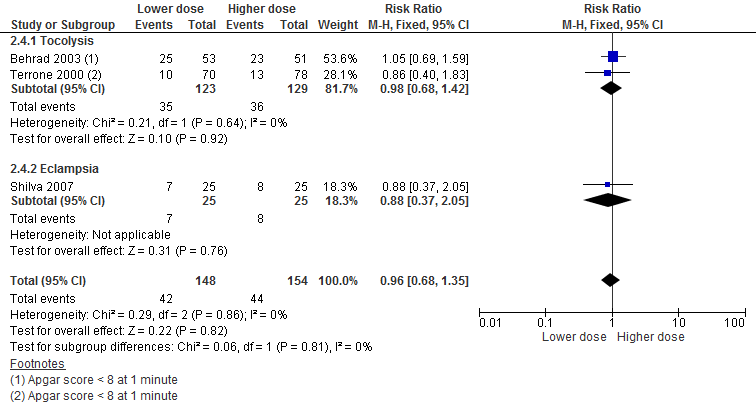
**

**Figure 60. Forest plot of Comparison: 2 Lower versus higher dose regimens of magnesium sulphate, outcome: 2.4 Apgar score < 7 at 1 minute**

**
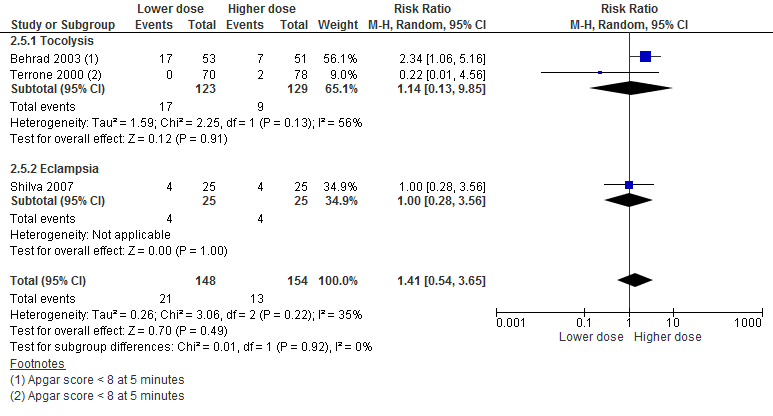
**

**Figure 61. Forest plot of Comparison: 2 Lower versus higher dose regimens of magnesium sulphate, outcome: 2.5 Apgar score < 7 at 5 minutes**

**
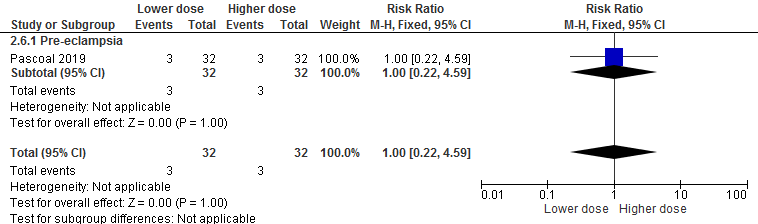
 Figure 62. Forest plot of Comparison: 2 Lower versus higher dose regimens of magnesium sulphate, outcome: 2.6 Resuscitation**

**
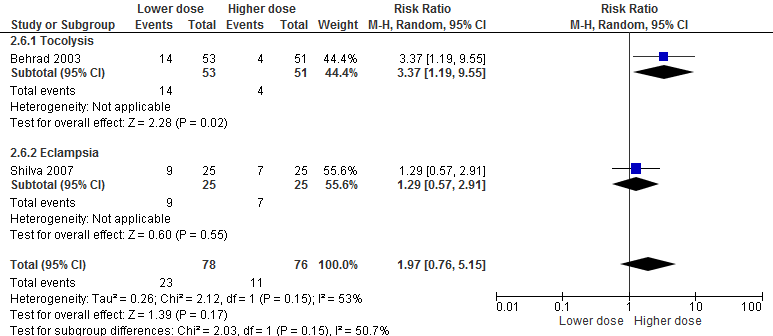
**

**Figure 63. Forest plot of Comparison: 2 Lower versus higher dose regimens of magnesium sulphate, outcome: 2.7 Respiratory distress syndrome**

**
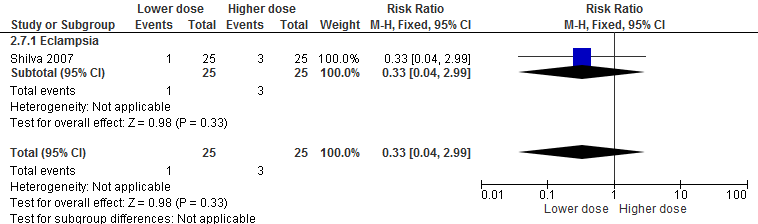
**

**Figure 64. Forest plot of Comparison: 2 Lower versus higher dose regimens of magnesium sulphate, outcome: 2.8 Respiratory depression**

**
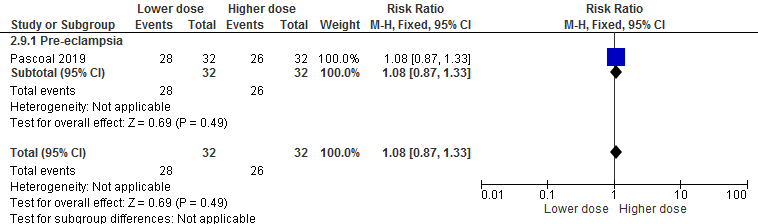
**

**Figure 65. Forest plot of Comparison: 2 Lower versus higher dose regimens of magnesium sulphate, outcome: 2.9 Respiratory disorders**

**
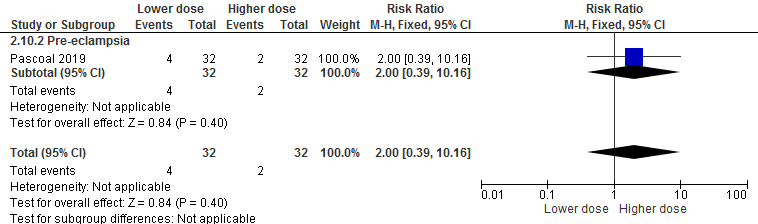
**

**Figure 66. Forest plot of Comparison: 2 Lower versus higher dose regimens of magnesium sulphate, outcome: 2.10 Mechanical ventilation**

**
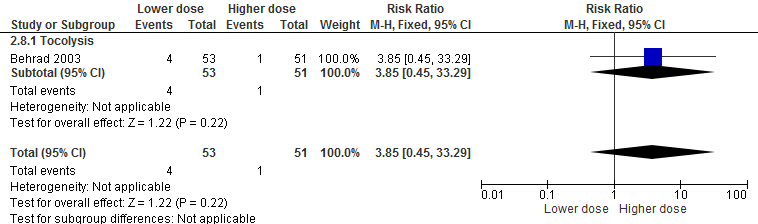
**

**Figure 67. Forest plot of Comparison: 2 Lower versus higher dose regimens of magnesium sulphate, outcome: 2.11 Bradycardia**

**
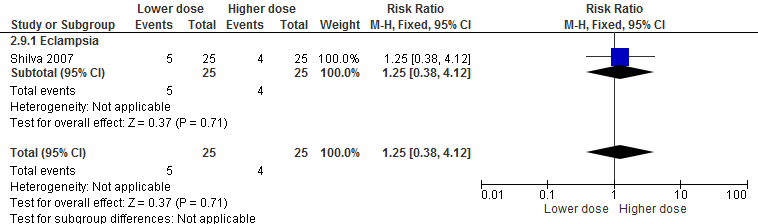
**

**Figure 68. Forest plot of Comparison: 2 Lower versus higher dose regimens of magnesium sulphate, outcome: 2.12 Jaundice**

**
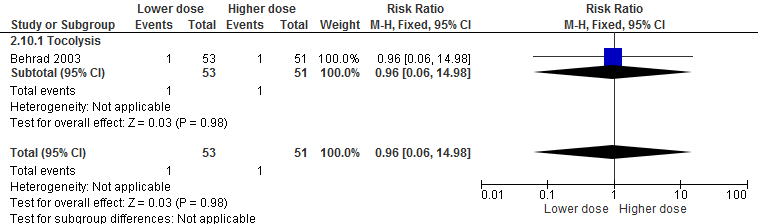
**

**Figure 69. Forest plot of Comparison: 2 Lower versus higher dose regimens of magnesium sulphate, outcome: 2.13 Hypoglycaemia**

**
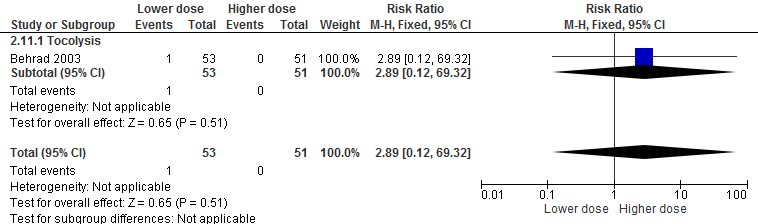
**

**Figure 70. Forest plot of Comparison: 2 Lower versus higher dose regimens of magnesium sulphate, outcome: 2.14 Hypocalcaemia**

**
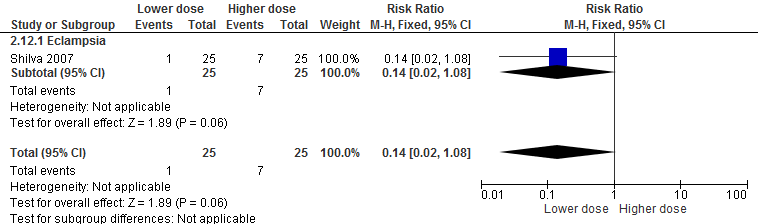
**

**Figure 71. Forest plot of Comparison: 2 Lower versus higher dose regimens of magnesium sulphate, outcome: 2.15 Hypotonia**

**
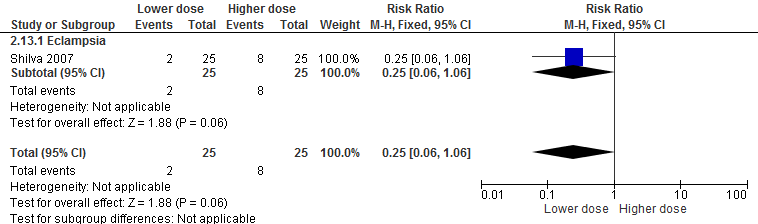
**

**Figure 72. Forest plot of Comparison: 2 Lower versus higher dose regimens of magnesium sulphate, outcome: 2.16 Requirement for calcium gluconate**

**
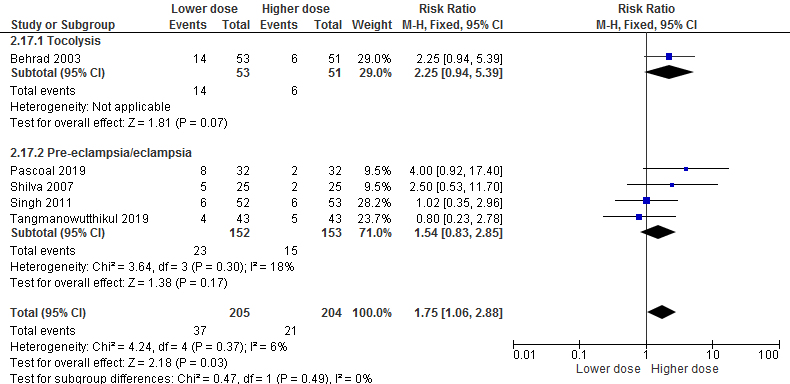
**

**Figure 73. Forest plot of Comparison: 2 Lower versus higher dose regimens of magnesium sulphate, outcome: 2.17 Neonatal intensive care unit admission**

**
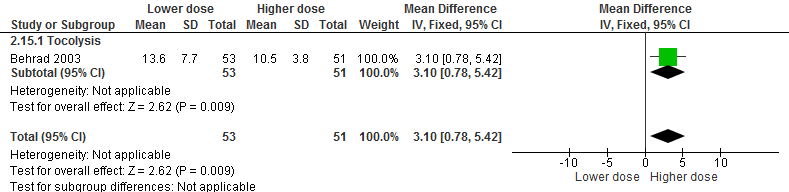
**

**Figure 74. Forest plot of Comparison: 2 Lower versus higher dose regimens of magnesium sulphate, outcome: 2.18 Neonatal intensive care unit stay (days)**

**
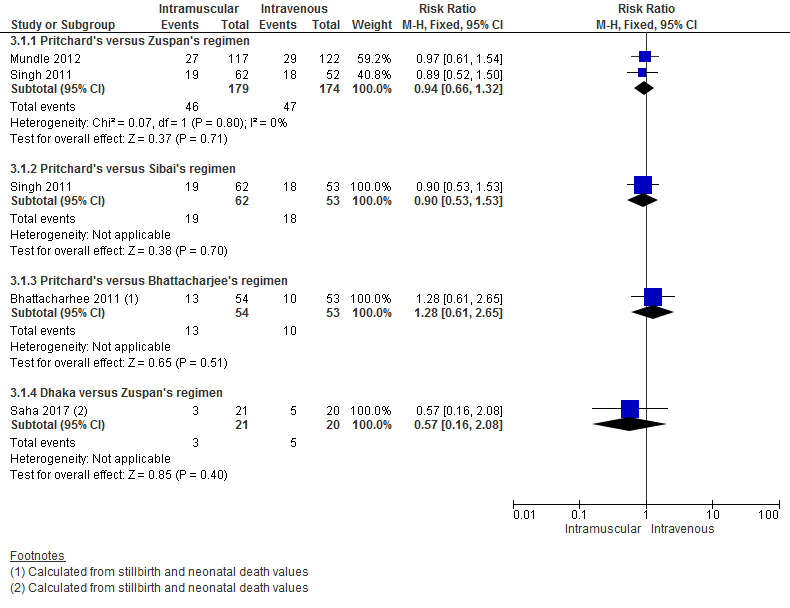
**

**Figure 75. Forest plot of Comparison: 3 Intramuscular versus intravenous maintenance dose of magnesium sulphate, outcome: 3.1 Perinatal death**

**
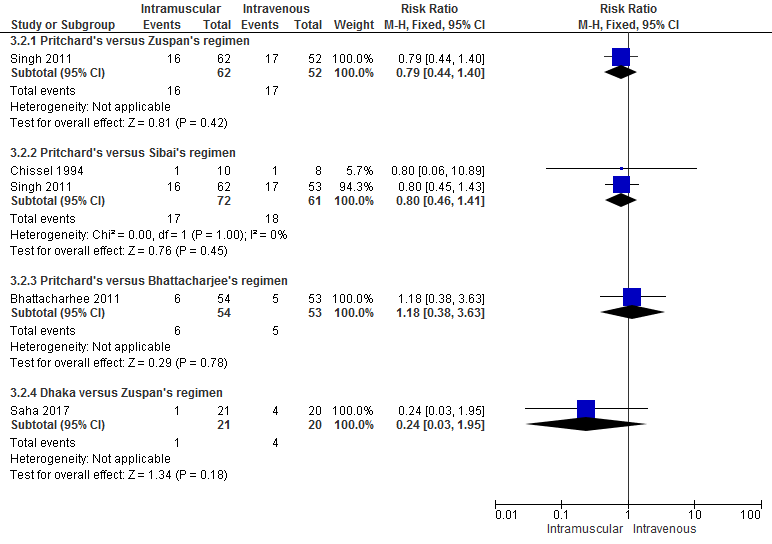
**

**Figure 76. Forest plot of Comparison: 3 Intramuscular versus intravenous maintenance dose of magnesium sulphate, outcome: 3.2 Stillbirth**

**
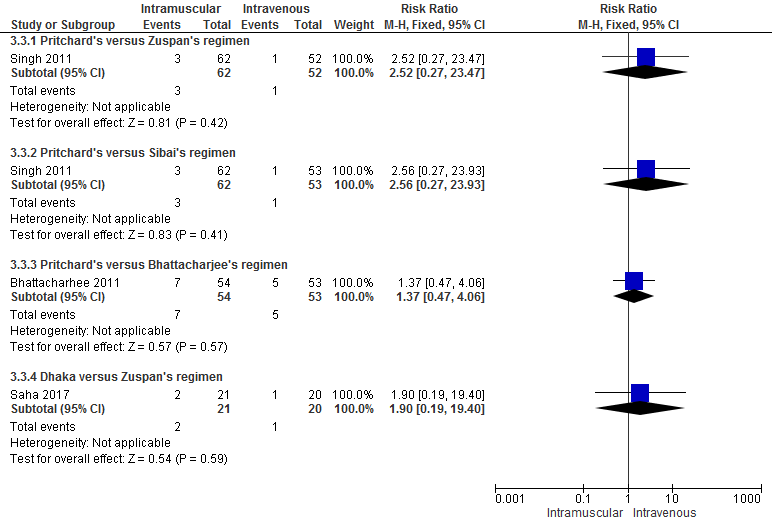
**

**Figure 77. Forest plot of Comparison: 3 Intramuscular versus intravenous maintenance dose of magnesium sulphate, outcome: 3.3 Neonatal death**

**
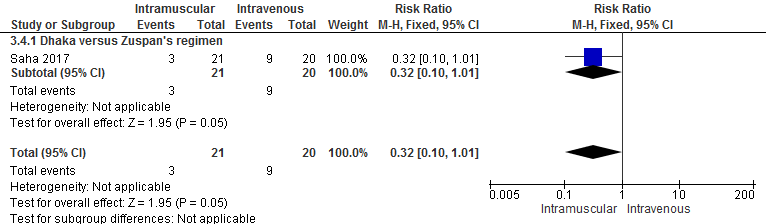
**

**Figure 78. Forest plot of Comparison: 3 Intramuscular versus intravenous maintenance dose of magnesium sulphate, outcome: 3.4 Apgar score < 7 at 1 minute**

**
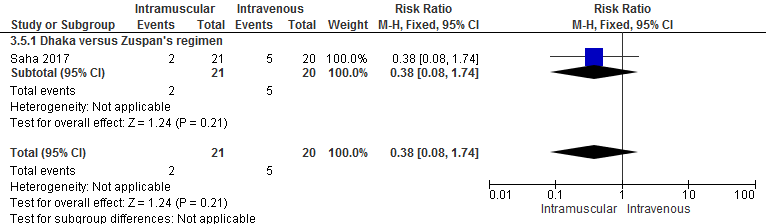
**

**Figure 79. Forest plot of Comparison: 3 Intramuscular versus intravenous maintenance dose of magnesium sulphate, outcome: 3.5 Apgar score < 7 at 5 minutes**

**
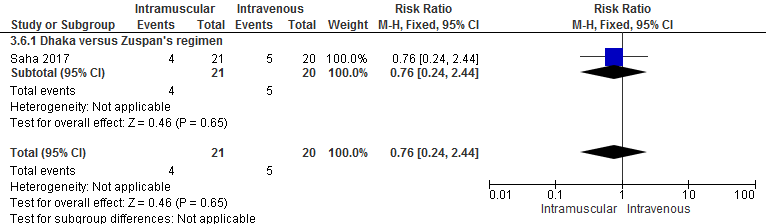
**

**Figure 80. Forest plot of Comparison: 3 Intramuscular versus intravenous maintenance dose of magnesium sulphate, outcome: 3.6 Respiratory distress syndrome**

**
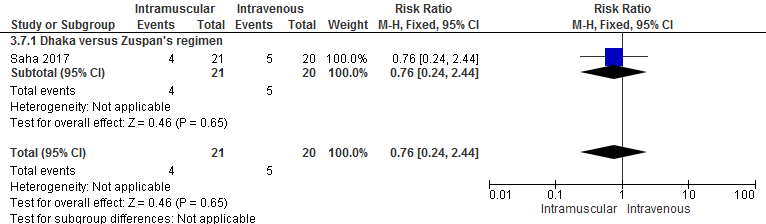
**

**Figure 81. Forest plot of Comparison: 3 Intramuscular versus intravenous maintenance dose of magnesium sulphate, outcome: 3.7 Jaundice**

**
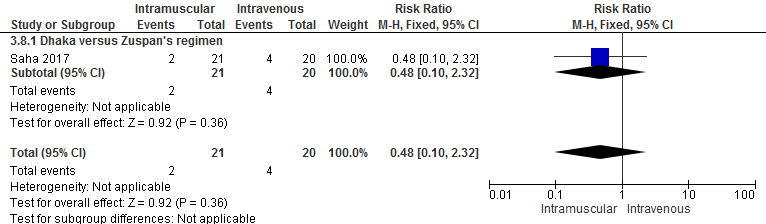
**

**Figure 82. Forest plot of Comparison: 3 Intramuscular versus intravenous maintenance dose of magnesium sulphate, outcome: 3.8 Hypotonia**

**
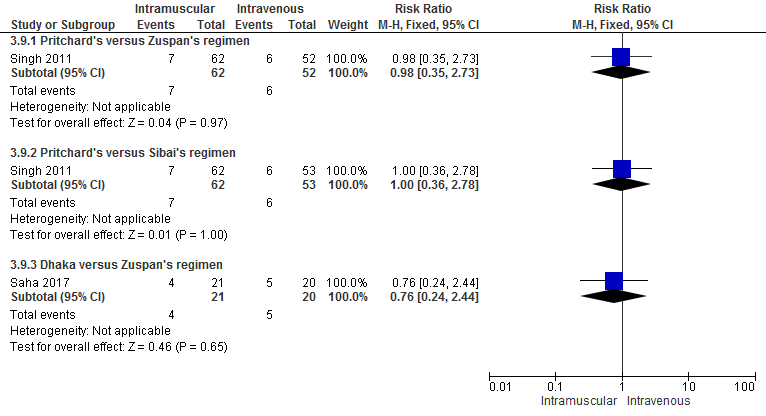
**

**Figure 83. Forest plot of Comparison: 3 Intramuscular versus intravenous maintenance dose of magnesium sulphate, outcome: 3.9 Neonatal intensive care unit admission**

**
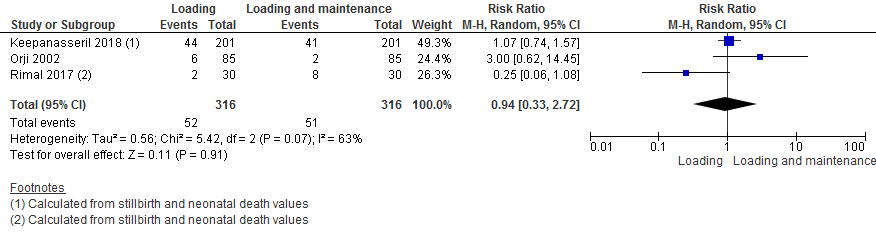
**

**Figure 84. Forest plot of Comparison: 4 Loading dose versus loading and maintenance doses of magnesium sulphate, outcome: 4.1 Perinatal death**

**
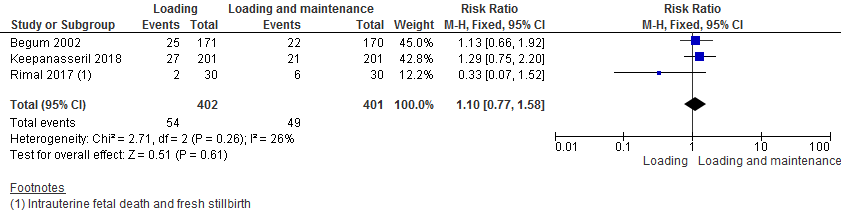
**

**Figure 85. Forest plot of Comparison: 4 Loading dose versus loading and maintenance doses of magnesium sulphate, outcome: 4.2 Stillbirth**

**
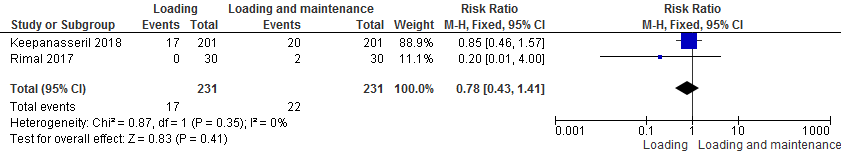
**

**Figure 86. Forest plot of Comparison: 4 Loading dose versus loading and maintenance doses of magnesium sulphate, outcome: 4.3 Neonatal death**

**
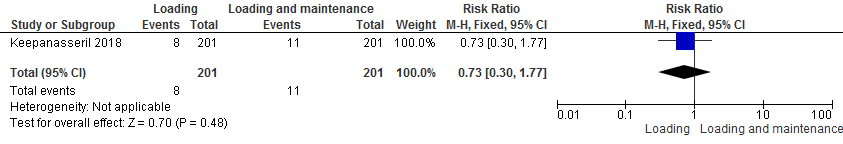
**

**Figure 87. Forest plot of Comparison: 4 Loading dose versus loading and maintenance doses of magnesium sulphate, outcome: 4.4 Neonatal death < 7 days**

**
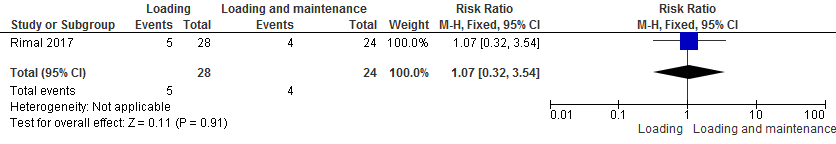
**

**Figure 88. Forest plot of Comparison: 4 Loading dose versus loading and maintenance doses of magnesium sulphate, outcome: 4.5 Apgar score < 7 at 0 minutes**

**
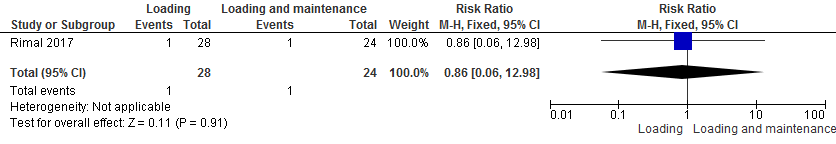
**

**Figure 89. Forest plot of Comparison: 4 Loading dose versus loading and maintenance doses of magnesium sulphate, outcome: 4.6 Apgar score < 7 at 1 minute**

**
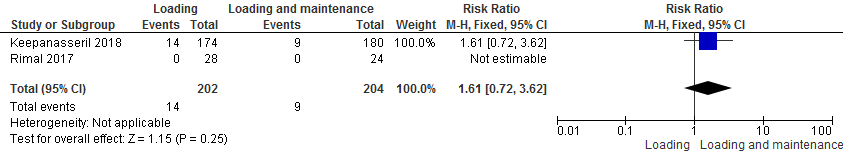
**

**Figure 90. Forest plot of Comparison: 4 Loading dose versus loading and maintenance doses of magnesium sulphate, outcome: 4.7 Apgar score < 7 at 5 minutes**

**
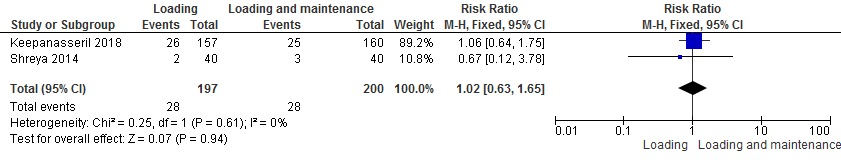
**

**Figure 91. Forest plot of Comparison: 4 Loading dose versus loading and maintenance doses of magnesium sulphate, outcome: 4.8 Neonatal intensive care unit admission for respiratory distress**

**
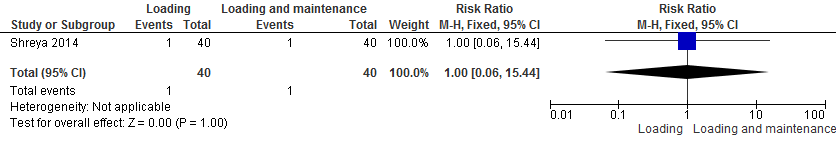
**

**Figure 92. Forest plot of Comparison: 4 Loading dose versus loading and maintenance doses of magnesium sulphate, outcome: 4.9 Neonatal intensive care unit admission for early onset sepsis**

**
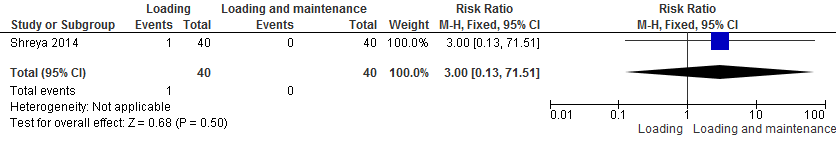
**

**Figure 93. Forest plot of Comparison: 4 Loading dose versus loading and maintenance doses of magnesium sulphate, outcome: 4.10 Neonatal intensive care unit admission for late onset sepsis**

**
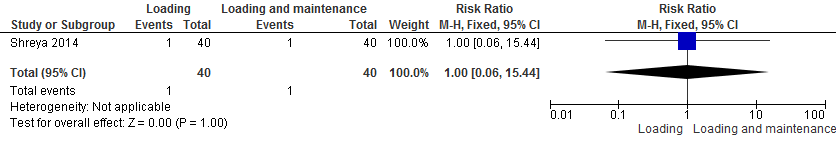
**

**Figure 94. Forest plot of Comparison: 4 Loading dose versus loading and maintenance doses of magnesium sulphate, outcome: 4.11 Neonatal intensive care unit admission for meconium aspiration syndrome**

**
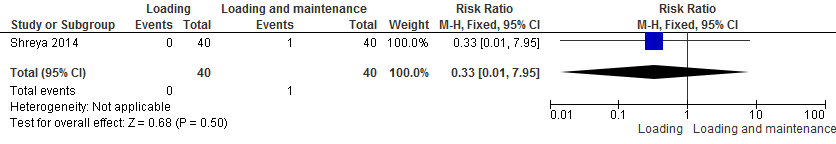
**

**Figure 95. Forest plot of Comparison: 4 Loading dose versus loading and maintenance doses of magnesium sulphate, outcome: 4.12 Neonatal intensive care unit admission for birth asphyxia**

**
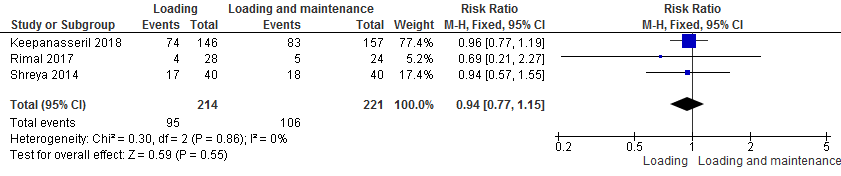
**

**Figure 96. Forest plot of Comparison: 4 Loading dose versus loading and maintenance doses of magnesium sulphate, outcome: 4.13 Neonatal intensive care unit admission**

**
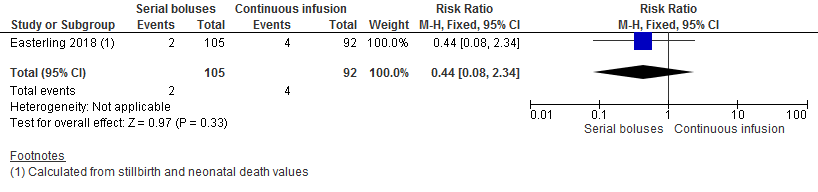
**

**Figure 97. Forest plot of Comparison: 5 Serial intravenous boluses versus continuous maintenance infusion of magnesium sulphate, outcome: 5.1 Perinatal death**

**
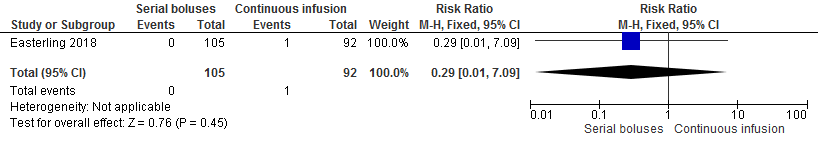
**

**Figure 98. Forest plot of Comparison: 5 Serial intravenous boluses versus continuous maintenance infusion of magnesium sulphate, outcome: 5.2 Stillbirth**

**Figure 99. Forest plot of Comparison: 5 Serial intravenous boluses versus continuous maintenance infusion of magnesium sulphate, outcome: 5.3 Neonatal death**

**Figure 100. Forest plot of Comparison: 5 Serial intravenous boluses versus continuous maintenance infusion of magnesium sulphate, outcome: 5.4 Intubated at birth**

**Figure 101. Forest plot of Comparison: 5 Serial intravenous boluses versus continuous maintenance infusion of magnesium sulphate, outcome: 5.5 Mechanical ventilation**

**Figure 102. Forest plot of Comparison: 5 Serial intravenous boluses versus continuous maintenance infusion of magnesium sulphate, outcome: 5.6 Bradycardia (< 110 beats per minute)**

**Figure 103. Forest plot of Comparison: 5 Serial intravenous boluses versus continuous maintenance infusion of magnesium sulphate, outcome: 5.7 Special care baby unit admission**

**Figure 104. Forest plot of Comparison: 6 Short versus standard maintenance course of magnesium sulphate, outcome: 6.1 Stillbirth**

**Figure 105. Forest plot of Comparison: 6 Short versus standard maintenance course of magnesium sulphate, outcome: 6.2 Birth asphyxia**

**Figure 106. Forest plot of Comparison: 7 Slower versus standard rate of loading dose of magnesium sulphate, outcome: 7.1 Stillbirth**

**Figure 107. Forest plot of Comparison: 8 Weaning versus no weaning of magnesium sulphate, outcome: 8.1 Apgar score < 7 at 5 minutes**
