# Supplementary figures and images for: Antenatal magnesium sulphate and adverse neonatal outcomes: A systematic review and meta-analysis
Source: PLoS Med. 2019 Dec 6;16(12):e1002988. doi: 10.1371/journal.pmed.1002988 (PMC6897495; doi:10.1371/journal.pmed.1002988)

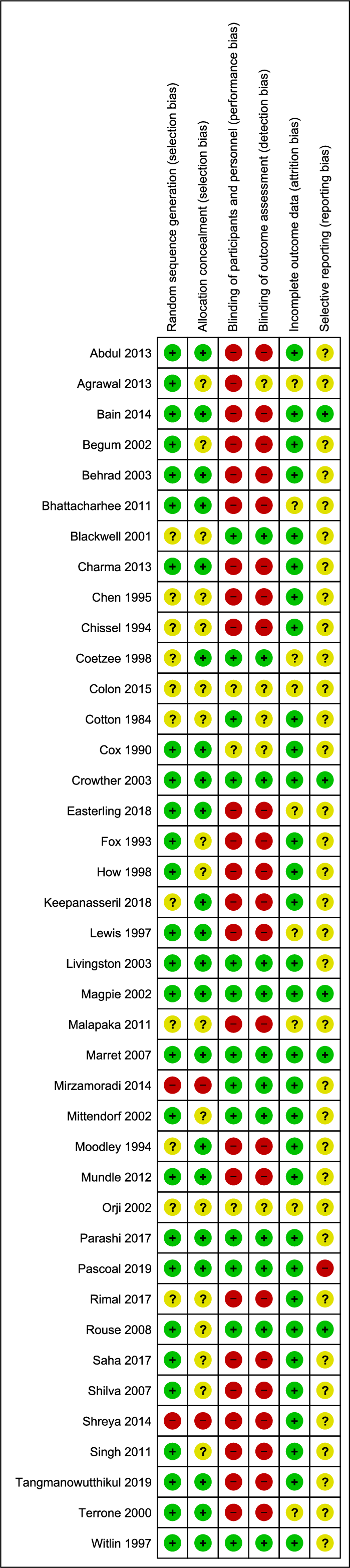

Supplement: S1 Fig — Risk of bias summary showing judgements about each risk of bias item for the 40 included randomised trials. Green represents ‘low risk of bias’; yellow, ‘unclear risk of bias’; red, ‘high risk of bias’. (TIF) [file pmed.1002988.s002.tif]
