# Supplementary material for: Antenatal magnesium sulphate and adverse neonatal outcomes: A systematic review and meta-analysis
Source: PLoS Med. 2019 Dec 6;16(12):e1002988. doi: 10.1371/journal.pmed.1002988 (PMC6897495; doi:10.1371/journal.pmed.1002988)
Supplement: S1 PRISMA Checklist — (DOCX) [file pmed.1002988.s003.docx]

| **Section/topic** | **#** | **Checklist item** | **Reported on page #** |
| --- | --- | --- | --- |
| TITLE | | |  |
| Title | 1 | Identify the report as a systematic review, meta-analysis, or both.  **Quote:** “Antenatal magnesium sulphate and adverse neonatal outcomes: a systematic review and meta-analysis” | Title |
| ABSTRACT | | |  |
| Structured summary | 2 | Provide a structured summary including, as applicable: background; objectives; data sources; study eligibility criteria, participants, and interventions; study appraisal and synthesis methods; results; limitations; conclusions and implications of key findings; systematic review registration number.  **Quote:** “Background. There is widespread, increasing use of magnesium sulphate in obstetric practice for pre-eclampsia, eclampsia, and preterm fetal neuroprotection; benefit for preventing preterm labour and birth (tocolysis) is unproven. We conducted a systematic review and meta-analysis to assess whether antenatal magnesium sulphate is associated with unintended adverse neonatal outcomes. Methods and findings. CINAHL, The Cochrane Library, LILACS, MEDLINE, Embase, TOXLINE and Web of Science, were searched (inceptions to 3 September 2019)…” | Abstract, paragraphs 1-5 |
| INTRODUCTION | | |  |
| Rationale | 3 | Describe the rationale for the review in the context of what is already known.  **Quote:** “Introduction. Antenatal magnesium sulphate is commonly used in obstetric practice. Systematic reviews and clinical practice guidelines support its use when given for maternal neuroprotection in pre-eclampsia or eclampsia [1-3], and for neuroprotection of the fetus in women at risk of preterm birth (for cerebral palsy prevention) [4-7]…” | Introduction, paragraphs 1-3 |
| Objectives | 4 | Provide an explicit statement of questions being addressed with reference to participants, interventions, comparisons, outcomes, and study design (PICOS).  **Quote:** “The aim of our study therefore was to conduct a comprehensive systematic review to assess whether antenatal magnesium sulphate is associated with including perinatal death and other unintended adverse neonatal outcomes.” | Introduction, paragraph 4 |
| METHODS | | |  |
| Protocol and registration | 5 | Indicate if a review protocol exists, if and where it can be accessed (e.g., Web address), and, if available, provide registration information including registration number.  **Quote:** “We conducted a systematic review following the Preferred Reporting Items for Systematic Reviews and Meta-analyses (PRISMA) and PRISMA Harms guidance; the relevant checklist is provided in S1 PRISMA Checklist. Prior to conduct, this systematic review was registered with PROSPERO: International prospective register of systematic reviews (CRD42013004451) [18]. The Australian Cerebral Palsy Alliance Research Foundation funded review protocol is available in S2 Text.” | Methods, paragraph 1; and S2 Text |
| Eligibility criteria | 6 | Specify study characteristics (e.g., PICOS, length of follow-up) and report characteristics (e.g., years considered, language, publication status) used as criteria for eligibility, giving rationale.  **Quote:** “**Inclusion criteria. Studies. We included randomised and quasi-randomised controlled trials as well as controlled non-randomised studies (non-randomised trials, cohort studies, case-control studies), and case reports. We excluded cross-sectional studies and case series. We included studies available as abstracts only, along with full-text publications…”** | Methods, paragraphs 3-5 |
| Information sources | 7 | Describe all information sources (e.g., databases with dates of coverage, contact with study authors to identify additional studies) in the search and date last searched.  **Quote:** “Search strategy. Comprehensive searches of the bibliographic databases CINAHL, The Cochrane Library, LILACS, MEDLINE, Embase, TOXLINE and Web of Science, were undertaken from their respective inceptions to 3 September 2019, using combinations of MeSH and free text terms. The search strategies are available in S3 Text.” | Methods, paragraph 2; and S3 Text |
| Search | 8 | Present full electronic search strategy for at least one database, including any limits used, such that it could be repeated. | S3 Text |
| Study selection | 9 | State the process for selecting studies (i.e., screening, eligibility, included in systematic review, and, if applicable, included in the meta-analysis).  **Quote:** “Study selection. After screening all titles and abstracts, we obtained full-text articles for studies which appeared to meet the inclusion criteria. All full-text articles were assessed for inclusion. Each stage was carried out by two reviewers, and we resolved any discrepancies through discussion, or if required, we consulted a third reviewer…” | Methods, paragraph 6 |
| Data collection process | 10 | Describe method of data extraction from reports (e.g., piloted forms, independently, in duplicate) and any processes for obtaining and confirming data from investigators.  **Quote:** “Data extraction and management. For included studies, data were extracted using a standardised form, including information regarding design, participants, the magnesium sulphate regimen(s), the control/comparison if applicable, neonatal adverse outcomes reported, results relevant to the review and the risk of bias…” | Methods, paragraph 7 |
| Data items | 11 | List and define all variables for which data were sought (e.g., PICOS, funding sources) and any assumptions and simplifications made. | Methods, paragraph 7 |
| Risk of bias in individual studies | 12 | Describe methods used for assessing risk of bias of individual studies (including specification of whether this was done at the study or outcome level), and how this information is to be used in any data synthesis.  **Quote:** “Assessment of risk of bias. Quality appraisal of intervention studies was undertaken utilising established guidelines provided in the Cochrane Handbook for Systematic Reviews of Interventions [19]. The quality assessment of observational studies was guided by the Response to Intervention (RTI) International Item Bank for Assessing Risk of Bias and Confounding for Observational Studies of Interventions or Exposures [20].” | Methods, paragraph 8 |
| Summary measures | 13 | State the principal summary measures (e.g., risk ratio, difference in means).  **Quote:** “Data synthesis and analysis. Data analyses were undertaken by study design. Statistical analyses for randomised trials were performed using Review Manager, version 5.3 [21]. We presented quantitative data from individual studies as risk ratios (RR) for dichotomous outcomes and mean differences (MD) for continuous outcomes, with 95% confidence intervals (CI)...” | Methods, paragraph 9 |
| Synthesis of results | 14 | Describe the methods of handling data and combining results of studies, if done, including measures of consistency (e.g., I^2^) for each meta-analysis.  **Quote:** “For all outcomes, we carried out analyses as far as possible on an intention-to-treat basis. Pooled estimates were calculated using fixed-effect meta-analysis (Mantel-Haenszel method) where there was a sufficient quantity of data, with clinical homogeneity…” | Methods, paragraphs 9-11 |
| Risk of bias across studies | 15 | Specify any assessment of risk of bias that may affect the cumulative evidence (e.g., publication bias, selective reporting within studies). | Methods, paragraph 8 |
| Additional analyses | 16 | Describe methods of additional analyses (e.g., sensitivity or subgroup analyses, meta-regression), if done, indicating which were pre-specified.  **Quote:** “For our primary review outcome (perinatal death) and other mortality outcomes, we conducted subgroup analyses based on indication for use, and characteristics of the magnesium sulphate loading and maintenance dose regimens, as these factors were considered likely to influence outcomes…” | Methods, paragraph 10 |
| RESULTS | | |  |
| Study selection | 17 | Give numbers of studies screened, assessed for eligibility, and included in the review, with reasons for exclusions at each stage, ideally with a flow diagram.  **Quote:** “Results. Study selection. The results of the search strategy, including the sources of the studies, their assessment and final inclusion are shown in Fig 1. The database searching identified 5890 records, and other searching identified a further 11 records. Review of the titles and abstracts and exclusion of irrelevant and duplicate records yielded 777. Of these, we excluded 572 for the documented reasons (see S4 Text for list of records excluded due absence of an English translation). We included a total of 205 articles, relating to 197 studies. See S5 Text for references to all included studies.” | Results, paragraph 1; and Fig 1 |
| Study characteristics | 18 | For each study, present characteristics for which data were extracted (e.g., study size, PICOS, follow-up period) and provide the citations.  **Quote:** “Evidence from randomised controlled trials. Forty randomised trials were included, the characteristics of which are detailed in S6 Table, and the risk of bias assessments summarised in Fig 2, S7 Fig, and S8 Table [22-61]…”  **Quote:** “Evidence from non-randomised comparative studies. One hundred and thirty-eight non-randomised studies were included: five non-randomised trials, 35 prospective cohort studies (seven with nested case-control analyses), 82 retrospective cohort studies (16 with nested case-control analyses), eight non-concurrent cohort studies, and eight case-control studies [62-199]. The characteristics of the studies, and risk of bias assessments are detailed in S6 Table and S8 Table…”  **Quote:** “Evidence from case reports. Nineteen reports describing a total of 134 babies exposed to antenatal magnesium sulphate with adverse outcomes were included [200-218] (see Table 9; the detailed characteristics of cases are presented in S11 Table).” | Results paragraphs 2, 27-28 and 36; S5 Text, S6 Table and S11 Table |
| Risk of bias within studies | 19 | Present data on risk of bias of each study and, if available, any outcome level assessment (see item 12).  **Quote:** “Forty randomised trials were included, the characteristics of which are detailed in S6 Table, and the risk of bias assessments summarised in Fig 2, S7 Fig, and S8 Table [22-61]…”  **Quote:** “One hundred and thirty-eight non-randomised studies were included… The characteristics of the studies, and risk of bias assessments are detailed in S6 Table and S8 Table…” | Results paragraph 2-3, 27-28; Fig 2, S7 Fig and S8 Table |
| Results of individual studies | 20 | For all outcomes considered (benefits or harms), present, for each study: (a) simple summary data for each intervention group (b) effect estimates and confidence intervals, ideally with a forest plot. | Tables 1, 4 and 6; S9 Appendix |
| Synthesis of results | 21 | Present results of each meta-analysis done, including confidence intervals and measures of consistency. | Tables 1, 4, 6; S9 Appendix |
| Risk of bias across studies | 22 | Present results of any assessment of risk of bias across studies (see Item 15). | Results paragraph 2-3 and 27-28; Fig 2 |
| Additional analysis | 23 | Give results of additional analyses, if done (e.g., sensitivity or subgroup analyses, meta-regression [see Item 16]).  **Quote:** “Subgroup analyses: When considering indication for use, the ‘tocolysis’ subgroup showed an increase in perinatal death (RR 7.99; 95% CI 1.00 to 63.49; 2 trials, 257 babies; Analysis 1.1.1) which was not observed in the ‘pre-eclampsia’ or ‘fetal neuroprotection’ subgroups...” | Results, paragraphs 5 and 9; Tables 2, 3, and 5 |
| DISCUSSION | | |  |
| Summary of evidence | 24 | Summarize the main findings including the strength of evidence for each main outcome; consider their relevance to key groups (e.g., healthcare providers, users, and policy makers).  **Quote:** “Discussion. Overall, no clear difference in our primary review outcome, perinatal death, was shown...” | Discussion, paragraphs 1-6 |
| Limitations | 25 | Discuss limitations at study and outcome level (e.g., risk of bias), and at review-level (e.g., incomplete retrieval of identified research, reporting bias).  **Quote:** “Strengths and limitations. The main limitations of our review relate to missing data for important outcomes across most studies, heterogeneity of included studies, and inclusion of published data only…” | Discussion, paragraphs 7-11 |
| Conclusions | 26 | Provide a general interpretation of the results in the context of other evidence, and implications for future research.  **Quote:** “In conclusion, our findings do not support any clear associations between perinatal death nor other adverse neonatal outcomes and antenatal magnesium sulphate exposure when given for the beneficial indications of maternal neuroprotection in pre-eclampsia/eclampsia, and fetal neuroprotection in cerebral palsy prevention…” | Discussion, paragraph 12 |
| FUNDING | | |  |
| Funding | 27 | Describe sources of funding for the systematic review and other support (e.g., supply of data); role of funders for the systematic review.  **Quote:** “This work was carried out with funding from the Cerebral Palsy Alliance Research Foundation, Australia (https://research.cerebralpalsy.org.au/), grant PG2015 (ES, PM, MM, CC). The funder had no role in study design, data collection and analysis, decision to publish, or preparation of the manuscript.” | This is provided in the financial disclosure section of the manuscript submission system, as requested by the journal. |

*From:*  Moher D, Liberati A, Tetzlaff J, Altman DG, The PRISMA Group (2009). Preferred Reporting Items for Systematic Reviews and Meta-Analyses: The PRISMA Statement. PLoS Med 6(6): e1000097. doi:10.1371/journal.pmed1000097

For more information, visit: **www.prisma-statement.org**.
