# Supplementary material for: Antenatal magnesium sulphate and adverse neonatal outcomes: A systematic review and meta-analysis
Source: PLoS Med. 2019 Dec 6;16(12):e1002988. doi: 10.1371/journal.pmed.1002988 (PMC6897495; doi:10.1371/journal.pmed.1002988)
Supplement: S1 Table — (DOCX) [file pmed.1002988.s004.docx]

**Characteristics of included studies – randomised controlled trials**

| **Study; design** | **Setting** | **Participants** | **MgSO4 indication** | **Relevant comparison groups** | **Outcome measure(s)** | **Funding and conflicts** |
| --- | --- | --- | --- | --- | --- | --- |
| Abdul 2013; RCT | Nigeria  2008 | N = 72 women and their babies  Inclusions: women with antepartum, intrapartum or postpartum E including imminent E  Exclusions: women with E in critical conditions, with hypotension and low RR | E | Lower dose regimen: modified ‘Dhaka regimen’: 4 g IV LD and 5 g IM LD; 2.5 g/4 hours IM MD for 24 hours post birth or last convulsion, N = 39 women and their babies  Higher dose regimen: ‘Pritchard’s regimen’’: 4 g IV LD and 10 g IM LD; 5 g IM/4 hours MD for 24 hours post birth or convulsion, N = 33 women and their babies  All women: 2 g IV given for breakthrough convulsions | Perinatal death | Funding: NR  Conflicts: none |
| Agrawal 2013; RCTAbstract | India  1 year | N = 75 women and their babies  Inclusions: women with E  Exclusions: NR | E | Lower dose regimen: 4 g IV LD and 6 g IM LD; 3 g/4 hour IM MD until 24 hours after birth, N = 36 women and their babies  Higher dose regimen: “standard dose… Pritchard regimen”, N = 39 women and their babies | Not in meta-analysis: “neonatal outcome” | NR |
| Bain 2014; RCT | Australia  Time period NR | N = 51 women and their babies  Inclusions: women with a single or twin pregnancy at < 30 weeks GA, if birth was planned or expected within 24 hours  Exclusions: women in the 2^nd^ stage of labour, who had already received MgSO4, or had any of the following contraindications (absent patellar reflexes, hypocalcaemia, RR < 16 breaths/minute, renal failure, urine output < 100 mL during last 4 hours) | FN | Slower rate of LD: 4 g IV LD over 60 minutes, N = 25 women and their babies  Standard rate of LD: 4 g IV LD over 20 minutes, N = 26 women and their babies  Both groups: 1 g/hour IV MD until birth or for up to 24 hours | Stillbirth | Funding: 1 author supported by The University of Adelaide  Conflicts: 2 authors report none; 2 authors were also authors on a relevant Cochrane review; 1 author was the PI of the ACTOMgSO4 RCT; 2 authors were members of a guideline panel for relevant national guidelines |
| Begum 2002; RCT | Bangladesh  1999 | N = 401 women and their babies  Inclusions: women with E eligible for MgSO4  Exclusions: contraindications for MgSO4 therapy (e.g. oliguria, renal failure, absence tendon reflex), comatose patients, women who received MgSO4 from outside, women whose pregnancy was continued | E | LD only: 4 g IV LD over 15-20 minutes and 6 g IM LD, N = 202 women and their babies  LD and MD: 4 g IV LD over 15-20 minutes and 6 g IM LD; 2.5 g/4 hours IM MD for 24 hours after birth or last convulsion, N = 199 women and their babies  All women: for recurrent convulsion, women in LD only group received 2.5 g IV and MD was started for 24 hours; in women receiving MD already, a further 2.5 g IV given and MD continued | Stillbirth | NR |
| Behrad 2003; RCT | Iran  2000-2001 | N = 100 women and their babies  Inclusions: singleton or twin gestation between 24 and 35 weeks GA, spontaneous preterm labour (uterine contractions > 4 per 20 minutes along with 1 of the following observations: cervical dilatation of ≥ 1 cm but < 5 cm, cervical effacement ≥ 80% and/or progressive cervical dilation and effacement), ability to provide informed consent  Exclusions: higher order multiple gestations, ROM, non-reassuring fetal assessment (abnormalities of the fetal heart rate pattern), evidence of intrauterine infection (temperature of 38 or higher, leucocytosis, uterine tenderness, malodorous discharge), vaginal bleeding, patients with history of DM, myasthenia gravis or any other neuromuscular diseases, impaired renal function, hypotension, maternal bradycardia, atrioventricular block, and inability or refusal to provide informed consent | T | Lower dose regimen: 4 g IV LD over 20 minutes; 2 g/hour MD, N = 50 women and their babies  Higher dose regimen: 6 g IV LD over 20 minutes; 2 g/hour IV MD, increased if required up to 4 g/hour, N = 50 women and their babies  All women: successful T = < 4 contractions/hour with no further advancement in cervical dilation/effacement; failed T (and cessation of treatment) = dilation progressed to 6 cm and/or contractions persisted > 30 minutes at maximum dose | Perinatal death, stillbirth, neonatal death, Apgar score < 8 at 1 minute, Apgar score < 8 at 5 minutes, RDS, bradycardia, hypoglycaemia, hypocalcaemia, NICU admission, NICU stay (mean ± SD) (days) | NR |
| Bhattacharhee 2011; RCT | India  2007-2009 | N = 144 women and their babies  Inclusions: women admitted with antepartum, intrapartum or postpartum E  Exclusions: E with added complications (e.g. cerebrovascular accident, heart failure, renal failure, pulmonary oedema), referred cases who had already received initial dosage of MgSO4 at the referring centre | E | IM MD: ‘Pritchard’s regimen’: 4 g IV slow bolus LD and 10 g IM LD; 5 g/4 hours IM MD, N = 72 women and their babies  IV MD: 4 g IV slow bolus LD; 6 g/8 hours IV LD, N = 72 women and their babies  All women: 2 g IV bolus if needed for recurrent convulsions | Perinatal death, stillbirth, neonatal death | Funding: NR  Conflicts: none |
| Blackwell 2001; RCT | Author from USA  Time period NR | N = 22 women and their babies  Inclusions: women with singleton pregnancies at > 32 weeks GA complicated by PPROM (< 37 weeks GA), PROM > 18 hours (GA > 18 weeks), or clinical chorioamnionitis  Exclusions: any indication for MgSO4 therapy (seizure prophylaxis or T), known maternal hypersensitivity to MgSO4, fetal structural defects, FGR (birthweight < 10^th^ percentile for GA), systemic maternal infection (e.g. pneumonia or pyelonephritis), advanced cervical dilation (≥ 8 cm) or imminent delivery, women with medical disorders such as any renal, cardiac or pulmonary disease, pulmonary hypertension or myasthenia gravis | FN | MgSO4: 6 g IV LD over 20-30 minutes; 2 g/hour IV MD until birth, N = 11 women and their babies  Placebo: matched volume of Ringer’s lactate solution of 20-30 minutes as a LD; continuous infusion of matched volume Ringer’s lactate solution as MD until birth N = 11 women and their babies | Neonatal death, CLD (BPD), NEC, IVH grade 3/4 (‘major’) | NR |
| Charma 2013; RCT | Nigeria  2011 | N = 112 women and their babies  Inclusions: women with E  Exclusion criteria: women with E who had received any anticonvulsant elsewhere before referral; added complications like stroke, renal failure and heart failure | E | Short MD: 4 g IV slow bolus LD and 10 g IM LD; 5 g/4 hours IM MD, for 2 doses only, N = 56 women and their babies  Standard MD: ‘Prichard’s regimen’: 4 g IV slow bolus LD and 10 g IM LD; 5 g/4 hours IM MD for 24 hours after birth or last convulsion, N = 56 women and their babies  All women: 2 g IV for recurrent convulsions | Stillbirth, birth asphyxia | Funding: NR  Conflicts: none |
| Chen 1995; RCT | Taiwan  1989-1992 | N = 64 women and their babies  Inclusions: signs of severe hypertension (BP > 150/100 mmHg) with 1 or more features of severe PE (systolic BP 166 mmHg or higher; diastolic BP 110 mmHg or higher; proteinuria persistent 2+ or more; oliguria 500 mL or less in 24 hours; serum creatinine elevated; thrombocytopenia; hyperbilirubinemia; sGOT elevation marked; visual disturbance; headache; upper abdominal pain; pulmonary oedema or cyanosis; obvious FGR)  Exclusions: IUD, chronic hypertension superimposed with PE, E at admission | PE | MgSO4: 4 g IV LD over 10 minutes; 1 g/hour IV MD until 1 day after birth, N = 34 women and their babies  No treatment: no MgSO4, N = 30 women and their babies | Apgar score ≤ 6 at 1 minute | NR |
| Chissel 1994; RCT | South Africa  Time period NR | N = 17 women and their babies  Inclusions: women with severe PE and imminent E; proteinuria of at least 1+ assessed by a semi quantitative dipstick method, and diastolic BP of 120 mmHg or more which did not settle during a 4 hour observation period  Exclusions: NR | PE | IM MD: ‘Pritchard’s regimen’: 4 g IV LD over 15 minutes and 10 g IM LD; 5 g/4 hours IM MD for 24 hours, providing delivery had occurred, N = 9 women and their babies  IV MD: ‘Sibai’s regimen’: 6 g IV LD over 15 minutes; 2 g/hour IV MD for 24 hours, providing delivery had occurred N = 8 women and their babies | Stillbirth | Funding: support from South African Medical Research Council  Conflicts: NR |
| Coetzee 1998; RCT | South Africa  1991 | N = 822 women and their babies  Inclusions: women with severe PE (2 or more of the following: diastolic BP ≥ 110 mmHg, significant proteinuria, and symptoms of imminent E) where a decision to terminate pregnancy had been made [note: twin pregnancies were included]  Exclusions: women younger than 16 years, already receiving MgSO4 or other anticonvulsants | PE | MgSO4: 4 g IV LD over 20 minutes; 1 g/hour IV MD until 24 hours after birth, N = 345 women and their babies (analysed)  Placebo: saline placebo as above, N= 240 women and their babies (analysed) | Stillbirth | NR |
| Colon 2015; RCTAbstract | Authors from USA  Time period NR | N = 30 women and their babies  Inclusions: women between 24-34 weeks GA presenting with vaginal bleeding and uterine contractions and diagnosed with non-severe placental abruption  Exclusion criteria: NR | T | MgSO4: IV, N = 15 women and their babies  Placebo: IV placebo, N = 15 women and their babies | Not in meta-analysis: NICU admission | NR |
| Cotton 1984; RCT | Authors from USA  Time period NR | N = 54 women and their babies  Inclusions: women between 26 and 34 weeks GA (and estimated fetal weight between 750-2000 g) in preterm labour (where, following hydration, persistent uterine contractions occurred ≥ 3 in 10 minutes, and cervical examination suggested active labour (any of the following: 1) evidence of progressive cervical dilatation overtime; 2) a dilatation of 2 cm or greater; 3) cervical effacement of ≥ 80%; or 4) spontaneous ROM)); with singleton or twin pregnancies  Exclusions: women with a cervical dilatation > 4 cm; women with evidence of fetal pulmonary maturity or bacteria on Gram’s stain | T | MgSO4: 4 g IV LD over 15 minutes; 2 g/hour IV MD – women with intact membranes, discontinued after 12 hours if no uterine activity; in women with ROM, continued for > 48 hours, N = 16 women and their babies  Placebo: continuous IV infusion of dextrose in lactated Ringer’s solution at 125 ml/hour, N = 19 women and their babies  [19 women and their babies in terbutaline group not considered further] | Neonatal death, RDS, NEC, sepsis (positive culture), hypoglycaemia on NICU admission, PDA (requiring either medical or surgical treatment), seizures, IVH (ICH) | Funding: supported by NIH and the Ariel Kaare Rosholt Weathers-Lowin Medical Research Foundation  Conflicts: NR |
| Cox 1990; RCT | USA  1987-1989 | N = 156 women and their babies  Inclusions: preterm labour (defined by regular uterine contractions associated with cervical dilation ≥ 1 cm but < 5 cm); GA between 24-34 weeks; intact fetal membranes; no maternal or fetal complications necessitating delivery (singleton and twin pregnancies)  Exclusions: NR | T | MgSO4: 4 g IV LD; 2 g/hour IV MD, increased to 3 g/hour if contractions persisted after 1 hour; continued for 24 hours (with re-treatment later if needed), N = 76 women and their babies  Placebo: IV physiologic saline solution at 80 mL/hour for 24 hours. N = 80 women and their babies | Perinatal death, stillbirth, neonatal death, death > 28 days, before discharge, RDS (requiring ventilator) NEC, IVH (ICH), NICU admission, hospital stay (mean ± SD) (days) | NR |
| Crowther 2003; RCT | Australia and New Zealand  1996-2000 | N = 1062 women and their babies  Inclusions: women pregnant with single, twin, triplet or quadruplet foetuses < than 30 weeks GA, where birth was planned or expected within 24 hours  Exclusions: women in the 2^nd^ stage of labour, women who had received MgSO4 therapy in this pregnancy, or with contraindications to MgSO4 therapy (RR < 16/minute, absent patellar reflexes, urine output < 100 mL in the previous 4 hours, renal failure, hypocalcaemia) | FN | MgSO4: 4 g IV LD over 20 minutes; 1 g/hour IV MD until birth or for up to 24 hours, N = 535 women and their babies  Placebo: 8 mL sodium chloride solution/20 minutes; 2 mL/hour until birth or for up to 24 hours, N = 527 women and their babies | Perinatal death, stillbirth, neonatal death, death > 28 days, before discharge, Apgar score < 7 at 5 minutes, MV, CLD, NEC, IVH, IVH grade 3/4, PVL  Not in meta-analyses: length of hospital stay (median, range) (days)  Additional outcomes from Paradisis 2012 (N = 87 babies), nested within Crowther: PDA treated, surfactant, volume expansion, dobutamine, dopamine, any inotrope, SVC flow < 41 mL/kg/min 1^st^ 24 hours, RVO < 120 mL/kg/min 1^st^ 24 hours, mean BP < 10^th^ centile 1^st^ 24 hours, pneumothorax, pulmonary haemorrhage | Funding: supported by NHMRC, Channel 7 Research Foundation of SA, Queen Victoria Hospital Research Foundation, and The University of Adelaide  Conflicts: none |
| Easterling 2018; RCT | Egypt  2015-2016 | N = 200 women and their babies  Inclusions: women with severe PE, pregnant or ≤ 24 hours postpartum, deemed to benefit from treatment with MgSO4  Exclusions: women who had experienced an E seizure, who had received MgSO4 within 24 hours of enrolment, or who had a serum creatinine > 1.2 mg/dL at the time of enrolment | PE | Serial IV bolus regimen: 6 g IV LD over 30 minutes using Springfusor spring-loaded pump, through flow control tubing; 2 g IV bolus over 10 minutes every 2 hours as MD, N = 100 women and their babies  Continuous infusion regimen: 4 g IV LD over 20 minutes; 1 g/hour IV MD by mini-drip, N = 100 women and their babies | Perinatal death, stillbirth, neonatal death, intubated at delivery, MV, bradycardia, SCBU admission | Funding: supported by Merck for Mothers  Conflicts: none |
| Fox 1993; RCT | Authors from USA  Time period NR | N = 90 women and their babies  Inclusions: patients between 34 and 37 weeks GA, > 15 but < 45 years of age, in documented preterm labour with cervical change, capable of giving informed consent  Exclusions: cervical dilatation ≥ 3 cm, unknown GA, ROM, or with medical or obstetric conditions necessitation delivery (such as PIH, cardiac disease, fetal distress, or haemorrhage); evidence of suspected anomalies and maternal allergy to MgSO4 | T | MgSO4: 4 g IV bolus LD; 2-4 g/hour IV MD until uterine quiescence [oral magnesium gluconate used as further MD], N = 45 women and their babies  No treatment: conservative management, with hydration, sedation and observation; labour was allowed to continue without intervention after admission to the labour suite, N = 45 women and their babies | Perinatal death, stillbirth, neonatal death, RDS, TTN, NEC, poor feeding, hyperbilirubinaemia, IVH, IVH grade 3/4, hospital stay (mean ± SD) (days)  Not in meta-analyses: days with the use of the ventilator (mean), NICU time for infants who did have complications (mean) | Funding: supported by Vicksburg Hospital Medical Foundation  Conflicts: NR |
| How 1998; RCT | USA  1992-1995 | N = 145 women and their babies  Inclusions: patients between 24-34 completed weeks GA with PPROM (documented by obvious vaginal pooling and/or confirmed by ferning and alkaline pH using nitrazine paper) (singleton and twin pregnancies)  Exclusions: evidence of chorioamnionitis and/or fetal stress or advanced active labour (≥ 3 cm dilatation by sterile speculum exam) on admittance; patient’s refusal; obstetrical indications for expedient delivery (PE/E, abruptio placenta, lethal anomalies, severe IUGR) | T | MgSO4: in the presence of ≥ 6 contractions/hour: 6 g IV LD; 2 g/hour IV MD, increased up to 5 g/hour; maintained for 4 hours, gradually decreased by 1-2 g/hour, maintained for 6-8 hours before discontinuation (could be re-started), N = 78 women and their babies  No treatment: expectant management, N = 67 women and their babies | Stillbirth, death > 28 days, before discharge, RDS (HMD), TTN, MV, oxygen required, CLD, apnoea and bradycardia, NEC, sepsis, IVH  Not in meta-analyses: length of nursery stay (median, IQR, range) (days), ventilator support (median, IQR, range) (days), oxygen required (median, IQR, range) (days) | NR |
| Keepanasseril 2018; RCT | India  2011-2013 | N = 402 women and their babies  Inclusions: women with a singleton pregnancy complicated by severe PE  Exclusions: women with plasma creatinine value ≥ 2 mg/dL, deranged coagulation profile, platelet count < 50,000/mm3, past diagnosis of myasthenia gravis, or seizure disorders, already on MgSO4 prophylaxis on admission | PE | LD only: 4 g IV LD over 10-15 minutes and 6 g IM LD, N = 201 women and their babies  LD and MD: ‘Dhaka regimen’: 4 g IV LD over 10-15 minutes and 6 g IM LD; 2.5 g/4 hours IM MD, for 24 hours postpartum, N = 201 women and their babies  All women: 2 g IV given for recurrence of convulsions, and 2.5 g/4 hours IM MD continued until 24 hours post birth or last convulsion | Perinatal death, stillbirth, neonatal death, neonatal death < 7 days, Apgar score < 7 at 5 minutes, NICU admission for RD, NICU admission | Funding: NR  Conflicts: none |
| Lewis 1997; RCT | USA  1993-1996 | N = 144 women and their babies  Inclusions: women with intact membranes between 24-35 weeks GA who had undergone successful single-agent tocolysis with MgSO4; preterm labour: defined as regular uterine contractions with either documented cervical change or a cervix dilated ≥ 2 cm and 75% effaced; successful tocolysis: uterine quiescence for ≥ 12 hours with no further cervical change (requiring approximately 24 hours for completion)  Exclusions: NR | T | Weaning: MgSO4 weaned by approximately 1 g every 4 hours; when the dose was < 1 g/hour it was discontinued, and women were monitored for 4 hours, N = 72 women and their babies analysed  No weaning: MgSO4 abruptly stopped and women observed for 4 hours, N = 69 women and their babies analysed  All women had received: 6 g IV LD over 20 minutes; 2-3.5 g/hour IV MD | Apgar score < 7 at 5 minutes | NR |
| Livingston 2003; RCT | USA  1996-2001 | N = 222 women and their babies  Inclusions: women at term or preterm, who developed mild PE (systolic BP ≥ 140 mmHg or diastolic BP ≥ 90 mmHg on 2 occasions ≥ 6 hours apart, in association with new onset proteinuria, defined as +1 or greater on dipstick on ≥ 2 occasions) before onset of labour; women admitted for planned caesarean birth and who developed mild PE during the postpartum period were also included (singleton and twin pregnancies)  Exclusions: women with chronic hypertension or severe PE | PE | MgSO4: 6 g IV LD over 20 minutes; 2 g/hour IV MD, continued for 12 hours, or until 12 hours postpartum, N = 109 women and their babies  Placebo: identical administration of indistinguishable IV saline, N = 113 women and their babies | Meconium at delivery | NR |
| Magpie 2002; RCT | 33 counties (Africa, the Americas, Asia-Pacific region, Europe)  1998-2001 | N = 10,141 women and their babies  Inclusions: women with PE and there was uncertainty about whether to use MgSO4; irrespective of whether they had had an anticonvulsant at a referring hospital, or whether the pregnancy was singleton or multiple (the woman had not given birth, or was 24 hours or less postpartum; BP was 90 mm Hg diastolic or 140 mm Hg systolic or more on ≥ 2 occasions; proteinuria was ≥ 1+; and there was clinical uncertainty about whether MgSO4 would be beneficial)  Exclusions: hypersensitivity to MgSO4, hepatic coma with a risk of renal failure, or myasthenia gravis | PE | MgSO4: 4 g IV LD over 10-15 minutes; MD of either: 1 g /hour IV for 24 hours, or 5 g/4 hours IM (plus 10 g IM with LD) for 24 hours, N = 5071 women and their babies  Placebo: as above, N = 5070 women and their babies  All women: trial treatment could be continued > 24 hours if considered necessary by clinician; if woman had a convulsion, treatment stopped and MgSO4 used | Perinatal death, stillbirth, neonatal death, death > 28 days, before discharge, early neonatal death, late neonatal death, Apgar score < 7 at 5 minutes, intubated at delivery, MV, seizures, persistent parenchymal echogenicity, abnormal ventriculomegaly, NICU admission, SCBU admission > 7 days or death, SCBU admission > 7 days, still in hospital at 6 weeks | Funding: UK MRC, UK Department for International Development, the UNDP/UNFPA/WHO/World Bank Special Programme of Research, Development and Research Training in Human Reproduction  Conflicts: none |
| Malapaka 2011; RCT | India  2007-2009 | N = 126 women and their babies  Inclusions: women with E or imminent E  Exclusions: NR | E | Lower dose regimen: for women with E: 4 g IV LD over 15-20 minutes; 2 g IM or slow IV/3 hours until 24 hours after last convulsion or abortion/birth, whichever was later; if convulsions recurred 30 minutes after LD, an additional 2 g IV/IM given and MD continued; therapy was considered failed if convulsions continued after 2 additional doses; no LD for women with imminent E N = 72 women and their babies  Higher dose regimen: ‘Pritchard’s regimen’: 4 g IV LD and 5 g IM as LD; 5 g IM/4 hours MD until 24 hours after last convulsion or abortion/birth, N = 54 women and their babies | Perinatal death, stillbirth, neonatal death | Funding: NR  Conflicts: none |
| Marret 2007; RCT | France  1997-2003 | N = 573 women and their babies  Inclusions: pregnant women with a singleton, twin or triplet very preterm fetus < 33 weeks GA if birth was expected or planned within 24 hours  Exclusions: women could not have received betamimetics, aminoglycosides or steroids for ≥1 hour; women with foetuses with severe malformations or chromosomal abnormalities, or if they met ≥ 1 of the following criteria: hypotension, cardiac rhythm abnormalities, hydroelectrolyte abnormalities, renal insufficiency, ingestion during last 24 hours of calcium channel blockers, digitalins or indomethacin, persistent signs of cardiovascular toxicity or tachycardia > 1 hour after cessation of tocolytic intake, myasthenia or indication for emergency caesarean; women with pregnancy-associated vascular disease (PE, FGR, haemolysis, elevated liver-function test results, low-platelet syndrome, retroplacental haematoma) | FN | MgSO4: 4 g IV LD over 30 minutes, N = 286 women and their babies analysed  Placebo: 40 mL infusion of isotonic saline over 30 minutes, N = 278 women and their babies analysed | Perinatal death, stillbirth, neonatal death, Apgar score < 7 at 5 minutes, intubated at delivery (tracheal intubation and/or epinephrine), RDS, MV (endotracheal ventilation), non-invasive ventilation, CLD (oxygen dependency at 36 weeks), apnoea and bradycardia, NEC, hypotension, seizures, IVH, PVL, any WMI, severe WMI, severe WMI or death | Funding: supported by the French Department of Health obtained in 1997 and a grant from Rouen University Hospital  Conflicts: NR |
| Mirzamoradi 2014; qRCT | Iran  2010-2012 | N = 92 women and their babies  Inclusions: pregnant women with GA < 34 weeks, hospitalised for PROM and labour complaints; the absence of concomitant disease such as chorioamnionitis or a history of drug sensitivity to MgSO4; no previous use of ; to ‘curb’ labour complain in a recent pregnancy; the absence of twin or multiple pregnancy  Exclusions: probable chorioamnionitis; progress of labour (4 cm cervical dilatation); allergy or medical complications in combination with MgSO4; fatal fetal anomalies; non-reassuring fetal status; severe FGR; severe PE/E; maternal haemorrhage with haemodynamic instability | T | MgSO4: 4 g IV LD over 20 minutes; 2 g/hour IV LD until 24 hours after complete cessation of uterine contractions, N = 46 women and their babies  Placebo, N = 46 women and their babies | Death > 28 days, before discharge (infant death), RDS, sepsis, IVH, NICU admission | Funding: NR  Conflicts: none |
| Mittendorf 2002; RCT | USA  1995-1997 | N = 57 women and their babies – in neuroprotective arm of the RCT  Inclusions: women in active preterm labour (cervical dilation > 4 cm), with or without PROM, at > 24 but < 34 completed weeks GA, with reassuring fetal assessment, and absence of clinical features that were suggestive initially of infection or PE  Exclusions: mothers with triplet or higher order gestations; women with PE | FN | MgSO4: 4 g IV bolus LD, N = 29 women and their babies  Placebo: saline control, N = 28 women and their babies | Perinatal death, stillbirth, neonatal death, IVH, IVH grade 3/4, PVL | Funding: the United Cerebral Palsy Research and Educational Foundation  Conflicts; NR |
| Moodley 1994; RCT | South Africa  Time period NR | N = 228 women and their babies  Inclusions: women with severe proteinuric hypertension (PE) (diastolic BP 110 mm Hg or greater, not settled with bed rest and sedation within 4-6 hours, with proteinuria of + or greater, detected by a semi-quantitative ‘dipstick’ method in either a clean-catch specimen or a catheter specimen of urine), or judged to have imminent E requiring delivery (severe hypertension and other symptoms such as severe persistent headache, nausea and omitting, visual disturbances, and epigastric pain or signs such as clonus and brisk reflexes) (including twins and singleton pregnancies)  Exclusions: prior anticonvulsant therapy or antihypertensive drugs | PE | MgSO4: ‘Pritchard’s regimen’: 4 g IV LD over 20 minutes and 10 g IM LD; 5 g/4 hours IM MD, with a maximum of 6 doses, n = 112 women and their babies  No treatment, N = 116 women and their babies | Perinatal death, stillbirth, neonatal death (early) | Funding: support from MRC of South Africa  Conflicts: NR |
| Mundle 2012; RCT | India  2008-2009 | N = 300 women and their babies  Inclusions: all pregnant women diagnosed with PE whose providers deemed would benefit from MgSO4 – systolic BP ≥ 140 mm Hg or a diastolic BP ≥ 100 mm Hg and proteinuria ≥ 1+; had not given birth or were 24 hours or less postpartum; exhibited urine output > 100 mL or more during the previous 4 hours or > 25 mL/hour; agreed to comply with the study procedures; 18 years or older.  Exclusions: women with E or seizing at the time of enrolment; received MgSO4 therapy 24 hours prior to study enrolment | PE | IM MD: ‘Pritchard’s regimen’: 4 g IV LD and 10 g IM LD; 5 g/4 hours IM MD continued for 24 hours and stopped when clinically indicated, N = 153 women and their babies  IV MD: ‘Zuspan’s regimen’: 4 g IV LD; 1 g/hour IV MD (using Springfusor pump) continued for 24 hours and stopped when clinically indicated, N = 147 women and their babies | Perinatal death | Funding: support from the John D. and Catherine T. Macarthur Foundation, and Go Medical, Subiaco, Australia (provided pump and tubing at reduced rate)  Conflicts: NR |
| Orji 2012; RCTAbstract | Africa  2011 | N = 170 women and their babies  Inclusions: severe PE  Exclusions: NR | PE | LD only: 4 g IV LD over 15 minutes, and 10 g IM MD, N = 85 women and their babies  LD and MD: ‘Pritchard’s regimen’: 4 g IV LD over 15 minutes and 10 g IM LD; 5 g IM/4 hours MD 24 hours, N = 85 women and their babies | Perinatal death | NR |
| Parashi 2017; RCT | Iran  Time period NR | N = 120 women and their babies  Inclusions: pregnant women with PROM at 34 weeks GA  Exclusions: hypertension, PE, trauma, gestational or aggravated DM, any type of metabolic disease affecting the pregnancy outcome, long-term drug use, gestational histories affecting the pregnancy outcomes | FN | MgSO4: 6 g IV LD over 20-30 minutes; 2 g/hour MD during 12 hours before labour,  N = 60 women and their babies  Placebo: “conventional treatment with normal saline infusion”, N = 60 women and their babies | Stillbirth, IVH, IVH grade 3/4H, intensive care unit stay (mean ± SD) (days) | NR |
| Pascoal 2019; RCT | Brazil  2015-2016 | N = 62 women and their babies  Inclusions: women with severe PE (defined by ACOG criteria), prescribed MgSO4  Exclusions: E prior to initial MgSO4 LD, use of other medicines or illicit drugs that could interfere with maternal haemodynamics, contraindications to MgSO4 (known hypersensitivity to the drug, oliguria with urinary output below 25 mL per hour, or severe myasthenia), acute or chronic kidney disease and a diminished level of consciousness | PE | Lower dose regimen: 6 g IV LD over 20 minutes, 1 g/hour IV MD, N = 31 women and their babies  Higher dose regimen: 6 g IV LD over 30 minutes; 2 g/hour IV MD, N = 31 women and their babies | Neonatal death, need for resuscitation, ‘respiratory disorders’, MV, NICU admission | Funding: supported by IMIP  Conflicts: none |
| Rimal 2017; RCT | Nepal  2014-2015 | N = 60 women and their babies  Inclusions: diagnosed cases of severe PE who were admitted to the ward, gave written informed consent, and were in labour or planned for termination of pregnancy  Exclusions: women < 20 GA, and who had received MgSO4 before admission to the ward | PE | LD only: 4 g IV LD over 5 minutes and 10 g IM LD, N = 30 women and their babies  LD and MD: ‘Pritchard’s regimen’: 4 g IV LD over 5 minutes and 10 g IM LD; MD every 3 hours IM for 24 hours from time of birth or last convulsion, N = 30 women and their babies  All women: for recurrent convulsions, 2 g IV given over 5 minutes; MD continued until 24 hours after birth/last dose | Perinatal death, stillbirth, neonatal death, Apgar score < 7 at 0 minutes, Apgar score < 7 at 1 minute, Apgar score < 7 at 5 minutes, NICU admission | Funding: NR  Conflicts: none |
| Rouse 2008; RCT | USA  1997-2004 | N = 2241 women and their babies  Inclusions: women carrying singletons or twins at 24 through 31 weeks GA, at high risk for spontaneous delivery because of ROM at 22-31 weeks GA, or because of advanced preterm labour with dilatation of 4-8 cm and intact membranes; or if an indicated preterm delivery was anticipated within 2-24 hours (e.g. because of FGR)  Exclusions: women where delivery was anticipated within < 2 hours or if cervical dilatation > 8 cm; with ROM < 22 weeks, with unwillingness of the obstetrician to intervene for the benefit of the fetus, major fetal anomalies or death, maternal hypertension or PE, maternal contraindications to MgSO4 (e.g. severe pulmonary disorders), and receipt of IV MgSO4 within the previous 12 hours | FN | MgSO4: 6 g IV LD over 20-30 minutes; 2 g/hour IV MD until birth or 12 hours, N = 1096 women and their babies  Placebo: identical appearing placebo as above, N = 1145 women and their babies  All women: if delivery had not occurred after 12 hours, the infusion was discontinued and resumed when delivery was imminent; if at least 6 hours had passed another LD was given | Perinatal death (fetal or infant death < 1 year), Apgar score < 7 at 5 minutes, intubated at delivery, resuscitation in delivery room (oxygen bag, mask or both; any; chest compressions), RDS, MV, CLD (BPD), NEC, sepsis (culture-proven), hypotension (treated with vasopressors), PDA, ROP, generalised hypotonicity, seizures, IVH, IVH grade 3/4, PVL  Hirtz 2015 (presents data for all children, term ultrasounds, N = 1776 babies; children born < 32 weeks GA, most severe findings, N = 1613 babies): IVH, IVH grade 3/4, PVL, ventriculomegaly, echodensity, echolucency, any of above  Horton 2015 (presents data for singleton children born to mothers with PPROM only, N = 1259 babies): death (to hospital discharge), Apgar score < 7 at 5 minutes, RDS, NEC, sepsis (culture-proven), ROP, IVH grade 3/4, PVL, composite (any of RDS, NEC, sepsis, ROP, IVH grade 3/4, PVL, death)  Vilchez 2018 (presents data for women with singleton pregnancies, with no documented congenital anomalies, that received MgSO4 or placebo, N = 1894 babies; and reports subgroup analyses based on race/ethnicity: African-America, N = 852; Caucasian, N = 686; Hispanic, N = 338; Asian, N = 18): stillbirth/death, low Apgar score, resuscitation, assisted ventilation, RD, TTN, surfactant use, NEC, ROP, NICU admission, composite outcome (≥ 1 complication)  Vilchez 2018b (presents data for 2096 children born to women with BMI data; and reports subgroup analyses based on BMI): perinatal death (assumed to be as per Rouse 2008) | Funding; support from NICHD and the National Institute of Neurological Disorders and Stroke  Conflicts: none |
| Saha 2017; RCT | India  Time period NR | N = 41 women and their babies  Inclusions: patients with antepartum and intrapartum E; E was diagnosed if a patient with PE or hypertension experienced convulsions after 20 weeks GA  Exclusions: patients with E who experienced complications such as coma, pulmonary oedema, oliguria, ICH, who had already received MgSO4, phenytoin and diazepam before attending the hospital | E | IM MD: ‘Zuspan’s regimen’: 4 g IV LD; 1 g/hour IV MD for 24 hours after last convulsion or birth, N = 20 women and their babies  IV MD: ‘Dhaka regimen’: 4 g IV LD and 6 g IM LD; 2.5 g/4 hour IM MD for 24 hours after last convulsion or birth, N = 21 women and their babies  All women: in cases of convulsion recurrence, 2 g IV administered | Perinatal death, stillbirth, neonatal death, Apgar score < 7 at 1 minute, Apgar score < 7 at 5 minutes, RD, jaundice, hypotonia, NICU admission | Funding: Department of Science and Technology (DST Government Organisation), Chandigarh Administration, India  Conflicts: none |
| Shilva 2007; RCTBrief communication | India  Time period NR | N = 50 women and their babies  Inclusions: women with antepartum E  Exclusion: renal failure, pulmonary oedema, having received MgSO4 before coming into the hospital | E | Lower dose regimen: ‘Dhaka regimen’, N = 25 women and their babies  Higher dose regimen, N = 25 women and their babies  All women: in cases of convulsion recurrence, 2 g IV administered | Perinatal death, stillbirth, neonatal death, Apgar score < 7 at 1 minute, Apgar score < 7 at 5 minutes, RD, respiratory depression, jaundice, hypotension, hypotonia, requirement for calcium gluconate, NICU admission | NR |
| Shreya 2014; qRCT | India  2010-2012 | N = 80 women and their babies  Inclusions: all pregnant women with imminent E having one of the following: persistent headache, visual disturbances, epigastric pain; or pregnant women with E: convulsions in a pregnant woman that cannot be attributed to other causes  Exclusions: not willing to participate; women receiving any anticonvulsant or MgSO4 prior to arrival at the hospital; contraindications for MgSO4 such as respiratory depression, renal failure, hypersensitivity, heart block, Addison’s disease, severe hepatitis, myocardial damage, myasthenia gravis | PE/E | LD only: 4 g IV LD over not less than 3 minutes, and 4 g IM LD, N = 40 women and their babies  LD and MD: ‘Pritchard’s regimen’: 4 g IV LD over not less than 3 minutes, followed by 10 g IM LD; 5 g IM/4 hours MD, for 24 hours following convulsion or birth, N = 40 women and their babies  All women: 2 g IV over 2 minutes for convulsion recurrence after 30 minutes | NICU admission for RD, NICU admission for early onset sepsis, NICU admission for late onset sepsis, NICU admission for meconium aspiration syndrome, NICU admission for birth asphyxia, NICU admission | NR |
| Singh 2011; RCT | India  2 year period | N = 158 women and their babies  Inclusions: patients presenting with E  Exclusions: women diagnosed with other causes of convulsions in pregnancy like cerebral malaria and epilepsy | E | IM: ‘Pritchard’s regimen’: 4 g IV LD over 3-5 minutes and 10 g IM LD; 5 g/4 hours IM until 24 hours after delivery or last convulsion, N = 60 women and their babies  IV: ‘Zuspan’s regimen’:  4 g IV LD over 5-10 minutes; 1 g/hour IV MD until 24 hours after delivery or last convulsion, N = 49 women and their babies  IV: ‘Sibai’s regimen’:  6 g IV LD over 15-20 minutes; 2 g/hour IV until 24 hours after delivery or last convulsion, N = 49 women and their babies  All women: if convulsions persisted 15 minutes post LD, 2 g IV given | Perinatal death, stillbirth, neonatal death, NICU admission | NR |
| Tangmanowutthikul 2019 | Thailand  2018 | N = 86 women and their babies  Inclusions: women with PE with severe features (according to ACOG guideline), admitted with GA ≥ 24 weeks  Exclusions: serum creatinine > 1.3 mg/dL, hypersensitivity to MgSO4, myocardial damage, diabetic coma, heart block and myasthenia gravis. | PE | Lower dose regimen: Weight-adjusted protocol: 4 g IV LD; IV MD: 1.2 g/hour for < 60 kg, 1.3 g/hour for 60-79.9 kg, 1.4 g/hour for 80-99.9 kg and 1.5 g/hour for ≥ 100 kg; continued until 24 hours after birth, N = 43 women and their babies  Higher dose regimen: 4 g IV LD; 2 g/hour IV MD, N = 43 women and their babies  Serum concentrations monitored at 2 and 4 hours, adjusted and continued until 24 hours after birth | Perinatal death, stillbirth, neonatal death, NICU admission | Funding: support from Dr. Thammanoon Wisittanawat, director of Udonthani Hospital  Conflicts: None |
| Terrone 2000; RCT | USA  1997-1998 | N = 160 women and their babies  Inclusions: singleton or twin gestation between 24-34 weeks GA, spontaneous preterm labour (advancement seen on cervical examination with uterine contractions while the patient was admitted to the triage unit or dilatation of 2 cm and effacement of 80% with ≥ 6 uterine contractions/hour), and ability to provide informed consent  Exclusions: higher-order multiple gestations, ROM, non-reassuring fetal assessment, evidence of intrauterine infection, treatment with any tocolytic agent before maternal transport, and inability or refusal to provide informed consent, inability to tolerate high doses of MgSO4 (e.g. renal failure) | T | Lower dose regimen: 4 g IV LD over 20 minutes; 2 g/hour IV MD, N = 78 women and their babies  Higher dose regimen: 4 g IV LD over 20 minutes; 5 g/hour IV MD, N = 82 women and their babies  All women: if after 1^st^ hour contractions/cervical dilation or effacement continued MD increased by 1 g/hour until successful T or treatment failure | Apgar score < 7 at 1 minute, Apgar score < 7 at 5 minutes | Funding: supported by Vicksburg Hospital Medical Foundation  Conflicts: NR |
| Witlin 1997; RCT | USA  1995-1996 | N = 135 women and their babies  Inclusions: women with a GA of at least 37 weeks, with recent-onset hypertension (systolic BP ≥ 140 mm Hg or diastolic BP ≥ 90 mm Hg) and proteinuria (≥ 300 mg/24 hours)  Exclusions: women meeting criteria for severe PE; fetal malpresentation; congenital anomalies; non-reassuring fetal testing; contraindications to the use of MgSO4; contraindications to a trial of labour | PE | MgSO4: 6 g IV LD over 15-20 minutes; 2 g/hour IV MD, continued until 12 hours postpartum, N = 67 women and their babies  Placebo: saline infusion identical in appearance, N = 68 women and their babies | Apgar score ≤ 6 at 1 minute, Apgar score ≤ 6 at 5 minutes | NR |

Abbreviations: ACOG: American College of Obstetrics and Gynecologists; BP: blood pressure; BPD: bronchopulmonary dysplasia; CLD: chronic lung disease; CP: cerebral palsy; dL: decilitre; DM: diabetes mellitus; DST: Defence Science and Technology; E: eclampsia; g: grams; FGR: fetal growth restriction; GA: gestational age; HMD: hyaline membrane disease; ICH: intracranial haemorrhage; IM: intramuscular; IMIP: Instituto de Medicina Integral Prof. Fernando Figueira; IQR: interquartile range; IUD: intrauterine death; IUGR: intrauterine growth restriction; IV: intravenous; IVH: intraventricular haemorrhage; kg: kilograms; LD: loading dose; MD: maintenance dose; mg: milligrams; MgSO4: magnesium sulphate; MRC: Medical Research Council; MV: mechanical ventilation; N: number; NEC: necrotising enterocolitis; NHMRC: National Health and Medical Research Council; NICHD: Eunice Kennedy Shriver National Institute of Child Health and Human Development; NICU: neonatal intensive care unit; NIH: National Institutes of Health; NINDS: National Institute of Neurological Disorders and Stroke; NR: not reported; PDA: patent ductus arteriosus; PE; pre-eclampsia; PI: primary investigator; PIH: pregnancy-induced hypertension; PPROM: preterm premature rupture of membranes; PROM: premature rupture of membranes; PVL: periventricular leucomalacia; qRCT: quasi-randomised controlled trial; RCT: randomised controlled trial; RD: respiratory distress; RDS: respiratory distress syndrome; ROM: rupture of membranes; RR: respiratory rate; RVO: right ventricular output; SCBU: special care baby unit; SD: standard deviation; sGOT: serum glutamic-oxaloacetic transaminase; SVC: superior vena cava; TTN: transient tachypnoea of the newborn; UK: United Kingdom; UNDP/UNFPA/WHO: United National Development Programme/United National Population Fund/Word Health Organization; USA: United States of America; WMI: white matter injury

**Characteristics of included studies – non-randomised studies**

| **Study; design** | **Setting** | **Participants** | **MgSO4 indication** | **Relevant comparison groups** | **Outcome measure(s)** | **Funding and conflicts** |
| --- | --- | --- | --- | --- | --- | --- |
| Adama-Hondegla 2013; unclear: results appear to be presented as RCS with CCS(N) | Togo  2007-2009 | N = 170 women, 178 babies  Inclusions: (singleton/twin) newborns > 28 weeks GA from women diagnosed with E before birth (definition provided)  Exclusions: NR | E | 1: Babies still living at the 7^th^ day of life, N = 147 babies  2: Stillbirths and neonatal deaths in the 1^st^ 7 days, N = 31 babies | MgSO4 exposure (10 g in 500 mL, IV 20 drops/minute) | NR |
| Alexander 2006; PCS | USA  2000-2004 | N = from 72004 births, 87 women with E and their babies included in analyses  Inclusions: women with and without GH (detailed definition provided)  Exclusions: NR | GH/PE | 1: No GH and no MgSO4 with E, N = 49 women and their babies  2: GH and IV MgSO4 with E, N = 11 and their babies  3: GH and no MgSO4 with E, N = 27 and their babies | Adverse outcome composite, perinatal death | NR |
| Alston 2016; NCCS | USA  2004-2009 | N = 169 babies  Inclusions: neonates born from singleton pregnancies between 24-34 weeks GA  Exclusions: birth before 24 weeks GA or after 34 weeks GA, indication for preterm birth was something other than spontaneous preterm labour, or the infant chart could not be linked to the maternal chart | T | 1: Period of MgSO4 use for T (2004-2006) (6 g IV LD; 2 g/hour IV MD), N = 102 (90 babies in analyses)  2: Period of no MgSO4 use for T (2007-2009), N = 67 (64 babies in analyses) | Hospital stay (days) (mean, no measure of variance), neonatal death, RDS, BPD, sepsis, NEC, IVH | NR |
| Ambadkar 2017; PCS | Authors from India  1 year period | N = 120 women and babies  Inclusions: women admitted to labour ward ≥ 34 weeks GA with PE/E  Exclusions: women who received different dosages of MgSO4, who required discontinuation of MgSO4 due to toxicity, who had chronic medical disorders or anomalous fetuses, APH, or any other co-existing major obstetric problem | PE/E | 1: MgSO4 (Pritchard’s regimen: 4 g IV over 10 minutes and 5 g IM LD; 5 g/4 hours IM MD), N = 60 babies  2: Matched patients with PE not requiring MgSO4 or any other anticonvulsant, N = 60 babies  1: MgSO4 and NICU admission, yes, N = 13 babies  2: MgSO4 and no NICU admission, N = 47 babies | Neonatal death, NICU admission, hypotonia, RD, meconium passage (< 6, 6-12, > 12 hours)  NICU admission: MgSO4 dose (categories: LD, LD + 1, LD + 2, LD + 3, LD + 4, LD + 5, LD + 7 doses), duration of MgSO4 (< 6, 6-12, 12-18, ≥ 18 hours), time elapsed since last MgSO4 dose and birth (1-2, 2-3, 3-4, 4-5, > 5 hours) | Funding: NR Conflicts: none |
| Bajaj 2018; RCS | USA  2012-2013 | N = 7014 babies  Inclusions: babies born at 29+0 to 33+6 weeks GA who were enrolled in the NRN MPT registry  Exclusions: babies in whom a prenatal diagnosis caused a decision to withdraw or limit intensive care | NR | 1: Routine care without resuscitation, N = 1684 babies  2: Oxygen or CPAP but not ventilation, intubation or CPR, N = 2279 babies  3: Bag and mask ventilation but not intubation or CPR, N = 1831 babies  4: ETT intubation but not CPR, N = 1034 babies  5: CPR, N = 186 babies | MgSO4 exposure | Funding: supported by NIH, NICHD, NCATS  Conflicts: none |
| Basu 2012; RCS | USA  2006-2010 | N = 475 babies  Inclusions: preterm neonates born between 24-32 weeks GA admitted to the NICU  Exclusions: neonates with major congenital malformations or chromosomal anomalies, and those born to women who received MgSO4 for PE/E | FN | 1: MgSO4 (6 g IV LD over 30 minutes; 2 g/hour IV MD until birth), N = 289 babies  2: No MgSO4, N = 186 babies | Survival without IVH/PVL, resuscitation, intubation, BPD, IVH/PVL. ROP, PDA, LOS (days) (mean ± SD), death | NR |
| Belden 2017; unclear: results appear to be presented as RCS with CCS(N) | USA  2012-2013 | N = 83 babies born to 72 women  Inclusions: neonates born at 24 weeks GA or greater who were admitted to the NICU and whose mothers received MgSO4 infusions prior to birth  Exclusions: neonates with independent factors that could lead to feeding intolerance, including congenital abnormalities, such as gastroschisis and neonatal abstinence syndrome | FN/PE | 1: Enteral feeding intolerance, N = 49 babies  2: No feeding intolerance, N = 34 babies  1: MgSO4 > 80 g, N = NR  2: MgSO4 ≤ 80 g, N = NR | MgSO4 dose (g) (mean ± SD)  Enteral feeding intolerance, parenteral nutrition (days) (measure NR) | Funding: NR Conflicts: none |
| Bertello Grecco 2019; PCS  Abstract | Authors from Argentina  Time period NR | N = 93 women and their babies  Inclusions: women > 18 years with PE  Exclusions: NR | PE | 1: MgSO4 IV ≤ 24 hours, N = 51 women and their babies  2: MgSO4 IV > 24 hours, N = 42 women and their babies | Neonatal death, respiratory depression, hypotonia, NICU admission | NR |
| Black 2006; PCS | Authors from USA  Time period NR | N = 134 babies  Inclusions: preterm infants who received neonatal care, < 35 weeks GA at birth and considered high-risk for developmental and health problems because they either weighed < 1,500 g at birth or required MV  Exclusions: infants with congenital diagnoses associated with developmental problems (such as Down syndrome, congenital hydrocephalus, or microcephaly) or symptomatic from substance exposure; infants with family situations such that obtaining consent would be impossible or intrusive | PE/PIH/HELLP/T | 1: MgSO4 with (N = 45)/without (N = 5) steroids, N = 50 babies  2: No MgSO4 with (N = 38)/without steroids (N = 46), N = 84 babies | Ventilation (days) (mean ± SD), methylxanthines (days) (mean ± SD), NBRS (mean ± SD), IVH | Funding: NIH NINR  Conflicts: none |
| Blackwell 2002; PCS | Authors from USA  Time period NR | N = 39 babies  Inclusions: term women (≥ 37 weeks GA) with PE (mild or severe) who received MgSO4, and controls who did not receive MgSO4  Exclusions: FGR (birthweight < 10th centile for GA), multiple gestations, fetal structural anomalies, clinical chorioamnionitis or prior exposure to MgSO4 | PE | 1: MgSO4 (6 g IV LD over 20-30 minutes; 2 g/hour IV MD until birth), N = 13 babies  2: No MgSO4, N = 26 babies | Troponin I ≥ 1.0 ng/mL | NR |
| Bonta 2000; PCS  Abstract | USA  1995-1999 | N = 379 women and babies  Inclusions: women requiring IV MgSO4 who delivered while receiving treatment  Exclusions: NR | T | 1: MgSO4 IV < 72 hours, N = 199 babies  2: MgSO4 IV > 72 hours, N = 45 babies  3: No MgSO4, N = 135 babies | HsPDA treated with indomethacin | NR |
| Bozhurt 2016; RCS | Turkey  2010-2012 | N = 387 babies  Inclusions: preterm infants < 32 weeks GA born to women with PE, who survived to 2 years  Exclusions: NR | PE | 1: MgSO4 (6 g IV LD over 20 minutes; 2 g/hour IV MD for 24 hours), N = 59 babies  2: No MgSO4, N = 328 babies | RDS, BPD, hypoglycaemia, apnoea, PDA, IVH grade 3/4 and PVL, PVL only, culture proven sepsis, NEC grade ≥ II, ROP grade > 3 | Funding: NR Conflicts: none |
| Boyle 2018; RCS  Abstract | USA  2012-2015 | N = 285 women and babies  Inclusions: women who delivered non-anomalous singleton fetuses at term by primary caesarean with a category 2 or 3 FHR tracing as an indication for birth  Exclusions: NR | NR | 1: MgSO4, N = 16 babies  2: No MgSO4, N = 271 babies  [note discrepancy between total N and group Ns reported] | Composite adverse neonatal outcome (defined as Apgar score < 7 at 5 minutes, arterial cord pH < 7.1 and/or base deficit ≥ 12, admission to the NICU, need for immediate neonatal resuscitation beyond bulb suction and stimulation, or hospitalisation ≥ 3 days) | NR |
| Brazy 1982; RCS | USA  1979 | N = 56 babies  Inclusions: infants born before 36^th^ week GA to women with early and severe hypertension in pregnancy (resting diastolic BP ≥ 110 mmHg and proteinuria); for each infant of mother with hypertension, a control infant (born to a normotensive mother) was selected on the basis of GA and birth order  Exclusions: no major congenital abnormalities, major disease (IDD, sickle cell anaemia, renal failure, heart disease), no known exposure to insulin or tocolytic agents; for control infants: no known exposure to sedatives, diuretics or drugs with antihypertensive properties | ESHP | 1: Hypertensive women treated with IV MgSO4, N = 28 babies  2: Non-hypertensive women, with no MgSO4, N = 28 babies | Days hospitalised (mean ± SD), thrombocytopenia, leukopenia, neutropenia, DIC, severe respiratory disease, TTN, delayed adaptation, PDA, hypotension, delayed stooling (> 24 hours), ileus, hypotonia, other disease (CNS haemorrhage, air block, acute renal failure, NEC), neonatal death, death after 28 days of age, before hospital discharge, stillbirth | NR |
| Brookfield 2015; unclear: results appear to be presented PCS with CCS(N)  Abstract | Authors from USA  2012-2014 | N = 55 women and babies  Inclusions: pregnant women prescribed MgSO4 for FN/PE < 32 weeks GA (4 g LD; 2 g/hour MD)  Exclusions: NR | FN/PE | 1: Resuscitation (oxygen, bag/mask, intubation, chest compressions), N = 27 babies  2: No resuscitation, N = 28 babies | MgSO4 dose (g) (mean ± SD) | NR |
| Brookfield 2016; RCS (secondary analysis of RCT)  Abstract | NR (see Rouse 2008) | N = 1496 women and babies  Inclusions: women who had received 1 course of antenatal steroids and had PPROM at < 32 weeks  Exclusions: NR | FN | 1: MgSO4, N = 735 babies  2: No MgSO4, N = 761 babies | RDS, ventilation | NR |
| Brown 2019; unclear, appears to be presented as CCS  Abstract | Canada  2002-2016 | N = 218 babies  Inclusions: babies born < 31 weeks GA diagnosed with SH (SBP > 100 mmHg requiring treatment, before discharge from NICU, or on 3 consecutive occasions during outpatient follow-up), and controls matched for GA, sex and birthweight  Exclusions: babies who died < 7 days of age | NR | 1: SH, N = 109 babies  2: No SH, N = babies | MgSO4 exposure | NR |
| Canterino 1999; RCS | USA  1990-1996 | N = 918 babies  Inclusions: inborn neonates with GA from 23-34 weeks and birthweights from 500-1750 g  Exclusions: major congenital anomalies, neonatal death by day 3, transfers before day 3 | PE/T | 1: MgSO4, N = 398 babies  2: No MgSO4, N = 520 babies  1: Abnormal cranial sonograms, N = 39 babies  2: Normal findings, N = 125 babies  1: Severe lesions, N = 27 babies  2: Normal findings, N = 127 babies | Apgar score < 7 at 5 minutes, RD, neonatal death, abnormal sonograms (any PVL or IVH), severe lesions (any PVL, PVL with IVH, or IVH grade 3/4) | NR |
| Cawyer 2019; RCS  Abstract | USA  2008-2011 | N = 2468 women and their babies  Inclusions: women with PE without severe features at any time in pregnancy, who delivered > 32 weeks GA  Exclusions: NR | PE | 1: MgSO4 at any time during delivery hospitalisation, N = 1353 babies  2: No MgSO4, N = 1115 babies | Perinatal or neonatal death, NICU admission | NR |
| Cho 2014; RCS  Abstract | NR | N = 570 women and babies  Inclusions: pregnant women with preterm birth and their paired neonates  Exclusions: NR | NR | 1: MgSO4 IV, N = 101 babies  2: No MgSO4, N =469 babies | Hypocalcaemia | NR |
| Chowdhury 2009; unclear: PCS (or NRT) | India  2001-2015 | N = 630 women (529 babies born to antepartum/intrapartum cases)  Inclusions: consecutive women with clinical diagnosis of E regardless of when/where the convulsions occurred, whether pregnancy was single/multiple, and whether baby had been delivered  Exclusions: women with convulsions due to epilepsy or other causes | E | 1: MgSO4 by Pritchard’s regimen (8 g IV over 2-3 minutes and 5 g IM in each buttock LD; 5 g/4 hours IM MD for at least 24 hours after birth/last convulsion), N = 480 women (406 babies born to antepartum/intrapartum cases)  2: MgSO4 by low-dose IV regimen (4 g IV LD over 2-3 minutes; 0.6 g/hour IV MD for at least 24 hours after birth/last convulsion), N = 150 women (123 babies born to antepartum/intrapartum cases) | Stillbirth, early neonatal death due to birth asphyxia and prematurity (1^st^ 7 days), perinatal death | NR |
| Chun 2014; RCS  English abstract; article in Korean | Authors from Korea  2003-2013 | N = 209 women and babies  Inclusions: women who delivered vaginally with the diagnosis of PE  Exclusions: CD | PE | 1: MgSO4, N = 119 babies  2: No MgSO4, N = 90 babies | Apgar score < 7 at 1 minute, Apgar score < 7 at 5 minutes, NICU admission | CD |
| Cuff 2018; RCS | Authors from USA  2014-2015 | N = 224 women and their babies: 44 women and 54 babies exposed to MgSO4 within 12 hours of birth  Inclusions: women who had a preterm birth < 32 weeks GA and had received MgSO4 prior to birth  Exclusions: women with PE, known fetal anomalies, and/or stillbirth | FN | 1: 2014 (dosing according to BEAM trial: 6 g IV LD; 2 g/hour IV MD for 12 hours), N = 18 babies exposed within 12 hours of birth  2: 2015 (dosing according to PREMAG trial: 4 g IV MD; no MD), N = 36 babies exposed within 12 hours of birth | Apgar score < 7 at 5 minutes, ROP grade 3/4, IVH grade 3/4 | Funding: NR  Conflicts: none |
| Das 2015; PCS | India  2011-2012 | N = 100 women and their babies  Inclusions: women with antepartum or intrapartum E (definition provided) > 20 weeks GA, presenting in obstetric, labour and/or wards, who have informed consent  Exclusions: women with complications such as renal failure, severe pulmonary oedema with respiratory failure, cerebrovascular accident, and DIC, who received MgSO4 before coming to hospital, with known seizure disorders, with multiple pregnancies, with infants with congenital malformations or birthweights < 1000 g | E | 1: 8 g MgSO4, N = 20 babies  2: > 8 g MgSO4, N = 80 babies  MgSO4: low-dose regimen: 3 g IV over 15 minutes and 2.5 g IM in each buttock LD; 2.5 g/4 hours IM in alternate buttocks MD | Apgar score < 7 at 1 minute, Apgar score < 7 at 5 minutes, Apgar score ≤ 3 at 1 minute, Apgar score ≤ 3 at 5 minutes, respiratory depression, intubation in delivery room, bradycardia, hypotonia, hyporeflexia, NICU admission, significant respiratory support in NICU, time to 1^st^ stool > 24 hours, time to 1^st^ void > 48 hours, number of episodes of feeding intolerance ≥ 3, stillbirth, neonatal death due to complications of hypermagnesemia | Funding: Burdwan Medical College  Conflicts: none |
| Deering 2005; RCS | USA  12 month period | N = 221 babies  Inclusions: all preterm admissions  Exclusions: congenital anomalies, admission > 24 hours of life, hydrops fetalis, no SNAP score calculated, transport back to the referring hospital before all information could be obtained | PE/T | 1: MgSO4, N = 103 babies  2: No MgSO4, N = 118 babies | SNAP score (mean ± SD), SNAP score > 10 | Funding: no funding  Conflicts: NR |
| De Jesus 2015; RCS | USA  2011-2012 | N = 1544 babies  Inclusions: infants born between 23+0-28+6 weeks GA and enrolled in the generic database  Exclusions: NR | FN/PIH/T | 1: MgSO4, N = 1091 babies  2: No MgSO4, N = 453 babies | Delivery room resuscitation (PPV via bag and mask, any CPAP devices, intubation, chest compression and epinephrine), delivery room intubation, day 1 MV, day 1 ET MV, day 3 MV, day 3 ET MV, hypotension, PDA treated (medical or surgical), RDS, pulmonary haemorrhage, traditional BPD, late onset sepsis/meningitis, NEC stage II or greater, ROP any stage, IVH or parenchymal haemorrhage, cPVL, death, cumulative days on MV (median, Q1, Q3), cumulative days on oxygen support (median, Q1, Q3), LOS (median, Q1, Q3) | Funding: NIH and NICHD  Conflicts: none |
| del Moral 2007; RCS | USA  1995-2004 | N = 954 babies included, 941 babies analysed  Inclusions: neonates with birthweights between 500-1000 g admitted to NICU  Exclusions: NR | PE/T | 1: MgSO4 (4-6 g IV bolus LD; 2 g/hour IV MD), N = 546 babies  2: No MgSO4, N = 395 babies | PDA, death, IVH grade 3/4, PVL | Funding: Supported by University of Miami, Project Newborn  Conflicts: NR |
| delValle 1998; unclear: PCS  Abstract | NR (“single regional perinatal center”)  1994 | N = 110 babies  Inclusions: all VLBW  Exclusions: NR | NR | 1: MgSO4, N = 34 babies  2. No MgSO4, N = 76 babies | Surfactant treatment, indomethacin treatment, PDA, NEC, IVH, PVL | NR |
| Derks 2016; NCCS  Abstract | Netherlands  2011-2015 | N = 207 babies  Inclusions: extremely preterm infants born 24-28 weeks GA  Exclusions: NR | FN | 1: 2 years post MgSO4 implementation, N = 99 babies  2: 2 years pre MgSO4 implementation, N = 108 babies | PWML at 30 and 40 weeks MRI, early intubation for respiratory insufficiency, hypotension | NR |
| De Silva 2018; RCS (within report of ITS) | Canada  2011-2015 | N = 14108 babies  Inclusions: babies born at 24+0-31+6 weeks GA and collected information about use of MgSO4 for FN as well as pregnancy characteristics  Exclusions: babies with congenital anomalies were excluded, as in prior CNN analyses | FN | 1: MgSO4 for FN, N = 5314 babies  2: No MgSO4, N = 7238 babies  3: MgSO4 for another indication, N = 1556 babies | Intensive resuscitation (either chest compressions or intubation and ventilation or epinephrine administration in the delivery room) | Funding: CIHR  Conflicts: none |
| de Veciana 1995; RCS | USA  1985-1991 | N = 73 women, 80 babies  Inclusions: women with diagnosis of advanced preterm labour (occurrence of painful, palpable uterine contractions ≥ 2 times in 10 minutes for > 1 hour); cervical dilation ≥ 3.5 cm on admission; estimated GA < 36 weeks; available information of labour course and pregnancy outcome  Exclusions: documented ROM, amniocentesis documenting pulmonary maturity; history consistent with incompetent cervix ± cerclage in place; clinical evidence of amnionitis; vaginal bleeding on admission | T | 1: MgSO4 IV (4-6 g IV LD; 2-4 g/hour IV MD for at least 48 hours and no longer than 7 days), N = 44 women, 48 babies  2: No MgSO4 tocolysis, N = 29 women, 32 babies | Days in hospital (mean ± SD), days intubated (mean ± SD), RDS (mild to severe), RDS severe, IVH grade I-IV, NEC, neonatal death, Apgar score < 7 at 5 minutes | NR |
| Downey 2017; RCS | USA  2007-2013 | N = 28035 babies  Inclusions: ELBW (≤ 1000 g) infants discharged during study period  Exclusions: outborn infants, and infants with severe congenital anomalies | FN/PE/T | 1: MgSO4, N = 11789  1: No MgSO4, N = 16246 | SIP, neonatal death in 1^st^ 21 days of life, surgical NEC, medical NEC, death, NEC or SIP, IVH grade 3/4 | Funding: some authors receive support from NIH, USA government, NICHD, NHLBI, FDA, Cempra Pharmaceuticals, and ‘industry’; others report no conflicts. Sponsors had no involvement in design, collection, analysis, interpretation, writing and submission. |
| Drassinower 2015; RCS (secondary analysis of RCT)  Abstract | NR (see Rouse 2008) | N = 1047 women and babies  Inclusions: non-anomalous, singleton pregnancies receiving MgSO4 or placebo, exposed for > 3 hours at time of birth  Exclusions: women exposed for < 3 hours or if drug was discontinued prior to birth | FN | 1: MgSO4, N = 461 babies  2: Placebo, N = 586 babies | Composite of immediate outcomes (Apgar score < 7 at 5 minutes, oxygen administration in delivery room, intubation, chest compressions, hypotension, hypotonicity), Apgar score < 7 at 5 minutes, oxygen bag, mask or both, intubation, chest compressions, hypotension treated with vasopressors, generalised hypotonicity, RDS, MV, seizures, IVH, death | NR |
| Duffy 2012; RCS | USA  4 year period | N = 5387 women and babies  Inclusions: women who reached the second stage of labour, born ≥ 37 weeks GA, and had ≥ 10 minutes of EFM tracing in 30 minutes before birth and an available umbilical arterial cord blood gas, with singleton pregnancies, cephalic presentation and no known fetal anomalies  Exclusions: women who delivered by caesarean before labour or before complete dilatation | Severe PE | 1: MgSO4 (6 g IV LD; 2 g/hour IV MD), N = 248 babies  2: No MgSO4, N = 5139 babies | Composite adverse outcome (fetal acidemia, base excess ≤ -12.00, SCBU or NICU admission) | Funding: 1 author supported by Robert Wood Johnson Foundation  Conflicts: none |
| Edwards 2018; RCS (secondary analysis of RCT), also presents CC(N) analysis | USA  1997-2004 | N = 1944 women and babies  Inclusions: women considered at high risk of preterm birth between 24-31 weeks GA (risk based on presentation with ROM between 22-31 weeks GA, spontaneous labour with cervical dilation of 4-8 cm, or providers anticipated an indicated preterm birth within 2-24 hours)  Exclusions: multiple gestation, chromosomal abnormalities, stillbirth, congenital anomalies, or missing information regarding the presence or absence of clinical chorioamnionitis | FN | Chorioamnionitis, N = 228 mother and babies  1: MgSO4, N = 109 babies  2: No MgSO4, N = 119 babies  No chorioamnionitis, N = 1716 women and babies  1: MgSO4, N = 839 babies  2: No MgSO4, N = 877 babies  MgSO4: 6 g IV LD over 20-30 minutes; 2 g/hour IV MD until birth or 12 hours; re-treatment permitted | MgSO4 exposure  IVH, NEC, BPD | Funding: NR  Conflicts: none |
| Elimian 2002; RCS | USA  1998-2001 | N = 401 babies  Inclusions: premature neonates born between 23-34 weeks GA following preterm labour with intact membranes or PPROM  Exclusions: neonates exposed to MgSO4 for seizure prophylaxis | T | 1: MgSO4 (4 g IV LD; 2-3 g/hour IV MD), N = 190 babies  2: No to MgSO4 with/without other tocolytics, N = 211 babies  1: MgSO4 for > 24 hours, N = 79 babies  2. MgSO4 for ≤ 24 hours, N = 111 babies | Apgar score < 7 at 5 minutes, RDS, surfactant, antibiotics, PDA, IVH/PVL, NEC, sepsis, neonatal death (1^st^ 28 days) | NR |
| Elliot 2003; RCS  Abstract | Authors from USA  1997-2003 | N = 14092 babies  N = 9782 babies in groups of interest (excluded terbutaline and combination T groups)  Inclusions: 24-32 weeks GA at birth, no major congenital anomalies, no PE, birthweight > 400 g  Exclusions: NR | T | 1: MgSO4, N = 6186 babies  2: No MgSO4, N = 3596 babies | Neonatal death, IVH, NEC, ROP | NR |
| Farkouh 2001; RCS | USA  1997-2000 | N = 12876 babies  Inclusions: non-anomalous live born babies admitted to the NICU between 23-34 completed weeks GA  Exclusions: newborns transferred to/from an outside facility < 28 days, or whose discharge status was unknown | PE/T | 1: MgSO4, N = 4612 babies  2: No MgSO4, N = 8264 babies | Neonatal death (in NICU < 28 days) | Funding: supported by Pediatrix Medical Group  Conflicts: NR |
| FineSmith 1997; CCS | USA  1992-1994 | N = 54 babies (from sample of 492)  Inclusions: birthweight < 1750 g, survival for > 7 days, ≥ 1 CUS > 7 days, with all information on infant’s chart/labour and birth record: GA, history of maternal complications and reason for prematurity, length of ROM, antenatal steroid exposure, MgSO4 exposure, other tocolytic agent exposure, mode of birth, evidence of PE, Apgar score, days intubation  Additional inclusions for cases: cPVL  Additional inclusions for controls: selected from pool infants with normal CUS or IVH grade 1; control pool infants with birthweight outside of range in cPVL group were removed; 2 controls selected per case  Exclusions: NR | PE/T | 1: cPVL, N = 18 babies  2: No cPVL, N = 36 babies | MgSO4 exposure | NR |
| Gano 2016; unclear: results appear to be presented as PCS with CCS(N) | USA  2011-2015 | N = 73 babies  Inclusions: premature newborns < 33 weeks GA admitted to NICU  Exclusions: clinical evidence of a congenital malformation or syndrome, congenital infection or status too unstable for transport to MRI | FN/PE/T | 1: MgSO4, N = 49 babies  2: No MgSO4, N = 24 babies  1: Cerebellar haemorrhage, N = 27 babies  2: No cerebellar haemorrhage, N = 46 babies | Cerebellar haemorrhage (and size), WMI, IVH  MgSO4 exposure | Funding: supported by NIH; 1 author supported by University of California (funded Mark and Lynne Benioff and Bill and Melinda Gates Foundation)  Conflicts: none |
| Garcia Alonso 2018; PCS | Spain  2012-2015 | N = 118 babies  Inclusions: infants born before 32 weeks GA whose mothers received MgS04 as a neuroprotective agent and who were admitted to the NICU, and infants with same GA, who were born in the same time period  Exclusions: infants with other risk factors that contributed to an immediate poor development: a polymalformed patient and another patient who passed away during the 1^st^ hour of life after a placental abruption | FN | 1: MgSO4 (4 g IV LD over 30 minutes; 1 g/hour IV MD until birth or 24 hours; re-treatment permitted), N = 62 babies  2: No MgSO4, N = 56 babies | Resuscitation, IMV, surfactant, BPD, PDA, death, IVH, NEC, PVL, ROP | Funding: NR  Conflicts: none |
| Gasparyan 2017; unclear: PCS  English abstract; article in Russian | CD | N = 62 women and babies  Inclusions: neonates of recurrent delivering women born between 27-28 weeks GA  Exclusions: CD | FN | 1: MgSO4, N = 37 babies  2: No MgSO4, N = 25 babies | IVH, IVH grade 3/4 | CD |
| Ghidini 2001; CCS | USA  1995-1996 | N = 69 babies  Inclusions for cases: consecutive infants diagnoses with NEC  Inclusions for controls: each case matched with 2 controls: next infants admitted to NICU with similar GA and gender, but no NEC  Exclusions: infants with structural or chromosomal anomalies | PE/T (4 g IV bolus LD; 2 g/hour IV MD) | 1: NEC, N = 23 babies  2: No NEC, N = 46 babies | MgSO4 exposure | NR |
| Gibbins 2013; RCS | USA  2007-2011 | N = 373 women and their babies (313 born < 32 weeks in analyses for relevant outcomes)  Inclusions: selected pregnant women with threatened or planned birth < 32 weeks GA  Women eligible for MgSO4 for FN protocol: admitted with viable fetus < 32 weeks with either 1) preterm labour, 2) PPROM, or 3) obstetric/medical indication for birth (e.g. severe PE/FGR)  Exclusions: NR | Predominately FN (unclear whether also given for PE/T) | 1: MgSO4 (6 g IV bolus LD; 2 g/hour IV MD until birth or when birth no longer considered imminent; repeat dosing permitted), N = 247 women (223 babies born < 32 weeks analysed)  2: No MgSO4, N = 126 (90 babies born < 32 weeks analysed) | Apgar score < 7 at 1 minute, Apgar score < 7 at 5 minutes, resuscitation (none, oxygen bag and mask, intubation, chest compressions), discharged alive, NICU admission, LOS (days) (median, range), individual morbidities, hypotonia | Funding: NR  Conflicts: none |
| Girsen 2015; RCS | USA  1999-2002 | N = 2166 women and babies  Inclusions: singleton pregnancies from the Cesarean Registry that were coded for maternal PE and > 37 weeks GA at birth  Exclusions: cases with uterine rupture, chorioamnionitis and major congenital malformations, cases with absence of available data on each variable | PE | 1: MgSO4, N = 1747 babies  2: No MgSO4, N = 419 babies | NICU admission, NICU admission within 2 hours of birth, NICU LOS (days) (median, range), LOS (days) (median, range), Apgar score < 7 at 1 minute, Apgar score < 7 at 5 minutes, Apgar score < 7 at 10 minutes, RDS, ventilation support within 24 hours of birth, prolonged hypotonicity within 72 hours of birth, seizures, sepsis, HIE, neonatal death, prolonged NICU LOS (≥ 8 days) | Funding: assistance of NICHD, the MFMU Network and the Protocol Subcommittee recognised  Conflicts: none |
| Gonzalez-Quintero 2001; PCS  Abstract | USA  1992-1999 | N = 851 babies  Inclusions: neonates with birthweights 500-1000 g  Exclusions: NR | NR | 1: MgSO4, N = 438 babies  2: No MgSO4, N = 413 babies | Overall survival, early survival (alive at 7 days), severe RDS, IVH, PDA, early PDA (< 7 days) | NR |
| Greenberg 2011; RCS with CCS(N) | USA  2006-2008 | N = 252 babies (note: discrepancies in text and tables)  Inclusions: singleton newborn infants who were born at ≥ 35 weeks GA to mothers who received MgSO4 before birth for PE  Exclusions: multiple gestations, neonates who met any antenatal criteria for automatic NICU admission (maternal clinical chorioamnionitis before birth and major fetal anomalies requiring immediate evaluation) | PE | 1: NICU admission, N = 52 babies  2: Well baby nursery admission, N = 200 babies  MgSO4: 4 g IV LD over 20-30 minutes; 1-2 g/hour IV MD | Duration of MgSO4 (hours) (mean ± SD), MgSO4 dose (g) (mean ± SD), > 12 hours MgSO4 exposure, > 30 g MgSO4 exposure | Funding: none  Conflicts: NR |
| Greenberg 2013; RCS | USA  2006-2008 | N = 264 babies  Inclusions: singleton newborns ≥ 37 weeks GA born to women with a diagnosis of PE  Exclusions: multiple gestations, neonates who met any antenatal criteria for automatic NICU admission including a clinical diagnosis of maternal chorioamnionitis and major fetal anomalies requiring immediate evaluation | PE | 1: MgSO4, N = 190 babies  2: No MgSO4, N = 74 babies  1: < 12 hours MgSO4 (< 30 g), N = 132 babies  2: ≥ 12 hours (≥ 30 g), N = 58 babies  MgSO4: 4 g IV LD over 20-30 minutes; 2 g/hour IV MD | Meconium stained AF, NICU admission, initial admission (NICU vs. well baby nursery), primary admit diagnosis (RDS, rule out sepsis, hypotonia, hypothermia, LBW, hyperbilirubinaemia, hypermagnesemia, other), NICU LOS (days) (median, IQR), treatments needed (respiratory, fluids/nutritional support, antibiotics, phototherapy) | Funding: Oscar G and Elsa S Mayer Family Foundation  Conflicts: none |
| Grether 1998; CCS | USA  1983-1985 | N = 168 babies (128 analysed)  Inclusions for cases: infants who weighed < 1500 g at birth and died in 1^st^ 28 days  Exclusions for cases: death from congenital anomalies, in the context of home birth or birth in a military facility, birthweight < 500 g or GA < 22 weeks, birthweight > 1500 g, multiple congenital anomalies  Inclusions for controls: singletons born weighing < 1500 g from the same birth years and counties as the neonatal deaths, who survived to age 3 years and did not have disabling CP | T | 1: Neonatal death, N = 85 babies (53 analysed)  2: Survival to 3 years with no disabling CP, N = 85 babies (75 analysed)  1: Neonatal death no maternal PE, N = 21 babies  2: Survival to 3 years with no disabling CP no maternal PE, N = 35 babies | MgSO4 exposure, MgSO4 exposure for PE, MgSO4 exposure for T | Funding: supported by authors institutions  Conflicts: NR |
| Grimbly 2015; unclear: results appear to be presented as RCS with CCS(N)  Abstract | Authors from USA  2013-2014 | N = 175 babies  Inclusions: neonates < 33 weeks GA at birth  Exclusions: congenital anomalies or conditions that may adversely affect breathing or ventilation | NR | 1: Hypoglycaemia within 1^st^ hour of birth, N = 69 babies  2: No hypoglycaemia, N = 106 babies | MgSO4 exposure | NR |
| Gulcan 2006; PCS | Turkey  2003-2004 | N = 200 babies  Inclusions: consecutive preterm newborns < 37 weeks GA admitted to the NICU  Exclusions: congenital anomaly, birth at another hospital and admission after 24 hours of age with no information on stool passage, death before stool passage, and development of NEC before stool passage | T | 1: MgSO4 (4 g IV LD; 2-3 g/hour IV MD), N = 35 babies  2: No MgSO4, N = 165 babies | RDS | NR |
| Gursoy 2015; PCS | Turkey  2011-2013 | N = 50 babies  Inclusions: AGA neonates born 26-34 weeks GA, whose mothers were exposed to MgSO4 (cases); birthweight and GA matched neonates whose mothers did not received MgSO4 (controls)  Exclusions: neonates with absence of antenatal Doppler examination or presence of absent/reversed end diastolic velocity in umbilical artery, congenital malformation, perinatal asphyxia, and chorioamnionitis | PE/T | 1: MgSO4 (6 g IV LD over 30 minutes; 0.8 g/hour IV MD until birth), N = 25 babies  2: No MgSO4, N = 25 babies | Hypotension, hypertension, NEC, RDS, PDA, ICH stage I-II, feeding intolerance | Funding: NR  Conflicts: none |
| Havranek 2011; RCS | Authors from USA  Time period: NR | N = 56 babies  Inclusions: GA < 37 weeks, and birthweight < 2500 g and AGA  Exclusions: presence of major congenital anomalies, the administration of vasoactive drugs other than caffeine, or a diagnosis of anaemia or polycythaemia | PE/T | 1: MgSO4 in 24 hours prior to birth (4-6 g IV bolus LD; 2-3 g/hour IV MD prior to birth), N = 27 babies  2: No MgSO4, N = 29 babies | Caffeine treatment, ventilator support, phototherapy, umbilical artery catheter, enteral feedings day 1, death during hospitalisation, NEC | NR |
| Hechtman 2002; unclear: RCS with CCS(N)  Abstract | Authors from USA  1997-2000 | N = 85 babies  Inclusions: singleton pregnancies ≥ 23 weeks GA, with birthweights between 500-1000 g  Exclusions: women with PE, PROM or fetal structural or chromosomal abnormalities | T | 1: Neonatal deaths at < 28 days or prior to hospital discharge, N = 19 babies  2: Survivors, N = 66 babies | MgSO4 exposure, MgSO4 dose (g) (median, range), exposure to > 48 g total MgSO4 | NR |
| Holcomb 1991; unclear: NCCS | USA  Time period: NR | N = 23 women, 33 babies  Inclusions for exposed: infants born to women who received IV MgSO4 for T for > 7 days  Inclusions for non-exposed: 2 infants selected for each exposed infant (having no more than 3 days exposure to MgSO4), matched for single/multiple pregnancies and for GA at birth ± 2 weeks  Exclusions: women with pregnancies complicated by IDD, metabolic bone disease, thyroid disease, renal disease, infection with syphilis, toxoplasma, rubella, cytomegalovirus; neonates without chest radiograph in 1^st^ 48 hours | T | 1: MgSO4 > 7 days IV, N = 11 babies  2: No MgSO4, or MgSO4 for < 3 days, N = 22 babies | Definitely abnormal chest radiograph (proximal humeri, radiographic abnormalities: transverse radiolucent and/or sclerotic bands) | NR |
| Hom 2018; RCS  Abstract | Authors from USA  2013-2016 | N = 52 women and babies  Inclusions: women with PPROM up to 34 weeks GA  Exclusions: NR | FN | 1: MgSO4, N = 26 women and babies  2: No MgSO4, N = 26 women and babies | IVH | Funding: NR Conflicts: none |
| Hong 2019; RCS  Abstract | Authors from Korea  2012-2016 | N = 598 babies  Inclusions: babies born 24+0 to 31+6 weeks GA  Exclusions: NR | NR (includes FN) | 1: January 2012-December 2013: MgSO4 for FN not adopted (16.2% exposure), N = 270 babies  2: January 2014-March 2016: MgSO4 for FN used routinely (60.6% exposure), N = 264 babies  3: April 2016-December 2016: MgSO4 abandoned (potential NEC risk) (14.0% exposure), N = 64 babies  1: MgSO4, N = 213 babies  2: No MgSO4, N = 385 babies | Neonatal death, neonatal death due to NEC, NEC, severe NEC (grade ≥ 2), “other neonatal outcomes” | NR |
| Igarashi 1995; unclear: RCS (or NCCS)  English abstract; article in Japanese | CD | N = 42 babies  Inclusions: hypermagnesemic preterm infants born to women treated with MgSO4 for T, and preterm infants born to normal women  Exclusions: CD | T | 1: Hypermagnesemic infants exposed to MgSO4, N = 27 babies (with (N = 15) and without (N = 12) complications)  2: Infants born to “normal mothers”, N = 15 babies | Respiratory and cardiovascular symptoms (respiratory depression, hypotonia, hypotension, requirement for prolonged dopamine and calcium gluconate) | CD |
| Imamoglu 2014; PCS | Turkey  2011-2012 | N = 53 babies  Inclusions: neonates between 26-34 weeks GA  Exclusions: neonates without antenatal Doppler examination and with absent/reversed end diastolic velocity in umbilical artery; neonates with clinical conditions such as congenital malformation, chromosomal anomaly, perinatal asphyxia, sepsis, PROM, chorioamnionitis, polycythaemia, anaemia and whose mothers had multiple pregnancies; neonates with uncertain GA; women having ritodrine for tocolysis; neonates who suffered from hypotension, exposed to drug therapies that could change CBF velocity or arterial blood pressure and any metabolic pathology | PE/T | 1: MgSO4 (6 g IV LD over 30 minutes; 0.8 g/hour IV MD until birth), N = 20 babies  2: No MgSO4, N = 33 babies | RDS, PDA, IVH, caffeine treatment, ibuprofen, inotrope use, phototherapy | Funding: NR  Conflicts: none |
| James 2015; PCS | Ireland  2013-2014 | N = 38 babies  Inclusions: preterm infants < 29 weeks GA exposed to MgSO4; infants not exposed to MgSO4 matched for birthweight, GA, mode of birth  Exclusions: NR | FN | 1: MgSO4 within 4 hours of birth (4 g IV LD over 20 minutes; no subsequent infusion), N = 19 babies  2: No MgSO4, N = 19 babies | IVH grade 3/4, inotropes (1^st^ week), pulmonary haemorrhage, NEC, CLD, death before discharge, early onset sepsis, invasive ventilation days 1 and 2, PDA days 1 and 2 | Funding: support from the EU and Friends of the Rotunda  Conflicts: none |
| Jazayeri 2003; RCS | Authors from USA  1998-2001 | N = 72 women and babies  Inclusions: women who delivered after PPROM < 34 weeks GA who received corticosteroids and antibiotics (cases: received MgSO4; controls matched for GA within a week)  Exclusions: women with clear indication for birth (chorioamnionitis, abnormal fetal surveillance, maternal haemorrhage) | T | 1: MgSO4, N = 36 babies  2: No MgSO4, N = 36 babies | NICU LOS (days) (mean ± SE), meconium, RDS, IVH, NEC, sepsis, neonatal death | NR |
| Jeanneteau 2014; RCS  Abstract | Authors in France  2011-2012 | N = 119 women and their babies  Inclusions: women with fetuses < 33 weeks GA whose birth was planned or expected within 24 hours  Exclusions: NR | FN | 1: MgSO4 (4 g IV LD; 1 g/hour IV MD until birth or for 12 hours), N = 81 women  2: No MgSO4, N = 38 women | Apgar score < 7 at 5 minutes, closed cardiac massage, adrenaline, “neonatal morbi-mortality” | NR |
| Jones 2018; RCS  Abstract | Authors in USA  2012-2014 | N = 120 babies  Inclusions: infants born between 32+0-33+6 weeks GA  Exclusions: NR | PE/T | 1: MgSO4, N = NR  2: No MgSO4, N = NR | Adverse bowel events | Funding: NR Conflicts: none |
| Jung 2018; RCS | South Korea  2005-2013 | N = 184 women and their babies  Inclusions: singleton pregnancies complicated by PPROM at 23+0-31+6 weeks GA who were hospitalised and received MgSO4 for T (MgSO4 group) or did not receive T (MgSO4, beta-adrenergic receptor agonists, or calcium-channel blockers) (no MgSO4 group)  Exclusions: patients who were not candidates for expectant management of pregnancy at admission, such as those with intrauterine infection, significant vaginal bleeding, placental abruption, cord prolapse, non-reassuring fetal status, or advanced labour, patients with multifetal gestations, pregnancy associated hypertension, fetal anomalies, and IUGR | T | 1: MgSO4 (6 g IV LD over 30 minutes; 1 g/hour IV MD until uterine quiescence), N = 143 women and babies  2: No MgSO4, N = 41 women and babies | Stillbirth, neonatal death, early neonatal death, perinatal death, Apgar score < 7 at 5 minutes, pulmonary hypoplasia, RDS, BPD, NEC, early onset sepsis, ROP, ROP grade 2/3, hearing impairment, NICU LOS (days) (mean ± SD), IVH, IVH grade 3/4, PVL, bone abnormalities | Funding: no support  Conflicts: none |
| Kamilya 2005; NCCS | India  1995-1997 and 2002-2004 | 2002-2004: N = 26752 births, 769 women with E, 474 women with antepartum/intrapartum E, 481 babies  1995-1997: N = 31352 births, 877 women with E, 731 women with antepartum/intrapartum E, 724 babies  Inclusions: all births; antepartum and intrapartum E included for assessment of perinatal death  Exclusions: NR | E | 1: 2002-2004 (almost universal MgSO4 use), N = 481 babies  2: 1995-1997 (no MgSO4 use), N = 724 babies | Perinatal death | NR |
| Kamyar 2015a; RCS  Abstract | Authors in USA  2003-2013 | N = 271 babies  Inclusions, non-anomalous singleton infants weighing ≤ 1000 g  Exclusions: NR | FN/PE/T | 1: MgSO4, N = 133 babies  2: No MgSO4, N = 138 babies | Composite morbidity (IVH, PVL,  BPD, NEC, RDS, ROP and/or death), neonatal death, individual morbidities | NR |
| Kamyar 2015b; RCS  Abstract | USA  2003-2013 | N = 1246 babies  Inclusions: infants born 23+0 to 31+6 weeks GA  Exclusions: multiple gestations, fetal anomalies, aneuploidy | FN/PE/T | 1: MgSO4, N = 457 babies  2: No MgSO4, N = 789 babies | Composite morbidity  (IVH, BPD, NEC, and/or death prior to hospital discharge), neonatal death | NR |
| Kamyar 2015c; RCS (secondary analysis of RCT)  Abstract | NR (see Rouse 2008) | N = 2431 babies  Inclusions: women with non-anomalous singleton and twin gestations born ≥ 24 weeks GA  Exclusions: NR | FN | Males, N = 1147 babies  1: MgSO4, N = 643 babies  2: No MgSO4, N = 504 babies  Females, N = 1284 babies  1: MgSO4, N = 536 babies  2: No MgSO4, N = 748 babies | Composite severe morbidity (IVH grade 3/4, PVL, BPD, NEC, and/or death) | NR |
| Kamyar 2016a; RCS (secondary analysis of RCT) | See Rouse 2008 | N = 396 babies  Inclusions: women diagnosed with intrapartum clinical chorioamnionitis (clinical diagnosis of chorioamnionitis with ≥ 1 of: maternal temperature of > 37.8°C or antibiotic administration for the documented indication of chorioamnionitis; chorioamnionitis noted on placental pathology alone was not considered sufficient for a diagnosis of chorioamnionitis)  Exclusions: no chorioamnionitis, twins, loss to follow up before birth | FN | 1: MgSO4 (6 g IV LD over 20-30 minutes; 2 g/hour IV MD), N = 192 babies  2: Placebo, N = 204 babies | Stillbirth or death by age 1, severe composite morbidity (1 or more of: sepsis, severe IVH, PVL, NEC stage 2/3, BPD), sepsis, severe IVH, PVL, NEC stage 2/3, BPD, death before hospital discharge | Funding: support from The University of Utah and National Center for Research Resources and the National Center for Advancing Translational Sciences, NIH  Conflicts: none |
| Kamyar 2016b; RCS with CCS(N) (secondary analysis of RCT) | USA  1997-2004 | N = 697 babies  Inclusions: singleton and twin infants admitted, randomised, and born between 23.0-27.9 weeks GA  Exclusions: infants with chromosomal abnormalities, major congenital malformations, and/or with incomplete outcomes | FN | 1: MgSO4, N = 332 babies  2: No MgSO4, N = 365 babies  1: MgSO4, N = 148 babies born < 26 weeks GA  2: No MgSO4, N = 145 babies born < 26 weeks GA  MgSO4 exposed babies  1: NICU death and/or NEC stage 2/3, N = 73 babies  2: Survival without NEC stage 2/3, N = 259 babies | Death and/or NEC stage 2/3  MgSO4 infusing at birth, total amount of MgSO4 received (g) (mean ± SD) | Funding: supported by NIHCD  Conflicts: NR |
| Katayama 2011; RCS | Japan  2004-2008 | N = 160 babies  Inclusions: extremely preterm neonates (GA < 28 weeks), all who received prophylactic indomethacin within 6 hours of birth  Exclusions: infants with chromosomal abnormalities | T (1 woman for PE) | 1: MgSO4 (no LD; 0.5-1 g/hour IV MD), N = 41 babies  2: No MgSO4, N = 119 babies  MgSO4  1: Low dose (< 50 g), N = 19 babies  2: High dose (≥ 50 g), N = 22 babies | Early closure of DA, symptomatic PDA, successful response to indomethacin of PDA, successful response to surgical treatment of PDA, failure of early closure of DA | Funding: NR  Conflicts: none |
| Kelly 1992; PCS  Abstract | NR | N = 10 women and babies  Inclusions: women treated with continuous MgSO4 infusion for preterm labour, and controls (30-35 weeks GA)  Exclusions NR | T | 1: MgSO4 IV, N = 5 babies  2: No MgSO4, N = 5 babies | Morbidity | NR |
| Khodapanahandeh 2008; CCS | Iran  2004-2005 | N = 121 babies  Inclusions: VLBW (< 1500 g) infants admitted (cases: IVH grade 3/4)  Exclusions: deaths during the 1^st^ 48 hours of life | T | 1: IVH grade 3/4, N = 39 babies  2: No IVH grade 3/4, N = 82 babies | MgSO4 exposure | NR |
| Kimberlin 1998; RCS | USA  1992-1992 | N = 308 babies (363 in death analyses)  Inclusions: singleton infants ≤ 1000 g with a GA ≥ 20 weeks who were not the product of an induced abortion or antepartum stillbirth, who survived > 2 days after birth, who were born without major anomalies, who were deemed potentially viable by the obstetricians, and would have undergone a caesarean birth for fetal indications  Exclusions: infants born to women diagnosed with PE | T | 1: MgSO4, N = 124 babies (138 for death analyses)  2: No MgSO4, N = 184 babies (225 for death analyses) | Death ≤ 2 days, death 3-120 days, intact survival (survival to hospital discharge or 120 days without any serious morbidities), death at ≥ 2 days and < 120 days, IVH grade 3/4, ROP grade 3/4, abnormal neurological evaluation, seizure activity, NEC requiring surgery, oxygen dependence at discharge, duration of ventilation (days) (median, measure of variance NR), NICU LOS (days) (mean ± SD) | NR |
| Koksal 2002; unclear: results appear to be presented as PCS with CCS(N) | Turkey  Year NR | N = 120 babies  Inclusions: live born infants with birthweights 750-1500 g  Exclusions: NR | T | 1: Infants with IVH; with severe abnormalities, grade 3/4 GMH-IVH or PVL, N = 18 babies  2: Infants with minimal, grade 1/2 GMH-IVH or no abnormalities, N = 102 babies | MgSO4 exposure | NR |
| Kuban 1992; PCS | USA  1984-1987 | N = 449 babies  Inclusions: weighed 1500 g or less, and had a CUS performed within the 1^st^ 15 days after birth  Exclusions: NR | PE/T | 1: MgSO4, N = 90 babies  2: No MgSO4, N = 359 babies | GMH-IVH | Funding: The Joint Program of Neonatology  Conflicts: NR |
| Lai 2017; RCS  Abstract | Authors from Taiwan  12 year study | N = NR  Inclusions: women diagnosed with PE who delivered ≥ 37 weeks GA  Exclusions: NR | PE | 1: MgSO4, N = NR  2: No MgSO4, N = NR | Muscle tone scores (units NR), SCBU admission, NICU admission, delayed adaptation | NR |
| Lee 2013; RCS  English abstract; article in Korean | Korea  2005-2012 | N = 81 babies  Inclusions: VLBW infants born to women with PE who had been admitted to the NICU  Exclusions: NR | PE | 1: MgSO4, N = 20 babies  2: No MgSO4, N = 61 babies  1: MgSO4 and sPDA, N = 15 babies  2: MgSO4 and no sPDA, N = 5 babies | RDS, ventilation, sPDA, operated PDA, ROP, NEC, IVH grade ≥ 1, PVL, death | CD |
| Lee 2015; RCS  Abstract | Korea  2007-2013 | N = 570 women and babies  Inclusions: pregnant women with preterm birth, and their neonates  Exclusions: NR | T | 1: MgSO4 IV, N = 101 babies  2: No MgSO4, N = 469 babies | Hypocalcaemia | NR |
| Leung 2016; unclear: results appear to be presented as PCS with CCS(N) | USA  1999-2003 | N = 289 babies  Inclusions: neonates born < 33 weeks GA with birthweight < 1501 g who were enrolled in a prospective study of the relationship of inflammatory markers and invasive ureaplasma with respiratory and CUS outcomes; with documented hearing screen  Exclusions: congenital brain/neural tube defects, confirmed congenital infections and no available cord blood or venous sample within 12 hours of birth | PE/E/T | 1: Passed hearing screen, N = 244 babies  2: Failed hearing screen, N = 45 babies | MgSO4 exposure, MgSO4 and betamethasone exposure | Funding: supported by the University of Maryland and NIH  Conflicts: none |
| Leviton 1997; PCS | Authors from USA  1991-1993 | N = 1331 women and 1518 babies  Inclusions: infants weighing 500 to 1500 g when born  Exclusions: death before CUS, or unavailability of CUS and information about receipt of MgSO4 and potential confounders | Unclear (PE/PIH/T) | 1: MgSO4, N = 678 babies  2: No MgSO4, N = 840 babies | IVH, PEA: early, late, any, hypoechoic image, late hypoechoic image, ventriculomegaly | Funding: National Institute of Neurological Disorders and Stroke  Conflicts: NR |
| Lipsitz 1971; unclear: PCS (or NRT) | Authors from USA  Time period NR | N = 37 babies  Inclusions: newborn infants (all between 33-42 weeks GA) born to toxaemic women who received MgSO4 as per regimens to the right  Exclusions: NR | PE/E | 1: MgSO4 IV LD and MD (2-4 g IV LD; 1 g/hour IV MD - Zuspan’s regimen), N = 29 babies  2: MgSO4 IV LD, IM MD (2-3 g IV LD; IM MD), N = 8 babies | Apgar score < 7 at 1 minute, Apgar score < 7 at 5 minutes, clinical score of 3, clinical score > 0 (the higher the score, with a maximum of 3, the greater the apparent toxicity of excess Mg: 1 point for flaccidity and hyporeflexia, 1 for resuscitation or assisted ventilation, 1 for week or absent cry unrelated to tracheal intubation), death, resuscitation, assisted ventilation | NR |
| Lloreda-Garcia 2016; NCCS  English abstract; article in Spanish | CD | N = 107 babies  Inclusions: preterm infants < 32 week exposed to MgSO4 for FN, and a historic group immediately prior to this treatment  Exclusions: infants that had not reached lung maturity with corticosteroids | FN | 1: MgSO4, N = 56 babies  2: No MgSO4, N =51 babies | Resuscitation, Apgar score ≤ 5 at 1 minute, Apgar ≤ 5 at 5 minutes, CPAP/nasal IMV, CMV, HFOV, surfactant treatment, PDA treated, vasoactive drugs, blood products, sepsis confirmed, pathological brain ultrasound, no stools at 48 hours, no bowel movements at 72 hours , NEC, death, CRIB (median, range), meconium evacuation delay, parenteral nutrition | CD |
| Martin 1998; RCS | Authors from USA  1992-1994 | N = 193 women and babies  Inclusions = pregnancies complicated by severe PE (according to the ACOG criteria) between 26-32 weeks GA, and (for comparison), pregnancies delivered due to preterm labour during the same period between 26-32 weeks GA  Exclusions: diagnosis of PPROM | PE/T | 1: MgSO4, N = 118 babies  2: No MgSO4, N = 75 babies | IVH | NR |
| Matsuda 1997; RCS with CCS(N) | Japan  1992-1994 | N = 139 babies born to 114 women; and a further 51 control babies  Inclusions: neonates born to all pregnant women who received IV MgSO4 (cases); neonates born to pregnant women given no MgSO4 in the same period (controls)  Exclusions: pregnancies complicated by metabolic bone disease, thyroid disease, renal disease, congenital infections (syphilis, toxoplasmosis, cytomegalovirus) | PE/T | 1: MgSO4 (according to Zuspan’s regimen: 4 g IV LD over 30 minutes; 1-2 g/hour IV MD), N = 114 women and 139 babies  2: No MgSO4, N = 51 babies  1: MgSO4 and bone abnormalities, N = 13 babies  2: MgSO4 and no bone abnormalities, N = 101 babies | Bone abnormalities  GA at start of MgSO4 (weeks) (mean ± SD), duration of MgSO4 (days) (mean ± SD), MgSO4 dose (g) (mean ± SD) | NR |
| McGuiness 1980; NRT | Authors from USA  1977-1978 | N = 37 women and their babies  Inclusions: consecutive women with PE, thought clinically to be at term with appropriately grown infants (study group); normotensive women at term with appropriately grown infants (control group)  Exclusions: NR | PE | 1: MgSO4 (4 g IV LD over 30 minutes; 1-2 g/hour IV MD), N = 23 women and their babies  2: Dextrose-water or dextrose-saline, N = 14 women and their babies | Significant birth asphyxia, hypocalcaemia | NR |
| McPherson 2014; RCS (secondary analysis of RCT) | USA  1997-2004 | N = 933 women and their babies  Inclusions: women at high risk for preterm delivery between 24-31 weeks GA because of ROM (between 22-31 weeks GA), spontaneous labour with cervical dilation of 4-8 cm, or anticipated indicated preterm delivery within 2-24 hours, with singleton, non-anomalous fetuses (diagnosed before or after birth) randomised to MgSO4 who received the study drug  Exclusions: NR | FN | 1: MgSO4 < 12 hours cumulative, N = 356 women and babies  2: MgSO4 12-18 hours cumulative, N = 341 women and babies  3: MgSO4 > 18 hours cumulative, N = 236 women and babies  MgSO4: 6 g IV LD over 20-30 minutes; 2 g/hour IV MD until birth or for 12 hours; re-treatment permitted | Apgar score < 7 at 5 minutes, resuscitation in delivery room (oxygen blow-by, oxygen bag, mask or both, intubation, chest compressions), NEC, ROP, RDS, MV, BPD, seizures, any IVH, IVH grade 3/4, NICU admission | Funding: NR  Conflicts: none |
| Mikhael 2019; RCS | USA  2010-2016 | N = 302 babies  Inclusions: babies with a birthweight ≤ 1000 g and/or GA ≤ 28 weeks with no congenital gastrointestinal anomalies  Exclusions: NR | NR (includes FN) | 1: MgSO4 ≤ 7 days prior to birth, N = 210 babies  2: No MgSO4 ≤ 3 days prior to birth, N =192 babies  1: MgSO4 ≤ 3 days prior to birth, N = 179 babies  2: No MgSO4 ≤ 3 or ≤ 7 days prior to birth, N = 123 babies  1: MgSO4 ≤ 3 days prior to birth, N = 179 babies  2: No MgSO4 ≤ 3 days prior to birth, N = 31 babies  1: Pre MgSO4 protocol implementation, N = 112 babies  2: Post MgSO4 protocol implementation, N = 190 babies  4 g IV LD over 30 minutes; 2 g/hour IV MD for 12 hours or until birth; repeated if birth not within 12 hours (repeat LD if > 6 hours has passed since discontinuation) | Death, early death, postnatal steroids, NEC, early NEC, SIP, early SIP, SIP or NEC or death, early SIP or NEC or death, late onset-sepsis, postnatal NSAIDs for PDA, IVH ≥ grade 3  Early defined as: 1^st^ 2 weeks of life | Funding: NIH  Conflicts: none |
| Mitani 2011; RCS with CCS(N) | Japan  2006-2007 | N = 425 babies  Inclusions: single, spontaneous preterm births born between 22-31 weeks GA  Exclusions: chromosomal abnormalities and/or anomalous births | T | 1: MgSO4 (4 g IV LD over 30 minutes; 1-2 g/hour IV MD), N = 236 babies  2: No MgSO4, N = 189 babies  1: Adverse outcome (IVH, PVL, CP, infantile death), N = 80 babies  2: Good outcome, N = 315 babies  1: < 2 days MgSO4, N = 49 babies  2: > 2 days MgSO4, N = 174 babies  1: MgSO4 and adverse outcome, N = 49 babies  2: MgSO4 and good outcome, N = 174 babies | Fetal and neonatal death, Apgar score < 7 at 1 minute, Apgar score < 7 at 5 minutes, RDS, IVH, PVL  MgSO4 exposure  Combined adverse outcome (death, IVH, PVL, CP), IVH, PVL  Duration of MgSO4 (hours) (median, range) | NR |
| Mittendorf 2005; RCS (secondary analysis of RCT)  Abstract | NR (see Mittendorf 2002) | N = 146 babies  Inclusions: surviving neonates with data on antenatal MgSO4 exposure and neuropathogenesis (IVH grade 2 and/or LSV)  Exclusions: NR | FN/T | 1: MgSO4 0 to 4 g, N = 90 babies  2: MgSO4 5 to 49 g, N = 23 babies  3: MgSO4 ≥ 50 g, N = 33 babies | IVH grade 3 and/or LSV | NR |
| Mittendorf 2009; RCS (secondary analysis of RCT)  Abstract | NR (see Mittendorf 2002) | N = 140 babies  Inclusions: babies with HUS data linked to MgSO4 exposure  Exclusions: NR | FN/T | 1: No MgSO4, N = 64 babies (note: women received other tocolytic)  2: MgSO4 > 0 to < 10 g, N = 27 babies  3: MgSO4 10 to < 30 g, N = 8 babies  4: 30 to < 50 g, N = 11 babies  5: ≥ 50 g, N = 30 babies | TSV | NR |
| Morag 2015; RCS | Israel  2015 | N = 645 women and 705 babies  Inclusions for ‘study group’: infants born 34+0-35+6 weeks GA, born alive  Inclusions for matched term infants: infants born 37+0-41+6 weeks GA within 2 weeks of index case, matched for gender and mode of birth  Exclusions: infants born at 36 weeks GA, diagnosed with genetic syndromes or major malformations | PE | 1: Preterm infants, N = 235 babies  2: Term infants, N = 470 babies  1: Preterm infants with MgSO4, N = 10 women  2: Preterm infants with no MgSO4, N = 168 women | Respiratory disease (including RDS, TTN and disorders of air leak such as pneumothorax and pneumomediastinum) | Funding: NR  Conflicts: none |
| Morag 2016; RCS | Authors from Israel  2012-2013 | N = 190 babies  Inclusions: infants admitted to a tertiary care NICU, born < 32 weeks GA without congenital anomalies or known genetic disorders Exclusions: NR | FN/PE | 1: MgSO4 (5 g IV LD over 30 minutes; 2 g/hour IV MD), N = 145 babies  2: No MgSO4, N = 45 babies | Apgar score < 7 at 1 minute, Apgar score < 7 at 5 minutes, IV (days) (mean ± SD), treated early hypotension, intubation, oxygen at 28 days, oxygen at 36 weeks, proven NEC, sepsis, IVH grade 3/4/PVL, discharge (week) (mean ± SD), death | Funding: NR  Conflicts: none |
| Moschos 2001; CCS  Abstract | 2 year period | N = 75 babies  Inclusions: NEC cases, and controls matched for GA  Exclusions: infants with structural or chromosomal anomalies | NR | 1: NEC, N = 25 babies  2: No NEC, N = 50 babies | MgSO4 exposure | NR |
| Murata 2005; unclear: results appear to be presented as RCS with CCS(N) | Japan  1992-1997 | N = 201 babies  Inclusions: all appropriate for date babies born at 24 to 33 weeks GA (maternal transports with predominant indications for maternal transfers and then all inborn neonates)  Exclusions: babies with major anomalies, with IVH grade 3/4, or who died within 2 weeks after birth | T | 1: cPVL, N = 35 babies  2: No cPVL, N = 166 babies  MgSO4: 1-4 g/hour IV LD; 0.5-2 g/hour IV MD | MgSO4 exposure | NR |
| Nakamura 1991; unclear: results appear to be presented as RCS with CCS(N)  Abstract | NR | N = 58 women and their babies  Inclusions: premature or PE women treated with MgSO4 and their newborns (4 g IV LD over 30 minutes, 1-2.5 g/hour IV MD)  Exclusions: NR | PE | 1: Ileus, N = NR  2: No ileus, N = NR | MgSO4 dose (g) (mean ± SD) | NR |
| Narasimhulu 2017; RCS | USA  2013-2014 | N = 304 women and babies  Inclusions: neonates born at 24-33+6 weeks GA admitted to the NICU  Exclusions: neonates with major congenital malformations or chromosomal anomalies, neonates transferred from outside facilities | FN/PE | 1: MgSO4 (4 g IV LD for PE, 6 g IV LD for FN; 2 g/hour IV MD), N = 237 women and babies  2: No MgSO4, N = 67 women and babies | Apgar score ≤ 5 at 1 minute, Apgar score ≤ 5 at 5 minutes, delivery room resuscitation, hypotension, hypocalcaemia, IVH grade 3/4, BPD, ROP grade 3+, PVL, intubation, NEC, PDA, death, composite outcome (death, IVH grade 3/4, BPD, ROP grade 3+, PVL, NEC), NICU LOS (days) (median, Q1-Q3) | Funding: NR  Conflicts: none |
| Nassar 2006; RCS | Lebanon  1995-2003 | N = 155 women  Inclusions: all women admitted for IV MgSO4 T at ≥ 25 weeks GA  Exclusions: women who required a combination of other tocolytics and those with an underlying maternal disease like pre-existing hypertension or renal disease | T | 1: MgSO4 > 48 hours, N = 78 women, 112 babies  2: MgSO4 ≤ 48 hours, N = 77 women, 86 babies  MgSO4: 4 g IV LD over 20 minutes; 2 g/hour, increased up to 4 g/hour IV MD | Apgar score < 4 at 1 minute, Apgar score < 7 at 5 minutes, hypotonia, IVH, neonatal deaths (per 1,000), abnormal bone mineralisation | NR |
| Nelson 1995; CCS | USA  1983-1985 | N = 117 babies  Inclusions for cases: singletons who weighed < 1500 g at birth, survived to age 3 years (residents in California to that age), with moderate or severe congenital CP  Exclusions: children with mild CP, with abnormalities of tone/reflexes but no functional impairment, and those with isolated hypotonia or disability acquired after 1^st^ 28 days of life, or non-accidental head trauma in 1^st^ month of life  Inclusions for controls: randomly selected, approximately 2 per case, from the population of infants < 1500 g at birth, born in same counties and birth years, who survived to age 3 years | PE/T | 1: CP, N = 42 babies  2: No CP, N = 75 babies  For review outcomes:  1: No CP, MgSO4, N = 27 babies [discrepancy in text/table]  2: No CP, no MgSO4, N = 48 babies | Apgar score < 6 at 5 minutes, ICH/IVH | Funding: supported by Center for Environmental Health and Injury Control, CDC, ATSDR, US Public Health Service, and US DHHS Maternal and Child Health Bureau  Conflicts: NR |
| Nunes 2018; RCS | Brazil  2009-2014 | N = 75 women, 99 babies (94 available for analyses)  Inclusions: patients with premature newborn deliveries between 24-32 weeks GA  Exclusions: NR | FN | 1: MgSO4 (4 g IV LD over 30 minutes; no MD), N = 26 babies  2: No MgSO4, N = 68 babies | Abnormal heart rate, abnormal respiratory rate (tachypnoea), abnormal temperature (hypothermia, hyperthermia), oxygen saturation < 95%, Hemoglucotest abnormal (hypoglycaemia, hyperglycaemia), anaemia (haemoglobin < 16.4 g/dL), ventilation: non-invasive, ventilation: ET | Funding: NR  Conflicts: none |
| Okusanya 2012; NRT | Nigeria  2008-2009 | N = 103 women and their babies  Inclusions: severe PE or E at the antenatal and labour ward (detailed definitions provided)  Exclusions: women who had diazepam prior to arrival and those with history of chronic seizure disorder | PE/E | 1: MgSO4 10 g IM LD; 5 g/4 hours IM MD until 24 hours after birth or last convulsion, N = 54 women and their babies  2: MgSO4 4 g IV and 10 g IM LD; 5 g/4 hours IM MD until 24 hours after birth or last convulsion, N = 49 women and their babies | Apgar score < 7 at 5 minutes, perinatal death | NR |
| Ozlu 2019; RCS | Turkey  2011-2016 | N = 280 babies  Inclusions: babies hospitalised in the NICU ≤ 32 weeks GA with completed antenatal steroid doses  Exclusions: babies who did not receive complete antenatal steroid doses, who had congenital anomalies, who were exposed to MgSO4 for PE, or who were sent to another hospital | FN | 1: 2014-2016 (post MgSO4 implementation; unclear uptake), N = 108 babies  2: 2011-2012 (pre MgSO4 implementation), N = 172 babies  MgSO4 implemented in 2013: 6 g IV LD over 30 minutes, 2 g/hour IV MD until birth or 24 hours | Death, resuscitation at birth, RDS, ventilator support, ventilation (days) (mean ± SD, and median, minimum and maximum), BPD, oxygen use (days) (mean ± SD, and median, minimum and maximum), NEC, early neonatal sepsis, feeding intolerance, could not get full enteral feeding, could not start any enteral feeding, starting day of enteral feeding (day) (mean ± SD, and median, minimum and maximum), time of full enteral feeding (day) (mean ± SD, and median, minimum and maximum), PDA, ROP, IVH, IVH grade 3/4, duration of hospital stay (days) (mean ± SD, and median, minimum and maximum) | Funding: none  Conflicts: none |
| O Reilly 2016; RCS  Abstract | Ireland  2012 | N = 100 babies  Inclusions: preterm infants born 24-32 weeks GA whose mothers did/did not receive MgSO4 IV  Exclusions: NR | FN | 1: MgSO4 IV, N = 55 babies  2: No MgSO4, N = 45 babies | Duration of intubation (hours) (median, variance measure NR) | NR |
| Palatnik 2019; CCS | USA  2011-2015 | N = 779 babies  Inclusions: babies born from singleton/twin pregnancies at 23+1 to 31+6 weeks GA  Cases: babies diagnosed with early onset neonatal sepsis on blood or CSF culture in 1^st^ 72 hours of life, or who died in the 1^st^ week of life  Controls: eligible babies who did not meet criteria for cases  Exclusions: babies transferred from outside institutions, born following pregnancies complicated by major fetal anomaly or with no intent for resuscitation | NR | 1: Cases (early onset neonatal sepsis or death in 1^st^ week of life), N = 73 babies  2: Controls, N = 706 babies | MgSO4 exposure | Funding: none  Conflicts: none |
| Paneth 1991; PCS | USA  1984-1987 | N = 1037 babies  Inclusions: geographically representative sample of infants weighing 2000 g or less born or cared for in 3 NICUs  Exclusions: infants with insufficient information about details of labour and birth to permit satisfactory classification of maternal exposure | PE/PEH/T | 1: MgSO4, N = 362 babies  2: No MgSO4, N = 675 babies | GM/IVH, PEL/VE, neonatal death | Funding: NINDS  Conflicts: NR |
| Perlman 1995; RCS  Abstract | Authors from USA  1988-1992 | N = 1025 babies  Inclusions: singleton preterm infants < 1500 g  Exclusions: NR | PIH | 1: MgSO4, N = 192 babies  2: No MgSO4, N = 833 babies | PV-IVH, IVH grade 3/4 | NR |
| Petrov 2013; NRT  Abstracts (2) | Authors from Moldova | N = 140 women and babies  Inclusions: pregnant women with monofetal pregnancies, from 26-33+6 weeks GA, who delivered to 34 weeks GA  Exclusions NR | FN | 1: MgSO4 (IV LD over 15 minutes; 1g/hour IV MD), N = 80 babies  2: Placebo, N = 60 babies | Neurological complications, haemodynamic complications | NR |
| Petrova 2012; RCS with CCS(N) | USA  2004-2008 | N = 178 babies  Inclusions: GA 23-31 weeks, no congenital malformations or reports of maternal hypertension or PE, HUS done during 1^st^ 14 days postpartum  Inclusions for controls: double-match approach used, matched by exact GA in completed weeks and by same/similar birthweight (± 100 g); due to non-availability or > 1 control for ELBW infants, the authors selected 1 control; where several controls available, 1 randomly selected  Exclusion criteria: NR | T | 1: IVH, N = 89 babies  2: No IVH, N = 89 babies  MgSO4: 4-6 g IV LD over 30 minutes; 1-3 g/hour IV MD until 12-24 hours uterine quiescence | MgSO4 exposure | Funding: Memorial Research Fund  Conflicts: none |
| Qasim 2017; unclear: results appear to be presented as PCS with CCS(N)  Abstract | Authors from USA | N = 105 babies  Inclusions: premature infants < 32 weeks GA and < 1500 g  Exclusions: NR | NR | 1: MgSO4, N = 95 babies  2: No MgSO4, N = 10 babies | HsPDA | NR |
| Rantonen 2001; PCS | Finland  1996-1998 | N = 55 babies (19 were in a ritodrine exposure group and not further considered)  Inclusions: preterm infants consecutively born at < 33 weeks GA, with no major congenital malformations, and written informed consent  Exclusions: no exclusions | PE/T | 1: MgSO4 (5 g IV LD over 20 minutes; 1-2 g/hour IV MD), N = 19 babies  2: No MgSO4 (born immediately after exposed infants, with no T or anticonvulsants), N = 19 babies | Dexamethasone, dopamine, dobutamine, surfactant, PDA, PV-IVH grade 1-4, PV-IVH grade 3/4, HIE/increased echodensity, RDS and MV, NICU admission, death, blood-culture confirmed septicaemia | Funding: supported by Research Foundation of the Orion Corporation, T Turku University Foundation, and the Sigrid Juselius Foundation, Finland  Conflicts: NR |
| Rasch 1982; PCS | USA  Time period NR | N = 79 babies  Inclusions: infants of mothers with PE treated with MgSO4, infants of women with PE not treated with MgSO4, and infants of normal women (definition for PE provided)  Exclusions:  Infants born to women with pre-existing hypertension or other chronic diseases, preterm infants and infants with complicating factors such as tight nuchal cord, documented late decelerations, signs of sepsis, or asphyxia, infants whose mothers had received general anaesthesia or doses of sedative drugs within 2.5 hours of birth | PE | 1: Born to PE women treated with MgSO4 (4 g IV and 10 g IM LD; 0.3 g/hour IV MD until birth), N = 36 babies  2: Born to PE women with no MgSO4, N = 18 babies  3: Born to normal women, N = 25 babies | Poor sucking and cry response, cyanosis during feedings, requirement for IV fluid treatment, neurologic section of the Dubowitz examination at birth, over 24 hours after birth, individual measures of Dubowitz examination | NR |
| Rattray 2014; NCCS | USA  2009-2010, 2011 | N = 155 babies  Inclusion: singleton and twin gestation ELBW (< 1000 g) inborn and admitted to NICU  Exclusions: infants born with major congenital malformations or chromosomal anomalies | FN | 1: Pre-MgSO4 FN protocol (January 2009 – July 2010; 50.6% MgSO4), N = 81 babies  2: During MgSO4 FN protocol (July – November 2010; 78.3% MgSO4), N = 23 babies  3: After MgSO4 FN protocol (January – October 2011; 60.8% MgSO4), N = 51 babies  MgSO4: 6 g IV LD; 2 g/hour IV MD until birth or 12 hours; re-treatment permitted | Postnatal hydrocortisone, postnatal NSAIDs, SIP or death, SIP, death | Funding: NR  Conflicts: none |
| Rauf 2017; RCS | Turkey  2011-2016 | N = 107 women and babies  Inclusions: maternal age between 18-39 years, with singleton pregnancies born before 32nd week of pregnancy  Exclusions: pregnant women who were treated with MgSO4 for T or E prophylaxis, multiple pregnancies, fetal death, associated fatal congenital anomalies or chromosomal abnormalities, women with contraindications for MgSO4 use | FN | 1: MgSO4 (6 g IV LD over 30 minutes; 2 g/hour IV MD until birth or up to 12 hours), N = 46 babies  2: No MgSO4, N = 61 babies | Active resuscitation at birth (respiratory support with ET intubation), NICU LOS (days) (mean ± SD), respiratory support, MV, nasal CPAP, nasal SMIV, oxygen hood, IVH and grade 1-4, PVL, convulsion, hypotonia, encephalopathy, ROP, neonatal death | NR |
| Rhee 2012; PCS | USA  2001 | N = 23 women and 22 babies (with usable specimens)  Inclusions for exposed: women exposed to MgSO4 for PE/T at the time of birth  Inclusions for unexposed: women who presented in labour with no evidence of PE or preterm labour and did not require MgSO4  Exclusions: women with multiple gestations and women unable to give consent | PE/T | 1: MgSO4 (‘standard protocol’), N = 11 women, 10 babies  2: No MgSO4, N = 12 women, 12 babies | NICU admission, Apgar score < 7 at 5 minutes | Funding: NR  Conflicts: none |
| Riaz 1998; PCS with CCS(N) | USA  1995-1996 | N = 52 babies  Inclusions: infants whose GA was ≥ 34 weeks and whose mothers received a minimum of 12 hours of MgSO4 (study group), and the next infant of similar GA born after enrolment of study infant (control group)  Exclusions: infants with severe congenital anomalies, neuromuscular disorders, significant parenchymal lung disease, and adverse intrapartum event | PIH/T | 1: MgSO4 (1-3 g/hour IV), N = 26 babies  2: No MgSO4, N = 26 babies  1: MgSO4 and NICU admission, N = 12 babies  2: MgSO4 and no NICU admission, N = 14 babies | Hypotonia, delivery room support (bag and mask ventilation), NICU admission, delayed adaptation, presumed or ruled-out sepsis, delayed feeding (1^st^ feeding ≥ 8 hours after birth), feeding intolerance, hospital stay (days) (mean ± SD), apnoea density (mean ± SD), apnoea ≥ 15 seconds (associated with bradycardia) (mean ± SD), apnoea ≥ 10 seconds (mean ± SD), pathologic apnoea (≥ 15 seconds associated with bradycardia)  MgSO4 dose (g) (mean ± SD), duration of MgSO4 (hours) (mean ± SD) | NR |
| Rizzolo 2019; RCS  Abstract | Canada  2013-2017 | N = 3788 babies  Inclusions: babies born 23+0-28+6 weeks GA admitted to NICUs participating in the Canadian Neonatal Network  Exclusions: NR | FN | 1: MgSO4, N = NR  2: No MgSO4, N = NR | Death or SNI (grade ≥ 3 IVH and/or PVL) | NR |
| Sahin 2001; PCS | Turkey  1995-1996 | N = 40 babies  Inclusions for ‘cases’: newborns from women with PE or E who had been treated with MgSO4  Inclusions for ‘controls’: newborn from normal pregnant women, who did not receive any drug that could affect the contractility of smooth muscles  Exclusions: NR | PE/E | 1: MgSO4 (4 g IV and 10 g IM LD, 5 g in each buttock; 5 g/4 hours IM MD until 24 hours after birth), N = 20 babies  2: No MgSO4, N = 20 babies | Not voiding in 1^st^ 24 hours, residual urine after 1^st^ micturition (> 5 mL), urinary tract abnormality, neurologic pathology | NR |
| Sakae 2017; NCCS | Japan  2008-2015 | N = 45 women, 48 babies  Inclusions: all women who had been diagnosed with early-onset severe PE and were treated (diagnosis made according to Japanese criteria (detail provided)  Exclusions: NR | PE | 1: Post-protocol: April 2013 onwards (100% MgSO4 use), N = 17 women, 19 babies  2: Pre-protocol: prior to April 2013 (36% MgSO4 use), N = 28 women, 29 babies  1: > 48 hours MgSO4, N = 17 women, 19 babies  2: ≤ 48 hours MgSO4, N = 10 women, 10 babies  3: No MgSO4, N = 18 women, 19 babies  MgSO4: 4 g IV LD; 1 g/hour IV MD until 24 hours after birth | Composite of serious complications (1 or more of: neonatal death, assisted ventilation with ETT > 24 hours, RDS, PPH, PDA, BPD, cPVL, IVH grade ≥ 3, NEC and sepsis) | Funding: NR  Conflicts: none |
| Salafia 1995; unclear: results appear to be presented as RCS with CCS(N | USA  1988-1993 | N = 406 women and their babies  Inclusions: all women delivering with GA < 32 weeks  Exclusions: stillbirth, fetal congenital anomalies, multiple gestation, maternal diabetes mellitus, chronic hypertension, hydrops fetalis, placenta previa, and elective birth for IUGR | T (4 g IV LD over 20 minutes; 2 g/hour IV MD) | 1: Early GM-IVH (≤ 72 hours), N = 44 babies  2: Late GM-IVH (> 72 hours), N = 21 babies  3: No GM-IVH, N = 341 babies | MgSO4 exposure | NR |
| Sarkar 2009; unclear: results appear to be presented as RCS with CCS(N) | USA  2001-2007 | N = 59 babies  Inclusions: infants with birthweight < 1500 g admitted to the NICU, with severe IVH (grade 3/4) determined by routine cranial sonography (during 1^st^ 7-10 days of life)  Exclusions: NR | NR | 1: IVH grade 3, N = 28 babies  2: IVH grade 4, N = 31 babies | MgSO4 exposure | NR |
| Schanler 1997; PCS | Authors from USA  Time period NR | N = 41 babies  Inclusions: infants born pregnant women between 24-32 weeks GA; 1) preterm labour treated for > 1 week with strict bed rest and IV MgSO4; 2) similar women in whom strict bed rest was ordered for the obstetrical indications of either placenta praevia or preterm labour  Exclusions: systemic illness (diabetes mellitus, chorioamnionitis, medications known to affect calcium metabolism) | T | 1: MgSO4 (6 g IV LD over 30 minutes; 2 g/hour (1.5-3.5 g/hour) IV MD, discontinued in second stage of labour/at caesarean); N = 16 women, 22 babies  2: No MgSO4, N = 15 women, 19 babies | Apgar score < 7 at 5 minutes, LOS (days) (mean ± SD), HMD, PDA, IVH, NEC, birth depression, oxygen treatment, oxygen treatment > 1 month, MV, MV > 1 week, methylxanthine treatment for apnoea | Funding: support from General Clinical Research Center, Baylor College of Medicine/Texas Children’s Hospital Clinical Research Center, NIH; and USDA/ARS.  Conflicts: NR |
| Scudiero 2000; RCS with CCS(N) | USA  1986-1999 | N = 127 babies  Inclusions: infants with birthweights 700-1249 g, born following preterm labour treated with MgSO4 for T  Exclusions: infants born to women with PE or PE superimposed on chronic hypertension, infants with birthweights < 700 g and > 1249 g, fetuses and neonates with major congenital anomalies | T | 1: Fetal or neonatal deaths, N = 18 babies  2: Survivors, N = 109 babies  1: MgSO4 ≤ 24 g, N = 43 babies  2: MgSO4 > 24 but ≤ 48 g, N = 25 babies  3: MgSO4 > 48 g, N = 59 babies | MgSO4 for T > 48 g, and ≤ 48 g vs > 48 g  Death | Funding: supported by University of Chicago  Conflicts: NR |
| Shalabi 2017; RCS | Canada  2011-2014 | N = 4355 babies  Inclusions: infants born between 22-27 weeks GA and admitted to any of the 29 tertiary level neonatal units participating in the CNN  Exclusion criteria: infants with a major congenital anomaly or who were moribund on admission, those who had missing data regarding MgSO4 administration | Any | 1: MgSO4 IV, N = 2055 babies  2: No MgSO4, N = 2300 babies | Apgar score < 7 at 5 minutes, SNAP-II score > 20, MV day 1, prophylactic indomethacin, PDA treated with indomethacin, postnatal steroids for hypotension, postnatal steroid for BPD, PDA treated (indomethacin or ibuprofen), postnatal steroids or PDA treatment, NEC stage II or higher, SIP, NEC or SIP, death prior to discharge, NEC or SIP associated death, IVH grade 3/4 or PVL, ROP stage 3 or above or ROP treated, BPD, nosocomial infection | Funding: no specific funding, CNN coordinating centre supported by CIHR  Conflicts: none |
| Shamsuddin 2005; NRT | Pakistan  2001 | N = 265 women and their babies (207 antepartum/intrapartum PE/E cases)  Inclusions: women with E or severe PE; pregnancy > 28 weeks GA, blood pressure > 140/100 mmHg, urine output > 30 mL/hour and respiratory rate > 16/minute  Exclusions: urine output < 30 mL/hour, absent patellar reflex, respiratory rate < 16/minute | PE/E | 1: MgSO4 LD at home before referral to hospital (4 g IV over 20 minutes and 3 g IM LD in each buttock), N = 102 women and babies  2: No MgSO4 before referral to hospital, N = 105 women and babies | Asphyxia, stillbirth | Funding: WHO  Conflicts: NR |
| Shokry 2010; PCS | Saudi Arabia  2007-2008 | N = 48 women and their babies  Inclusions: singleton pregnancies with risk of preterm labour, intact fetal membranes, no major fetal congenital anomalies and no maternal or fetal complications necessitating immediate delivery, 30-34 weeks GA  Inclusions for ‘controls’: infants born immediately after reaching the hospital, where the mother had a contraindication for tocolysis/MgSO4 but fulfilled other inclusions  Exclusions: women with any significant complications during pregnancy or birth such as PE and those with multiple pregnancies, all infants with perinatal asphyxia, infection, anaemia and polycythaemia | T | 1: MgSO4 (4 g IV LD over 20 minutes; 1-2 g/hour IV MD), N = 28 women and their babies  2: No MgSO4, N = 20 women and their babies | RDS, PV-IVH, seizures, MV, surfactant use, inotropic drug use, PDA, neonatal death | Funding: NR  Conflicts: none |
| Stetson 2019; NCCS  Research Letter | USA  2002-2014 | N = 110 babies  Inclusions: children who received a CP diagnosis, delivered before 32 weeks GA  Exclusions: NR | FN/PE | 1: 2002-2008 (pre-BEAM trial, 36% uptake MgSO4), N = 42 babies  2: 2009-2014 (post-BEAM trial, 62% uptake MgSO4), N = 68 babies | BPD, IVH | Funding: supported by NICHD, Cerebral Palsy Foundation, March of Dimes Prematurity Research Center  Conflicts: none |
| Stockley 2018; RCS | Canada  2010-2011 | N = 336 babies (defined according to fetal standards: estimated fetal weight < 10^th^ centile); or 177 babies (defined according to neonatal standards: actual birthweight < 10^th^ centile)  Inclusions: growth-restricted babies < 28 weeks GA admitted to 1 of the tertiary NICUs participating in the CNN, who were assessed in neurodevelopmental follow up clinics at 18-36 months CA  Exclusions: babies with major congenital or chromosomal anomalies, planned palliative care prior to birth, or with missing data | NR | Growth restriction (fetal standards)  1: Intrapartum MgSO4 exposure, N = 112 babies  2: No MgSO4, N = 224 babies  Growth restriction (neonatal standards)  1: Intrapartum MgSO4 exposure, N = 61 babies  2: No MgSO4, N = 116 babies | Death in NICU and post-discharge, Apgar score < 7 at 5 minutes, chest compression or epinephrine, SNAP-II score > 20, BPD, NEC, late-onset sepsis, ROP stage 3/4/5 or treated, IVH grade 1/2, IVH grade 3/4, | Funding: none (specific for this study)  Conflicts: none |
| Suh 2015; RCS  English abstract; article in Korean | Authors from Korea  2009-2013 | N = 586 babies  N = 150 babies of relevance (excluded normotensive controls)  Inclusions: term infants who were delivered from normotensive and antihypertensive drug ± MgSO4 treated women  Exclusions: CD | HD | 1: Antihypertensive drugs and MgSO4, N = 40 babies  2: Antihypertensive drugs only, N = 110 babies | LOS (days) (mean ± SD), duration of ventilation (days) (mean ± SD), duration of oxygen (days) (mean ± SD), RDS, BPD, moderate to severe, BPD, PDA treated (medication ± operation), ROP treated with laser, NEC, IVH grade ≥ 2, PVL, death | CD |
| Teng 2006; unclear: results appear to be presented as RCS with CCS(N) | Authors from USA  2002-2003 | N = 184 babies  Inclusions: all viable singleton premature infants without lethal anomalies, born between 23-30 weeks GA and were admitted to the NICU  Exclusions: NR | PE/T | 1: Early hypotension, N = 75 babies  2: No early hypotension, N = 109 babies | MgSO4 exposure | NR |
| Verma 2006; unclear: results appear to be presented as RCS with CCS(N) | USA  2000-2001 | N = 45 babies  Inclusions: all ELBW < 1000 g at birth infants admitted consecutively to the NICU  Exclusions: infants suffering chromosomal anomalies, major congenital malformation or any organ system or hydrops fetalis | PE/T | 1: PIE on chest radiograph, N = 11 babies  2: no PIE on chest radiograph, N = 34 babies | MgSO4 dose (g) (mean ± SD), MgSO4 dose ≥ 10 g | Funding: General Clinical Research Grant  Conflicts: NR |
| Weintraub 2001; RCS | Israel  1995-1998 | N = 2794 babies (have not considered the 263 babies and 177 infants exposed to ritodrine and indomethacin)  Inclusions: VLBW newborn infants (birthweight < 1500 g), at GA 24-32 weeks, with a CUS examination during the 1^st^ 24 days of life  Exclusions: death in delivery room, < 24 weeks or > 32 weeks GA, born to mother with PIH, no CUS examination, receipt of combination of tocolytic drugs | T | 1: MgSO4 (≥ 12 hours before birth), N = 341 babies  2: No tocolysis (for ≥12 hours before birth), N = 2013 babies | PVH/IVH grade 3/4 | NR |
| Weisz 2015; RCS | Canada  2011-2012 | N = 6015 babies  Inclusions: infants born 23+0 to 31+6 weeks GA  Exclusions: infants with major congenital anomalies and those who were moribund on admission (i.e. a physician, in consultation with the parents, had made an explicit decision not to provide life support at the time of birth); infants whose MgSO4 exposure status was missing | FN/PE/T/UK | 1: MgSO4 for FN, N = 1387 babies  2: No MgSO4, N = 3868 babies  23-28 weeks GA  1: MgSO4 for FN, N = 731 babies  2: No MgSO4, N = 1813 babies  29-31 weeks GA  1: MgSO4 for FN, N = 656 babies  2: No MgSO4, N = 2055 babies  1: MgSO4 for FN, N = 1387 babies  2: MgSO4 for PE/T = 214 babies  3: MgSO4 for UK = 546 babies  1: MgSO4 for any indication, N = 2147 babies  2: No MgSO4, N = 3868 babies | Any resuscitation (mask CPAP or PPV, ETT intubation and ventilation, chest compressions or epinephrine) , CPAP only, bag/mask or neopuff ventilation, intubation and ventilation, chest compressions, epinephrine (ETT or IV), Apgar score < 7 at 5 minutes, surfactant use, SNAP-II score > 20, intensive resuscitation (intubation and ventilation, or chest compressions or epinephrine administration in delivery room), death, BPD, NEC stage ≥ II, IVH grade 3/4 or PVL, ROP stage ≥ II, sepsis, composite outcome (morality or any major morbidity) | Funding: supported by CIHR and Ontario Ministry of Health and Long-term Care, individual participating hospitals  Conflicts: 1 author supported by CIHR |
| Whitsel 2004; RCS  Abstract | Authors from USA  1997-2002 | N = 118 babies  Inclusions: non-anomalous infants ≤ 1000 g and/or < 28 weeks GA  Exclusions: NR | NR | 1: MgSO4, N = NR  2: No MgSO4, N = NR | Death, late bacterial sepsis | NR |
| Whitten 2015; unclear: results appear to be presented as RCS with CCS(N)  Abstract | USA  2013 | N = 6791 babies  Inclusions: term (> 37 weeks GA) singleton neonates  Exclusions: NR | NR | 1: LOS ≤ 3 days, N = 6472 babies  2: LOS ≥ 4 days, N = 319 babies | MgSO4 exposure | NR |
| Wiswell 1996; PCS  Abstracts (2) | Authors from USA  1991-1994 | N = 137 babies  Inclusions: ventilated preterm infants > 33 weeks GA  Exclusions: NR | PIH/T | 1: MgSO4, N = 61 babies  2: No MgSO4, N = 76 babies | NEC, ICH grade 3/4, cPVL in survivors ≥ 21 days, ICH grade 3/4 or cPVL | Funding: Supported in part by NIH 5RO1 HD21453-06  Conflicts: NR |
| Wutthigate 2017; unclear: results appear to be presented as PCS with CCS(N) | Thailand  2015 | N = 57 women, 63 babies  Inclusions: pregnant women who received intrapartum MgSO4  Exclusions: women with known fetal conditions affecting infant neurological ability, including congenital anomalies and major chromosomal abnormalities, women under general anaesthesia (risk of respiratory depression) | PIH/T (4 g IV LD; 2 g/hour IV MD) | 1: Apnoeic episodes, N = 8 babies  2: No apnoeic episodes, N = 55 babies | MgSO4 dose (reported as mg/dL) (mean ± SD) | Funding: Faculty of Medicine Siriraj Hospital, Mahidol University  Conflicts: none |
| Yokoyama 2010; RCS | Japan  2005-2007 | N = 117 babies  Inclusions for ‘cases’: newborns whose mothers had received IV MgSO4 for > 5 days for T  Inclusions for ‘cases’: newborns whose mothers did not received MgSO4 in same period; matched for GA, birthweight and number of multiple gestations  Exclusions: NR | T | 1: MgSO4 (4 g IV LD over 1 hour; 1-2 g/hour IV MD), N = 58 babies  2: No MgSO4, N = 59 babies | RDS, IVH, PDA, ROP, death, NEC, bone change (osteopenic radiolucent bands at metaphyses of long bones) | NR |
| Young 1977; NRT | USA  1974-1975 | N = 144 women and their babies  Inclusions: women with PE or E based on criteria of the American Committee on Maternal Welfare  Exclusions: NR | PE/E | 1: MgSO4 ‘push’ IV (10 g IM LD and 2 g IV LD over 10 minutes; MD of 2 g IV slow push over 10 minutes every 1-2 hours), N = 97 women and babies  2: MgSO4 continuous IV (10 g IM LD; 1 g/hour IV MD), N = 47 women and babies | Perinatal death | NR |

Abbreviation: ACOG: American College of Obstetricians and Gynecologists; AF: amniotic fluid; AGA: appropriately grown for age; APH: antepartum haemorrhage; ATSDR: Agency for Toxic Substances and Disease Registry; BP: blood pressure; BPD: bronchopulmonary dysplasia; CA: corrected age; CBF: cerebral blood flow; CCS: case-control study; CCS(N): case-control study (nested); CD: cannot determine; CPR: cardiopulmonary resuscitation; CSF: cerebrospinal fluid; DC: Center for Disease Control and Prevention; CIHR: Canadian Institutes of Health Research; CLD: chronic lung disease; CMV: continuous mandatory ventilation; CNN: Canadian Neonatal Network; CNS: central nervous system; CP: cerebral palsy; CPAP: continuous positive airway pressure; cPVL: cystic periventricular leucomalacia; CRIB: clinical risk index for babies; CUS: cranial ultrasound; DA: ductus arteriosus; DHHS: Department of Health & Human Service; DIS: disseminated intravascular coagulation; E: eclampsia; EFM: electronic fetal monitoring; ELBW: extremely low birthweight; ESHP: early and severe hypertension in pregnancy; ET: endotracheal; ETT: endotracheal tube; EU: European Union; FDA: Food and Drug Administration; FGR: fetal growth restriction; FHR: fetal heart rate; FN: fetal neuroprotection; g: grams; GA: gestational age; GH: gestational hypertension; GM-IVH: germinal matrix intraventricular haemorrhage; HD: hypertensive disorders of pregnancy; HFOV: high frequency oscillatory ventilation; HMD: hyaline membrane disease; HsPDA: haemodynamically significant patent ductus arteriosus; HUS: head ultrasound; ICH: intracranial haemorrhage; IDD: insulin-dependent diabetes; IM: intramuscular; IMV: intermittent mandatory ventilation; IQR: interquartile range; IV: intravenous; ITS: interrupted time series; IUGR: intrauterine growth restriction; IVH: intraventricular haemorrhage; LD: loading dose; LOS: length of stage; LSV: lenticulostriate vasculopathy; MD: maintenance dose; MFMU: Maternal Fetal Medicines Unit; Mg: magnesium; MgSO4: magnesium sulphate; mL: millilitres; MPT: moderately preterm; MRI: magnetic resonance imaging; MV: mechanical ventilation; N: number; NBRS: Neurobiologic Risk Scale; NCATS: National Center for Advancing Translational Sciences; NCCS: non-concurrent cohort study; NEC: necrotising enterocolitis; NHLBI: National Heart, Lung, and Blood Institute; NICHD: National Institute of Child Health and Human Development; NICU: neonatal intensive care unit; NINDS: National Institute of Neurological Disorders and Stroke; NINR: National Institute of Nursing Research; NIH: National Institutes of Health; NR: not reported; NRN: Neonatal Research Network; NRT: non-randomised trial; NS: not significant; NSAIDs: non-steroidal anti-inflammatory drugs; PCS: prospective cohort study; PDA: patent ductus arteriosus; PE: pre-eclampsia; PEA: parenchymal echo abnormality; PIE: pulmonary interstitial emphysema; PIH: pregnancy-induced hypertension; PPH: persistent pulmonary hypertension; PPROM: preterm premature rupture of membranes; PROM: premature rupture of membranes; PPV: positive pressure ventilation; PROM: premature rupture of membranes; PV-IVH: periventricular intraventricular haemorrhage; PVL: periventricular haemorrhage; PWML: punctate white matter lesions; RCS: retrospective cohort study; RCT: randomised controlled trial; RD: respiratory distress; RDS: respiratory distress syndrome; ROM: rupture of membranes; ROP: retinopathy of prematurity; SCBU: special care baby unit; SD: standard deviation; SE: standard error; SH: systemic hypertension; SIP: spontaneous intestinal perforation; SMIV: synchronized intermittent mandatory ventilation; SNAP: Score For Neonatal Acute Physiology; SNI: severe neurological injury; sPDA: significant PDA; T: tocolysis; TSV: thalamostriate or mineralising vasculopathy; TTN: transient tachypnoea of the newborn; UK; unknown; USA: United States of America; USDA/ARS: Unites States Department of Agriculture, Agricultural Research Service; VLBW: very low birthweight; WHO: World Health Organization; WMI: white matter injury
