# Supplementary material for: Antenatal magnesium sulphate and adverse neonatal outcomes: A systematic review and meta-analysis
Source: PLoS Med. 2019 Dec 6;16(12):e1002988. doi: 10.1371/journal.pmed.1002988 (PMC6897495; doi:10.1371/journal.pmed.1002988)
Supplement: S2 Table — (DOCX) [file pmed.1002988.s005.docx]

**Risk of bias of included studies – randomised controlled trials**

| **Study** | **Random sequence generation (selection bias)** | **Allocation concealment (selection bias)** | **Blinding of participants and personnel (performance bias)** | **Blinding of outcome assessment (detection bias)** | **Incomplete outcome data (attrition bias)** | **Selective reporting (reporting bias)** |
| --- | --- | --- | --- | --- | --- | --- |
| Abdul 2013 | Low risk: Quote: "computer-generated numbers." | Low risk: Quote: "delivered by sealed opaque envelope." | High risk: Quote: "our trial was not blinded." | High risk: Quote: "our trial was not blinded." | Low risk: No apparent missing outcome data. | Unclear risk: The study protocol is not available. Unclear whether the published report includes all pre-specified outcomes. Limited pre-specification of outcomes in methods of report. |
| Agrawal 2013Abstract | Low risk: Quote: "using a computer generated random table." | Unclear risk: No detail provided (abstract only). | High risk: No blinding (assumed due to nature of intervention and control). | Unclear risk: No detail provided (abstract only). | Unclear risk: No detail provided (abstract only). | Unclear risk: The study protocol is not available. It is unclear whether the published report includes all pre-specified outcomes. Abstract only. |
| Bain 2014 | Low risk: Quote: "The randomisation sequence was computer generated." | Low risk: Quote: "treatment allocated by the telephone randomisation service at the University of Adelaide." | High risk: Quote: "The midwives, who could not be blinded to the treatment group allocation…. Midwives and obstetricians were asked not to discuss treatment group allocation with the women." | High risk: No blinding reported. | Low risk: No apparent missing data. | Low risk: Trial registration (ACTRN12605000765628) available, and outcomes reported as pre-specified. |
| Begum 2002 | Low risk: Quote: "patients were randomly assigned by lottery… we randomly selected a piece of paper from a box to determine if the patient was to receive only loading or both loading and the maintenance schedule." | Unclear risk: No detail provided. | High risk: No blinding (assumed due to nature of intervention and control). | High risk: No blinding reported. | Low risk: No apparent missing data. | Unclear risk: The study protocol is not available. It is unclear whether the published report includes all pre-specified outcomes. No clear pre-specification of outcomes in methods of report. |
| Behrad 2003 | Low risk: Quote: "Patients were randomly assigned by computer-generated random number allocation." | Low risk: Quote: "with consecutively numbered opaque envelopes." | High risk: No blinding (assumed due to nature of intervention and control). | High risk: No blinding reported. | Low risk: No apparent missing data. | Unclear risk: The study protocol is not available. It is unclear whether the published report includes all pre-specified outcomes. Limited pre-specification of outcomes in methods of report. |
| Bhattacharhee 2011 | Low risk: Quote: "computer-generated randomisation protocol." | Low risk: Quote: "the allocation was concealed in sealed, sequentially numbered, brown envelopes, which had been prepared by the statistician at each centre." | High risk: Quote: "Because of the nature of the drug administration, the patients and the doctors responsible for drug administration were not blinded to the randomisation allocation." | High risk: No blinding reported. | Unclear risk: Reason(s) for missing neonatal outcome data not reported (53/67 and 54/70 babies included in analyses). | Unclear risk: The study protocol is not available. It is unclear whether the published report includes all pre-specified outcomes. Secondary outcomes included "maternal and perinatal outcomes." |
| Blackwell 2001 | Unclear risk: Quote: "Randomization was conducted through the hospital pharmacy." | Unclear risk: As above. | Low risk: Quote: "Patients received the study medication in unlabeled intravenous bags, and all clinicians, labor and delivery personnel, and research nurses were blinded to which medication each patient received." | Low risk: As above, and "Placental examination was performed by pathologists blinded to patient study group." | Low risk: No apparent missing data. | Unclear risk: The study protocol is not available. It is unclear whether the published report includes all pre-specified outcomes. Unclear pre-specification of outcomes in methods of report. |
| Charma 2013 | Low risk: Quote: "using a computer generated randomization protocol." | Low risk: Quote: "The allocation was concealed in sealed sequentially numbered brown envelops." | High risk: Quote: "Because of the nature of drug administration, the doctors and nurses responsible for drug administration were not blinded to the randomization allocation." | High risk: No blinding reported. | Low risk: Relatively low proportion of missing data (6/56 and 8/56), balanced between groups. | Unclear risk: The study protocol is not available. It is unclear whether the published report includes all pre-specified outcomes. "Both maternal and perinatal outcomes were recorded." |
| Chen 1995 | Unclear risk: Quote: "were all randomized." | Unclear risk: As above. | High risk: No blinding (no placebo used). | High risk: No blinding reported. | Low risk: No apparent missing data. | Unclear risk: The study protocol is not available. It is unclear whether the published report includes all pre-specified outcomes. "The clinical data, and fetal and maternal complications, and outcome of both groups were analysed." |
| Chissel 1994 | Unclear risk: Quote: "patients were randomly allocated." | Unclear risk: As above. | High risk: No blinding (assumed due to nature of intervention and control). | High risk: No blinding reported. | Low risk: No apparent missing data. | Unclear risk: The study protocol is not available. It is unclear whether the published report includes all pre-specified outcomes. "Clinical outcome... were recorded for both groups." |
| Coetzee 1998 | Unclear risk: Quote: "women were randomised." | Low risk: Quote: "women were allocated using sealed opaque envelopes containing a card instructing the use of solution A or solution B. These cards (but not the envelopes) were consecutively numbered. Envelopes were distributed in mixed batches of 20 and these always had equal numbers of A and B." | Low risk: Placebo used. Quote: "The sterile solutions were prepared by the hospital pharmacy… The identity of the solutions marked A or B were changed periodically by pharmacy without the knowledge of the investigators. The identity of the solutions was revealed only on completion of the study." | Low risk: As above. | Unclear risk: On completion of the study 123/822 random envelopes and data sheets could not be retrieved from patient records, and it was not possible to determine who had been randomised to the treatment and placebo groups – these women were excluded from there study. | Unclear risk: The study protocol is not available. It is unclear whether the published report includes all pre-specified outcomes. Only the primary outcome detailed in the methods of the report. |
| Colon 2015Abstract | Unclear risk: Quote: "were randomized." | Unclear risk: No detail provided (abstract only). | Unclear risk: Saline placebo used (limited detail provided; abstract only). | Unclear risk: No detail provided (abstract only). | Unclear risk: No detail provided (abstract only). | Unclear risk: The study protocol is not available. It is unclear whether the published report includes all pre-specified outcomes. Abstract only. |
| Cotton 1984 | Unclear risk: Quote: "patients were randomized." | Unclear risk: As above. | Low risk: Placebo used. | Unclear risk: No detailed provided. | Low risk: 1 women in the treatment group was lost to follow up and excluded from analyses. | Unclear risk: The study protocol is not available. It is unclear whether the published report includes all pre-specified outcomes. |
| Cox 1990 | Low risk: Quote: "Patients were assigned to treatment or control groups by means of a random number table." | Low risk: Quote: "group allocation predetermined and placed in consecutively numbers and sealed envelopes." | Unclear risk: Saline used in control group; serial magnesium serum level determinations in the treatment group indicate that blinding may not have been achieved. | Unclear risk: No detail provided. | Low risk: No apparent missing data. | Unclear risk: The study protocol is not available. It is unclear whether the published report includes all pre-specified outcomes. Outcomes not clearly pre-specified in methods of report. |
| Crowther 2003 | Low risk: Quote: "The study randomization numbers were generated by computer." | Low risk: Quote: "managed by nonclinical staff at the University of Adelaide’s Maternal Perinatal Clinical Trials Unit… Each study number was placed on a masked treatment pack… Eligible women… were enrolled by taking the next treatment pack." | Low risk: Placebo used. Quote: "All perinatal staff were blinded to treatment group allocation." | Low risk: As above. Quote: "Surviving children were assessed at a corrected age of 2 years by developmental paediatricians and psychologists blinded to treatment group allocation." | Low risk: Outcome data up to discharge available for all 1062 women and 1255 infants alive at randomisation; 2 year corrected age outcomes available for 1047 children (99% of survivors); 14 children (9 in the magnesium group and 5 in the placebo group) did not have 2 year cerebral palsy assessment and were excluded from analyses. | Low risk: The study protocol is not available. It is unclear whether the published report includes all pre-specified outcomes, however methods of report list detailed outcomes which are subsequently reported in the results. |
| Easterling 2018 | Low risk: Quote: “randomisation code based on a computerised pseudo-random number generator.” | Low risk: Quote: “sequentially numbered, sealed, opaque envelope.” | High risk: Quote: “open-label.” | High risk: No blinding reported. | Unclear risk: 99.1% (105/106) neonates from serial IV bolus group analysed (1 had missing data); 90.2% (92/102) neonates from continuous infusion group analysed (2 born to mothers enrolled postpartum, 6 discharged prior to birth, 2 had missing data); small imbalance in exclusions between groups | Unclear risk: The study protocol is not available. Unclear whether the published report includes all pre-specified outcomes. No pre-specification of outcomes in trial registration (NCT02091401). |
| Fox 1993 | Low risk: Quote: "in which the group selection was generated from a table of random numbers." | Unclear risk: Insufficient detail provided. Quote: "The randomization was performed by using the sealed-envelope method… A disinterested third party (the pharmacy) was in charge of selection of the envelope for each patient." | High risk: Quote: "The treating physicians did not have access to the randomization envelopes but were not blinded to group assignment." No placebo used. | High risk: No blinding reported. | Low risk: No apparent missing data. | Unclear risk: The study protocol is not available. It is unclear whether the published report includes all pre-specified outcomes. Methods of report indicate other neonatal outcomes of interest which are not subsequently reported in results. |
| How 1998 | Low risk: Quote: "generated from a table of random numbers." | Unclear risk: Insufficient detail provided. Quote: "sealed envelope." | High risk: No blinding (no placebo used). | High risk: No blinding reported. | Low risk: No apparent missing data. | Unclear risk: The study protocol is not available. It is unclear whether the published report includes all pre-specified outcomes. Outcomes not clearly pre-specified in methods of report. |
| Keepanasseril 2018 | Unclear risk: Insufficient detail provided. Quote: "Randomisation was done by a predetermined schedule that used a block approach… Randomisations schedule and the allocation concealment was done by a person unrelated to the study." | Low risk: Quote: "Resident doctor on duty opened the randomisation schedule placed in sequential opaque envelopes." | High risk: No blinding (due to nature of intervention and control). | High risk: No blinding reported. | Low risk: No apparent missing data. | Unclear risk: The study protocol is not available. It is unclear whether the published report includes all pre-specified outcomes. |
| Lewis 1997 | Low risk: Quote: "Randomization was accomplished by use of a random number table." | Low risk: Quote: "The randomization cards were placed in an opaque envelope that remained sealed until informed consent was obtained after successful tocolysis. The randomization cards were stored in an area away from clinical care." | High risk: No blinding (due to nature of intervention and control). | High risk: No blinding reported. | Unclear risk: 144 women were included in the study, 3 delivered elsewhere and were not included in analyses (not reported from which groups these women were excluded from). Neonatal outcome data does not appear to take into account the 18 sets of twins. | Unclear risk: The study protocol is not available. It is unclear whether the published report includes all pre-specified outcomes. Only primary outcome pre-specified in methods of report. |
| Livingston 2003 | Low risk: Quote: "Computer-generated group assignment was devised by simple randomization sequence." | Low risk: Quote: "Study group assignment was by sealed, consecutively numbered, opaque envelopes." | Low risk: Placebo used. Quote: "All medication was mixed in the pharmacy and labelled “study drug” to maintain allocation concealment." | Low risk: Quote: "data were collected from chart abstraction… Investigators remained blinded during data collection." | Low risk: No apparent missing data. | Unclear risk: The study protocol is not available. It is unclear whether the published report includes all pre-specified outcomes. Outcomes not clearly pre-specified in methods of report. |
| Magpie 2002 | Low risk: Quote: "with an allocation sequence based on a block size of eight, also generated by the Clinical Trial Service Unit." | Low risk: Quote: "Hospitals with reliable access to telephones used a central telephone randomisation service at the Clinical Trial Service Unit, in Oxford... Hospitals without reliable access to telephones used a local pack system." | Low risk: Placebo used. Quote: "The magnesium sulphate and placebo ampoules were identical, and the solutions looked the same." | Low risk: As above. | Low risk: 5 women excluded (2 in each group due to no data; 1 in magnesium sulphate group due to wrong trial); follow up data available for 99.7% women randomised before delivery and 98.6% of babies. | Low risk: The study protocol is not available. It is unclear whether the published report includes all pre-specified outcomes, however methods of report list detailed outcomes which are subsequently reported in the results. |
| Malapaka 2011 | Unclear risk: Quote: "The women were randomly assigned to the groups." | Unclear risk: As above. | High risk: No blinding (assumed due to nature of intervention and control). | High risk: No blinding reported. | Unclear risk: Insufficient detail provided; group numbers (N=72 and N=54) unbalanced. | Unclear risk: The study protocol is not available. It is unclear whether the published report includes all pre-specified outcomes. |
| Marret 2007 | Low risk: Quote: "Randomisation numbers were generated by computer." | Low risk: Quote: "Central telephone randomisation was managed by staff of the NICU." | Low risk: Quote: "The two solutions looked identical so that the women were unaware of whether they received a MgSO4 or placebo solution. Treatment assignment was single blind." | Low risk: As above. Quote: "For all surviving infants, CUS was conducted by a senior neonatologist or radiologist in each centre separately and in a blind manner relative to treatment allocation." | Low risk: 573 women were randomised, 9 women were excluded from the 3 centres that included < 5 women; 564 women were analysed; all fetuses alive at randomisation (magnesium: 352, placebo: 336) included in mortality analyses. | Low risk: Quote: "This study is registered as an International Standard Randomised Controlled Trial, number 00120588." Not able to locate registration, however methods of report list detailed outcomes which are subsequently reported in the results. |
| Mirzamoradi 2014 | High risk: Quasi-randomised. Quote: "those who had an odd code were allocated to the intervention group and others to the control group." | High risk: Quote: "For random allocation, treatment diets (intervention and control groups) were packed in separate packages and coded from 1 to 92 by an expert midwife. When an eligible patient was accepted into the study, another expert interviewed her and a code from 1 or 92 was assigned to questionnaires regardless of medicinal packages coding." | Low risk: Placebo used. Quotes: "None of the research staff were aware of the treatment allocation of patients in order for blinding purposes;" "All steps were considered blinding principles in the control group too." | Low risk: As above. | Low risk: No apparent missing data. | Unclear risk: While the trial registration (IRCT2012091810876N1) is available, this registration was retrospective. Outcomes not well pre-specified in methods of report, e.g. "fetal and maternal complications." |
| Mittendorf 2002 | Low risk: Quote: "a computerized program… was used." | Unclear risk: No detail provided. | Low risk: Placebo used. Quote: "doubly masked." | Low risk: Quote: "The technicians and researchers who processed all biologic specimens were masked to previous and subsequent health outcomes."; "The developmentalist was masked to the antenatal exposure variables." | Low risk: No apparent missing data. | Unclear risk: The study protocol is not available. It is unclear whether the published report includes all pre-specified outcomes. |
| Moodley 1994 | Unclear risk: Insufficient detail provided. Quote: "Patients were randomly distributed." | Low risk: Quote: "randomly distributed… using the next of a set of consecutively numbered, sealed, opaque envelopes." | High risk: No blinding (no placebo used). | High risk: No blinding reported. | Low risk: No apparent missing data. | Unclear risk: The study protocol is not available. It is unclear whether the published report includes all pre-specified outcomes. Outcomes not well pre-specified in the methods of report: "Main Outcome Measure: The onset of convulsions and both maternal and fetal complications between the groups." |
| Mundle 2012 | Low risk: Quote: "a randomization sequence generated by computer with blocks of 10." | Low risk: Quote: "consecutive opaque envelopes." | High risk: Quote: "However, it was not possible to blind women and providers to the treatment." | High risk: No blinding reported. | Low risk: No apparent missing data. | Unclear risk: The study protocol is not available. It is unclear whether the published report includes all pre-specified outcomes; methods of report details additional neonatal outcomes which are not subsequently reported in the results. |
| Orji 2012Abstract | Unclear risk: Insufficient data provided. Quote: "randomized." | Unclear risk: No detail provided (abstract only). | Unclear risk: Quote: "single blind." Unclear who was blinded. | Unclear risk: As above. | Unclear risk: No detail provided (abstract only). | Unclear risk: The study protocol is not available. It is unclear whether the published report includes all pre-specified outcomes. Abstract only. |
| Parashi 2017 | Low risk: Quote: "by means of a computer-generated randomization list." | Low risk: Quote: "by using sealed opaque medication packets that numbers and used consecutively." | Low risk: Placebo used. | Low risk: Quotes: "double-blind"; "they all were assessed with ultrasonography by an experienced radiologist who was blind about the groups." | Low risk: No apparent losses. | Unclear risk: While the trial registration (IRCT2016080729223N1) is available, this registration was retrospective, and no clear pre-specification of outcomes in methods of report. |
| Pascoal 2019 | Low risk: Quote: “randomization list was prepared using the Random Allocation software program, version 1.0.” | Low risk: Quote: “The numbered envelopes were sent to the high-risk unit and to the intensive care unit where the women were consecutively assigned to one of the maintenance regimens. The numbered envelope containing the ampoules was only opened at the time of preparation of the maintenance dose of magnesium sulfate.” | Low risk: Quotes: “The pharmacist received the randomization list of numbers… defining whether the patient would be in the 1-gram/hour or 2-grams/hour group. The pharmacist then prepared ampoules with distilled water for the 1-gram/hour group, and ampoules with a total of 6 grams of magnesium sulfate for the 2-grams/hour group. Both sets of ampoules were identical in color and size. Only the pharmacist was aware of the contents of the ampoules;” and “Throughout the entire study, the investigators, the attending physicians, and the patients remained unaware of the group to which the patient had been allocated.” | Low risk: Quote: “Throughout the entire study, the investigators, the attending physicians, and the patients remained unaware of the group to which the patient had been allocated.” | Low risk: No apparent missing data. | High risk: Trial was terminated early due to poor recruitment; outcomes not reported as pre-specified in original registration (NCT02396030). |
| Rimal 2017 | Unclear risk: Quote: "The participants were randomized." | Unclear risk: No detail provided. | High risk: No blinding (assumed due to nature of intervention and control). | High risk: No blinding reported. | Low risk: No apparent missing data. | Unclear risk: The study protocol is not available. It is unclear whether the published report includes all pre-specified outcomes. No clear pre-specification of outcomes in methods of report. |
| Rouse 2008 | Low risk: Quote: "Group assignment was made according to a computer-generated random sequence." | Unclear risk: No detail provided. | Low risk: Placebo used. | Low risk: As above. Quote: "double-blind." | Low risk: 9/1096 and 4/1145 women in the magnesium and placebo groups were lost to follow up before delivery; thus 1087/1096 and 1141/1145 women were included in maternal analyses; all live born infants included in neonatal analyses; 1133/1188 and 1203/1256 fetuses/children included in primary outcome in the magnesium and placebo groups. | Low risk: While the trial registration (NCT00014989) is available, this registration was retrospective, however methods of report list detailed outcomes which are subsequently reported in the results. |
| Saha 2017 | Low risk: Quote: "according to computer generation." | Unclear risk: No detail provided. | High risk: No blinding (assumed due to nature of intervention and control). | High risk: No blinding reported. | Low risk: No apparent missing data. | Unclear risk: While the trial registration (CTRI/ 2009 000339, 05–08-2009) is available, this registration appears to have been retrospective; need for calcium gluconate detailed in methods and trial registration as an outcome of interest but not subsequently reported. |
| Shilva 2007 | Low risk: Quote: "the patients were randomized using a Tippet table." | Unclear risk: No detail provided (short report). | High risk: No blinding (assumed due to nature of intervention and control). | High risk: No blinding reported. | Low risk: No apparent missing data. | Unclear risk: The study protocol is not available. It is unclear whether the published report includes all pre-specified outcomes. Outcomes not clearly pre-specified in short report: "compared for maternal and neonatal outcome." |
| Shreya 2014 | High risk: Quote: "Randomisation was done by giving above regimen alternatively." | High risk: As above. | High risk: No blinding (assumed due to nature of intervention and control). | High risk: No blinding reported. | Unclear risk: No apparent missing data. | Unclear risk: The study protocol is not available. It is unclear whether the published report includes all pre-specified outcomes. Outcomes not clearly pre-specified in short report: "Maternal and fetal complications." |
| Singh 2011 | Low risk: Quote: "Patients were randomly allocated by means of a random number generator." | Unclear risk: No detail provided. | High risk: No blinding (assumed due to nature of interventions and control). | High risk: No blinding reported. | Low risk: No apparent missing data. | Unclear risk: The study protocol is not available. It is unclear whether the published report includes all pre-specified outcomes. Outcomes not clearly pre-specified in methods, "maternal mortality and morbidity and perinatal mortality and morbidity." |
| Tangmanowutthikul 2019 | Low risk: Quote: “using block randomisation by computer generated random number.” | Low risk: Quote: “sealed in opaque envelops.” | High risk: No blinding (assumed due to nature of intervention and control). | High risk: No blinding reported. | Low risk: No apparent missing data (unclear as to whether 1 and 2 neonates are missing from NICU admission data, based on percentages reported). | Unclear risk: The study protocol is not available. Unclear whether the published report includes all pre-specified outcomes. No pre-specification of outcomes in trial registration (TCTR20180122001). |
| Terrone 2000 | Low risk: Quote: "computer-generated random number allocation." | Low risk: Quote: "consecutively numbered opaque envelopes." | High risk: No blinding (assumed due to nature of intervention and control). | High risk: No blinding reported. | Unclear risk: Quote: "Patients who were considered to have treatment failure were excluded from further analyses because the time to tocolysis could not be assessed"; 148/160 women were included in analyses. | Unclear risk: The study protocol is not available. It is unclear whether the published report includes all pre-specified outcomes. |
| Witlin 1997 | Low risk: Quote: "Randomization was performed by the use of computer-generated tables of random numbers." | Low risk: Quote: "sealed, sequentially numbered, opaque envelopes." | Low risk: Placebo used. Quote: "Women randomized to placebo infusion received saline solution that was identical in appearance to the magnesium sulfate infusion and was likewise prepared in and dispensed by the hospital pharmacy." | Low risk: As above. | Low risk: No apparent missing data. | Unclear risk: The study protocol is not available. It is unclear whether the published report includes all pre-specified outcomes. |

**Risk of bias of included studies – non-randomised studies**

| **Study** | **Were valid and reliable measures implemented consistently? (detection bias; confounding)** | **Any attempt to balance the allocation between the groups or match groups? (confounding)** | **Were important confounding variables not taken into account? (confounding)** | **Do the inclusion and/or exclusion criteria vary across groups? (selection bias)**  **Does the strategy for recruiting participants differ across groups? (selection bias; confounding)**  **Is the selection of the comparison group inappropriate? (selection bias; confounding)** | | **Does the study fail to account for important variations from the proposed protocol? (performance bias)** | | **Was the outcome assessor not blinded? (detection bias)** | | **Was the length of follow-up different across groups? (attrition bias)**  **In cases of high or differential loss to follow-up, was the impact assessed? (attrition bias; detection bias)** | | **Are any important primary outcomes missing from the results? (selective outcome reporting)**  **Are any important harms missing from the results? (selective outcome reporting)** | **Overall risk of bias** |
| --- | --- | --- | --- | --- | --- | --- | --- | --- | --- | --- | --- | --- | --- |
| Adama-Hondegla 2013 | Cannot determine: retrospective file review; limited detail/definitions provided | Cannot determine | Cannot determine | Not further assessed, although study reports that logistic regression was used, and aORs were presented in tables, no detail was provided of variables adjusted for | | | | | | | | | High |
| Alexander 2006 | Yes: prospective data collection with use of database (verified accuracy); though outcomes not clearly pre-defined in methods | No | Yes | Not further assessed; no adjustment for confounders | | | | | | | | | High |
| Alston 2016 | Cannot determine: retrospective record review; limited detail/definitions provided | Yes | Yes | Not further assessed, although study reports a multivariable logistic regression model was used (considering variables found to differ in univariate comparisons), for the review outcomes of interest, there was no adjustment for confounders | | | | | | | | | High |
| Ambadkar 2017 | Cannot determine: while neonatologists were reported to be ‘blinded’ to study, unclear if this was feasible and limited detail/definitions provided | Cannot determine, reported to be “matched” | Yes | Not further assessed; no adjustment for confounders | | | | | | | | | High |
| Bajaj 2018 | Yes: prospectively collected data by trained research personnel using standard definitions | Yes | Partially: multivariable logistic regression analysis performed to assess the association between levels of resuscitation with selected morbidities after adjusting for centre, GA, SGA status, any antenatal steroids, and multiple birth | No | | Cannot determine | | Yes, not blinded | | No | | No | Moderate |
| Basu 2012 | Yes: retrospective chart review, though detail/definitions provided | Yes | Partially: adjustment for GA, birthweight and multiple gestations (for PDA, ROP and LOS only) | No | | Cannot determine | | Yes, not blinded | | No | | No (though results of multivariate analysis for PDA, ROP and LOS were incompletely reported) | Moderate to high |
| Belden 2017 | Yes: retrospective chart review, though detail/definitions provided | Yes | Partially: results of multivariate logistic regression incompletely reported | No | | Cannot determine | | Yes, not blinded | | No | | No | Moderate to high |
| Bertello Grecco 2019  Abstract | Cannot determine | Cannot determine | Cannot determine | Not further assessed; abstract only | | | | | | | | | Unclear |
| Black 2006 | Yes: prospective outcome collection, detail/definitions provided | No | Yes | Not further assessed; no adjustment for confounders | | | | | | | | | High |
| Blackwell 2002 | Yes: prospective outcome collection, detail/definitions provided | No | Yes | Not further assessed; no adjustment for confounders | | | | | | | | | High |
| Bonta 2000  Abstract | Cannot determine | Cannot determine | Cannot determine | Not further assessed; abstract only | | | | | | | | | Unclear |
| Bozkurt 2016 | Cannot determine: retrospective record review, no detail/definitions for outcomes of interest | Yes | Yes | Not further assessed, although results of multinomial logistic regression analysis are reported for cerebral palsy; no adjustment for review outcomes of interest | | | | | | | | | High |
| Boyle 2018  Abstract | Cannot determine | Cannot determine | Cannot determine | Not further assessed; abstract only (though logistic regression was used for univariate and multivariable analyses for the composite outcome) | | | | | | | | | Unclear |
| Brazy 1982 | Yes: retrospective collection, though detail/definitions provided | Yes: controls matched based on GA and birth order | Yes | Not further assessed; no adjustment for confounders | | | | | | | | | High |
| Brookfield 2015  Abstract | Cannot determine | Cannot determine | Cannot determine | Not further assessed; abstract only (does report that multivariate techniques were used, controlling for GA, mode of anaesthesia, MgSO4 indication, total dose of MgSO4 and infant sex) | | | | | | | | | Unclear |
| Brookfield 2016*  Abstract | Cannot determine | Cannot determine | Cannot determine | Not further assessed; abstract only (does report that multivariate logistic regression adjusted for diabetes, delivery route, other tocolytics, and GA at birth) | | | | | | | | | Unclear |
| Brown 2019  Abstract | Cannot determine | Cannot determine | Cannot determine | Not further assessed; abstract only (though controls were matched, and aORs, from logistic regression presented) | | | | | | | | | Unclear |
| Canterino 1999 | Yes: retrospective collection, though detail/definitions provided | Yes | Partially: adjustment for GA, birthweight, antenatal steroids, chorioamnionitis, mode of birth, Apgar score < 7 at 5 minutes, RDS; or ‘clinical group’ (for abnormal sonograms, severe lesions only) | No | | Cannot determine | | No, blinded assessment by radiologist | | No | | No | Moderate |
| Cawyer 2016  Abstract | Cannot determine | Cannot determine | Cannot determine | Not further assessed; abstract only (though reports multivariable logistic regression was used to adjust for confounding) | | | | | | | | | Unclear |
| Cho 2014  Abstract | Cannot determine | Cannot determine | Cannot determine | Not further assessed; abstract only (does report that a multiple linear regression model was used) | | | | | | | | | Unclear |
| Chowdhury 2009 | Cannot determine: prospective collection, though limited detail/definitions provided (and some data self-reported by women who were discharged undelivered) | No | Yes | Not further assessed; no adjustment for confounders | | | | | | | | | High |
| Chun 2014  English abstract | Cannot determine | Cannot determine | Cannot determine | Not further assessed; English abstract only (article in Korean) | | | | | | | | | Unclear |
| Cuff 2018 | Yes: abstracted from PINS database (validated research database) | Yes | Partially: binary logistic regression controlling for race, PTL, gestational age, corticosteroid (betamethasone or dexamethasone), birthweight, and indomethacin exposure, to control for potential confounding and co-linear variables, for IVH only | No | | Cannot determine | | Yes, not blinded | | No | | No | Moderate to high |
| Das 2015 | Yes: prospective collection, with detail/definitions provided | No | Yes | Not further assessed; no adjustment for confounders | | | | | | | | | High |
| Deering 2005 | Yes: retrospective database review, with detail/definitions provided | Yes | Partially: adjustment for GA, birthweight, chorioamnionitis and steroid use | No | | Cannot determine | | Yes, not blinded | | No | | No | Moderate to high |
| De Jesus 2015 | Yes: use of prospectively collected database, (limited) detail/definitions provided | Yes | Partially: adjustment for centre, GA, antenatal steroids and PIH/E (for delivery room intubation, day 1 MV, day 1 ET MV, day 3 MV, day 3 ET MV, hypotension and PDA treated) | No | | Cannot determine | | Yes, not blinded | | No | | No | Moderate |
| del Moral 2007 | Cannot determine: prospective collection from maternal/newborn records using standardised forms; PDA only assessed for confirmation when suspected clinically (and no detail/definitions for IVH, PVL) | Yes | Partially: adjustment for GA or birthweight, race, gender, mode of birth, antenatal steroids, presence of chorioamnionitis, MgSO4 indication (for PDA only) | No | | Cannot determine | | Yes, not blinded | | No | | No | Moderate to high |
| delValle 1998  Abstract | Cannot determine | Cannot determine | Cannot determine | Not further assessed; abstract only | | | | | | | | | Unclear |
| Derks 2016  Abstract | Cannot determine | Cannot determine | Cannot determine | Not further assessed; abstract only | | | | | | | | | Unclear |
| De Silva 2018 | Yes: use of prospectively collected database, detail/definitions provided | Yes | Partially: adjustment for multiple gestation, gender, GA at birth, birthweight < 10^th^ centile, outborn status, mode of birth, antenatal corticosteroid use) | No | | Cannot determine | | Yes, not blinded | | No | | No | Moderate |
| de Veciana 1995 | Cannot determine: retrospective chart review, limited detail/definitions provided (e.g. for IVH, NEC) | No | Yes | Not further assessed; no adjustment for confounders | | | | | | | | | High |
| Downey 2017 | Yes: use of database (based on EMRs), (limited) detail/definitions provided | Yes | Partially: adjustment for site, GA at birth, multiple gestation, antenatal steroids, antibiotics, prolonged ROM, SGA, sex, discharge year, postnatal hydrocortisone, postnatal indomethacin) | No | | Cannot determine | | Yes, not blinded | | No | | No | Moderate |
| Drassinower 2015*  Abstract | Cannot determine | Cannot determine | Cannot determine | Not further assessed; abstract only (does report that log-linear regression was used to control for potential confounders, and two outcomes (composite, intubation) reported to be adjusted for GA at birth, sepsis, SGA and alcohol use) | | | | | | | | | Unclear |
| Duffy 2012 | Yes: retrospective collection from ERMs, detail/definitions provided | Yes | Yes | Not further assessed; although study reported multivariable logistic regression analyses, there was no adjustment for review outcomes of interest | | | | | | | | | High |
| Edwards 2018* | Cannot determine: prospective data collection in original RCT, with detail/definitions provided; however, authors acknowledge subjectivity of definition used for chorioamnionitis to define groups | Yes | Partially: adjustment for sex only | No | | Cannot determine | | No, original RCT was blinded | | No | | No | Moderate to high |
| Elimian 2002 | Yes: retrospective chart review, detail/definitions provided | Yes | Partially: adjustment for antenatal confounding variables – assumed to be GA ≤ 28 weeks, antibiotics, antenatal steroids, and chorioamnionitis (for neonatal death only) | No | | Cannot determine | | Yes, blinding of steroid exposure for neurosonograms only | | No | | No | Moderate to high |
| Elliot 2003  Abstract | Cannot determine | Cannot determine | Cannot determine | Not further assessed; abstract only (does report that multivariate analysis corrected for mode of delivery and exposure to antenatal steroids) | | | | | | | | | Unclear |
| Farkouh 2001 | Yes: retrospective analysis using prospectively collected database (monitored for accuracy), though limited detail/definitions provided | Yes | Partially: controlled for GA and indication for therapy, antenatal steroids, terbutaline use, bleeding, caesarean section) | No | | Cannot determine | | Yes, not blinded | | No | | No | Moderate to high |
| FineSmith 1997 | Yes: retrospective record review, with blinded re-review of CUS | Yes: controls matched for GA range only | Partially: logistic regression included GA, MgSO4, Apgar scores at 1 and 5 minutes, duration of intubation, reason for prematurity and type of birth) | Yes (CCS); no; no | | Cannot determine | | No, radiologist blinded to exposure/s | | No | | No | Moderate to high |
| Gano 2016 | Yes: prospective data collection, with detail/definitions provided | Yes | Partially: adjustment for postmenstrual age at MRI, VLBW, intubation at birth, prolonged MV, hypotension, symptomatic PDA, prenatal steroids) | No | | Cannot determine | | No, MRI review blinded to clinical history; and case note review blinded to MRI findings | | No | | No | Moderate |
| Garcia Alonso 2018 | Yes: prospective data collection, detail/definitions provided | Yes: controls matched by GA and time period | Partially: for some outcomes (resuscitation, surfactant, BPD, ROP) control for GA and birthweight) | Yes; no; no | | Cannot determine | | Yes, not blinded | | No | | No (though results for some outcomes incompletely reported (e.g. P = NS; and “no longer statistically significant”) | Moderate to high |
| Gasparyan 2017  English abstract | Cannot determine | Cannot determine | Cannot determine | Not further assessed; English abstract only (article in Russian) | | | | | | | | | Unclear |
| Ghidini 2001 | Yes: retrospective chart review, detail/definitions provided | Yes: controls matched by GA (similar) and gender | Partially: control for diagnosis of preterm labour only | Yes; no; no | | Cannot determine | | Yes, not blinded | | No | | No | Moderate to high |
| Gibbins 2013 | Cannot determine: retrospective record review using standardised forms, however detail/definitions for some outcomes (e.g. “individual morbidities”) lacking | No | Yes | Not further assessed; no adjustment for confounding | | | | | | | | | High |
| Girsen 2015 | Yes: retrospective review using prospectively collected database, with detail/definitions provided | Yes | Partially: adjustment for receipt of public insurance, maternal age, race type of birth, birthweight, GA at birth (for NICU admission and NICU admission ≥ 8 days) | No | | Cannot determine | | Yes, not blinded | | No | | No | Moderate to high |
| Gonzalez-Quintero 2001 Abstract | Cannot determine | Cannot determine | Cannot determine | Not further assessed; abstract only (does report that logistic regression was used) | | | | | | | | | Unclear |
| Greenberg 2011 | Yes: retrospective chart review, with detail/definitions provided | Yes | Partially: controlled for GA, Apgar score at 1 minute, birthweight, caesarean birth, severe PE | No | | Cannot determine | | Yes, not blinded | | No | | No | Moderate to high |
| Greenberg 2013 | Yes: retrospective chart review, with detail/definitions provided | Yes | Partially: controlled for GA, public insurance, birthweight, caesarean birth, chronic hypertension and severe PE (for NICU admission only) | No | | Cannot determine | | Yes | | No | | No | Moderate to high |
| Grether 1998 | Yes: retrospective review, with detail/definitions provided | Yes: controls matched for birthweight range, birth years and counties | Partially: below factors adjusted for separately (while controlling for birthweight, GA): placental infection definite, placental infection definite or possible, infection, sex, maternal age, level of hospital care, maternal bleeding, presentation at birth, surgical birth, exposure to in utero corticosteroid, abruptio placentae, placenta praevia, hypertension | Yes; no; no | | Cannot determine | | Yes, not blinded (control data collected without knowledge of status; cases data collected with no blinding of case status) | | No (though records complete for 85% cases, 90% controls; and additional cases excluded, leaving 62% for analysis) | | No | Moderate to high |
| Grimbly 2015  Abstract | Cannot determine | Cannot determine | Cannot determine | Not further assessed; abstract only | | | | | | | | | Unclear |
| Gulcan 2006 | Cannot determine: prospective collection, though some outcomes lacking detail/definitions (e.g. RDS) | Yes: GA matching for some outcomes | Yes | Not further assessed; no control for confounders for review outcomes of interest | | | | | | | | | High |
| Gursoy 2015 | Yes: prospective collection, with detail/definitions provided | Yes: matched for birthweight and GA | Yes | Not further assessed; no control for confounders | | | | | | | | | High |
| Havranek 2011 | Cannot determine: limited detail/definitions provided re: clinical outcome collection methods | Yes | Yes | Not further assessed; though linear regression analysis was used for superior mesenteric artery blood flow velocity, there was no adjustment for confounders for review outcomes of interest | | | | | | | | | High |
| Hechtman 2002  Abstract | Cannot determine | Cannot determine | Cannot determine | Not further assessed; abstract only (does report that multiple stepwise logistic regression was used, and reports results controlling for GA, betamethasone therapy, clinical chorioamnionitis, and delivery mode) | | | | | | | | | Unclear |
| Holcomb 1991 | Cannot determine: categories for defining chest radiographs not well defined | Yes: matched for single/multiple gestation and GA | Yes | Not further assessed; no control for confounders | | | | | | | | | High |
| Hom 2018  Abstract | Cannot determine | Cannot determine | Cannot determine | Not further assessed; abstract only | | | | | | | | | Unclear |
| Hong 2019  Abstract | Cannot determine | Cannot determine | Cannot determine | Not further assessed; abstract only | | | | | | | | | Unclear |
| Igarashi 1995  English abstract | Cannot determine | Cannot determine | Cannot determine | Not further assessed; English abstract only (article in Japanese) | | | | | | | | | Unclear |
| Imamoglu 2014 | Yes: prospective collection, with detail/definitions provided | No | Yes | Not further assessed; no control for confounders | | | | | | | | | High |
| James 2015 | Yes: prospective collection, with detail/definitions (some limited) provided | Yes: matching for birthweight, GA, and mode of birth | Yes/Partially | Not further assessed; logistic regression used for CLD only and controlled only for antenatal steroid exposure; no control for confounders for other review outcomes | | | | | | | | | High |
| Jazayeri 2003 | Yes: retrospective chart review, though detail/definitions provided | Yes: matching based on PPROM at same GA | Yes | Not further assessed; no control for confounders for review outcomes of interest | | | | | | | | | High |
| Jeanneteau 2014  Abstract | Cannot determine | Cannot determine | Cannot determine | Not further assessed; abstract only | | | | | | | | | Unclear |
| Jones 2018  Abstract | Cannot determine | Cannot determine | Cannot determine | Not further assessed; abstract only | | | | | | | | | Unclear |
| Jung 2018 | Cannot determine: retrospective record review, detail/definitions (somewhat limited) provided | No | Yes | Not further assessed: no control for confounders | | | | | | | | | High |
| Kamilya 2005 | Cannot determine: retrospective review; very limited detail | No | Yes | Not further assessed: no control for confounders | | | | | | | | | High |
| Kamyar 2015a  Abstract | Cannot determine | Cannot determine | Cannot determine | Not further assessed; abstract only (does report the use of multivariable models adjusting for GA, betamethasone exposure, mode of delivery, nulliparity, and PE) | | | | | | | | | Unclear |
| Kamyar 2015b  Abstract | Cannot determine | Cannot determine | Cannot determine | Not further assessed; abstract only (does report the use of a multivariable logistic regression model that included GA, betamethasone exposure, and nulliparity) | | | | | | | | | Unclear |
| Kamyar 2015c*  Abstract | Cannot determine | Cannot determine | Cannot determine | Not further assessed; abstract only (does report the use of multivariable models that included GA as a covariate) | | | | | | | | | Unclear |
| Kamyar 2016a* | Yes: prospective data collection in original RCT, detail/definitions provided; however, authors acknowledge subjectivity of definition used for chorioamnionitis to define inclusion | Yes | Partially: adjusted for GA at birth, maternal years of education, maternal race, IUGR, illicit drug use, smoking status, sex | No | | Cannot determine | | No, original RCT was blinded | | No | | No | Moderate to high |
| Kamyar 2016b* | Yes: prospective data collection in original RCT, detail/definitions provided (though recognition of lack of SIP data, and possibility of diagnostic overlap) | Yes | Partially: adjustment for birth GA, treatment group, fetal sex, SGA, chorioamnionitis, caesarean section, hypotension during initial resuscitation, postnatal exposure to indomethacin, sepsis, IVH | No | | Cannot determine | | No, original RCT was blinded | | No | | No | Moderate to high |
| Katayama 2011 | Yes: retrospective chart review, though detail/definitions provided | Yes | Partially: adjusted for confounders “including” antenatal steroids, ritodrine tocolysis, PROM | No | | Cannot determine | | Yes, not blinded | | No | | No | Moderate to high |
| Kelly 1992  Abstract | Cannot determine | Cannot determine | Cannot determine | Not further assessed; abstract only | | | | | | | | | Unclear |
| Khodapanahandeh 2008 | Yes: retrospective record review, though detail/definitions provided | Yes: controls also VLBW | Partially: multivariate analysis, included factors significant in univariate analyses: GA, birthweight, Apgar score at 5 minutes, resuscitation, tocolytic therapy, apnoea, MV, HMD, haematocrit, PaCO2 maximum in 1^st^ 3 days, symptomatic hypotension 1^st^ 3 days | Yes; no; no | | Cannot determine | | Yes, not blinded | | No | | No | Moderate to high |
| Kimberlin 1998 | Yes: retrospective review of prospectively collected data, with detail/definitions provided | Yes | Partially: controlled for birthweight, GA, race, gender, mode of birth, chorioamnionitis, surfactant treatment, antenatal steroids) | No | | Cannot determine | | Yes, not blinded | | No | | No | Moderate to high |
| Koksal 2002 | Yes: prospective collection with detail/definitions provided (though note: maternal interviews were used in conjunction with chart reviews) | No | Yes | Not further assessed: no control for confounders | | | | | | | | | High |
| Kuban 1992 | Yes: prospective collection with detail/definitions provided (though note: maternal interviews were used in conjunction with chart/pharmacy sheet reviews) | Yes | Partially: stepwise logistic regression analysis included PE related variables; covariates (mode of birth, labour, birthweight, GA, intubation, lowest pH, antenatal steroids, mother’s weight/height were allowed to compete | No | | Cannot determine | | No, assessment of CUS blinded to exposures | | No | | No | Moderate to high |
| Lai 2017  Abstract | Cannot determine | Cannot determine | Cannot determine | Not further assessed; abstract only ( does report that multinomial logistic regression was used) | | | | | | | | | Unclear |
| Lee 2013  English abstract | Cannot determine | Cannot determine | Cannot determine | Not further assessed; English abstract only (article in Korean; does present ORs adjusted for GA) | | | | | | | | | Unclear |
| Lee 2015  Abstract | Cannot determine | Cannot determine | Cannot determine | Not further assessed; abstract only (does report that a multiple linear regression model was used) | | | | | | | | | Unclear |
| Leung 2016 | Yes: prospective enrolment and retrospective record review, with detail/definitions provided | Yes | Partially: models included: GA, birthweight, Apgar scores at 1 and 5 minutes, antenatal exposure to betamethasone, MgSO4, an interaction term, maternal antibiotics, surfactant, CUS abnormalities, duration of ventilation, CLD, duration of furosemide, gentamicin and amphotericin, FIRS, PDA treatment with indomethacin) | No | Cannot determine | | Yes, not blinded for review outcomes (blinding for placental histology) | | No | | No | | Moderate |
| Leviton 1997 | Yes: prospective collection, with definition provided (though note: maternal interview and record review used for some measures) | Yes | Partially: adjusted for GA, birthweight z score, antenatal corticosteroids, PE, PIH, route of birth and labour | No | Cannot determine | | Yes, not blinded | | No | | No | | Moderate to high |
| Lipsitz 1971 | Cannot determine: prospective collection, however limited detail/definitions provided (particularly in methods) | No | Yes | Not further assessed: no control for confounders | | | | | | | | | High |
| Lloreda-Garcia 2016  English abstract | Cannot determine | Cannot determine | Cannot determine | Not further assessed; English abstract only (article in Spanish) | | | | | | | | | Unclear |
| Martin 1998 | Yes: retrospective review, though detail/definitions provided | Yes: matched by time period and GA range | Yes | Not further assessed; no control for confounders | | | | | | | | | High |
| Matsuda 1997 | Yes: retrospective review, though detail/definitions provided | No: controls born in same period only | Yes | Not further assessed; no control for confounders | | | | | | | | | High |
| McGuiness 1980 | Cannot determine: prospective collection, though outcomes not well defined in methods | Yes: controls in same GA range and also appropriately grown | Yes | Not further assessed; no control for confounders | | | | | | | | | High |
| McPherson 2014* | Yes: prospective data collection in original RCT, with detail/definitions provided | Yes | Yes | Not further assessed; no control for confounders for review outcomes of interest (multivariable regression analyses adjusted for confounders for CP/death assessment only) | | | | | | | | | High |
| Mikhael 2019 | Cannot determine: methods for data collection not clearly reported | Yes | Partially: multivariate logistic regression analysis adjusted for significantly different baseline characteristics (maternal hypertension, antenatal steroids, antenatal indomethacin) for composite outcomes only | No | | Cannot determine | | Yes, not blinded | | No | | No | Moderate to high |
| Mitani 2011 | Yes: retrospective records review, with detail/definitions provided | Yes | Partially: reports that multivariate analyses/logistic regression used – assumed adjustment for GA on admission, corticosteroid, GA at birth, birthweight, Apgar scores at 1 and 5 minutes (for composite adverse outcome only) | No | | Cannot determine | | Yes (blinding for CP assessment, not review outcomes) | | No | | No | Moderate to high |
| Mittendorf 2005*  Abstract | Cannot determine | Cannot determine | Cannot determine | Not further assessed; abstract only (does report that there was adjustment for confounding) | | | | | | | | | Unclear |
| Mittendorf 2009*  Abstract | Cannot determine | Cannot determine | Cannot determine | Not further assessed; abstract only (does report that a logistic model controlling for birthweight and funisitis was used) | | | | | | | | | Unclear |
| Morag 2015 | Cannot determine/no: retrospective review of prospective data, detail/definitions clear for ‘cases’; data for term ‘controls’ collected from records | Yes: matched for birth date (within 2 weeks), gender, mode of birth | Partially: reports significant factors from multivariate analysis: maternal age, primiparity, antenatal steroids, SGA, caesarean birth, MgSO4 treatment | Yes; no; no/cannot determine | | Cannot determine | | Yes, not blinded | | No | | No | Moderate to high |
| Morag 2016 | Yes: retrospective chart review, though detail/definitions provided | Yes | Yes | Not further assessed; though multiple linear regression analysis was conducted for neurodevelopmental follow up outcomes, no control for confounders for review outcomes of interest; additionally, protocol deviation in defining groups for comparison: “The mean iMgC was used as a cut-off in exposed infants as a second option because a comparison between those with normal serum concentrations, that is, 1.9 to 2.7 mg/dL, and those with elevated concentrations >2.7 did not reveal any difference between the groups.” | | | | | | | | | High |
| Moschos 2011  Abstract | Cannot determine | Cannot determine | Cannot determine | Not further assessed; abstract only (does report the results of logistic regression analysis) | | | | | | | | | Unclear |
| Murata 2005 | Yes: retrospective record review, though detail/definitions provided | Yes | Partially: multivariate analysis included: Apgar score < 5 at 5 minutes, cord length > 40 cm, indomethacin exposure, birthweight and GA | No | | Cannot determine | | Yes (only placental assessment blinded) | | No | | No | Moderate to high |
| Nakamura 1991  Abstract | Cannot determine | Cannot determine | Cannot determine | Not further assessed; abstract only | | | | | | | | | Unclear |
| Narasimhulu 2017 | Cannot determine: retrospective chart review, limited detail/definitions provided (some outcomes not pre-defined) | Yes | Yes | Not further assessed; for our comparison of interest, no control for confounding (though for comparisons of neonatal serum magnesium concentrations, logistic regression was used adjusting for birthweight and multiple gestation; and linear regression was used controlling for maternal MgSO4 dose) | | | | | | | | | High |
| Nassar 2006 | Cannot determine: retrospective chart review, limited detail/definitions provided (some outcomes not pre-defined) | No | Yes | Not further assessed; no control for confounders | | | | | | | | | High |
| Nelson 1995 | Cannot determine retrospective record review, with recognised limitations (e.g. for availability of CUS), and limited detail/definitions for outcomes of interest | Yes: controls matched for birthweight range, counties, and year | Yes | Not further assessed; while multivariate logistic regression analyses were conducted for CP, there was no control for confounders for review outcomes of interest | | | | | | | | | High |
| Nunes 2018 | Yes: retrospective record review, though detail/definitions provided | No | Yes | Not further assessed; no control for confounders | | | | | | | | | High |
| Okusanya 2012 | Cannot determine: prospective data collection, though limited detail/definitions provided, and reporting for perinatal mortality unclear | No | Yes | Not further assessed: no control for confounders; unclear reporting of perinatal mortality results | | | | | | | | | High |
| O Reilly 2016  Abstract | Cannot determine | Cannot determine | Cannot determine | Not further assessed; abstract only (does report that the data were controlled for GA) | | | | | | | | | Unclear |
| Ozlu 2019 | Yes: retrospective collection, though detailed definitions given | No | Yes | Not further assessed: no control for confounders | | | | | | | | | High |
| Palatnik 2019 | Cannot determine: medical record review, with limited definitions provided | Yes: though no matching of cases and controls | Partially: factors associated with sepsis or death in bi-variable analyses were retained for further analyses (models of multivariable logistic regression for the outcome sepsis) | Yes, no, no | | Cannot determine | | Yes, not blinded | | No | | No | Moderate to high |
| Paneth 1991 | Yes: prospective collection, with detail/definitions provided (note: maternal interviews also used) | Yes | Partially: adjustment for GA, fetal growth ratio, gender, multiple birth status, mode of birth, labour status, amnionitis, PE and pre-existing hypertension | No | | Cannot determine: authors note in discussion some analyses not pre-specified | | Yes, not blinded | | No | | No | Moderate |
| Perlman 1995  Abstract | Cannot determine | Cannot determine | Cannot determine | Not further assessed; abstract only (does report that stepwise logistic regression was used) | | | | | | | | | Unclear |
| Petrov 2013  Abstract | Cannot determine | Cannot determine | Cannot determine | Not further assessed; abstract only | | | | | | | | | Unclear |
| Petrova 2012 | Yes: retrospective collection with use of database/discharge files (though limited detail/definitions) | Yes: matching by GA and birthweight | Partially: controlled for PPROM, ventilation after birth, severity of distress | Yes; no; no | | Cannot determine | | Yes, no blinding (though matching was blinded) | | No | | No | Moderate to high |
| Qasim 2017  Abstract | Cannot determine | Cannot determine | Cannot determine | Not further assessed; abstract only (does report that logistic regression correcting for GA and birthweight was used) | | | | | | | | | Unclear |
| Rantonen 2001 | Yes: prospective data collection, with detail/definitions provided (limited for clinical outcomes) | No | Yes | Not further assessed: no adjustment for confounders (for outcomes of interest; in haemodynamic analyses, PV-IVH and graded ductal shunting were taken for covariates) | | | | | | | | | High |
| Rasch 1982 | Yes: prospective data collection, with detail/definitions provided | No | Yes | Not further assessed; no adjustment for confounders | | | | | | | | | High |
| Rattray 2014 | Cannot determine: retrospective collection, methods of collection not detailed | Yes | Partially: stepwise regression tested for an interaction effect between MgSO4 exposure and GA and SIP; a further model examined neonatal hydrocortisone and indomethacin exposure independently and as an interaction | No | | Cannot determine | | Yes, not blinded | | No | | No | Moderate to high |
| Rauf 2017 | Cannot determine: retrospective record review, outcomes not well defined | No | Yes | Not further assessed; no adjustment for confounders | | | | | | | | | High |
| Rhee 2012 | Yes: prospective collection with detail/definitions provided | No | Yes | Not further assessed; no adjustment for confounders | | | | | | | | | High |
| Riaz 1998 | Yes: prospective collection, including using records, with detail/definitions provided | Yes: matching by “similar gestation” | Yes | Not further assessed; no adjustment for confounders | | | | | | | | | High |
| Rizzolo 2019  Abstract | Cannot determine | Cannot determine | Cannot determine | Not further assessed; abstract only (though reports multivariate logistic regression models adjusted for patient characteristics) | | | | | | | | | Unclear |
| Sahin 2001 | Cannot determine: prospective collection, though detail/definitions not provided | No | Yes | Not further assessed; no adjustment for confounders | | | | | | | | | High |
| Sakae 2017 | Cannot determine: retrospective collection, with unclear methods for collection and limited detail/definitions | No | Yes | Not further assessed; while multiple logistic regression analysis was used to determine independent components of management protocol that contributed to the absence of serious neonatal complications, there was no adjustment for confounders for review outcome comparisons | | | | | | | | | High |
| Salafia 1995 | Yes: retrospective record review, with detail/definitions provided | Yes | Partially: stepwise regression and multivariate regression analyses assessed specific factors related to early/late GM-IVH | No | | Cannot determine | | Yes, not blinded | | No | | No | Moderate to high |
| Sarkar 2009 | Yes: retrospective collection using database/records, with detail/definitions provided | Yes | Partially: stepwise logistic multivariate regression analysis assessed factors related to severe IVH, controlling for effects of other potential confounders (GA, birthweight,  prenatal steroid use, MgSO4,  and 5-minute Apgar score < 6) | No | | Cannot determine | | Yes, not blinded | | No | | No | Moderate to high |
| Schanler 1997 | Cannot determine: prospective data collection, but limited detail/definitions provided | Cannot determine: “similar women” | Yes | Not further assessed; no adjustment for confounders | | | | | | | | | High |
| Scudiero 2000 | Yes: retrospective records review, with detail/definitions provided | Yes | Partially: multivariate logistic regression used to assess effect of > 48 g MgSO4, taking into account other possible predictors (delivery year, receipt of betamethasone, acute maternal disease, maternal race, birthweight, total dose of MgSO4) | No | | Cannot determine | | No, collection blinded to mortality outcomes | | No | | No | Moderate to high |
| Shalabi 2017 | Yes: retrospective review using database, with detail/definitions provided (though limited for covariates) | Yes | Partially: multiple logistic regression included covariates: gender, GA, SGA, Apgar score < 7 at 5 minutes, MV on day 1, antenatal steroid use, prophylactic indomethacin and indomethacin for PDA treatment | No | | Cannot determine | | Yes, not blinded | | No | | No | Moderate |
| Shamsuddin 2005 | Cannot determine: prospective data collection with structured sheets, though some reporting by family members, limited detail/definitions | No/Cannot determine | Yes | Not further assessed; no adjustment for confounders | | | | | | | | | High |
| Shokry 2010 | Yes: prospective data collection, detail/definitions provided | No | Yes | Not further assessed; no adjustment for confounders | | | | | | | | | High |
| Stetson 2019  Research Letter | Cannot determine: limited methodological detail provided | Yes | Yes/Partially | Not further assessed, multiple logistic regression analyses controlled for confounders for CP severity assessment, but no adjustment for outcomes of interest | | | | | | | | | High |
| Stockley 2018 | Yes: data collected by CNN and CNFUN using standard manuals of operations and definitions; CNN has been shown to have high consistency and reliability | Yes | Partially: multivariable logistic regression analyses adjusting for: maternal hypertension, caesarean birth, multiple gestation, GA, male sex, and SNAP-II score > 20 | No | | Cannot determine | | Yes, not blinded | | No | | No | Moderate |
| Suh 2015  Abstract | Cannot determine | Cannot determine | Cannot determine | Not further assessed; English abstract only (articles in Korean) | | | | | | | | | Unclear |
| Teng 2006 | Yes: retrospective record/database review, with detail/definitions provided | Yes | Partially: factors significantly associated with early hypotension (birthweight, GA, 1 and 5 minute Apgar scores, presence of labour, MgSO4, PE and RDS) were incorporated into multiple logistic regression model | No | | Cannot determine | | Yes, not blinded | | No | | No | Moderate to high |
| Verma 2006 | Yes: retrospective record review, with detail/definitions provided | Yes | Partially: multivariate logistic regression controlled for maximum mean FiO2 and MAP during first 7 days of life,  1 and 5 min Apgar scores, GA and surfactant requirement | No | | Cannot determine | | No, blinded outcome assessments | | No | | No | Moderate to high |
| Weintraub 2001 | Yes: retrospective collection using database, with detail/definitions provided | Yes | Partially: multivariate logistic regression included: tocolysis, antenatal steroid therapy, multiple birth, PROM, amnionitis, mode of birth, GA, birthweight, 1 and 5 minute Apgar scores, RDS, PDA, MV, pneumothorax, sepsis | No | | Cannot determine | | Yes, not blinded | | No | | No | Moderate to high |
| Weisz 2015 | Yes: retrospective collection using database, with detail/definitions provided | Yes | Partially: adjusted for GA, sex, SGA, outborn status, chorioamnionitis, mode of birth, antenatal corticosteroid use and multiple gestation (also accounting for correlated data within each site (or site effects)) | No | | Cannot determine | | Yes, not blinded | | No | | No | Moderate |
| Whitsel 2004  Abstract | Cannot determine | Cannot determine | Cannot determine | Not further assessed; abstract only (does report that multivariate logistic regression and analysis of covariance models were used to control for possible confounders) | | | | | | | | | Unclear |
| Whitten 2015  Abstract | Cannot determine | Cannot determine | Cannot determine | Not further assessed; abstract only (does report that regression analysis was used as indicated) | | | | | | | | | Unclear |
| Wiswell 1996  Abstract | Cannot determine | Cannot determine | Cannot determine | Not further assessed; abstract only | | | | | | | | | Unclear |
| Wutthigate 2017 | Yes: prospective data collection with detail/definitions provided | No | Yes | Not further assessed; no adjustment for confounders | | | | | | | | | High |
| Yokoyama 2010 | Yes: retrospective record review, though detail/definitions provided | Yes: birthweight and GA were matched | Yes | Not further assessed; though logistic regression analyses was used to assess risks for increasing ALP concentrations, there was no adjustment for confounding for outcomes of interest | | | | | | | | | High |
| Young 1977 | Cannot determine: NRT with limited detail/definitions provided | No | Yes | Not further assessed; no adjustment for confounders | | | | | | | | | High |

Abbreviations: ALP: alkaline phosphatase; aOR: adjusted odds ratios; BPD: bronchopulmonary dysplasia; CCS: case-control study; CLD: chronic lung disease; CNFUN: Canadian Neonatal Follow-Up Network; CNN: Canadian Neonatal Network; CP: cerebral palsy; CUS: cranial ultrasound; E: eclampsia; EMR: electronic medical records; ET: endotracheal; FIRS: fetal inflammatory response syndrome; GA: gestational age; GM-IVH: germinal matrix intraventricular haemorrhage; HMD: hyaline membrane disease; IUGR: intrauterine growth restriction; IVH: intraventricular haemorrhage; LOS: length of stay; MAP: mean arterial pressure; MgSO4: magnesium sulphate; MRI: magnetic resonance imaging; MV: mechanical ventilation; NEC: necrotising enterocolitis; NICU: neonatal intensive care unit; NRT: non-randomised trial; NS: not significant; ORs: odds ratios; P: p value; PDA: patent ductus arteriosus; PE: pre-eclampsia; PIE: pregnancy induced hypertension; PINS: Perinatal Information System; PTL: preterm labour; PV-IVH: periventricular intraventricular haemorrhage; PPROM: preterm premature rupture of membranes; PROM: premature rupture of membranes; PVL periventricular leucomalacia; RCT: randomised controlled trial; RDS: respiratory distress syndrome; ROP: retinopathy of prematurity; ROM: rupture of membranes; SGA: small for gestational age; SIP: spontaneous intestinal perforation; SNAP: Score for Neonatal Acute Physiology-II; VLBW: very low birthweight
