# Supplementary material for: Antenatal magnesium sulphate and adverse neonatal outcomes: A systematic review and meta-analysis
Source: PLoS Med. 2019 Dec 6;16(12):e1002988. doi: 10.1371/journal.pmed.1002988 (PMC6897495; doi:10.1371/journal.pmed.1002988)
Supplement: S3 Table — (DOCX) [file pmed.1002988.s006.docx]

**Results from non-randomised studies**

| **Study; design; overall risk of bias** | **Participants; MgSO4 indication** | **Relevant comparison groups** | **Outcome measure(s)** | **Result(s)** | **Direction of effect, indicating benefit (**✓**), harm (**🗶**) or no clear difference (~)** |
| --- | --- | --- | --- | --- | --- |
| Adama-Hondegla 2013; RCS with CCS(N); high | N = 170 women, 178 babies; E | 1: Babies still living at the 7^th^ day of life, N = 147 babies  2: Stillbirths and neonatal deaths in the 1^st^ 7 days (perinatal deaths), N = 31 babies | MgSO4 exposure | aOR 1.04; P > 0.05 | ~ |
| Alexander 2006; PCS; high | N = from 72004 births, 87 women with E and their babies included in analyses; GH/PE | 1: No GH and no MgSO4 with E, N = 49 women and their babies  2: GH and MgSO4 with E, N = 11 and their babies  3: GH and no MgSO4 with E, N = 27 and their babies | Adverse outcome composite (cord pH < 7.0, Apgar score < 4 at 5 minutes, stillbirth or neonatal death, and an unanticipated admission of a term infant to the NICU) | 12.2% vs. 9.1% vs. 11.1% | NR |
|  |  |  | Perinatal death | 6.1% vs. 0% vs. 11.1% | NR |
| Alston 2016; NCCS; high | N = 169 babies; T | 1: MgSO4 use, N = 102 (90 babies in analyses)  2: No MgSO4 use, N = 67 (64 babies in analyses) | Hospital stay (days) (mean, no measure of variance) | P = 0.89 | ~ |
|  |  |  | Neonatal death | No events | ~ |
|  |  |  | RDS | P = 0.17 | ~ |
|  |  |  | BPD | P = 0.10 | ~ |
|  |  |  | Sepsis | P = 0.74 | ~ |
|  |  |  | NEC | P = 0.40 | ~ |
|  |  |  | IVH | P = 0.47 | ~ |
| Ambadkar 2017; PCS; high | N = 120 women and babies; PE/E | 1: MgSO4, N = 60 babies  2: No MgSO4, N = 60 babies | Neonatal death | No events | ~ |
|  |  |  | NICU admission | P = 0.007 | 🗶 |
|  |  |  | Hypotonia | P = 0.028 | 🗶 |
|  |  |  | RD | P = 0.143 | ~ |
|  |  |  | Meconium passage (< 6, 6-12, > 12 hours) | P > 0.05 | ~ |
|  |  | 1: MgSO4 and NICU admission, yes, N = 13 babies  2: MgSO4 and no NICU admission, N = 47 babies | MgSO4 dose (categories: LD, LD + 1, LD + 2, LD + 3, LD + 4, LD + 5, LD + 7 doses) | P = 0.506 | ~ |
|  |  |  | Duration of MgSO4 (< 6, 6-12, 12-18, ≥ 18 hours) | P = 0.341 | ~ |
|  |  |  | Time between last MgSO4 dose and birth (1-2, 2-3, 3-4, 4-5, > 5 hours) | P = 0.0441 “the closer the last dose… the higher the rate” | 🗶 |
| Bajaj 2018; RCS; moderate | N = 7014 babies; NR | 1: Routine care without resuscitation, N = 1684 babies  2: Oxygen or CPAP, N = 2279 babies  3: Bag and mask ventilation, N = 1831 babies  4: ETT intubation, N = 1034 babies  5: CPR, N = 186 babies | MgSO4 exposure (unadjusted) | P < 0.0001 | ✓ |
|  |  |  | MgSO4 exposure, 1 as reference, vs. 2 (adjusted for centre, GA, SGA status, any antenatal steroids, and multiple births) | aOR 0.96; 95% CI 0.81-1.14 | ~ |
|  |  |  | MgSO4 exposure, 1 as reference, vs. 3 (adjusted as above) | aOR 0.98; 95% CI 0.82-1.18 | ~ |
|  |  |  | MgSO4 exposure, 1 as reference, vs. 4 (adjusted as above) | aOR 0.65; 95% CI 0.52-0.81 | ✓ |
|  |  |  | MgSO4 exposure, 1 as reference, vs. 5 (adjusted as above) | aOR 0.40; 95% CI 0.24-0.67 | ✓ |
| Basu 2012; RCS; moderate to high | N = 475 babies; FN | 1: MgSO4, N = 289 babies  2: No MgSO4, N = 186 babies | Survival without IVH/PVL | P = 0.25 | ~ |
|  |  |  | Resuscitation | P = 0.42 | ~ |
|  |  |  | Intubation | P = 0.34 | ~ |
|  |  |  | BPD | P = 0.43 | ~ |
|  |  |  | IVH/PVL | P = 0.36 | ~ |
|  |  |  | Neonatal death | P = 0.52 | ~ |
|  |  |  | ROP | P = 0.02 | 🗶 |
|  |  |  | PDA | P = 0.01 | 🗶 |
|  |  |  | LOS (days) (mean ± SD) | P = 0.01 | 🗶 |
|  |  |  | PDA, ROP, LOS (days) (mean ± SD) (multivariate logistic regression, controlled for GA and birthweight; and multiple gestations) | “Multivariate analysis performed showed that… the increased incidences of PDA, ROP, LOS… were no longer statistically significant, although the odds of developing these complications were 1.6 times more likely than in those exposed to antenatal magnesium.” | ~ |
| Belden 2017; RCS with CCS(N); moderate to high | N = 83 babies; FN/PE | 1: Enteral feeding intolerance, N = 49 babies  2: No feeding intolerance, N = 34 babies | MgSO4 dose (g) (mean ± SD) | P = 0.04 | 🗶 |
|  |  |  | MgSO4 dose (g) (mean ± SD) (multivariate logistic regression, accounting for differences between groups – assumed to be GA, birthweight, 1 and 5 minute Apgar scores) | “The strongest predictors were prematurity and cumulative maternal magnesium sulfate dose.” | 🗶 |
|  |  |  | MgSO4 dose (g) (mean ± SD), in relation to birthweight, < 1250 g, 1250-1500 g, > 1500 g | P = 0.47  P = 0.57  P = 0.48 | ~ |
|  |  | 1: MgSO4 > 80 g, N = NR  2: MgSO4 ≤ 80 g, N = NR | Enteral feeding intolerance | P = 0.04 | 🗶 |
|  |  |  | Parenteral nutrition (days) (measure NR)^ | P < 0.01 | 🗶 |
| Bertello Grecco 2019; PCS; unclear  Abstract | N = 93 women and their babies; PE | 1: MgSO4 ≤ 24 hours, N = 51 women and their babies  2: MgSO4 < 24 hours, N = 42 women and their babies | “presence of respiratory depression, admission to intensive care unit, hypotonia, and neonatal mortality” | “No statistically significant differences were observed comparing both groups in neonatal variables.” | ~ |
| Black 2006; PCS; high | N = 134 babies; PE/PIH/HELLP/T | 1: MgSO4 with (N = 45)/without (N = 5) steroids, N = 50 babies  2: No MgSO4 with (N = 38)/without steroids (N = 46), N = 84 babies | Ventilation (days) (mean ± SD) | “no significant differences among groups.” | ~ |
|  |  |  | Methylxanthines (days) (mean ± SD) | “no significant differences among groups.” | ~ |
|  |  |  | NBRS (mean ± SD)^ | “no significant differences among groups.” | ~ |
|  |  |  | IVH | “no differences among the groups on frequency or severity of IVH.”” | ~ |
| Blackwell 2002; PCS; high | N = 39 babies; PE | 1: MgSO4, N = 13 babies  2: No MgSO4, N = 26 babies | Troponin I ≥ 1.0 ng/mL (cardiac-specific protein used to detect myocardial injury)^ | P = 0.4 | ~ |
| Bonta 2000; PCS; unclear  Abstract | N = 379 women and babies; T | 1: MgSO4 < 72 hours, N = 199 babies  2: MgSO4 > 72 hours, N = 45 babies  3: No MgSO4, N = 135 babies | HsPDA treated with indomethacin | 28.1% vs. 55.6% vs. 35.6%; “Incidence… was 2:1 (> 72 group vs < 72 group)” | 🗶 |
| Bozhurt 2016; RCS; high | N = 387 babies; PE | 1: MgSO4, N = 59 babies  2: No MgSO4, N = 328 babies | RDS | “All of the p values are insignificant” | ~ |
|  |  |  | BPD | “All of the p values are insignificant” | ~ |
|  |  |  | Hypoglycaemia | “All of the p values are insignificant” | ~ |
|  |  |  | Apnoea | “All of the p values are insignificant” | ~ |
|  |  |  | PDA | “All of the p values are insignificant” | ~ |
|  |  |  | IVH grade 3/4 and PVL | “All of the p values are insignificant” | ~ |
|  |  |  | PVL only | “All of the p values are insignificant” | ~ |
|  |  |  | Culture proven sepsis | “All of the p values are insignificant” | ~ |
|  |  |  | NEC grade ≥ 2 | “All of the p values are insignificant” | ~ |
|  |  |  | ROP > 3 | “All of the p values are insignificant” | ~ |
| Boyle 2018; RCS; unclear  Abstract | N = 285 women and their babies; NR | 1: MgSO4, N = 16 babies  2: No MgSO4, N = 271 babies  [note discrepancy between total N and group Ns reported] | Composite adverse neonatal outcome (defined as Apgar score < 7 at 5 minutes, arterial cord pH < 7.1 and/or base deficit ≥ 12, admission to the NICU, need for immediate neonatal resuscitation beyond bulb suction and stimulation, or hospitalisation ≥ 3 days) (unadjusted OR from univariate logistic regression; aOR from multivariable logistic regression – confounders adjusted for NR) | OR 5.74; 95% CI 1.80-18.30; P < 0.01  aOR 4.29; 95% CI 1.24-14.81); P = 0.02 | 🗶 |
| Brazy 1982; RCS; high | N = 56 babies; ESHP | 1: Hypertensive women treated with MgSO4, N = 28 babies  2: Non-hypertensive women, with no MgSO4, N = 28 babies | Days hospitalised (mean ± SD) | P < 0.01 | 🗶 |
|  |  |  | Thrombocytopenia^ | P < 0.05 | 🗶 |
|  |  |  | Leukopenia^ | P < 0.001 | 🗶 |
|  |  |  | Neutropenia^ | P < 0.01 | 🗶 |
|  |  |  | DIC^ | P = NS | ~ |
|  |  |  | Severe respiratory disease^ | P = NS | ~ |
|  |  |  | TTN^ | P = NS | ~ |
|  |  |  | Delayed adaptation | P < 0.01 | 🗶 |
|  |  |  | PDA | P < 0.01 | 🗶 |
|  |  |  | Hypotension | P = NS | ~ |
|  |  |  | Delayed stooling (> 24 hours) | P < 0.05 | 🗶 |
|  |  |  | Ileus | P < 0.05 | 🗶 |
|  |  |  | Hypotonia | P < 0.05 | 🗶 |
|  |  |  | Other disease (CNS haemorrhage, air block, acute renal failure,^ NEC) | P = NS | ~ |
|  |  |  | Neonatal death | 7% vs. 7% | ~ |
|  |  |  | Death after 28 days of age, before hospital discharge^ | 11% vs. 0% | 🗶 |
|  |  |  | Stillbirth | No events | ~ |
| Brookfield 2015; PCS with CCS(N); unclear  Abstract | N = 55 women and babies; FN/PE | 1: Resuscitation, N = 27 babies  2: No resuscitation, N = 28 babies | MgSO4 dose (g) (mean ± SD) | P = 0.9 | ~ |
| Brookfield 2016; RCS; unclear  Abstract | N = 1496 women and babies; FN | 1: MgSO4, N = 735 babies  2: No MgSO4, N = 761 babies | RDS (multivariate logistic regression, adjusted for diabetes, mode of birth, other tocolytics, GA at birth) | aRR 0.97; 95% CI 0.84-1.12 | ~ |
|  |  |  | Ventilation (adjusted as above) | aRR 0.93; 95% CI 0.81-1.07 | ~ |
| Brown 2019; CCS; unclear  Abstract | N = 218 babies; NR | 1: SH, N = 109 babies  2: No SH, N = 109 babies | MgSO4 exposure (unadjusted P and aOR – confounders adjusted for NR) | P < 0.001  aOR 0.39; 95% CI: 0.20-0.75 | ✓ |
| Canterino 1999; RCS; moderate | N = 918 babies; PE/T | 1: MgSO4, N = 398 babies  2: No MgSO4, N = 520 babies | Apgar score < 7 at 5 minutes | P = 0.79 | ~ |
|  |  |  | RD | P = 0.38 | ~ |
|  |  |  | Neonatal death | P = 0.44 | ~ |
|  |  |  | Abnormal sonograms (any PVL or IVH) | P = 0.06 | ~ |
|  |  |  | Severe lesions (any PVL, PVL with IVH, or IVH grade 3/4) | P = 0.004 | ✓ |
|  |  |  | Severe lesions (any PVL, PVL with IVH, or IVH grade 3/4) (adjusted for clinical group) | aOR 1.11; 95% CI 0.73-1.68, P = 0.42 | ~ |
|  |  |  | Severe lesion (any PVL, PVL with IVH, or IVH grade 3/4) (adjusted for GA, birthweight, antenatal steroids, chorioamnionitis, mode of birth, Apgar score < 7 at 5 minutes, RDS) | aOR 1.10; 95% CI 0.70-1.74; P = 0.69 | ~ |
|  |  |  | Abnormal sonograms (any PVL or IVH) (adjusted for clinical group) | aOR 1.09; 95% CI 0.78-1.52; P = 0.40 | ~ |
|  |  |  | Abnormal sonograms (any PVL or IVH) (adjusted for GA, birthweight, antenatal steroids, chorioamnionitis, mode of birth, Apgar score < 7 at 5 minutes, RDS) | aOR 1.01; 95% CI 0.70-1.44; P= 0.97 | ~ |
|  |  | 1: Abnormal sonograms, N = 39 babies  2: Normal findings, N = 125 babies | Duration of MgSO4 (mean, SD) (hours) | P = 0.78 | ~ |
|  |  | 1: Severe lesions, N = 27 babies  2: Normal findings, N = 127 babies | Duration of MgSO4 (mean, SD) (hours) | P = 0.72 | ~ |
| Cawyer 2019; RCS; unclear  Abstract | N = 2468 women and their babies; PE | 1: MgSO4, N = 1353 babies  2: No MgSO4, N = 1115 babies | Perinatal or neonatal death (unadjusted P) | P = 1.00 | ~ |
|  |  |  | NICU admission (adjusted for maternal age, race/ethnicity, BMI, primary source of payment, tobacco use, illicit drug use, diabetes, chronic hypertension) | aOR 0.94; 95% CI 0.74-1.2 | ~ |
| Cho 2014; RCS; unclear  Abstract | N = 570 babies; NR | 1: MgSO4, N = 101 babies  2: No MgSO4 = 469 babies | Hypocalcaemia | “not different between groups.” | ~ |
| Chowdhury 2009; PCS (or NRT); high | N = 630 women (529 babies born to antepartum/intrapartum cases); E | 1: MgSO4 by Pritchard’s regimen, N = 480 women (406 babies born to antepartum/intrapartum cases  2: MgSO4 by low-dose IV regimen, N = 150 women (123 babies born to antepartum/intrapartum cases) | Stillbirth | 11.6% vs. 8.3% | ~ |
|  |  |  | Early neonatal death due to birth asphyxia and prematurity (1^st^ 7 days) | 15.0% vs. 10.4% | ~ |
|  |  |  | Perinatal death | OR 1.58; 95% CI 0.93-2.61; P = 0.075 | ~ |
| Chun 2014; RCS; unclear  English abstract | N = 209 women and babies; PE | 1: MgSO4, N = 119 babies  2: No MgSO4, N = 90 babies | Apgar score < 7 at 1 minute, primiparous women | P = 0.031 | 🗶 |
|  |  |  | Apgar score < 7 at 1 minute, multiparous women | P = 0.147 | ~ |
|  |  |  | Apgar score < 7 at 5 minutes, primiparous women | P = 0.017 | 🗶 |
|  |  |  | Apgar score < 7 at 5 minutes, multiparous women | P = 0.792 | ~ |
|  |  |  | NICU admission, primiparous women | P = 0.001 | 🗶 |
|  |  |  | NICU admission, multiparous women | P = 0.179 | ~ |
| Cuff 2018; RCS; moderate to high | N = 224 women and their babies: 44 women and 54 babies exposed to MgSO4 within 12 hours of birth; FN | 1: 2014 (BEAM trial: 6 g IV LD; 2 g/hour IV MD for 12 hours), N = 18 babies exposed within 12 hours of birth  2: 2015 (PREMAG trial: 4 g IV MD; no MD), N = 36 babies exposed within 12 hours of birth | Apgar score < 7 at 5 minutes, | P = 0.55 | ~ |
|  |  |  | ROP grade 3/4 | P = 0.57 | ~ |
|  |  |  | IVH grade 3/4 (binary logistic regression controlling for race, PTL, GA at delivery, corticosteroid exposure, birthweight, and indomethacin exposure) | P = 0.04  aOR 10.2; 95% CI 1.3-92 | 🗶 |
| Das 2015; PCS; high | N = 100 women and their babies; E | 1: 8 g MgSO4, N = 20 babies  2: > 8 g MgSO4, N = 80 babies | Apgar score < 7 at 1 minute | P = 0.000 | 🗶 |
|  |  |  | Apgar score < 7 at 5 minutes | P = 0.021 | 🗶 |
|  |  |  | Apgar score ≤ 3 at 1 minute^ | P = 0.002 | 🗶 |
|  |  |  | Apgar score ≤ 3 at 5 minutes^ | P = 0.003 | 🗶 |
|  |  |  | Respiratory depression | P = 0.02 | 🗶 |
|  |  |  | Intubation in delivery room | P = 0.01 | 🗶 |
|  |  |  | Bradycardia^ | P = 0.022 | 🗶 |
|  |  |  | Hypotonia | P = 0.012 | 🗶 |
|  |  |  | Hyporeflexia^ | P = 0.025 | 🗶 |
|  |  |  | NICU admission | P = 0.03 | 🗶 |
|  |  |  | Significant respiratory support in NICU^ | P = 0.000 | 🗶 |
|  |  |  | Time to 1^st^ stool > 24 hours | P = 0.011 | 🗶 |
|  |  |  | Time to 1^st^ void > 48 hours | P = 0.02 | 🗶 |
|  |  |  | Number of episodes of feeding intolerance ≥ 3^ | P = 0.000 | 🗶 |
|  |  |  | Stillbirth | P = 0.008 | 🗶 |
|  |  |  | Neonatal death due to complications of hypermagnesemia | P = 0.043 | 🗶 |
| Deering 2005; RCS; moderate to high | N = 221 babies; PE/T | 1: MgSO4, N = 103 babies (77 preterm labour)  2: No MgSO4, N = 118 babies (108 preterm labour) | SNAP score in 1^st^ 24 hours (mean ± SD) (multiple linear regression controlling for GA, birthweight, chorioamnionitis, steroid use)^ | P = 0.005 (“significant decrease”) | ✓ |
|  |  |  | SNAP score > 10 in 1^st^ 24 hours (multiple linear regression as above) | P < 0.001 | ✓ |
|  |  |  | SNAP score in 1^st^ 24 hours (mean ± SD), preterm labour only (multiple linear regression as above)^ | P = 0.047 | ✓ |
|  |  |  | SNAP score > 10 in 1^st^ 24 hours, preterm labour only (multiple linear regression as above) | P = 0.001 | ✓ |
| De Jesus 2015; RCS; moderate | N = 1544 babies; FN/PIH/T | 1: MgSO4, N = 1091 babies  2: No MgSO4, N = 453 babies | Delivery room resuscitation (PPV via bag and mask, any CPAP devices, intubation, chest compression and epinephrine) | P = 0.665 | ~ |
|  |  |  | Delivery room intubation | P = 0.157 | ~ |
|  |  |  | Delivery room intubation (multivariate logistic regression adjusted for centre, GA, antenatal steroids, and PIH/E) | aOR 1.20; 95% CI 0.88-1.65; P = 0.246 | ~ |
|  |  |  | Day 1 MV | P = 0.670 | ~ |
|  |  |  | Day 1 MV (multivariate logistic regression adjusted as above) | aOR 1.22; 95% CI 0.65-2.30; P = 0.540 | ~ |
|  |  |  | Day 1 ET MV | P = 0.023 | ✓ |
|  |  |  | Day 1 ET MV (multivariate logistic regression adjusted as above) | aOR 0.78; 95% CI 0.58-1.06; P = 0.109 | ~ |
|  |  |  | Day 3 MV | P = 0.190 | ~ |
|  |  |  | Day 3 MV (multivariate logistic regression adjusted as above) | aOR 0.65; 95% CI 0.40-1.04; P = 0.070 | ~ |
|  |  |  | Day 3 ET MV | P = 0.0002 | ✓ |
|  |  |  | Day 3 ET MV (multivariate logistic regression adjusted as above) | aOR 0.54; 95% CI 0.41-0.72; P <0.001 | ✓ |
|  |  |  | Day 1 hypotension | P = 0.043 | ✓ |
|  |  |  | Day 1 hypotension (multivariate logistic regression adjusted as above) | aOR 0.70; 95% CI 0.51-0.97; P = 0.031 | ✓ |
|  |  |  | PDA treated (medical or surgical) | P = 0.954 | ~ |
|  |  |  | PDA treated (medical or surgical) (multivariate logistic regression adjusted as above) | aOR 1.06; 95% CI 0.80-1.40; P = 0.696 | ~ |
|  |  |  | RDS | P = 0.747 | ~ |
|  |  |  | Pulmonary haemorrhage | P = 0.289 | ~ |
|  |  |  | Traditional BPD | P = 1.0 | ~ |
|  |  |  | Late onset sepsis/meningitis | P = 0.085 | ~ |
|  |  |  | NEC stage 2 or greater | P = 0.351 | ~ |
|  |  |  | ROP any stage | P = 0.359 | ~ |
|  |  |  | IVH or parenchymal haemorrhage | P = 0.294 | ~ |
|  |  |  | cPVL | P = 0.150 | ~ |
|  |  |  | Neonatal death | P = 0.223 | ~ |
|  |  |  | Cumulative days on MV (median, Q1, Q3) | P = 0.871 | ~ |
|  |  |  | Cumulative days on oxygen support (median, Q1, Q3) | P = 0.635 | ~ |
|  |  |  | LOS (median, Q1, Q3) | P = 0.621 | ~ |
| del Moral 2007; RCS; moderate to high | N = 941 babies; PE/T | 1: MgSO4, N = 546 babies  2: No MgSO4, N = 395 babies | PDA | P = 0.0178 | 🗶 |
|  |  |  | PDA (methods report co-variables: GA or birthweight, race, gender, mode of birth, antenatal steroids, presence of chorioamnionitis, MgSO4 indication) | “Logistic regression analysis showed that after controlling for confounding variables there was an increased risk of PDA in infants exposed to MgSO4.” | 🗶 |
|  |  |  | PDA, ≥ 26 weeks GA | P = 0.0399  “When stratified by gestational age the differences were significant only in the group of infants with a gestational age ≥ 26 weeks. Moreover, in these infants the incidence of PDA increased concomitantly with the dose of MgSO4 given to the mother.” | 🗶 |
|  |  |  | PDA, ≥ 26 weeks GA (methods report co-variables as above) | OR: 1.33; CI 1.12-1.58, per 50 g MgSO4  “Logistic regression analysis to adjust for co-variables indicated an increased risk of PDA with higher doses of MgSO4.” | 🗶 |
|  |  |  | PDA treated with surgical ligation | 30% vs. 34% “not different.” | ~ |
|  |  |  | Neonatal death (up to hospital discharge) | 18% vs. 22% “did not differ.” | ~ |
|  |  |  | IVH grade 3/4 | 12% vs. 13% “similar.” | ~ |
|  |  |  | PVL | 2.3% vs. 1.1% “similar.” | ~ |
| delValle 1998; PCS; unclear  Abstract | N = 110 babies; NR | 1: MgSO4, N = 34 babies  2. No MgSO4, N = 76 babies | Surfactant treatment, indomethacin treatment, PDA, NEC, IVH, PVL | “Infants exposed to maternal magnesium were comparable to non-exposed infants” | ~ |
| Derks 2016; NCCS; unclear  Abstract | N = 207 babies; FN | 1: Post MgSO4 implementation, N = 99 babies  2: Pre MgSO4 implementation, N = 108 babies | PWML at 30 weeks MRI^ | P = 0.002 | ✓ |
|  |  |  | PWML at 40 weeks MRI^ | “not [reduced]” | ~ |
|  |  |  | “neonatal complications, including early intubation for respiratory insufficiency or hypotension.” | “no increase” | ~ |
| De Silva 2018; RCS (within report of ITS); moderate | N = 14108 babies; FN | 1: MgSO4 for FN, N = 5314 babies  2: No MgSO4, N = 7238 babies  3: MgSO4 for another indication, N = 1556 babies | Intensive resuscitation (either chest compressions or intubation and ventilation or epinephrine administration in the delivery room) (adjusted for multiple gestation, gender, GA at birth, birthweight < 10^th^ centile, outborn status, mode of birth, antenatal corticosteroid use) 1 vs. 2 | aOR 0.63; 95% CI 0.54-0.73; P < 0.001 | ✓ |
|  |  |  | Intensive resuscitation (adjusted as above) 1 vs. 3 | aOR 0.81; 95% CI 0.66-0.99; P = 0.04 | ✓ |
| de Veciana 1995; RCS; high | N = 73 women, 80 babies; T | 1: MgSO4, N = 44 women, 48 babies  2: No MgSO4, N = 29 women, 32 babies | Days in hospital (mean ± SD) | P = NS | ~ |
|  |  |  | Days intubated (surviving neonates) (mean ± SD) | P = NS | ~ |
|  |  |  | Days intubated (babies with RDS) (mean ± SD) | P = 0.43 | ~ |
|  |  |  | RDS mild to severe | P = NS | ~ |
|  |  |  | RDS severe (requiring high pressure ventilation for more than 24 hours) | RR 0.47; CI 0.2-1.0; P = 0.04 | ✓ |
|  |  |  | IVH grade 1-4 | P = NS | ~ |
|  |  |  | NEC | P = NS | ~ |
|  |  |  | Neonatal death | P = NS | ~ |
|  |  |  | Apgar score < 7 at 5 minutes | P = NS | ~ |
| Downey 2017; RCS; moderate | N = 28035 babies; FN/PE/T | 1: MgSO4, N = 11789 babies  1: No MgSO4, N = 16246 babies | SIP (adjusted for site, GA at birth, multiple gestation, antenatal steroid exposure, antenatal antibiotic exposure, prolonged ROM, SGA age, sex, discharge year, postnatal hydrocortisone exposure, and postnatal indomethacin exposure) | aOR 1.08; 95% CI 0.91-1.29 | ~ |
|  |  |  | Neonatal death in 1^st^ 21 days of life (adjusted as above) | aOR 0.76; 95% CI 0.70-0.83 | ✓ |
|  |  |  | Surgical NEC (adjusted as above) | aOR 0.84; 95% CI 0.66-1.05 | ~ |
|  |  |  | Medical NEC (adjusted as above) | aOR 1.11; 95% CI 0.89-1.37 | ~ |
|  |  |  | Neonatal death, NEC or SIP (adjusted as above) | aOR 0.84; 95% CI 0.77-0.90 | ✓ |
|  |  |  | IVH grade 3/4 (adjusted as above) | aOR 0.97; 95% CI 0.88-1.06 | ~ |
| Drassinower 2015; RCS; unclear  Abstract | N = 1047 women and babies; FN | 1: MgSO4, N = 461 babies  2: Placebo, N = 586 babies | Composite of immediate outcomes (Apgar score < 7 at 5 minutes, oxygen in delivery room, intubation, chest compressions, hypotension, hypotonicity) (adjusted for GA at birth, sepsis, SGA, and alcohol use) overall; and birth ≥ 30 weeks GA | OR 0.92; 95% CI 0.79-1.08; P = 0.12  OR 0.91; 95% CI 0.73-1.14; P = 0.24 | ~ |
|  |  |  | Apgar score < 7 at 5 minutes overall; and birth ≥ 30 weeks GA | OR 0.82; 95% CI 0.59-1.14; P = 0.25  OR 1.13; 95% CI 0.56-2.28; P = 0.74 | ~ |
|  |  |  | Oxygen bag, mask or both overall; and birth ≥ 30 weeks GA | OR 1.10; 95% CI 0.80-1.41; P = 0.62  OR 0.89; 95% CI 0.71-1.11; P = 0.61 | ~ |
|  |  |  | Intubation overall; and birth ≥ 30 weeks GA (adjusted for GA at birth, sepsis, SGA, and alcohol use) | OR 0.89; 95% CI 0.71-1.11; P = 0.05  OR 0.53; 95% CI 0.32-0.88; P = 0.01 | ✓ |
|  |  |  | Chest compressions overall; and birth ≥ 30 weeks GA | OR 1.2; 95% CI 0.6-2.5; P = 0.71  OR 2.39; 95% CI 0.2-26.5; P = 0.46 | ~ |
|  |  |  | Hypotension treated with vasopressors overall; and birth ≥ 30 weeks GA | OR 0.8; 95% CI 0.6-1.2; P = 0.30  OR 0.55; 95% CI 0.28-1.09; P = 0.08 | ~ |
|  |  |  | Generalised hypotonicity overall; and birth ≥ 30 weeks GA | OR 0.78; 95% CI 0.46-1.22; P = 0.35  OR 0.89; 95% CI 0.31-2.60; P = 0.83 | ~ |
|  |  |  | RDS overall; and birth ≥ 30 weeks GA | OR 0.96; 95% CI 0.75-1.22; P = 0.72  OR NR for ≥ 30 weeks GA | ~ |
|  |  |  | MV overall; and birth ≥ 30 weeks GA | OR 0.84; 95% CI 0.66-1.07; P = 0.16  OR 0.80; 95% CI 0.54-1.18; P = 0.26 | ~ |
|  |  |  | Seizures overall; and birth ≥ 30 weeks GA | OR 0.99; 95% CI 0.37-2.67; P = 0.98  OR NR for ≥ 30 weeks GA | ~ |
|  |  |  | IVH overall; and birth ≥ 30 weeks GA | OR 0.81; 95% CI 0.60-1.1; P = 0.16  OR NR for ≥ 30 weeks GA | ~ |
|  |  |  | Neonatal death (assumed) overall; and birth ≥ 30 weeks GA | OR 1.27; 95% CI 0.78-2.08; P = 0.34  OR 2.35; 95% CI 0.69-7.90; P = 0.16 | ~ |
| Duffy 2012; RCS; high | N = 5387 women and babies; PE | 1: MgSO4, N = 248 babies  2: No MgSO4, N = 5139 babies | Composite adverse outcome (fetal acidemia, base excess ≤ -12.00, SCBU or NICU admission) | P = 0.11 | ~ |
| Edwards 2018; RCS; moderate to high | N = 1944 women and babies; FN | 1: Chorioamnionitis, N = 228 women and babies  2: No chorioamnionitis, N = 1716 women and babies | MgSO4 exposure | P = 0.76 | ~ |
|  |  |  | All below outcomes | Breslow-Day test P > 0.05 for all | ~ |
|  |  | 1: Chorioamnionitis and MgSO4, N = 109 babies  2: Chorioamnionitis and no MgSO4, N = 119 babies | IVH | OR 0.72; 95% CI 0.40-1.28; P = 0.26 | ~ |
|  |  |  | IVH (logistic regression adjusted for sex) | aOR 0.73; 95% CI 0.40-1.30 | ~ |
|  |  |  | NEC | OR 1.23; 95% CI 0.52-2.91; P = 0.64 | ~ |
|  |  |  | NEC (logistic regression adjusted for sex) | aOR 1.23; 95% CI 0.52-2.91 | ~ |
|  |  |  | BPD | OR 1.26; 95% CI 0.67-2.36; P = 0.48 | ~ |
|  |  |  | BPD (logistic regression adjusted for sex) | aOR 1.26; 95% CI 0.67-2.38 | ~ |
|  |  | 1: No chorioamnionitis and MgSO4, N = 839 babies  2: No chorioamnionitis and no MgSO4, N = 877 babies | IVH | OR 0.87; 95% CI 0.68-1.11; P = 0.26 | ~ |
|  |  |  | IVH (logistic regression adjusted for sex) | aOR 0.85; 0.66-1.09 | ~ |
|  |  |  | NEC | OR 1.15; 95% CI 0.82-1.62; P = 0.42 | ~ |
|  |  |  | NEC (logistic regression adjusted for sex) | aOR 1.17; 95% CI 0.83-1.64 | ~ |
|  |  |  | BPD | OR 1.05; 95% CI 0.81-1.35; P = 0.73 | ~ |
|  |  |  | BPD (logistic regression adjusted for sex) | aOR 1.03; 95% CI 0.79-1.33 | ~ |
| Elimian 2002; RCS; moderate to high | N = 401 babies; T | 1: MgSO4, N = 190 babies  2: No MgSO4, N = 211 babies | Apgar score < 7 at 5 minutes | P = 0.79 | ~ |
|  |  |  | RDS | P = 0.40 | ~ |
|  |  |  | Surfactant | P = 0.42 | ~ |
|  |  |  | Antibiotics | P = 0.0001 | 🗶 |
|  |  |  | PDA | P = 0.16 | ~ |
|  |  |  | IVH/PVL | P = 0.83 | ~ |
|  |  |  | NEC | P = 0.20 | ~ |
|  |  |  | Sepsis | P = 0.81 | ~ |
|  |  |  | Neonatal death (1^st^ 28 days) | P = 0.27 | ~ |
|  |  |  | Neonatal death (1^st^ 28 days) (adjustment for antenatal confounding variables) | aOR 0.66; 95% CI 0.28-1.54; P = 0.34 | ~ |
|  |  | 1: MgSO4 > 24 hours, N = 79 babies  2. MgSO4 ≤ 24 hours, N = 111 babies | Apgar score < 7 at 5 minutes | P = 0.32 | ~ |
|  |  |  | RDS | P = 0.91 | ~ |
|  |  |  | Surfactant | P = 0.31 | ~ |
|  |  |  | Antibiotics | P = 0.19 | ~ |
|  |  |  | PDA | P = 0.72 | ~ |
|  |  |  | IVH/PVL | P = 0.93 | ~ |
|  |  |  | NEC | P = 0.70 | ~ |
|  |  |  | Sepsis | P = 1.0 | ~ |
|  |  |  | Neonatal death (1^st^ 28 days) | P = 0.81 | ~ |
| Elliot 2003; RCS; unclear  Abstract | N = 9782 babies; T | 1: MgSO4, N = 6186 babies  2: No MgSO4, N = 3596 babies | Neonatal death | 7.2% vs. 7.3% | ~ |
|  |  |  | IVH | 5.7% vs. 4.4% | ~ |
|  |  |  | NEC | 4.3% vs. 4.8% | ~ |
|  |  |  | ROP | 5.2% vs. 3.2% | ~ |
|  |  |  | Morbidities (as above) | “Multivariate analysis… showed no difference” | ~ |
| Farkouh 2001; RCS; moderate to high | N = 12876 babies; PE/T | 1: MgSO4, N = 4612 babies  2: No MgSO4, N = 8264 babies | Neonatal death (death in NICU < 28 days) | OR 1.2; P = 0.06 | ~ |
|  |  |  | Neonatal death (death in NICU < 28 days) (stratified according to GA) | OR 0.67; 95% CI 0.54-0.84, P = 0.0005 | ✓ |
|  |  |  | Neonatal death (death in NICU < 28 days) (controlling for GA and MgSO4 indication) | aOR 0.70; 95% CI 0.56-0.89; P = 0.003  GA interaction: P = 0.653  MgSO4 indication interaction: P = 0.524 | ✓ |
|  |  |  | Neonatal death (death in NICU < 28 days) (logistic regression, controlling for: GA, antenatal steroids, terbutaline use, bleeding, caesarean section) | aOR 0.82; 95% CI 0.65-1.04; P = 0.108 | ~ |
| FineSmith 1997; CCS; moderate to high | N = 54 babies; PE/T | 1: cPVL, N = 18 babies  2: No cPVL, N = 36 babies | MgSO4 exposure | OR 0.19; 95% CI 0.039-0.988; P > 0.035 | ✓ |
|  |  |  | MgSO4 exposure (logistic regression including: GA, MgSO4, Apgar scores at 1 and 5 minutes, number of days intubation, reason for prematurity, type of birth) | Chi^2^ = 23.4; df = 12; P = 0.014  R statistic P < 0.03; df = 1 | ✓ |
| Gano 2016; PCS with CCS(N); moderate | N = 73 babies; FN/PE/T | 1: MgSO4, N = 49 babies  2: No MgSO4, N = 24 babies | Cerebellar haemorrhage^ | RR 0.45; 95% CI 0.26-0.81; P = 0.008 | ✓ |
|  |  |  | Cerebellar haemorrhage, size: < 3 mm vs. > 3 mm^ | P = 0.018 | ✓ |
|  |  |  | Cerebellar haemorrhage, number of foci: 1-3 vs. > 3^ | P = 0.028 | ✓ |
|  |  |  | WMI, absent/mild vs. moderate/severe^ | P = 0.53 | ~ |
|  |  |  | IVH, none/grade 1 vs. grade 3/4 | P = 0.23 | ~ |
|  |  | 1: Cerebellar haemorrhage, N = 27 babies  2: No cerebellar haemorrhage, N = 46 babies | MgSO4 exposure (none, for PE/T, for FN) | P = 0.021 | ✓ |
|  |  |  | MgSO4 exposure (univariate logistic regression) | OR 0.26; 95% CI 0.092-0.72; P = 0.010 | ✓ |
|  |  |  | MgSO4 exposure (multivariable logistic regression: adjusting for postmenstrual age at MRI, VLBW, intubation at birth, prolonged MV, hypotensive, symptomatic PDA) | aOR 0.18; 95% CI 0.049-0.65; P = 0.009 | ✓ |
|  |  |  | MgSO4 exposure (multivariable logistic regression, adjusted as above, and further for prenatal steroid exposure) | aOR 0.11; 95% CI 0.025-0.50; P = 0.004 | ✓ |
|  |  |  | MgSO4 exposure (multivariable logistic regression, adjusted as above): MgSO4 for PE/T | aOR 0.21; 95% CI 0.053-0.83; P = 0.026 | ✓ |
|  |  |  | MgSO4 exposure (multivariable logistic regression, adjusted as above): MgSO4 for FN | aOR 0.12; 95% CI, 0.019-0.77; P = 0.025 | ✓ |
| Garcia Alonso 2018; PCS; moderate to high | N = 118 babies; FN | 1: MgSO4, N = 62 babies  2: No MgSO4, N = 56 babies | Resuscitation | P = 0.04 | 🗶 |
|  |  |  | Resuscitation (multivariate analysis in presence of GA and birthweight) | “no longer statistically significant” | ~ |
|  |  |  | IMV | P = NS | ~ |
|  |  |  | Surfactant | P = 0.03 | 🗶 |
|  |  |  | Surfactant (multivariate analysis as above) | “no longer statistically significant” | ~ |
|  |  |  | BPD | P = 0.02 | 🗶 |
|  |  |  | BPD (multivariate analysis as above) | “no longer statistically significant” | ~ |
|  |  |  | PDA | P = NS | ~ |
|  |  |  | Neonatal death | P = 0.04 | ✓ |
|  |  |  | IVH | P = NS | ~ |
|  |  |  | NEC | P = NS | ~ |
|  |  |  | PVL | P = NS | ~ |
|  |  |  | ROP | P = 0.03 | 🗶 |
|  |  |  | ROP (multivariate analysis as above) | “no longer statistically significant” | ~ |
| Gasparyan 2017; PCS; unclear  English abstract | N = 62 women and babies; FN | 1: MgSO4, N = 37 babies  2: No MgSO4, N = 25 babies | IVH | “the conduction of neuroprotection does not significantly reduce IVH frequency.” | ~ |
|  |  |  | IVH grade 3/4 | 27.7% vs. 69.2%; “pronounced influence” | ✓ |
| Ghidini 2001; CCS; moderate to high | N = 69 babies; PE/T | 1: NEC, N = 23 babies  2: No NEC, N = 46 babies | MgSO4 exposure | OR 1.5; 95% CI 0.5-4.9; P = 0.4 | ~ |
|  |  |  | MgSO4 exposure (logistic regression, controlling for diagnosis of preterm labour) | P = 0.52 | ~ |
| Gibbins 2013; RCS; high | N = 373 women and their babies (313 delivered < 32 weeks in analyses for relevant outcomes); FN (unclear whether also given for PE/T) | 1: MgSO4, N = 223 babies  2: No MgSO4, N = 90 babies | Apgar score < 7 at 1 minute | P = 0.26 | ~ |
|  |  |  | Apgar score < 7 at 5 minutes | P = 0.58 | ~ |
|  |  |  | Resuscitation (none vs. oxygen vs. bag and mask vs. intubation vs. chest compressions) | P = 0.73 | ~ |
|  |  |  | Discharged alive | P = 0.52 | ~ |
|  |  |  | NICU admission | P > 0.99 | ~ |
|  |  |  | NICU LOS (days) (median range) | P = 0.93 | ~ |
|  |  |  | Individual morbidities | “did not differ significantly.” | ~ |
|  |  |  | Hypotonia | “There were no reports… in neonates exposed to magnesium.” | NA |
| Girsen 2015; RCS; moderate to high | N = 2166 women and babies; PE | 1: MgSO4, N = 1747 babies  2: No MgSO4, N = 419 babies | NICU admission | OR 1.9; 95% CI 1.4-2.7; P < 0.001 | 🗶 |
|  |  |  | NICU admission (multivariable logistic regression adjusted for potential confounding variables including receipt of public insurance, maternal age, race, type of birth, birthweight, GA at birth) | aOR 1.9; 95% CI 1.3-2.6 | 🗶 |
|  |  |  | NICU admission within 2 hours of birth | P = 0.01 | 🗶 |
|  |  |  | NICU LOS (days) (median, range) | P = 0.50 | ~ |
|  |  |  | LOS (days) (median, range) | P = < 0.001 | 🗶 |
|  |  |  | Apgar score < 7 at 1 minute | P = 0.01 | 🗶 |
|  |  |  | Apgar score < 7 at 5 minutes | P = 0.008 | 🗶 |
|  |  |  | Apgar score < 7 at 10 minutes^ | P = 0.86 | ~ |
|  |  |  | RDS | P = 0.16 | ~ |
|  |  |  | Ventilation support within 24 hours of birth | P = 0.07 | ~ |
|  |  |  | Prolonged hypotonicity within 72 hours of birth | P = 0.08 | ~ |
|  |  |  | Seizures | No events | ~ |
|  |  |  | Sepsis | P = 0.63 | ~ |
|  |  |  | HIE | P = 0.91 | ~ |
|  |  |  | Neonatal death | P = 0.44 | ~ |
|  |  |  | NICU LOS ≥ 8 days^ | OR 0.7; 95% CI 0.4-1.4 | ~ |
|  |  |  | NICU LOS ≥ 8 days (multivariable logistic regression adjusted as above)^ | aOR 0.7; 95% CI 0.3-2.3 | ~ |
| Gonzalez-Quintero 2001; PCS; unclear  Abstract | N = 851 babies; NR | 1: MgSO4, N = 438 babies  2: No MgSO4, N = 413 babies | Overall survival | “similar” | ~ |
|  |  |  | Early survival (alive at 7 days)  Early survival, infants < 700 g | P < 0.01  “more apparent”, P < 0.01 | ✓ |
|  |  |  | Severe RDS | “Similar rates” | ~ |
|  |  |  | IVH | “Similar rates” | ~ |
|  |  |  | PDA  PDA, infants < 700 g | OR 1.64; 95% CI 1.21-2.21; P < 0.01  “more evident”, P < 0.01 | 🗶 |
|  |  |  | Early PDA (< 7 days of life) | 50% vs. 45%; “not different” | ~ |
| Greenberg 2011; RCS with CCS(N); moderate to high | N = 242 babies (note: discrepancies in text and tables); PE | 1: NICU admission, N = 52 babies  2: Well baby nursery admission, N = 200 babies | Duration of MgSO4 (hours) (mean ± SD) | P < 0.001 | 🗶 |
|  |  |  | Duration of MgSO4 (hours) (mean ± SD) (multivariable regression analysis, controlled for GA, Apgar score at 1 minute, birthweight, caesarean birth, severe PE) | OR 1.06; 95% CI 1.02-1.10 | 🗶 |
|  |  |  | MgSO4 dose (g) (mean ± SD) | P < 0.001 | 🗶 |
|  |  |  | MgSO4 dose (g) (mean ± SD) (multivariable regression analysis controlled as above) | OR 1.03; 95% CI 1.01-1.05 | 🗶 |
|  |  |  | > 12 hours MgSO4 exposure (multivariable regression analysis controlled as above) | OR 2.81; 95% CI 1.31-6.03 | 🗶 |
|  |  |  | > 30 g MgSO4 exposure (multivariable regression analysis controlled as above) | OR 2.59; 95% CI 1.22-5.51 | 🗶 |
|  |  |  | Above outcomes, limited to neonates at ≥ 37 weeks GA, and controlling for operative birth for non-reassuring fetal status | “associations… remained… (data not shown)” | 🗶 |
| Greenberg 2013; RCS; moderate to high | N = 264 babies; PE | 1: MgSO4, N = 190 babies  2: No MgSO4, N = 74 babies | Meconium stained AF | P = 0.9 | ~ |
|  |  |  | NICU admission | P = 0.04 | 🗶 |
|  |  |  | NICU admission (multivariable regression analysis, controlled for GA, public insurance, birthweight, caesarean birth, chronic hypertension and severe PE) | aOR 3.69, 95% CI 1.13 to 11.99 | 🗶 |
|  |  |  | Initial admission (NICU vs. well baby nursery)^ | P = 0.6 | ~ |
|  |  |  | Primary NICU admission diagnosis (RD, rule out sepsis, hypotonia, hypothermia, LBW, hyperbilirubinaemia, hypermagnesemia, other)^ | P = 0.06 | ~ |
|  |  |  | NICU LOS (days) (median, IQR) | P = 0.4 | ~ |
|  |  |  | Respiratory treatments needed^ | P > 0.99 | ~ |
|  |  |  | Fluids/nutritional support needed | P = 0.04 | 🗶 |
|  |  |  | Antibiotics needed | P = 0.6 | ~ |
|  |  |  | Phototherapy needed | P = 0.6 | ~ |
|  |  | 1: < 12 hours MgSO4 exposure (< 30 g), N = 132 babies  2: ≥ 12 hours (≥ 30 g), N = 58 babies | NICU admission | P = 0.004 | 🗶 |
|  |  |  | NICU admission (multivariable regression analysis, assumed to be controlled as above) | aOR 2.54; 95% CI 1.05 to 6.18 | 🗶 |
|  |  |  | NICU admission (logistic regression of MgSO4 dose (g) and MgSO4 exposure (hours)) | “an increasing probability of NICU admission” [figures provided] | 🗶 |
| Grether 1998; CCS; moderate to high | N = 168 babies (128 analysed); T | 1: Neonatal death, N = 53 babies  2: Survival to 3 years with no disabling CP, N = 75 babies | MgSO4 exposure | OR 0.11; 95% CI 0.03 to 0.40 | ✓ |
|  |  |  | MgSO4 exposure for PE | 0% vs. 17.3% | ✓ |
|  |  | 1: Neonatal death no maternal PE, N = 21 babies  2: Survival to 3 years with no disabling CP no maternal PE, N = 35 babies | MgSO4 exposure | OR 0.25; 95% CI 0.6-1.1 | ~ |
|  |  |  | MgSO4 exposure (multiple linear logistic model adjusted for birthweight and GA) | aOR 0.09; 95% CI 0.01 to 0.93; P = 0.043 | ✓ |
|  |  |  | Above, adjusted for clinical or histologic diagnosis of placental infection | aOR 0.13; 95% CI 0.01-1.5 | ~ |
|  |  |  | Above, adjusted for clinical or histologic diagnosis of placental infection or suspected chorionitis | aOR 0.10; 95% CI 0.01-1.1 | ~ |
|  |  |  | Above adjusted clinical or histologic diagnosis of placental infection or maternal infection, including urinary tract infection versus no infection. | aOR 0.10; 95% CI 0.01-1.1 | ~ |
|  |  |  | Above adjusted for sex | aOR 0.09; 95% CI 0.01-0.96 | ✓ |
|  |  |  | Above, adjusted for maternal race | aOR 0.09; 95% CI 0.01-1.1 | ~ |
|  |  |  | Above, adjusted for maternal age | aOR 0.09; 95% CI 0.01-0.98 | ✓ |
|  |  |  | Above, adjusted for level of hospital care | aOR 0.09; 95% CI 0.01-0.93 | ✓ |
|  |  |  | Above, adjusted for maternal bleeding on admission | aOR 0.05; 95% CI 0.01-0.76 | ✓ |
|  |  |  | Above, adjusted for presentation at birth | aOR 0.11; 95% CI 0.01-1.2 | ~ |
|  |  |  | Above, adjusted for surgical birth | aOR 0.09; 95% CI 0.01-0.93 | ✓ |
|  |  |  | Above, adjusted for in utero exposure to corticosteroid | aOR 0.10; 95% CI 0.01-0.94 | ✓ |
|  |  |  | Above, adjusted for abruptio placentae | aOR 0.09; 95% CI 0.01-0.97 | ✓ |
|  |  |  | Above, adjusted for placenta praevia | aOR 0.07; 95% CI 0.01-0.83 | ✓ |
|  |  |  | Above, adjusted for hypertension or antihypertensive medications given during admission for birth | Not able to calculate due to 0 cells | NA |
| Grimbly 2015; RCS with CCS(N); unclear  Abstract | N = 175 babies; NR | 1: Hypoglycaemia, N = 69 babies  2: No hypoglycaemia, N = 106 babies | MgSO4 exposure | RR 0.67, P = 0.095  “antenatal administration of magnesium sulphate trended towards being protective” | ~ |
| Gulcan 2006; PCS; high | N = 200 babies; T | 1: MgSO4, N = 35 babies  2: No MgSO4, N = 165 babies | RDS | 0% vs. 27.9% | NR |
| Gursoy 2015; PCS; high | N = 50 babies; PE/T | 1: MgSO4, N = 25 babies  2: No MgSO4, N = 25 babies | Hypotension | No events | ~ |
|  |  |  | Hypertension | No events | ~ |
|  |  |  | NEC | No events | ~ |
|  |  |  | RDS | P = 0.3 | ~ |
|  |  |  | PDA | P = 0.83 | ~ |
|  |  |  | ICH stage I-2 | P = 0.12 | ~ |
|  |  |  | Feeding intolerance | P = 0.3 | ~ |
| Havranek 2011; RCS; high | N = 56 babies; PE/T | 1: MgSO4 in 24 hours prior to birth, N = 27 babies  2: No MgSO4, N = 29 babies | Caffeine treatment | P = 0.11 | ~ |
|  |  |  | Ventilator support | P = 0.14 | ~ |
|  |  |  | Phototherapy | P = 0.54 | ~ |
|  |  |  | Umbilical artery catheter^ | P = 0.57 | ~ |
|  |  |  | Enteral feedings day 1^ | P = 0.60 | ~ |
|  |  |  | Neonatal death (assumed) during hospitalisation | 3.7% vs. 6.9% | NR |
|  |  |  | NEC | 0% vs. 3.4% | NR |
| Hechtman 2002; RCS with CCS(N); unclear  Abstract | N = 85 babies; T | 1: Neonatal deaths, N = 19 babies  2: Survivors, N = 66 babies | MgSO4 exposure | P = 0.2 | ~ |
|  |  |  | MgSO4 dose (g) (median, range) | P = 0.2 | ~ |
|  |  |  | MgSO4 dose > 48 g | P = 0.4 | ~ |
|  |  |  | As above | “After controlling for GA, betamethasone therapy, clinical chorioamnionitis, and delivery mode, neither MgSO4 exposure nor total dose of antenatal MgSO4 had an impact on neonatal survival.” | ~ |
| Holcomb 1991; NCCS; unclear | N = 23 women, 33 babies; T | 1: MgSO4 > 7 days, N = 11 babies  2: No MgSO4 or < 3 days, N = 22 babies | Definitely abnormal chest radiograph (bones) (proximal humeri, radiographic abnormalities: transverse radiolucent and/or sclerotic bands) | P < 0.001 | 🗶 |
| Hom 2018; RCS; unclear  Abstract | N = 52 women and babies; FN | 1: MgSO4, N = 26 babies  2: No MgSO4, N = 26 babies | IVH | P = 0.35 | ~ |
| Hong 2019; RCS; unclear  Abstract | N = 598 babies; NR (includes FN) | 1: MgSO4 for FN not adopted (16.2% exposure), N = 270 babies  2: MgSO4 for FN routine (60.6% exposure), N = 264 babies  3: MgSO4 abandoned (14.0% exposure), N = 64 babies | Neonatal death | “not significantly different among the three periods” | ~ |
|  |  |  | Neonatal death due to NEC | P = 0.347 | ~ |
|  |  |  | NEC | P = 0.346 | ~ |
|  |  |  | NEC (grade ≥ 2) | “not significantly different among the three periods” | ~ |
|  |  |  | Other neonatal outcomes^ | “not significantly different among the three periods” | ~ |
|  |  | 1: MgSO4, N = 213 babies  2: No MgSO4, N = 385 babies | Neonatal death | “two groups were similar” | ~ |
|  |  |  | Neonatal death due to NEC | P = 0.885 | ~ |
|  |  |  | NEC | P = 0.171 | ~ |
|  |  |  | NEC (grade ≥ 2) | “two groups were similar” | ~ |
|  |  |  | Other neonatal outcomes^ | “two groups were similar” | ~ |
| Imamoglu 2014; PCS; high | N = 53 babies; PE/T | 1: MgSO4, N = 20 babies  2: No MgSO4, N = 33 babies | RDS | P = 0.8 | ~ |
|  |  |  | PDA | P = 0.7 | ~ |
|  |  |  | IVH | P = 0.52 | ~ |
|  |  |  | Caffeine treatment | P = 0.8 | ~ |
|  |  |  | Ibuprofen^ | P = 0.54 | ~ |
|  |  |  | Inotrope use | P = 0.87 | ~ |
|  |  |  | Phototherapy | P = 0.9 | ~ |
| Igarashi 1995; RCS; unclear  English abstract | N = 42 babies; T | 1: Hypermagnesemic infants exposed to MgSO4, N = 27 babies (with (N = 15) and without (N = 12) complications)  2: Infants born to “normal mothers”, N = 15 babies | See right | “In both control group and non-complication group, respiratory and cardiovascular symptoms were less found than in complication group. But the infants in complication group only had more symptoms such as respiratory depression, hypotonia, and hypotension than those in other groups. They required prolonged dopamine and calcium gluconate infusion. We speculated that complications could be attributed to disorders rather than hypermagnesemia." | NR |
| James 2015; PCS; high | N = 38 babies; FN | 1: MgSO4 within 4 hours of birth, N = 19 babies  2: No MgSO4, N = 19 babies | IVH grade 3/4 | P = 0.2 | ~ |
|  |  |  | Inotropes (1^st^ week) | P = 1.0 | ~ |
|  |  |  | Pulmonary haemorrhage | P = 0.1 | ~ |
|  |  |  | NEC | P = 0.3 | ~ |
|  |  |  | CLD | P = 0.04 | 🗶 |
|  |  |  | CLD (logistic regression, controlling for antenatal steroids) | P = 0.06 | ~ |
|  |  |  | Neonatal death (assumed) before discharge | P = 0.2 | ~ |
|  |  |  | Early onset sepsis | No events | ~ |
|  |  |  | Invasive ventilation, day 1 | P = 1.0 | ~ |
|  |  |  | Invasive ventilation, day 2 | P = 1.0 | ~ |
|  |  |  | PDA, day 1 | “all infants” | ~ |
|  |  |  | PDA, day 2 | 89.5% vs. 89.5% | ~ |
| Jazayeri 2003; RCS; high | N = 72 women and babies; T | 1: MgSO4, N = 36 babies  2: No MgSO4, N = 36 babies | NICU LOS (days) (mean ± SE) | P > 0.05 | ~ |
|  |  |  | Meconium | P = NS | ~ |
|  |  |  | RDS | P = NS | ~ |
|  |  |  | IVH | P = NS | ~ |
|  |  |  | NEC | P = NS | ~ |
|  |  |  | Sepsis | P = NS | ~ |
|  |  |  | Neonatal death | P = NS | ~ |
| Jeanneteau 2014; RCS; unclear  Abstract | N = 119 women and their babies; FN | 1: MgSO4, N = 81 women  2: No MgSO4, N = 38 women | Apgar score < 7 at 5 minutes | P = 0.03 | ✓ |
|  |  |  | Closed cardiac massage^ | P = 0.003 | ✓ |
|  |  |  | Adrenaline | P = 0.01 | ✓ |
|  |  |  | “neonatal morbi-mortality”^ | “no difference” | ~ |
| Jones 2018; RCS; unclear  Abstract | N = 120 babies; PE/T | 1: MgSO4, N = NR  2: No MgSO4, N = NR | Adverse bowel events^ | No events | ~ |
| Jung 2018; RCS; high | N = 184 women and their babies; T | 1: MgSO4, N = 143 women and babies  2: No MgSO4, N = 41 women and babies | Stillbirth, all infants, PPROM 23-27+6 weeks GA, 28-31+6 weeks GA | P = 0.0012; P = 0.0070; P = 0.4873 | ✓, ✓, ~ |
|  |  |  | Neonatal death, all infants, PPROM 23-27+6 weeks GA, 28-31+6 weeks GA | P = 0.6902; P = 0.8696; P = 0.4695 | ~, ~, ~ |
|  |  |  | Early neonatal death | P = 0.9169 | ~ |
|  |  |  | Perinatal death, all infants, PPROM 23-27+6 weeks GA, 28-31+6 weeks GA | P = 0.0375; P = 0.0651; P = 0.9051 | ✓, ~, ~ |
|  |  |  | Apgar score < 7 at 5 minutes | P = 0.7066 | ~ |
|  |  |  | Pulmonary hypoplasia^ | P = 0.8039 | ~ |
|  |  |  | RDS | P = 0.7255 | ~ |
|  |  |  | BPD | P = 0.5091 | ~ |
|  |  |  | NEC | P = 0.7437 | ~ |
|  |  |  | Early onset sepsis | P = 0.2239 | ~ |
|  |  |  | ROP | P = 0.7134 | ~ |
|  |  |  | ROP grade 2/3 | P = 0.7759 | ~ |
|  |  |  | Hearing impairment | P = 0.9028 | ~ |
|  |  |  | NICU LOS (days) (mean ± SD) | P = 0.6597 | ~ |
|  |  |  | IVH, all infants, PPROM 23-27+6 weeks GA, 28-31+6 weeks GA | RR 0.40; 95% CI 0.25-0.88  RR 0.35; 95% CI 0.17-0.71  RR 0.66; 95% CI 0.23-1.91 | ✓, ✓, ~ |
|  |  |  | IVH grade 3/4 | RR 0.37; 95% CI 0.06-2.14 | ~ |
|  |  |  | PVL, all infants, PPROM 23-27+6 weeks GA, 28-31+6 weeks GA | RR 0.60; 95% CI 0.39-0.94  RR 0.48; 95% CI 0.25-0.91  RR 0.71; 95% CI 0.39-1.29 | ✓, ✓, ~ |
|  |  |  | Bone abnormalities | 1: 4 cases (exposed for 4, 5, 20, 45 days respectively) | NA |
| Kamilya 2005; NCCS; high | N = 1205 babies; E | 1: 2002-2004 (MgSO4 use), N = 481 babies  2: 1995-1997 (no MgSO4 use), N = 724 babies | Perinatal death | 24.3% vs. 54.8%; “Recent changes in eclampsia management protocol by MgSO4 therapy and early CS have been instrumental in bringing down MMR and PNM in eclampsia cases.” | ✓ |
| Kamyar 2015a; RCS; unclear  Abstract | N = 271 babies; FN/PE/T | 1: MgSO4, N = 133 babies  2: No MgSO4, N = 138 babies | Composite morbidity (IVH, PVL, BPD, NEC, RDS, ROP and/or neonatal death) (multivariable model) | OR 1.19; CI 0.51-2.78; P = 0.69 | ~ |
|  |  |  | Neonatal death (multivariable model) | OR 0.79; CI 0.31-2.02; P = 0.74 | ~ |
|  |  |  | Individual morbidities (multivariable model) | “were also not increased.” | ~ |
| Kamyar 2015b; RCS; unclear  Abstract | N = 1246 babies; FN/PE/T | 1: MgSO4, N = 457 babies  2: No MgSO4, N = 789 babies | Composite morbidity (IVH, BPD, NEC, and/or neonatal death prior to hospital discharge) (multivariable model) | OR 1.20; CI 0.91, 1.57; P = 0.20 | ~ |
|  |  |  | Neonatal death (multivariable model) | OR 0.41; CI 0.16, 1.06; P = 0.07 | ~ |
| Kamyar 2015c; RCS; unclear  Abstract | N = 2431 babies; FN | Males, N = 1147 babies  1: MgSO4, N = 643 babies  2: No MgSO4, N = 504 babies | Composite severe morbidity (IVH grade 3/4, PVL, BPD, NEC, and/or neonatal death)  Males (multivariable model including GA as covariate) | OR 1.27; 0.94-1.72; P = 0.12 | ~ |
|  |  | Females, N = 1284 babies  1: MgSO4, N = 536 babies  2: No MgSO4, N = 748 babies | Composite severe morbidity (IVH grade 3/4, PVL, BPD, NEC, and/or neonatal death)  Females (multivariable model including GA as covariate) | OR 1.06; 0.74-1.49; P = 0.72 | ~ |
|  |  | Males vs. females | Interaction term for gender as above | P = 0.44 | ~ |
| Kamyar 2016a; RCS; moderate to high | N = 396 babies; FN | 1: MgSO4, N = 192 babies  2: Placebo, N = 204 babies | Stillbirth or death by age 1: all babies, and ≤ 28 weeks GA (adjusted multivariable log-binomial model, using backwards elimination (P < 0.20) for covariates: GA at birth, maternal years of education, maternal race/ethnicity, IUGR, illicit drug use, smoking status, and sex) | RR 1.68; 95% CI 0.85-3.32  RR 1.34; 95% CI 0.47-2.73 | ~, ~ |
|  |  |  | Severe composite morbidity (1 or more of: sepsis, severe IVH, PVL, NEC stage 2/3, BPD): all babies, and ≤ 28 weeks GA (adjusted as above) | RR 1.10; 95% CI 0.88-1.38  RR 1.07; 95% CI 0.86-1.34 | ~, ~ |
|  |  |  | Sepsis: all babies, and ≤ 28 weeks GA (adjusted as above) | RR 1.03; 95% CI 0.71-1.50  RR 1.01; 95% CI 0.67-1.51 | ~, ~ |
|  |  |  | Severe IVH: all babies, and ≤ 28 weeks GA (adjusted as above) | RR 0.36; 95% CI 0.10-1.27  RR 0.41; 95% CI 0.12-1.49 | ~, ~ |
|  |  |  | PVL: all babies, and ≤ 28 weeks GA (adjusted as above) | RR 0.37; 95% CI 0.08-1.78  RR 0.64; 95% CI 0.12-3.38 | ~, ~ |
|  |  |  | NEC stage 2/3: all babies, and ≤ 28 weeks GA (adjusted as above) | RR 1.36; 95% CI 0.58-3.20  RR 1.36; 95% CI 0.47-3.91 | ~, ~ |
|  |  |  | BPD: all babies, and ≤ 28 weeks GA (adjusted as above) | RR 1.29; 95% CI 0.91-1.82  RR 1.13; 95% CI 0.80-1.58 | ~, ~ |
|  |  |  | Neonatal death before hospital discharge: all babies, and ≤ 28 weeks GA (adjusted as above) | RR 1.46; 95% CI 0.64-3.33  RR 1.45; 95% CI 0.47-2.89 | ~, ~ |
| Kamyar 2016b; RCS with CCS(N); moderate to high | N = 697 babies; FN | 1: MgSO4, N = 332 babies  2: No MgSO4, N = 365 babies | Neonatal death before NICU discharge and/or NEC stage 2/3 (multivariable regression, adjusted for confounders: birth GA, treatment group, fetal sex, SGA, chorioamnionitis, caesarean section, hypotension during initial resuscitation, postnatal exposure to indomethacin, sepsis, IVH) | OR 1.01; 95% CI 0.69-1.47; P = 0.965 | ~ |
|  |  |  | Neonatal death before NICU discharge (multivariable regression, adjusted as above) | “MgSO4 was also not associated with elevated odds of the individual outcomes… in multivariable models (data not shown).” | ~ |
|  |  |  | NEC stage 2/3 (multivariable regression, adjusted as above) | As above | ~ |
|  |  | 1: MgSO4, N = 148 babies delivered < 26 weeks GA  2: No MgSO4, N = 145 babies delivered < 26 weeks GA | Neonatal death before NICU discharge and/or NEC stage 2/3 (unadjusted) | OR 1.82; 95% CI 1.10-3.03; P = 0.021 | 🗶 |
|  |  |  | Neonatal death before NICU discharge and/or NEC stage 2/3 (multivariable regression, controlled for confounders including birth GA and SGA) | aOR 1.90; 95% CI 1.12-3.22; P = 0.017 | 🗶 |
|  |  |  | Neonatal death before NICU discharge (multivariable regression, adjusted as above) | aOR of 1.83; 95% CI 1.03-3.27; P = 0.040 | 🗶 |
|  |  |  | NEC stage 2/3 (multivariable regression, adjusted as above) | aOR 1.38, 95% CI 0.64-3.00; P = 0.414 | ~ |
|  |  | MgSO4 exposed babies  1: Neonatal death before NICU discharge and/or NEC stage 2/3, N = 73 babies  2: Survival without NEC stage 2/3, N = 259 babies | MgSO4 infusing at birth | P = 0.700 | ~ |
|  |  |  | Total amount of MgSO4 received (g) (mean ± SD) | P = 0.595 | ~ |
| Katayama 2011; RCS; moderate to high | N = 160 babies; T | 1: MgSO4, N = 41 babies  2: No MgSO4, N = 119 babies | Early closure of the DA^ | P = 0.002 | 🗶 |
|  |  |  | Symptomatic PDA | P = 0.006 | 🗶 |
|  |  |  | Successful response to indomethacin of PDA^ | P = 0.210 | ~ |
|  |  |  | PDA treated with surgery | P = 0.210 | ~ |
|  |  |  | Failure of early closure of DA after indomethacin (univariate analysis)^ | OR 3.73; 95% CI 1.69-8.23 | 🗶 |
|  |  |  | Failure of early closure of DA after indomethacin (multivariate logistic regression analysis, adjusted for confounders including antenatal steroids, ritodrine tocolysis, PROM)^ | aOR 4.03; 95% CI 1.65-9.80; P = 0.002 | 🗶 |
|  |  |  | Symptomatic PDA (univariate analysis) | OR 2.81; 95% CI 1.33-5.92 | 🗶 |
|  |  |  | Symptomatic PDA (multivariate logistic regression analysis, adjusted for confounders, assumed to be as above) | aOR 2.26; 95% CI 1.01-5.04; P = 0.047 | 🗶 |
|  |  | MgSO4  1: Low dose (< 50 g), N = 19 babies  2: High dose (≥ 50 g), N = 22 babies | Early closure of the DA^ | 59% vs. 58%;“No significant differences” | ~ |
|  |  |  | Symptomatic PDA | 50% vs. 42%; “No significant differences” | ~ |
|  |  |  | Successful response to indomethacin of PDA^ | 73% vs. 63%; “No significant differences” | ~ |
|  |  |  | PDA treated with surgery | 27% vs. 37%; “No significant differences” | ~ |
| Kelly 1992; PCS; unclear  Abstract | N = 10 women and babies; T | 1: MgSO4, N = 5 babies  2: No MgSO4, N = 5 babies | See right^ | “Infants… did not sustain any increase in morbidity as a result of their in utero MgSO4 exposure.” | ~ |
| Khodapanahandeh 2008; CCS; moderate to high | N = 121 babies; T | 1: IVH grade 3/4, N = 39 babies  2: No IVH grade 3/4, N = 82 babies | MgSO4 exposure | P = 0.021 | 🗶 |
|  |  |  | MgSO4 exposure (multivariate logistic regression analysis, including factors significant in univariate analyses: GA, birthweight, Apgar score at 5 minutes, resuscitation, tocolytic therapy, apnoea, MV, HMD, haematocrit, PaCO2 maximum in 1^st^ 3 days, symptomatic hypotension 1^st^ 3 days) | OR 4.4; 95% CI 1.10-24.5 | 🗶 |
| Kimberlin 1998; RCS; moderate to high | N = 308 babies (363 in death analyses); T | 1: MgSO4, N = 124 babies (138 for death analyses)  2: No MgSO4, N = 184 babies (225 for death analyses) | Neonatal death ≤ 2 days^ | 10.1% vs. 18.2%; “lower” | ✓ |
|  |  |  | Neonatal death between 3-120 days | P = 0.10 | ~ |
|  |  |  | Intact survival (survival to hospital discharge or 120 days without any serious morbidities)^ | P = 0.54 | ~ |
|  |  |  | Intact survival (multiple logistic regression, controlling for birthweight, GA, race, gender, mode of birth, chorioamnionitis, surfactant treatment, antepartum steroid treatment)^ | OR 1.07; 95% CI 0.60-1.92 | ~ |
|  |  |  | Neonatal death at ≥ 2 days and < 120 days | P = 0.67 | ~ |
|  |  |  | IVH grade 3/4 | P = 0.34 | ~ |
|  |  |  | IVH grade 3/4 (multiple logistic regression as above) | OR 0.71; 95% CI 0.36-1.42 | ~ |
|  |  |  | ROP grade 3/4 | P = 0.59 | ~ |
|  |  |  | ROP grade 3/4 (multiple logistic regression as above) | OR 1.38; 95% CI 0.66-2.87 | ~ |
|  |  |  | Abnormal neurological evaluation^ | P = 0.91 | ~ |
|  |  |  | Abnormal neurological evaluation (multiple logistic regression as above)^ | OR 1.44; 95% CI 0.66-3.16 | ~ |
|  |  |  | Seizure activity | P = 0.35 | ~ |
|  |  |  | Seizure activity (multiple logistic regression as above) | OR 0.76; 95% CI 0.29-1.95 | ~ |
|  |  |  | NEC requiring surgery | P = 0.33 | ~ |
|  |  |  | NEC requiring surgery (multiple logistic regression as above) | OR 0.39; 95% CI 0.12-1.25 | ~ |
|  |  |  | Oxygen dependence at discharge | P = 0.97 | ~ |
|  |  |  | Oxygen dependence at discharge (multiple logistic regression as above) | OR 1.37; 95% CI 0.71-2.66 | ~ |
|  |  |  | Duration of ventilation (days) (median, measure of variance NR) | P = 0.08 | ~ |
|  |  |  | NICU LOS (days) (mean ± SD) | P = 0.07 | ~ |
| Koksal 2002; PCS with CCS(N); high | N = 120 babies; T | 1: GMH-IVH grade 3/4 or PVL, N = 18 babies  2: GMH-IVH grade 1/2 or no abnormalities, N = 102 babies | MgSO4 exposure | P < 0.05 | ✓ |
| Kuban 1992; PCS; moderate to high | N = 449 babies; PE/T | 1: MgSO4, N = 90 babies  2: No MgSO4, N = 359 babies | GMH-IVH | 4.4% vs. 18.9% | NR |
|  |  |  | GMH-IVH (risk among babies born to women with hypertension and proteinuria vs. neither condition): with MgSO4 vs. with no MgSO4 | OR 0.4; 95% CI 0.1-3.1 vs. OR 0.4; 9% CI 0.1-1.4 | ~; ~ |
|  |  |  | GMH-IVH (risk among babies born to women with PE): with MgSO4; with no MgSO4 | OR 0.7; 9% CI 0.1-7.3; NC due to value of 0 in 1 cell | ~; NA |
|  |  |  | GMH-IVH (risk among babies born to women who received MgSO4 vs. did not receive MgSO4): vaginal birth vs. abdominal birth | OR 0.2; 95% 0-1.2 vs. OR 0.3; 95% 0.1-1.2 | ~; ~ |
|  |  |  | GMH-IVH (risk among babies born to women who received MgSO4 vs. did not receive MgSO4): any labour vs. no labour | OR 0.3; 95% CI 0.1-0.99 vs. NC | ~; NA |
|  |  |  | GMH-IVH (risk among babies born to women who received MgSO4 vs. did not receive MgSO4): birthweight < 1000 g vs. birthweight ≥ 1000 g | OR 0.1; 95% 0-0.7 vs. OR 0.3; 95% CI 0.1-1.5 | ✓; ~ |
|  |  |  | GMH-IVH (risk among babies born to women who received MgSO4 vs. did not receive MgSO4): GA ≤ 30 weeks; GA > 30 weeks | OR 0.1; 95% CI 0-0.6 vs. OR 0.7; 95% CI 0.2-3.6 | ✓; ~ |
|  |  |  | GMH-IVH (risk among babies born to women who received MgSO4 vs. did not receive MgSO4): intubation vs. no intubation | OR 0.1; 95% CI 0-0.7 vs. OR 0.5; 95% CI 0.1-2.2 | ✓; ~ |
|  |  |  | GMH-IVH (risk among babies born to women who received MgSO4 vs. did not receive MgSO4): lowest pH < 7.2 vs. lowest pH ≥ 7.2 | NC; OR 0.3; 95% CI 0.1-0.8 | NA; ✓ |
|  |  |  | GMH-IVH (risk among babies born to women who received MgSO4 vs. did not receive MgSO4): antenatal steroids vs. no antenatal steroids | OR 0.1; 95% CI 0-0.98 vs. OR 0.3; 95% CI 0.1-1.3 | ✓; ~ |
|  |  |  | GMH-IVH (risk among babies born to women who received MgSO4 vs. did not receive MgSO4): mother’s weight/height > 75^th^ percentile vs. mother’s weight/height ≤ 75^th^ percentile | OR 0.2; 95% CI 0-1.7 vs. OR 0.2; 95% CI 0.1-1.0 | ~; ~ |
|  |  |  | See right | “In an attempt to identify which of the preeclampsia variables contributed unique and significant information about GMH risk and to control for possible confounding, we carried out a stepwise logistic regression analysis. An initial model included as potential predictors of GMH-IVH all of the preeclampsia-related variables described above. Only two variables, however, remained in the model when a .05 significance level was needed for entry. Diagnosis of preeclampsia conveyed the most information about reduced risk of GMH-IVH, followed closely by receipt of magnesium sulfate. The other preeclampsia variables (eg, pregnancyinduced hypertension, hypertension not identified as pregnancy induced, and proteinuria) did not provide additional unique information. Very similar results were obtained when covariates (eg, gestational age, birth weight, intubation, ratio of prepregnancy maternal weight to height, etc) were allowed to compete.” | ✓ |
| Lai 2017; RCS; unclear  Abstract | N = NR; PE | 1: MgSO4, N = NR  2: No MgSO4, N = NR | Muscle tone scores (units NR)^ | “lower in the MgSO4-exposed neonates… the difference did not reach statistical significance.” | ~ |
|  |  |  | SCBU admission (multinomial logistic regression)^ | OR 5.02; 95% CI 1.98-12.70 | 🗶 |
|  |  |  | NICU admission (multinomial logistic regression) | OR 3.90; 95% CI 0.49-30.99 | ~ |
|  |  |  | Delayed adaptation | “the rate… was higher in the MgSO4-exposed neonates, but again the difference was not statistically significant.” | ~ |
| Lee 2013; RCS; unclear  English abstract | N = 81 babies; PE | 1: MgSO4, N = 20  2: No MgSO4, N = 61 | RDS (adjusted for GA) | P = 0.076  aOR 1.34; 95% CI 0.32-5.53; P = 0.69 | ~ |
|  |  |  | Ventilation (adjusted for GA) | P = 0.277  aOR 0.99; 95% CI 0.26-3.88; P = 0.99 | ~ |
|  |  |  | sPDA (adjusted for GA) | P = 0.002  aOR 4.13; 95% CI 1.25-13.62; P = 0.02 | 🗶 |
|  |  |  | PDA treated with operation (adjusted for GA) | P = 1.0  aOR 1.02; 95% CI 0.07-15.75; P = 0.99 | ~ |
|  |  |  | ROP (adjusted for GA) | P = 0.149  aOR = 1.97; 95% CI 0.11-36.53; P = 0.65 | ~ |
|  |  |  | NEC (adjusted for GA) | P = 0.440  aOR = 0.35; 95% CI 0.41-2.98; P = 0.335 | ~ |
|  |  |  | IVH grade ≥ I (adjusted for GA) | P = 0.364  aOR 0.39; 95% CI 0.17-1.31; P = 0.13 | ~ |
|  |  |  | PVL (adjusted for GA) | P = 0.216  aOR 0.24; 95% CI 0.43-1.30; P = 0.10 | ~ |
|  |  |  | Neonatal death (adjusted for GA) | P = 1.0  aOR 1.12; 95% CI 0.25-4.96; P = 0.88 | ~ |
|  |  | 1: MgSO4 and sPDA, N = 15 babies  2: MgSO4 and no sPDA, N = 5 babies | MgSO4 dose (g) (mean ± SD) | P = 0.34 | ~ |
| Lee 2015; RCS; unclear  Abstract | N = 570 women and babies; T | 1: MgSO4, N = 101 babies  2: No MgSO4, N = 469 babies | Hypocalcaemia | “incidence… was not different between two groups” | ~ |
| Leung 2016; PCS with CCS(N); moderate | N = 289 babies; PE/E/T | 1: Passed hearing screen, N = 244 babies  2: Failed hearing screen, N = 45 babies | MgSO4 exposure | OR 0.38; 95% CI 0.2-0.74; P = 0.004 | ✓ |
|  |  |  | MgSO4 and betamethasone exposure | OR 0.31; 95% CI 0.16-0.60; P < 0.001 | ✓ |
|  |  |  | MgSO4 and betamethasone exposure (logistic regression model A: survivors failing hearing screen; factors entered into stepwise regression: GA, birthweight, Apgar score at 1 and 5 minutes, antenatal exposure to betamethasone, MgSO4 and an interaction term of betamethasone plus MgSO4, maternal antibiotics exposure, surfactant treatment, CUS abnormalities, duration of ventilation, CLD, duration on furosemide, gentamicin and amphotericin, FIRS, PDA treated with indomethacin) | OR: 0.37 (95% CI 0.11-0.81); P = 0.013 | ✓ |
|  |  |  | MgSO4 and betamethasone exposure (logistic regression model B: event-free survival, death (before discharge) or failure of hearing screen; factors entered into stepwise regression as above) | OR 0.33; 95% CI 0.17-0.66; P = 0.002 | ✓ |
| Leviton 1997; PCS; moderate to high | N = 1331 women and 1518 babies; unclear PE/PIH/T | 1: MgSO4, N = 678 babies  2: No MgSO4, N = 840 babies | IVH (logistic regression adjusted for GA, birthweight z score, antenatal corticosteroids, PE, PIH, route of birth and labour) | RR 1.0; 95% CI 0.7-1.3; P = 0.94 | ~ |
|  |  |  | PEA: early (logistic regression adjusted as above)^ | RR 1.3; 95% CI 0.8-2.2; P = 0.29 | ~ |
|  |  |  | PEA: late (logistic regression adjusted as above)^ | RR 0.8; 95% CI 0.5-1.5; P = 0.57 | ~ |
|  |  |  | PEA: any (logistic regression adjusted as above)^ | RR 1.0; 95% CI 0.7-1.5; P = 0.86 | ~ |
|  |  |  | PEA: hypoechoic image (logistic regression adjusted as above)^ | RR 1.2; 95% CI 0.7-2.0; P = 0.50 | ~ |
|  |  |  | PEA: late hypoechoic image (logistic regression adjusted as above)^ | RR 1.2; 95% CI 0.7-2.2; P = 0.51 | ~ |
|  |  |  | Ventriculomegaly^ | RR 1.1; 95% CI 0.7-1.7; P = 0.62 | ~ |
| Lipsitz 1971; PCS (or NRT); high | N = 37 babies; PE/E | 1: MgSO4 IV LD and MD, N = 29 babies  2: MgSO4 IV LD, IM MD, N = 8 babies | Apgar score < 7 at 1 minute | 75.9% vs. 37.5% | 🗶 |
|  |  |  | Apgar score < 7 at 5 minutes | 48.3% vs. 37.5% | 🗶 |
|  |  |  | Clinical score of 3^ | 44.8% vs. 37.5% | 🗶 |
|  |  |  | Clinical score > 0 (the higher the score, with a maximum of 3, the greater the apparent toxicity of excess Mg: 1 point for flaccidity and hyporeflexia, 1 for resuscitation or assisted ventilation, 1 for week or absent cry unrelated to tracheal intubation)^ | 82.8% vs. 62.5% | 🗶 |
|  |  |  | Neonatal death | 17.2% vs. 0% | 🗶 |
|  |  |  | Resuscitation | 48.3% vs. 37.5% | 🗶 |
|  |  |  | Assisted ventilation | 24.1% vs. 12.5% | 🗶 |
|  |  |  | Summary see right | “When magnesium sulfate is given intramuscularly to the mother, the newborn is usually not compromised by excess magnesium but may be affected. If continuous intravenous infusion of magnesium sulfate is used and especially if given for more than 24 hours, one can anticipate a newborn manifesting all the signs of hypermagnesemia” | 🗶 |
| Lloreda-Garcia 2016; NCCS; unclear  English abstract | N = 107 babies; FN | 1: MgSO4, N = 56 babies  2: No MgSO4, N = 51 babies | Resuscitation overall, < 30 weeks GA, ≥ 30 weeks GA | P = NS, P = NS, P = NS | ~, ~, ~ |
|  |  |  | Apgar score ≤ 5 at 1 minute overall, < 30 weeks GA, ≥ 30 weeks GA | P = NS, P = NS, P = NS | ~, ~, ~ |
|  |  |  | Apgar score ≤ 5 at 5 minutes | P = NS | ~ |
|  |  |  | CPAP/nasal IMV overall, < 30 weeks GA, ≥ 30 weeks GA | P = 0.03, P = NS, P = NS | 🗶, ~, ~ |
|  |  |  | CMV overall, < 30 weeks GA, ≥ 30 weeks GA | P = NS, P = 0.016, P = NS | ~, ✓, ~ |
|  |  |  | HFOV | P = NS | ~ |
|  |  |  | Surfactant treatment overall, < 30 weeks GA, ≥ 30 weeks GA | P = NS, P = 0.074, P = NS | ~, ~, ~ |
|  |  |  | PDA treated overall, < 30 weeks GA, ≥ 30 weeks GA | P = NS, P = NS, P = NS | ~, ~, ~ |
|  |  |  | Vasoactive drugs overall, < 30 weeks GA, ≥ 30 weeks GA^ | P = NS, P = 0.053, P = NS | ~, ~, ~ |
|  |  |  | Blood products overall, < 30 weeks GA, ≥ 30 weeks GA^ | P = NS, P = NS, P = 0.043 | ~, ~, 🗶 |
|  |  |  | Sepsis confirmed overall, < 30 weeks GA, ≥ 30 weeks GA | P = NS, P = NS, P = NS | ~, ~, ~ |
|  |  |  | Pathological brain ultrasound overall, < 30 weeks GA, ≥ 30 weeks GA^ | P = NS, P = NS, P = NS | ~, ~, ~ |
|  |  |  | No stools at 48 hours overall, < 30 weeks GA, ≥ 30 weeks GA | P = NS, P = 0.019, P = NS | ~, ✓, ~ |
|  |  |  | No bowel movements at 72 hours^ | P = NS | ~ |
|  |  |  | NEC overall, < 30 weeks GA, ≥ 30 weeks GA | P = NS, P = NS, P = NS | ~, ~, ~ |
|  |  |  | Neonatal death overall, < 30 weeks GA, ≥ 30 weeks GA | P = NS, P = NS, P = NS | ~, ~, ~ |
|  |  |  | CRIB (median, range)^ | P = NS | ~ |
|  |  |  | Meconium evacuation delay | P = NS | ~ |
|  |  |  | Parenteral nutrition^ | P = NS | ~ |
| Martin 1998; RCS; high | N = 193 women and babies; PE/T | 1: MgSO4, N = 118 babies  2: No MgSO4, N = 75 babies | IVH | P = 0.09 | ~ |
| Matsuda 1997; RCS with CCS(N); high | N= 139 babies born to 114 women; and a further 51 control babies; PE/T | 1: MgSO4, N = 114 women and 139 babies  2: No MgSO4, N = 51 babies | Bone abnormalities | P = 0.0101 | 🗶 |
|  |  | 1: MgSO4 and bone abnormalities, N = 13 women  2: MgSO4 and no bone abnormalities, N = 101 women | GA at start of MgSO4 (weeks) (mean ± SD) | P < 0.05 | 🗶 |
|  |  |  | Duration of MgSO4 (days) (mean ± SD) | P < 0.05 | 🗶 |
|  |  |  | MgSO4 dose (g) (mean ± SD) | P < 0.05 | 🗶 |
| McGuiness 1980; NRT; high | N = 37 women and their babies; PE | 1: MgSO4, N = 23 women and their babies  2: Dextrose-water or dextrose-saline, N = 14 women and their babies | Significant birth asphyxia | No events | ~ |
|  |  |  | Hypocalcaemia | “Although magnesium sulfate infusion cause a significant decline in maternal total calcium and ionized calcium levels, it was not associated with neonatal hypocalcemia.” | ~ |
| McPherson 2014; RCS; high | N = 933 women and their babies; FN | 1: MgSO4 < 12 hours, N = 356 women and babies  2: MgSO4 12-18 hours, N = 341 women and babies  3: MgSO4 > 18 hours, N = 236 women and babies | Apgar score < 7 at 5 minutes | 2 vs. 1: OR 0.95; 95% CI 0.64-1.41  3 vs. 1: OR 0.73; 95% CI 0.46-1.16  P = 0.37 | ~, ~, ~ |
|  |  |  | Resuscitation in delivery room (oxygen blow-by, oxygen bag, mask or both, intubation, chest compressions) | P = 0.07 | ~ |
|  |  |  | NEC | 2 vs. 1: OR 0.97; 95% CI 0.58-1.63  3 vs. 1: OR 1.11; 95% CI 0.61-1.86  P = 0.96 | ~, ~, ~ |
|  |  |  | ROP | 2 vs. 1: OR 1.16; 95% CI 0.81-1.64  3 vs. 1: OR 1.73; 95% CI 0.48-1.12  P = 0.09 | ~, ~, ~ |
|  |  |  | RDS | 2 vs. 1: OR 0.98; 95% CI 0.73-1.32  3 vs. 1: OR 0.92; 95% CI 0.66-1.28  P = 0.87 | ~, ~, ~ |
|  |  |  | MV | 2 vs. 1: OR 0.93; 95% CI 0.69-1.25  3 vs. 1: OR 0.86; 95% CI 0.62-1.20  P = 0.68 | ~, ~, ~ |
|  |  |  | BPD | 2 vs. 1: OR 0.96; 95% CI 0.66-1.41  3 vs. 1: OR 0.87; 95% CI 0.56-1.36  P = 0.84 | ~, ~, ~ |
|  |  |  | Seizures | 2 vs. 1: OR 1.25; 95% CI 0.38-3.39  3 vs. 1: OR 0.91; 95% CI 0.21-3.83  P = 0.88 | ~, ~, ~ |
|  |  |  | Any IVH | 2 vs. 1: OR 0.86; 95% CI 0.58-1.27  3 vs. 1: OR 0.92; 95% CI 0.60-1.41  P = 0.76 | ~, ~, ~ |
|  |  |  | IVH grade 3/4 | 2 vs. 1: OR 1.28; 95% CI 0.34-4.83  3 vs. 1: OR 0.36; 95% CI 0.04-2.25  P = 0.42 | ~, ~, ~ |
|  |  |  | NICU admission | “not different among groups after adjusting for gestational age at delivery (data not shown).” | ~ |
| Mikhael 2019; RCS; moderate to high | N = 302 babies; NR (includes FN) | 1: MgSO4 ≤ 7 days prior to birth, N = 210 babies  2: No MgSO4 ≤ 3 days prior to birth, N =192 babies | Death; all babies, and < 26 weeks GA | P = 0.69; P = 0.76 | ~ |
|  |  |  | Early death; all babies, and < 26 weeks GA | P = 0.28; P = 0.31 | ~ |
|  |  |  | Postnatal steroids | P = 0.67 | ~ |
|  |  |  | NEC; all babies, and < 26 weeks GA | P = 0.89; P = 0.42 | ~ |
|  |  |  | Early NEC; all babies, and < 26 weeks GA | P = 0.61; P = 0.52 | ~ |
|  |  |  | SIP; all babies, and < 26 weeks GA | P = 0.82; P = 0.73 | ~ |
|  |  |  | Early SIP; all babies, and < 26 weeks GA | P = 0.68; P = 0.79 | ~ |
|  |  |  | SIP or NEC or death: all babies, and < 26 weeks GA (unadjusted P); for all babies: aOR and P – adjusted for maternal hypertension, antenatal steroids, and antenatal indomethacin) | P = 0.81; P = 0.46  aOR 0.69; 95% CI 0.35-1.38; P = 0.29 | ~ |
|  |  |  | Early SIP or NEC or death: all babies, and < 26 weeks GA (unadjusted P); for all babies: aOR and P – adjusted for maternal hypertension, antenatal steroids, and antenatal indomethacin) | P = 0.79; P = 1.0  aOR 1.7; 95% CI 0.73-3.75; P = 0.22 | ~ |
|  |  |  | Late onset-sepsis | P = 0.075 | ~ |
|  |  |  | Postnatal NSAIDs for PDA | P = 0.15 | ~ |
|  |  |  | IVH ≥ grade 3 | P = 0.4 | ~ |
|  |  | 1: MgSO4 ≤ 3 days prior to birth, N = 179 babies  2: No MgSO4 ≤ 3 or 7 days prior to birth, N = 123 babies | Death; all babies, and < 26 weeks GA | P = 0.23; P = 0.27 | ~ |
|  |  |  | Early death; all babies, and < 26 weeks GA | P = 0.44; P = 0.43 | ~ |
|  |  |  | NEC; all babies, and < 26 weeks GA | P = 0.98; P = 0.31 | ~ |
|  |  |  | Early NEC; all babies, and < 26 weeks GA | P = 0.97; P = 0.84 | ~ |
|  |  |  | SIP; all babies, and < 26 weeks GA | P = 0.57; P = 0.36 | ~ |
|  |  |  | Early SIP; all babies, and < 26 weeks GA | P = 0.54; P = 0.44 | ~ |
|  |  |  | SIP or NEC or death; all babies, and < 26 weeks GA | P = 0.58; P = 0.29 | ~ |
|  |  |  | Early SIP or NEC or death; all babies, and < 26 weeks GA | P = 0.8412; P = 0.9 | ~ |
|  |  | 1: MgSO4 ≤ 3 days prior to birth, N = 179 babies  2: No MgSO4 ≤ 3 days prior to birth, N = 31 babies | Death; all babies, and < 26 weeks GA | P = 0.13; P = 0.16 | ~ |
|  |  |  | Early death; all babies, and < 26 weeks GA | P = 0.87; P = 1.0 | ~ |
|  |  |  | NEC; all babies, and < 26 weeks GA | P = 0.87; P = 0.51 | ~ |
|  |  |  | Early NEC; all babies, and < 26 weeks GA | P = 0.56; P = 0.65 | ~ |
|  |  |  | SIP; all babies, and < 26 weeks GA | P = 0.53; P = 0.3 | ~ |
|  |  |  | Early SIP; all babies, and < 26 weeks GA | P = 0.75; P = 0.69 | ~ |
|  |  |  | SIP or NEC or death; all babies, and < 26 weeks GA | P = 0.55; P = 0.44 | ~ |
|  |  |  | Early SIP or NEC or death; all babies, and < 26 weeks GA | P = 0.99; P = 0.83 | ~ |
|  |  | 1: Pre MgSO4 protocol, N = 112 babies  2: Post MgSO4 protocol, N = 190 babies | Death; all babies, and < 26 weeks GA | P = 0.3; P = 0.4 | ~ |
|  |  |  | NEC; all babies, and < 26 weeks GA | P = 0.5; P = 0.8 | ~ |
|  |  |  | SIP; all babies, and < 26 weeks GA | P = 0.6; P = 0.1 | ~ |
|  |  | 1: MgSO4 ≤ 3 days prior to birth, N = 179 babies | SIP or NEC or death, logistic regression modelling, each 10 g increase in MgSO4 cumulative dose | “correlated with an 18.9% decrease in SIP/NEC/death prior to discharge (95% CI 2.2–32.8%, *p* = 0.028)” | ✓ |
|  |  |  | SIP or NEC or death, logistic regression modelling, number of MgSO4 loading doses | “no significant association… OR = 1.07 (95% CI 0.53–2.1, *p* = 0.86)” | ~ |
|  |  |  | Early SIP or NEC or death, logistic regression modelling, each 10 g increase in MgSO4 cumulative dose | “correlated with … a 21.9% decrease in early SIP/NEC/death (95% CI 1.4 38.1%, *p* = 0.037)” | ✓ |
|  |  |  | Early SIP or NEC or death, logistic regression modelling, number of MgSO4 loading doses | “no significant association … early SIP/NEC/death, OR = 1.08 (95% CI 0.48–2.4, *p* = 0.86)” | ~ |
|  |  | 1: MgSO4 ≤ 7 days prior to birth, N = 210 babies  2: No MgSO4 ≤ 7 days prior to birth, N = 92 babies | SIP or NEC or death, SGA status | “found to be correlated with a reduction… by a likelihood ratio test with p = 0.07” | ~ |
|  |  |  | Early SIP or NEC or death; SGA babies only, and non-SGA babies only | OR 0.23; 95% CI 0.04-1.19; P = 0.079  OR 1.19; 95% CI 0.58-2.47; P = 0.63 | ~ |
| Mitani 2011; RCS with CCS(N); moderate to high | N = 425 babies; T | 1: MgSO4, N = 236 babies  2: No MgSO4, N = 189 babies | Perinatal death | P = 0.185 | ~ |
|  |  |  | Apgar score < 7 at 1 minute | P = 0.246 | ~ |
|  |  |  | Apgar score < 7 at 5 minutes | P = 0.817 | ~ |
|  |  |  | RDS | P = 0.543 | ~ |
|  |  |  | IVH | P = 0.879 | ~ |
|  |  |  | PVL | P = 0.630 | ~ |
|  |  | 1: Adverse outcome (IVH, PVL, CP, infantile death), N = 80 babies  2: Good outcome, N = 315 babies | MgSO4 exposure | P = 0.801 | ~ |
|  |  |  | MgSO4 exposure (multivariate logistic regression analysis; confounders NR) | OR 0.93; 95% CI 0.57-1.52  aOR 0.82; 95% CI 0.48-1.40 | ~ |
|  |  | 1: < 2 days MgSO4, N = 49 babies  2: > 2 days MgSO4, N = 174 babies | Combined adverse outcome (infantile death, IVH, PVL, CP) | P > 0.999 | ~ |
|  |  |  | IVH | P = 0.339 | ~ |
|  |  |  | PVL | P = 0.416 | ~ |
|  |  | 1: MgSO4 and adverse outcome, N = 49 babies  2: MgSO4 and good outcome, N = 174 babies | Duration of MgSO4 (hours) (median, range) | P = 0.31 | ~ |
| Mittendorf 2005; RCS; unclear  Abstract | N = 146 babies; FN/T | 1: MgSO4 0-4 g, N = 90 babies  2: MgSO4 5-49 g, N = 23 babies  3: MgSO4 ≥ 50 g, N = 33 babies | IVH grade 3 and/or LSV (adjusted for confounding), 2 vs. 3^ | aOR 0.13; 95% CI -Inf to 0.96; P = 0.045 | ✓ |
|  |  |  | IVH grade 3 and/or LSV (adjusted for confounding), 1 vs. 2^ | “trended toward neuopathogenesis (not significant; StatXact).” | ~ |
| Mittendorf 2009; RCS; unclear  Abstract | N = 140 babies; FN/T | 1: MgSO4 > 0 to < 10 g, N = 27 babies  2: MgSO4 10 to < 30 g, N = 8 babies  3: 30 to < 50 g, N = 11 babies  4: ≥ 50 g, N = 30 babies | TSV (Cochran-Armitage trend test of increasing exposures to MgSO4)^ | P = 0.22 | ~ |
|  |  |  | TSV (logistic model controlling for birthweight and funisitis), tocolytic MgSO4 : ≥ 50 g^ | P = 0.03 | 🗶 |
| Morag 2015; RCS; moderate to high | N = 645 women and 705 babies (235 preterm babies further considered); PE | 1: Preterm infants with MgSO4 exposure, N = 10 women  2: Preterm infants with no MgSO4 exposure, N = 168 women | Respiratory disease (including RDS, TTN and disorders of air leak such as pneumothorax and pneumomediastinum) (multivariate logistic regression analysis - factors from multivariate analysis: maternal age, primiparity, antenatal steroids, SGA, caesarean birth, MgSO4 treatment)^ | OR 5.17; 95% CI 1.29-20.64; P = 0.020 | 🗶 |
| Morag 2016; RCS; high | N = 190 babies; FN/PE | 1: MgSO4, N = 145 babies  2: No MgSO4, N = 45 babies | Apgar score < 7 at 1 minutes | P = 0.28 | ~ |
|  |  |  | Apgar score < 7 at 5 minutes | P = 0.03 | 🗶 |
|  |  |  | IV (days) (mean ± SD)^ | P = 0.01 “explained by out unit protocol, which recommended delay of enteral feeding in infants whose Mg concentrations are elevated.” | 🗶 |
|  |  |  | Treated early hypotension | P = 0.16 | ~ |
|  |  |  | Intubation | P = 0.44 | ~ |
|  |  |  | Oxygen at 28 days | P = 0.41 | ~ |
|  |  |  | Oxygen at 36 weeks | P = 0.34 | ~ |
|  |  |  | Proven NEC | P = 0.10 | ~ |
|  |  |  | Sepsis | P = 0.11 | ~ |
|  |  |  | IVH 3-IV/PVL | P = 0.55 | ~ |
|  |  |  | Discharge (week) (mean ± SD)^ | P = 0.22 | ~ |
|  |  |  | Neonatal death | P = 0.37 | ~ |
| Moschos 2001; CCS; unclear  Abstract | N = 75 babies; NR | 1: NEC, N = 25 babies  2: No NEC, N = 50 babies | MgSO4 exposure (logistic regression analysis) | OR 4; CI: 1.367-12.743; P = 0.016 | ✓ |
| Murata 2005; RCS with CCS(N); moderate to high | N = 201 babies; T | 1: cPVL, N = 35 babies  2: No cPVL, N = 166 babies | MgSO4 exposure (univariate analysis) | OR 0.14; 95% CI 0.040-0.95; P = 0.03 | ✓ |
|  |  |  | MgSO4 exposure (multivariate analysis including variables that were significant in the univariate analysis [Apgar score < 5 at 5 minutes, cord length > 40 cm, indomethacin exposed], with birthweight and GA by the logistic procedure) | OR 0.058; 95% CI 0.007-0.498; SE: 1.1 | ✓ |
| Nakamura 1991; RCS with CCS(N); unclear  Abstract | N = 58 women and their babies; PE | 1: Ileus, N = NR  2: No ileus, N = NR | MgSO4 dose (g) (mean ± SD) | P < 0.05 | 🗶 |
| Narasimhulu 2017; RCS; high | N = 304 women and babies; FN/PE | 1: MgSO4, N = 237 women and babies  2: No MgSO4, N = 67 women and babies | Apgar score ≤ 5 at 1 minute | P = 0.79 | ~ |
|  |  |  | Apgar score ≤ 5 at 5 minutes | P = 0.49 | ~ |
|  |  |  | Delivery room resuscitation | P = 0.56 | ~ |
|  |  |  | Hypotension | P = 0.01 | 🗶 |
|  |  |  | Hypocalcaemia | P = 0.01 | 🗶 |
|  |  |  | IVH grade 3/4 | P = 0.29 | ~ |
|  |  |  | BPD | P = 0.02 | 🗶 |
|  |  |  | ROP grade 3+ | P = 0.07 | ~ |
|  |  |  | PVL | P = 0.42 | ~ |
|  |  |  | Intubation | P = 0.36 | ~ |
|  |  |  | NEC | P = 0.75 | ~ |
|  |  |  | PDA | P = 0.02 | 🗶 |
|  |  |  | Neonatal death | P = 0.93 | ~ |
|  |  |  | Composite outcome (neonatal death, IVH grade 3/4, BPD, ROP grade 3+, PVL, NEC) | P = 0.06 | ~ |
|  |  |  | NICU LOS (days) (median, Q1-Q3) | P < 0.01 | 🗶 |
| Nassar 2006; RCS; high | N = 155 women, 198 babies; T | 1: MgSO4 > 48 hours, N = 78 women, 112 babies  2: MgSO4 ≤ 48 hours, N = 77 women, 86 babies | Apgar score < 4 at 1 minute^ | P = 0.597 | ~ |
|  |  |  | Apgar score < 7 at 5 minutes | P = 0.772 | ~ |
|  |  |  | Hypotonia | P = 0.635 | ~ |
|  |  |  | IVH | P = 0.210 | ~ |
|  |  |  | Neonatal deaths (per 1,000) | P = 0.614 | ~ |
|  |  |  | Abnormal bone mineralisation | 1: 3 cases vs. 2: 0 cases; “Abnormal bone mineralization was encountered in 3 neonates whose mothers received 4,400 and 5,500 g of MgSO4” | NA |
| Nelson 1995; CCS: high | N = 117 babies; 75 babies considered for review; PE/T | 1: No CP, MgSO4, N = 27 babies [discrepancy in text/table 29 vs. 27]  2: No CP, no MgSO4, N = 48 babies | Apgar score < 6 at 5 minutes | 23% vs. 9%; “None of these differences alone was statistically significant” | ~ |
|  |  |  | ICH/IVH, among those who underwent neuroimaging, among all | OR 0.33; 95% CI .12-1.0  OR 0.52; 95% CI .20-1.5 | ~  ~ |
| Nunes 2018; RCS; high | N = 75 women, 99 babies (94 available for analyses); FN | 1: MgSO4, N = 26 babies  2: No MgSO4, N = 68 babies | Heart rate (normal vs. abnormal)^ | OR 1.17; 95% CI 0.45-3.12; P = 0.106 | ~ |
|  |  |  | Respiratory rate (normal vs. abnormal)^ | OR 0.94; 95% CI 0.35-2.62; P = 0.017  (unclear why P value does not reflect OR) | ~ |
|  |  |  | Temperature (normal vs. abnormal)^ | OR 1.06; 95% CI 0.34-3.09; P = 0.011  (unclear why P value does not reflect OR) | ~ |
|  |  |  | Oxygen saturation (≥ 95% vs. < 95%)^ | OR 1.47; 95% CI 0.40-7.11; P = 0.319 | ~ |
|  |  |  | Hemoglucotest (normal vs. abnormal)^ | OR 1.20; 95% CI 0.42-3.72; P = 0.114 | ~ |
|  |  |  | Hemoglobin (≥ 16.4 vs. < 16.4 g/dL)^ | OR 1.00; 95% CI 0.36-2.64; P < 0.001  (unclear why P value does not reflect OR) | ~ |
|  |  |  | Ventilation (non-invasive vs. ET) | OR 2.01; 95% CI 0.80-5.23; P = 0.07 | ~ |
| Okusanya 2012; NRT; high | N = 103 women and their babies; PE/E | 1: 10 g MgSO4 LD, N = 54 (25 severe PE; 29 E) and their babies  2: 14 g MgSO4 LD, N = 49 (30 severe PE; 19 E) women and their babies | Apgar score < 7 at 5 minutes: severe PE women | P = 0.2373 | ~ |
|  |  |  | Apgar score < 7 at 5 minutes: E women | P = 0.9396 | ~ |
|  |  |  | Perinatal death: severe PE women | Results not clear  1: reports 19 livebirths, 6 perinatal deaths, and PMR 240 per 1000 vs. 2: reports 26 livebirths, 1 perinatal death, and PMR 35 per 1000 | NR |
|  |  |  | Perinatal death: E women | Results not clear  1: reports 6 perinatal deaths, and PMR 241 per 1000 [note: all women had IUFD prior to MgSO4] vs. 2: reports 0 perinatal deaths | NR |
| O Reilly 2016; RCS; unclear  Abstract | N = 100 babies; FN | 1: MgSO4, N = 55 babies  2: No MgSO4, N = 45 babies | Duration of intubation (hours) (median, variance measure NR) (controlled for GA) | P = 0.0011 | ✓ |
|  |  | 1: MgSO4, N = 55 babies | Duration of intubation (hours) (median, variance measure NR) | “Babies born to mothers who had received MgS04 closest to time of delivery remained intubated for a longer median of hours compare to those born to mothers who received it the longest amount of time before delivery.” | 🗶 |
|  |  |  | Duration of intubation (hours) (subgroups as reported to right) | “There was a notably variation in the length of hours of intubation among the subgroups who received MgS04 at different time periods before delivery. Subgroups were divided into babies whose mothers received MgS04 < 1, 0-4, > 4 h prior to delivery.” | NR |
| Ozlu 2019; RCS; high | N = 280 babies; FN | 1: 2014-2016 (post MgSO4 implementation), N = 108 babies  2: 2011-2012 (pre MgSO4 implementation), N = 172 babies | Neonatal death | P = 0.64 | ~ |
|  |  |  | Resuscitation at birth | P = 0.89 | ~ |
|  |  |  | RDS | P = 0.01 | ✓ |
|  |  |  | Ventilator support | P = 0.85 | ~ |
|  |  |  | Ventilation (days) (mean ± SD, and median, minimum and maximum) | P = 0.82 | ~ |
|  |  |  | BPD | P = 0.36 | ~ |
|  |  |  | Oxygen use (days) (mean ± SD, and median, minimum and maximum) | P = 0.65 | ~ |
|  |  |  | NEC | “None of the babies had necrotizing enterocolitis.” | ~ |
|  |  |  | Early neonatal sepsis | P = 0.25 | ~ |
|  |  |  | Feeding intolerance | P = 0.96 | ~ |
|  |  |  | Could not get full enteral feeding^ | 1: 6.4% vs. 2: 6.4% | NR |
|  |  |  | Could not start any enteral feeding^ | 1: 19.4% vs. 2: 19.8% | NR |
|  |  |  | Starting day of enteral feeding (day) (mean ± SD, and median, minimum and maximum)^ | P = 0.12 | ~ |
|  |  |  | Time of full enteral feeding (day) (mean ± SD, and median, minimum and maximum)^ | P = 0.82 | ~ |
|  |  |  | PDA | P = 0.63 | ~ |
|  |  |  | ROP | P = 0.11 | ~ |
|  |  |  | IVH | P = 0.32 | ~ |
|  |  |  | IVH grade 3/4 | P = 0.09 | ~ |
|  |  |  | Duration of hospital stay (days) (mean ± SD, and median, minimum and maximum) | P = 0.56 | ~ |
| Palatnik 2019; CCS; moderate to high | N = 779 babies; NR | 1: Early onset sepsis or death in 1^st^ week, N = 73 babies  2: No early onset sepsis or death in 1^st^ week, N = 706 babies | MgSO4 exposure | P = 0.024 | ✓ |
|  |  |  | MgSO4 exposure, model 1 (multivariable logistic regression, including GA at birth and birthweight as continuous variables, and the interaction between them) (confounders: maternal obesity, receipt of antibiotics prior to birth, receipt of 1 dose of steroids prior to birth, MgSO4 in labour, fever in labour, presence of meconium, GA at birth, birthweight) | aOR 0.82; 95% CI 0.35-1.94 | ~ |
|  |  |  | MgSO4 exposure, model 2 (multivariable logistic regression, including GA at birth as a continuous variable, birthweight dichotomised by VLBW, and the interaction between them) (confounders as above) | aOR 0.84; 95% CI 0.36-1.95 | ~ |
|  |  |  | MgSO4 exposure, model 3 (multivariable logistic regression, including GA at birth dichotomised by < 28 weeks, birthweight as a continuous variable, and the interaction between them) (confounders as above) | aOR 0.86; 95% CI 0.37-2.00 | ~ |
| Paneth 1991; PCS; moderate | N = 1037 babies; PE/PEH/T | 1: MgSO4, N = 362 babies  2: No MgSO4, N = 675 babies | GM/IVH | OR 0.89; 95% CI 0.65-1.20 | ~ |
|  |  |  | GM/IVH (multiple logistic regression adjusted for GA, fetal growth ratio, gender, multiple birth status, mode of birth, labour status, amnionitis, PE, and pre-existing hypertension) | aOR 0.89; 95% CI 0.64-1.25 | ~ |
|  |  |  | PEL/VE^ | OR 0.96; 95% CI 0.62-1.47 | ~ |
|  |  |  | PEL/VE (multiple logistic regression adjusted as above)^ | aOR 0.94; 95% CI 0.59-1.49 | ~ |
|  |  |  | Neonatal death in 1^st^ 28 days | OR 0.77; 95% CI 0.54-1.10 | ~ |
|  |  |  | Neonatal death in 1^st^ 28 days (multiple logistic regression adjusted as above) | aOR 0.83; 95% CI 0.53-1.30 | ~ |
| Perlman 1995; RCS; unclear  Abstract | N = 1025 babies; PIH | 1: MgSO4, N = 192 babies  2: No MgSO4, N = 833 babies | PV-IVH | OR 2.1; 95% CI 1.4-3.3; P < 0.05 (and stepwise logistic regression “shows that GA and Mg+ are the most significant predictors of PV-IVH”) | ✓ |
|  |  |  | PV-IVH, infants < 28 weeks | P = 0.70 | ~ |
|  |  |  | PV-IVH, infants 28-31 weeks | OR 3; 95 %CI 1.2-7.4; P < 0.05 | ✓ |
|  |  |  | IVH grade 3/4 | P < 0.05 | ✓ |
| Petrov 2013; NRT; unclear  Abstract | N = 140 women and babies; FN | 1: MgSO4, N = 80 babies  2: Placebo, N = 60 babies | Range of neurological complications^ | RR 0.55; 95% CI 0.32-0.95 | ✓ |
|  |  |  | Haemodynamic complications^ | P > 0.05 | ~ |
| Petrova 2012; RCS with CCS(N); moderate to high | N = 178 babies; T | 1: IVH, N = 89 babies  2: No IVH, N = 89 babies | MgSO4 exposure | OR 0.494; 95% CI 0.271-0.901 | ✓ |
|  |  |  | MgSO4 exposure (multiple logistic regression model, controlling for observed differences in frequency of PPROM, ventilation after birth, severity of distress - Apgar scores at 1 minute) | aOR 0.471; 95% CI 0.241-0.906 | ✓ |
| Qasim 2017; PCS with CCS(N); unclear  Abstract | N = 105 babies; NR | 1: MgSO4, N = 95 babies  2: No MgSO4, N = 10 babies | HsPDA (Pearson correlation analysis) | Negative correlation, r = -0.364, P < 0.001 | ✓ |
|  |  |  | HsPDA (regression model) | Negative linear relationship, -0.525, P < 0.001 | ✓ |
| Rantonen 2001; PCS; high | N = 55 babies (17 in ritrodrine exposure group not further considered); PE/T | 1: MgSO4, N = 19 babies  2: No MgSO4, N = 19 babies | See right | Note: below P values are reported for intergroup differences between groups 1, 2 and 3 (ritrodrine exposure) |  |
|  |  |  | Dexamethasone | P = 0.07 | ~ |
|  |  |  | Dopamine^ | P = 0.3 | ~ |
|  |  |  | Dobutamine^ | P = 0.8 | ~ |
|  |  |  | Surfactant | P = 0.7 | ~ |
|  |  |  | PDA | 5.3% vs. 10.5%; P = 0.02 | P value relates to group 2 vs. group 3 (ritodrine) |
|  |  |  | PIVH grade 1-4 | P = 0.03 | ✓ |
|  |  |  | PIVH grade 3/4 | P = 0.07 | ~ |
|  |  |  | HIE/increased echodensity | P = 0.8 | ~ |
|  |  |  | RDS and MV | P = 0.2 | ~ |
|  |  |  | NICU admission | All babies | ~ |
|  |  |  | Neonatal death | No events | ~ |
|  |  |  | Blood-culture confirmed septicaemia | No events | ~ |
| Rasch 1982; PCS; high | N = 79 babies; PE | 1: Born to PE women treated with MgSO4, N = 36 babies  2: Born to PE women with no MgSO4, N = 18 babies  3: Born to normal women, N = 25 babies | Poor sucking and cry response, 1 vs. 2 and 3^ | “poorer… until 48 hours of age” | ~ |
|  |  |  | Cyanosis during feedings | 1: 2 events | NA |
|  |  |  | Requirement for IV fluid treatment^ | 38.9% vs. 5.6% vs. 0% | 🗶 |
|  |  |  | Neurologic section of the Dubowitz examination at birth, over 24 hours after birth, 1 vs. 2 and 3^ | P < 0.001 | 🗶 |
|  |  |  | Individual measures on the Dubowitz examination^ | “those tasks which required repetitive muscle activity (head lag and ventral suspension) were accomplished less effectively by infants in Group A. Ability to perform single or low-frequency responses (arm and leg recoil) was also diminished at birth in Group A infants, but was similar for all groups by 6 hours of age.” | 🗶 |
|  |  |  | Neuromuscular transmission at 6 and 12 hours, “significant fade” 1 vs. 2 and 3 combined^ | P < 0.005 | 🗶 |
|  |  |  | Neuromuscular transmission at 24 hours, “significant fade”, 1 vs. 2 and 3 combined^ | P < 0.01 | 🗶 |
| Rattray 2014; NCCS; moderate to high | N = 155 babies; FN | 1: Pre-MgSO4 FN protocol (50.6% MgSO4), N = 81 babies  2: During MgSO4 FN protocol (78.3% MgSO4), N = 23 babies  3: After MgSO4 FN protocol (60.8% MgSO4), N = 51 babies | Postnatal hydrocortisone | P = 0.44 | ~ |
|  |  |  | Postnatal NSAIDs | P = 0.21 | ~ |
|  |  |  | SIP or neonatal death (before discharge), 1 vs. 2 vs. 3 | P = 0.45 | ~ |
|  |  |  | SIP or neonatal death (before discharge), 1 and 3 vs. 2 | P = 0.28 | ~ |
|  |  |  | SIP or neonatal death (before discharge) (multivariable analysis, MgSO4 dose (g) x GA) | P < 0.01 | 🗶 |
|  |  |  | SIP, 1 vs. 2 vs. 3 | P = 0.09 | ~ |
|  |  |  | SIP, 1 and 3 vs. 2 | P = 0.03 | 🗶 |
|  |  |  | Neonatal death (before discharge), 1 vs. 2 vs. 3 | P = 0.07 | ~ |
|  |  |  | Neonatal death (before discharge), 1 and 3 vs. 2 | P = 0.02 | 🗶 |
| Rauf 2017; RCS; high | N = 107 women and babies; FN | 1: MgSO4, N = 46 babies  2: No MgSO4, N = 61 babies | Active resuscitation at birth (ET intubation) | P = 0.015 | 🗶 |
|  |  |  | NICU LOS (days) (mean ± SD) | P = 0.929 | ~ |
|  |  |  | Respiratory support, MV, nasal CPAP, nasal SIMV, oxygen hood | P = 0.006 | 🗶, ✓ [group 1: higher rates of respiratory support, MV, nasal CPAP; lower rates of nasal SIMV, oxygen hood] |
|  |  |  | IVH | P = 0.049 | ✓ |
|  |  |  | IVH grade 1-4 | P = 0.91 | ~ |
|  |  |  | PVL | P = 0.43 | ~ |
|  |  |  | Convulsion | P = 0.63 | ~ |
|  |  |  | Hypotonia | P = 0.032 | 🗶 |
|  |  |  | Encephalopathy | P = 0.57 | ~ |
|  |  |  | ROP | P = 0.04 | 🗶 |
|  |  |  | Neonatal death | P = 0.015 | 🗶 |
| Rhee 2012; PCS; high | N = 23 women and 22 babies; PE/T | 1: MgSO4, N = 11 women, 10 babies  2: No MgSO4, N = 12 women, 12 babies | NICU admission | P = 0.02 | 🗶 |
|  |  |  | Apgar score < 7 at 5 minutes | P = 0.22 | ~ |
| Riaz 1998; PCS with CCS(N); high | N = 52 babies; PIH/T | 1: MgSO4, N = 26 babies  2: No MgSO4, N = 26 babies | Hypotonia | P < 0.001  “However, there was no association between either hypotonia at birth or Apgar scores, with… total maternal dose or duration of MgSO4 administered (p ≥ 0.29 [data not shown]).” | 🗶 |
|  |  |  | Delivery room support (bag and mask ventilation) | P = 0.19 | ~ |
|  |  |  | NICU admission | P = 0.49 | ~ |
|  |  |  | Delayed adaptation | P = 0.46 | ~ |
|  |  |  | Presumed or ruled-out sepsis | P = 0.75 | ~ |
|  |  |  | Delayed feeding (1^st^ feeding ≥ 8 hours after birth)^ | P = 0.12 | ~ |
|  |  |  | Feeding intolerance | P = 0.54 | ~ |
|  |  |  | Hospital stay (days) (mean ± SD) | P = 0.11 | ~ |
|  |  |  | Apnoea density (mean ± SD)^ | P = 0.37 | ~ |
|  |  |  | Apnoea ≥ 15 seconds (associated with bradycardia) (mean ± SD)^ | P = 0.96 | ~ |
|  |  |  | Apnoea ≥ 10 seconds (mean ± SD)^ | P = 0.16 | ~ |
|  |  |  | Pathologic apnoea (≥ 15 seconds associated with bradycardia) | P = 1.0 | ~ |
|  |  | 1: MgSO4 and NICU admission, N = 12 babies  2: MgSO4 and no NICU admission, N = 14 babies | MgSO4 dose (g) (mean ± SD), | P = 0.91 | ~ |
|  |  |  | Duration of MgSO4 (hours) (mean ± SD) | P = 0.97 | ~ |
| Rizzolo 2019; RCS; unclear  Abstract | N = 3788 babies; FN | 1: MgSO4, N = NR  2: No MgSO4, N = NR | Death or SNI (grade ≥ 3 IVH and/or PVL) (adjusted for GA, SGA, mode of birth, sex, multiple pregnancy and SNAP > 20) | aOR 0.87; 95% CI 0.71-1.05 | ~ |
| Sahin 2001; PCS; high | N = 40 babies; PE/E | 1: MgSO4, N = 20 babies  2: No MgSO4, N = 20 babies | Not voiding in 1^st^ 24 hours | No events | ~ |
|  |  |  | Residual urine after 1^st^ micturition (> 5 mL)^ | No events | ~ |
|  |  |  | Urinary tract abnormality^ | No events | ~ |
|  |  |  | Neurologic pathology^ | No events | ~ |
| Sakae 2017; NCCS; high | N = 45 women, 48 babies; PE | 1: Post-protocol: April 2013 onwards (100% MgSO4 use), N = 17 women, 19 babies  2: Pre-protocol: prior to April 2013 (36% MgSO4 use), N = 28 women, 29 babies | Composite of serious complications (1 or more of: neonatal death, assisted ventilation with ETT > 24 hours, RDS, PPH, PDA, BPD, cPVL, IVH grade ≥ 3, NEC and sepsis) (multiple logistic regression analysis, using the components of our management protocol (antenatal corticosteroids, IV nicardipine, MgSO4, indication for birth) as predictor variables) | aOR 10.07; 95% CI 1.70-103.71; P = 0.009 | 🗶 |
|  |  | 1: > 48 hours MgSO4, N = 17 women, 19 babies  2: ≤ 48 hours MgSO4, N = 10 women, 10 babies  3: No MgSO4, N = 18 women, 19 babies | Composite of serious complications | 1 vs. 2: P = 0.33  1 vs. 3: P = 0.003  2 vs. 3: P = 0.018 | ~  🗶  🗶 |
| Salafia 1995; RCS with CCS(N; moderate to high | N = 406 women and their babies; T | 1: Early GM-IVH, N = 44 babies  2: Late GM-IVH, N = 21 babies  3: No GM-IVH, N = 341 babies | MgSO4 exposure (factors significantly related to early GM-IVH in multivariate logistic regression: GA, MgSO4, antenatal steroids, volume expansion in 1^st^ 2 days, pressor agents in 1^st^ 3 days, acute amnion inflammation) | OR 2.33; 95% CI 1.128-4.814; P = 0.022; β = 0.846 | 🗶 |
| Sarkar 2009; RCS with CCS(N); moderate to high | N = 59 babies; NR | 1: IVH grade 3, N = 28 babies  2: IVH grade 4, N = 31 babies | MgSO4 exposure | P = 0.06 | ~ |
|  |  |  | MgSO4 exposure (multivariate logistic regression analysis, including GA, birthweight, prenatal steroid use, MgSO4 and Apgar score < 6 at 5 minutes) | OR 0.3; 95% CI 0.07-0.9; P = 0.04 | ✓ |
| Schanler 1997; PCS; high | N = 31 women, 41 babies; T | 1: MgSO4; N = 16 women, 22 babies  2: No MgSO4, N = 15 women, 19 babies | Apgar score < 7 at 5 minutes | 22.7% vs. 10.5% | ~ |
|  |  |  | LOS (days) (mean ± SD) | 46 ± 38 vs. 35 ± 22 | ~ |
|  |  |  | HMD | 36.4% vs. 10.5% | ~ |
|  |  |  | PDA | 13.6% vs. 0% | ~ |
|  |  |  | IVH | 22.7% vs. 10.5% | ~ |
|  |  |  | NEC | 4.5% vs. 0% | ~ |
|  |  |  | Birth depression^ | No events | ~ |
|  |  |  | Oxygen treatment | 68.2% vs. 68.4% | ~ |
|  |  |  | Oxygen treatment > 1 month^ | 13.6% vs. 5.3% | ~ |
|  |  |  | MV | 50.0% vs. 47.4% | ~ |
|  |  |  | MV > 1 week^ | 9.10% vs. 0% | ~ |
|  |  |  | Methylxanthine treatment for apnoea | 40.9% vs. 47.4% | ~ |
|  |  |  | Clinical diagnoses (as above) | “similar between groups" | ~ |
| Scudiero 2000; RCS with CCS(N); moderate to high | N = 127 babies; T | 1: Fetal or neonatal deaths (perinatal deaths), N = 18 babies  2: Survivors, N = 109 babies | MgSO4 for T > 48 g | P = 0.03 | 🗶 |
|  |  |  | MgSO4 for T ≤ 48 g vs > 48 g (multivariable logistic regression analysis, included year of birth, receipt of betamethasone, acute maternal disease, maternal race, birthweight, MgSO4 dose) | OR 4.72; 95% CI 1.12, 19.97; P = 0.035 | 🗶 |
|  |  | 1: MgSO4 ≤ 24 g, N = 43 babies  2: MgSO4 > 24 but ≤ 48 g, N = 25 babies  3: MgSO4 > 48 g, N = 59 babies | Perinatal death (Cochrane–Armitage trend test) | P = 0.03 | 🗶 |
|  |  |  | Perinatal death (1 vs. 2 only) | P = 1.0 | ~ |
| Shalabi 2017; RCS; moderate | N = 4355 babies; any | 1: MgSO4, N = 2055 babies  2: No MgSO4, N = 2300 babies | Apgar score < 7 at 5 minutes | P < 0.0001 | ✓ |
|  |  |  | SNAP-2 score > 20 | P = 0.0005 | ✓ |
|  |  |  | MV day 1 | P < 0.0001 | ✓ |
|  |  |  | Prophylactic indomethacin^ | P < 0.0001 | 🗶 |
|  |  |  | PDA treated with indomethacin | P = 0.31 | ~ |
|  |  |  | Postnatal steroids for hypotension^ | P = 0.37 | ~ |
|  |  |  | Postnatal steroid for BPD | P = 0.59 | ~ |
|  |  |  | PDA treated with indomethacin or ibuprofen | P = 0.33 | ~ |
|  |  |  | Postnatal steroid for BPD or hypotension; PDA treated with indomethacin or ibuprofen^ | P = 0.10 | ~ |
|  |  |  | NEC stage 2 or higher: all, 22-25 weeks GA, 26-27 weeks GA | P = 0.75; P = 0.45; P = 0.86 | ~, ~, ~ |
|  |  |  | NEC stage 2 or higher (multiple logistic regression, covariates included: gender, GA, SGA, Apgar score < 7 at 5 minutes, MV on day 1, antenatal steroid use, prophylactic indomethacin and indomethacin for PDA): all, 22-25 weeks GA, 26-27 weeks GA | aOR 0.92; 95% CI 0.75-1.14; P = 0.45  aOR 0.96; 95% CI 0.72-1.27; P = 0.81  aOR 0.88; 95% CI 0.65-1.20; P = 0.36 | ~  ~  ~ |
|  |  |  | SIP: all, 22-25 weeks GA, 26-27 weeks GA | P = 0.99; P = 0.69; P = 0.75 | ~, ~, ~ |
|  |  |  | SIP (multiple logistic regression, covariates as above): all, 22-25 weeks GA, 26-27 weeks GA | aOR 1.05; 95% CI 0.75-1.48; P = 0.75  aOR 1.13; 95% CI 0.74-1.72; P = 0.79  aOR 0.93; 95% CI 0.53-1.62; P = 0.69 | ~  ~  ~ |
|  |  |  | NEC or SIP: all, 22-25 weeks GA, 26-27 weeks GA^ | P = 0.62; P = 0.92; P = 0.53 | ~, ~, ~ |
|  |  |  | NEC or SIP (multiple logistic regression, covariates as above): all, 22-25 weeks GA, 26-27 weeks GA^ | aOR 0.88; 95% CI 0.73-1.07; P = 0.21  aOR 0.91; 95% CI 0.71-1.18; P = 0.43  aOR 0.85; 95% CI 0.64-1.13; P = 0.41 | ~  ~  ~ |
|  |  |  | Neonatal death prior to discharge: all, 22-25 weeks GA, 26-27 weeks GA | P < 0.0001; P < 0.0001; P = 0.56 | ✓, ✓, ~ |
|  |  |  | Neonatal death prior to discharge (multiple logistic regression, covariates as above): all, 22-25 weeks GA, 26-27 weeks GA | aOR 0.84; 95% CI 0.71-1.00; P = 0.054  aOR 0.75; 95% CI 0.61-0.93; P = 0.02  aOR 1.04; 95% CI 0.78-1.39; P = 0.74 | ~  ✓  ~ |
|  |  |  | NEC or SIP associated death: all, 22-25 weeks GA, 26-27 weeks GA | P = 0.18; P = 0.57; P = 0.21 | ~, ~, ~ |
|  |  |  | NEC or SIP associated death (multiple logistic regression, covariates as above): all, 22-25 weeks GA, 26-27 weeks GA | aOR 0.8; 95% CI 0.59-1.09; P = 0.16  aOR 0.89; 95% CI 0.61-1.31; P = 0.44  aOR 0.72; 95% CI 0.43-1.19; P = 0.17 | ~  ~  ~ |
|  |  |  | IVH grade 3/4 or PVL: all, 22-25 weeks GA, 26-27 weeks GA | P = 0.002; P = 0.001; P = 0.41 | ✓, ✓, ~ |
|  |  |  | IVH grade 3/4 or PVL (multiple logistic regression, covariates as above): all, 22-25 weeks GA, 26-27 weeks GA | aOR 0.91; 95% CI 0.78-1.07; P = 0.26  aOR 0.80; 95% CI 0.65-0.99; P = 0.048  aOR 1.07; 95% CI 0.85-1.35; P = 0.98 | ~  ✓  ~ |
|  |  |  | ROP stage 3 or above or ROP treated: all, 22-25 weeks GA, 26-27 weeks GA | P = 0.09; P = 0.06; P = 0.4 | ~, ~, ~ |
|  |  |  | ROP stage 3 or above or ROP treated (multiple logistic regression, covariates as above): all, 22-25 weeks GA, 26-27 weeks GA | aOR 0.81; 95% CI 0.65-0.999; P = 0.049  aOR 0.77; 95% CI 0.6-1.001; P = 0.063  aOR 0.88; 95% CI 0.6-1.29; P = 0.23 | ✓  ~  ~ |
|  |  |  | BPD: all, 22-25 weeks GA, 26-27 weeks GA | P = 0.18; P = 0.36; P = 0.24 | ~, ~, ~ |
|  |  |  | BPD (multiple logistic regression, covariates as above): all, 22-25 weeks GA, 26-27 weeks GA | aOR 0.92; 95% CI 0.79-1.06; P = 0.23  aOR 0.9; 95% CI 0.72-1.12; P = 0.82  aOR 0.93; 95% CI 0.77-1.12; P = 0.42 | ~  ~  ~ |
|  |  |  | Nosocomial infection: all, 22-25 weeks GA, 26-27 weeks GA^ | P = 0.04; P 0.0007; P = 0.85 | 🗶, 🗶, ~ |
|  |  |  | Nosocomial infection (multiple logistic regression, covariates as above): all, 22-25 weeks GA, 26-27 weeks GA^ | aOR 1.08; 95% CI 0.94-1.25; P = 0.26  aOR 1.26; 95% CI 1.03-1.53; P = 0.04  aOR 0.93; 95% CI 0.76-1.13; P = 0.76 | ~  🗶  ~ |
| Shamsuddin 2005; NRT; high | N = 265 women and their babies (207 antepartum/intrapartum PE/E cases); PE/E | 1: MgSO4 LD at home before referral to hospital, N = 102 women and their babies  2: No MgSO4 before referral to hospital, N = 105 women and their babies | Asphyxia | 18.6% vs. 27.6%; (“poorer” in group 2) | ✓ |
|  |  |  | Stillbirth | P < 0.001 | ✓ |
| Shokry 2010; PCS; high | N = 48 women and their babies; T | 1: MgSO4, N = 28 women and their babies  2: No MgSO4, N = 20 women and their babies | RDS | P = 0.762 | ~ |
|  |  |  | PIVH | P = 0.036 | ✓ |
|  |  |  | Seizures | P = 0.011 | ✓ |
|  |  |  | MV | P = 0.836 | ~ |
|  |  |  | Surfactant use | P = 0.874 | ~ |
|  |  |  | Inotropic drug use | P = 0.498 | ~ |
|  |  |  | PDA | P = 0.042 | 🗶 |
|  |  |  | Neonatal death | P = 0.480 | ~ |
| Stetson 2019; NCCS; high  Research Letter | N = 110 babies; FN/PE | 1: 2002-2008 (pre-BEAM trial, 36% uptake MgSO4), N = 42 babies  2: 2009-2014 (post-BEAM trial, 62% uptake MgSO4), N = 68 babies | BPD  1: 67% babies exposed to MgSO4 vs. 56% not exposed  2: 86% babies exposed to MgSO4 vs. 73% not exposed | P = 0.049 | 🗶 |
|  |  |  | IVH  1: 40% babies exposed to MgSO4 vs. 56% not exposed  2: 60% babies exposed to MgSO4 vs. 62% not exposed | P = 0.41 | ~ |
| Stockley 2018; RCS; moderate | N = 336 babies; NR | Growth restriction (fetal standards)  1: MgSO4, N = 112 babies  2: No MgSO4, N = 224 babies | Death in NICU and post-discharge (adjusted for GA, sex, mode of birth, multiple birth, SNAP-II > 20, maternal hypertension) | aOR (95% CI): 0.42 (0.19-0.95) | ✓ |
|  |  |  | Apgar score < 7 at 5 minutes | P = 0.65 | ~ |
|  |  |  | Chest compression or epinephrine | P = 0.70 | ~ |
|  |  |  | SNAP-II score > 20 | P = 0.36 | ~ |
|  |  |  | BPD (adjusted as above) | aOR 0.84; 95% CI 0.46-1.52 | ~ |
|  |  |  | NEC (adjusted as above) | aOR 0.38; 95% CI 0.15-1.00 | ~ |
|  |  |  | Late-onset sepsis (adjusted as above) | aOR 0.89; 95% CI 0.49-1.61 | ~ |
|  |  |  | ROP stage 3/4/5 or treated (adjusted as above) | aOR 0.80; 95% CI 0.34-1.88 | ~ |
|  |  |  | IVH grade 1/2 (adjusted as above) | aOR 1.02; 95% CI 0.53-1.94 | ~ |
|  |  |  | IVH grade 3/4 (adjusted as above) | aOR 0.55; 95% CI 0.20-1.51 | ~ |
|  |  | Growth restriction (neonatal standards)  1: MgSO4, N = 61 babies  2: No MgSO4, N = 116 babies | Death in NICU and post-discharge (adjusted as above) | aOR 0.37; 95% CI 0.15-0.95 | ✓ |
|  |  |  | Apgar score < 7 at 5 minutes | P = 0.83 | ~ |
|  |  |  | Chest compression or epinephrine | P = 0.49 | ~ |
|  |  |  | SNAP-II score > 20 | P = 0.60 | ~ |
|  |  |  | BPD (adjusted as above) | aOR 1.10; 95% CI 0.47-2.61 | ~ |
|  |  |  | NEC (adjusted as above) | aOR 0.57; 95% CI 0.16-2.00 | ~ |
|  |  |  | Late-onset sepsis (adjusted as above) | aOR 0.71; 95% CI 0.32-1.56 | ~ |
|  |  |  | ROP stage 3/4/5 or treated (adjusted as above) | aOR 0.96; 95% CI 0.34-2.73 | ~ |
|  |  |  | IVH grade 1/2 (adjusted as above) | aOR 0.54; 95% CI 0.20-1.42 | ~ |
|  |  |  | IVH grade 3/4 (adjusted as above) | aOR 0.68; 95% CI 0.20-2.34 | ~ |
| Suh 2015; RCS; unclear  English abstract | N = 150 babies (of relevance); HD | 1: MgSO4, N = 40 babies  2: No MgSO4, N = 110 babies | LOS (days) (mean ± SD) | P = 0.181 | ~ |
|  |  |  | Duration of ventilation(days) (mean ± SD) | P = 0.078 | ~ |
|  |  |  | Duration of oxygen (days) (mean ± SD) | P = 0.205 | ~ |
|  |  |  | RDS | P = 0.242 | ~ |
|  |  |  | BPD | P = 0.264 | ~ |
|  |  |  | Moderate to severe  BPD | P = 0.576 | ~ |
|  |  |  | PDA treated (medication ± operation) | P = 0.534 | ~ |
|  |  |  | ROP treated with laser | P = 0.086 | ~ |
|  |  |  | NEC | P = 0.528 | ~ |
|  |  |  | IVH grade ≥ 2 | P = 0.151 | ~ |
|  |  |  | PVL | P = 0.053 | ~ |
|  |  |  | Neonatal death | P = 0.320 | ~ |
| Teng 2006; RCS with CCS(N); moderate to high | N = 184 babies; PE/T | 1: Early hypotension, N = 75 babies  2: No early hypotension, N = 109 babies | MgSO4 exposure (identified as one of eight variables associated with hypotension, by univariate analysis) | OR 2.83; 95% CI 1.52-5.27; P < 0.01 | 🗶 |
|  |  |  | MgSO4 exposure (incorporated into multiple logistic regression model) | No longer associated | ~ |
|  |  |  | Positive blood culture | No events | ~ |
| Verma 2006; RCS with CCS(N); moderate to high | N = 45 babies; PE/T | 1: PIE^, N = 11 babies  2: No PIE, N = 34 babies | MgSO4 dose (g) (mean ± SD) | P = 0.02 | 🗶 |
|  |  |  | MgSO4 dose ≥ 10 g | P = 0.01 | 🗶 |
|  |  |  | MgSO4 dose ≥ 10 g (multivariate logistic regression analysis model, controlling for maximum mean FiO2 and MAP during 1^st^ 7 days of life, Apgar scores at 1 and 5 minutes, GA and surfactant requirement) | OR 19.8; 95% CI 1.5-263; P = 0.01 | 🗶 |
| Weintraub 2001; RCS; moderate to high | N = 2794 babies (have not considered the 263 babies and 177 infants exposed to ritodrine and indomethacin) | 1: MgSO4, N = 341 babies  2: No MgSO4, N = 2013 babies | PVH/IVH grade 3/4 | 10.0% vs. 15.1%; P < 0.01  (P relates to univariate analysis, considering tocolytic groups: 1: MgSO4, 2: no tocolysis, 3: ritodrine, and 4: indomethacin) | NR |
|  |  |  | PVH/IVH grade 3/4 (multivariate logistic regression analysis; considered for inclusion: tocolysis, antenatal steroids, multiple birth, PROM, amnionitis, mode of birth, GA, birthweight, Apgar score at 1 minute, Apgar score at 5 minutes, RDS, PDA, MV, pneumothorax, sepsis) | aOR 0.8; 95% CI 0.5-1.2 | ~ |
| Weisz 2015; RCS; moderate | N = 6015 babies; FN/PE/T/UK | 1: MgSO4 for FN, N = 1387 babies  2: No MgSO4, N = 3868 babies | Any resuscitation (mask CPAP or PPV, ETT intubation and ventilation, chest compressions or epinephrine) | P < 0.01 | 🗶 |
|  |  |  | CPAP only^ | P < 0.01 | 🗶 |
|  |  |  | Bag/mask or neopuff ventilation | P < 0.01 | ✓ |
|  |  |  | Intubation and ventilation | P = 0.03 | ✓ |
|  |  |  | Chest compressions | P < 0.01 | ✓ |
|  |  |  | Epinephrine (ETT or IV) | P = 0.02 | ✓ |
|  |  |  | Apgar score < 7 at 5 minutes | P = 0.20 | ~ |
|  |  |  | Surfactant use | P = 0.06 | ~ |
|  |  |  | SNAP-2 score > 20 | P = 0.28 | ~ |
|  |  |  | Intensive resuscitation (intubation and ventilation, or chest compressions or epinephrine administration in delivery room), unadjusted, and multiple logistic regression (with GEE to account for correlated data within each site/site effects), adjusted for: GA, sex, SGA, outborn status, chorioamnionitis, mode of birth, antenatal corticosteroid use, multiple gestation | OR 0.87; 95% CI 0.76-0.98; P = 0.02  aOR 0.88; 95% CI 0.66-1.17 | ✓, ~ |
|  |  |  | Neonatal death, unadjusted, and adjusted as above | OR 0.73; 95% CI 0.58-0.92; P < 0.01  aOR 0.61; 95% CI 0.40-0.94 | ✓, ✓ |
|  |  |  | BPD, unadjusted, and adjusted as above | OR 1.09; 95% CI 0.93-1.18; P = 0.28  aOR 1.13; 95% CI 0.92-1.38 | ~, ~ |
|  |  |  | NEC stage ≥ 2, unadjusted, and adjusted as above | OR 1.19; 95% CI 0.91-1.55; P = 0.20  aOR 0.99; 95% CI 0.73-1.34 | ~, ~ |
|  |  |  | IVH grade 3/4 or PVL, unadjusted, and adjusted as above | OR 0.95; 95% CI 0.79-1.15; P = 0.62  aOR 1.01; 95% CI 0.76-1.34 | ~, ~ |
|  |  |  | ROP stage ≥ 3, unadjusted, and adjusted as above | OR 1.01; 95% CI 0.74-1.36; P = 0.95  aOR 0.88; 95% CI 0.61-1.28 | ~, ~ |
|  |  |  | Sepsis, unadjusted, and adjusted as above | OR 1.09; 95% CI 0.92-1.29; P = 0.32  aOR 0.96; 95% CI 0.80-1.14 | ~, ~ |
|  |  |  | Composite outcome (morality or any major morbidity), unadjusted, and adjusted as above | OR 1.00; 95% CI 0.88-1.14; P = 0.97  aOR 1.03; 95% CI 0.83-1.29 | ~, ~ |
|  |  | 23-28 weeks GA  1: MgSO4 for FN, N = 731 babies  2: No MgSO4, N = 1813 babies | Intensive resuscitation, and adjusted as above | OR 0.79 (0.66 to 0.94); P < 0.01  aOR 0.89 (0.67 to 1.18) | ✓, ~ |
|  |  |  | Neonatal death, unadjusted, and adjusted as above | OR 0.65 (0.51 to 0.85); P < 0.01  aOR 0.65 (0.43 to 1.00) | ✓, ~ |
|  |  |  | BPD, unadjusted, and adjusted as above | OR 1.03 (0.85 to 1.25); P = 0.75  aOR 1.30 (1.03 to 1.65) | ~, ~ |
|  |  |  | NEC stage ≥ 2, unadjusted, and adjusted as above | OR 1.15 (0.84 to 1.57); P = 0.38  aOR 1.05 (0.78 to 1.42) | ~, ~ |
|  |  |  | IVH grade 3/4 or PVL, unadjusted, and adjusted as above | OR 0.96 (0.77 to 1.19); P = 0.69  aOR 1.11 (0.77 to 1.60) | ~, ~ |
|  |  |  | ROP stage ≥ 3, unadjusted, and adjusted as above | OR 0.96 (0.70 to 1.31); P = 0.79  aOR 0.86 (0.59 to 1.25) | ~, ~ |
|  |  |  | Sepsis, unadjusted, and adjusted as above | OR 0.97 (0.79 to 1.18); P = 0.76  aOR 0.91 (0.75 to 1.11) | ~, ~ |
|  |  |  | Composite outcome, unadjusted, and adjusted as above | OR 0.92 (0.77 to 1.09); P = 0.33  aOR 1.24 (0.97 to 1.61) | ~, ~ |
|  |  | 29-31 weeks GA  1: MgSO4 for FN, N = 656 babies  2: No MgSO4, N = 2055 babies | Intensive resuscitation, and adjusted as above | OR 0.67 (0.53 to 0.83); P < 0.01  aOR 0.82 (0.57 to 1.19) | ✓, ~ |
|  |  |  | Neonatal death, unadjusted, and adjusted as above | OR 0.44 (0.18 to 1.05); P = 0.06  aOR 0.74 (0.36 to 1.51) | ~, ~ |
|  |  |  | BPD, unadjusted, and adjusted as above | OR 0.67 (0.44 to 0.99); P = 0.05  aOR 0.73 (0.49 to 1.07) | ✓, ~ |
|  |  |  | NEC stage ≥ 2, unadjusted, and adjusted as above | OR 1.06 (0.63 to 1.78); P = 0.81  aOR 1.03 (0.57 to 1.85) | ~, ~ |
|  |  |  | IVH grade 3/4 or PVL, unadjusted, and adjusted as above | OR 0.67 (0.43 to 1.04); P = 0.07  aOR 0.66 (0.40 to 1.06) | ~, ~ |
|  |  |  | ROP stage ≥ 3, unadjusted, and adjusted as above | OR 1.53 (0.36 to 6.46); P = 0.56  aOR NC | ~, ~ |
|  |  |  | Sepsis, unadjusted, and adjusted as above | OR 1.23 (0.81 to 1.58); P = 0.48  aOR 1.23 (0.91 to 1.67) | ~, ~ |
|  |  |  | Composite outcome, unadjusted, and adjusted as above | OR 0.77 (0.59 to 1.01); P = 0.05  aOR 0.77 (0.59 to 1.01) | ~, ~ |
|  |  | 1: MgSO4 for FN, N = 1387 babies  2: MgSO4 for PE/T = 214 babies  3: MgSO4 for UK = 546 babies | Any resuscitation, 1 vs. 2; 1 vs. 3 | P = 0.97; P = 0.76 | ~, ~ |
|  |  |  | CPAP only, 1 vs. 2; 1 vs. 3^ | P < 0.01; P < 0.01 | 🗶, 🗶 |
|  |  |  | Bag/mask or neopuff ventilation, 1 vs. 2; 1 vs. 3 | P = 0.04; P = 0.35 | ✓, ~ |
|  |  |  | Intubation and ventilation, 1 vs. 2; 1 vs. 3 | P < 0.01; P < 0.28 | 🗶, ~ |
|  |  |  | Chest compressions, 1 vs. 2; 1 vs. 3 | P = 0.82; P = 0.60 | ~, ~ |
|  |  |  | Epinephrine (ETT or IV) , 1 vs. 2; 1 vs. 3 | P = 0.99; P = 0.54 | ~, ~ |
|  |  |  | Apgar score < 7 at 5 minutes, 1 vs. 2; 1 vs. 3 | P = 0.14; P = 0.12 | ~, ~ |
|  |  |  | Surfactant use, 1 vs. 2; 1 vs. 3 | P = 0.33; P = 0.53 | ~, ~ |
|  |  |  | SNAP-2 score > 20, 1 vs. 2; 1 vs. 3 | P < 0.01; P < 0.01 | ✓, ✓ |
|  |  | 1: MgSO4 for any indication, N = 2147 babies  2: No MgSO4, N = 3868 babies | Intensive resuscitation, and adjusted as above | OR 0.81; 95% CI 0.73-0.90; P < 0.01  aOR 0.87; 95% CI 0.69-1.13 | ✓, ~ |
|  |  |  | Neonatal death, unadjusted, and adjusted as above | OR 0.67; 95% CI 0.54-0.82; P < 0.01  aOR 0.64; 95% CI 0.46-0.89 | ✓, ✓ |
|  |  |  | BPD, unadjusted, and adjusted as above | OR 1.05; 95% CI 0.92-1.21; P = 0.44  aOR 1.11; 95% CI 0.94-1.30 | ~, ~ |
|  |  |  | NEC stage ≥ 2, unadjusted, and adjusted as above | OR 0.99; 95% CI 0.79-1.27; P = 0.99  aOR 0.89; 95% CI 0.768-1.17 | ~, ~ |
|  |  |  | IVH grade 3/4 or PVL, unadjusted, and adjusted as above | OR 0.86; 95% CI 0.73-1.01; P = 0.06  aOR 1.02; 95% CI 0.76-1.36 | ~, ~ |
|  |  |  | ROP stage ≥ 3, unadjusted, and adjusted as above | OR 0.99; 95% CI 0.76-1.29; P = 0.94  aOR 0.86; 95% CI 0.59-1.25 | ~, ~ |
|  |  |  | Sepsis, unadjusted, and adjusted as above | OR 1.12; 95% CI 0.97-1.30; P = 0.11  aOR 1.02; 95% CI 0.87-1.20 | ~, ~ |
|  |  |  | Composite outcome, unadjusted, and adjusted as above | OR 0.93; 95% CI 0.83-1.04; P = 0.19  aOR 1.03; 95% CI 0.85-1.24 | ~, ~ |
| Whitsel 2004; RCS; unclear  Abstract | N = 118 babies; NR | 1: MgSO4, N = NR  2: No MgSO4, N = NR | Neonatal death | “did not change the mortality rate” | ~ |
|  |  |  | Late bacterial sepsis | P = 0.046 | 🗶 |
| Whitten 2015; RCS with CCS(N); unclear  Abstract | N = 6791 babies; NR | 1: LOS ≤ 3 days^, N = 6472 babies  2: LOS ≥ 4 days, N = 319 babies | MgSO4 exposure | OR 6.72; P = 0.000 | 🗶 |
| Wiswell 1996; PCS; unclear  Abstractf | N = 137 babies; T/PIH | 1: MgSO4, N = 61 babies  2: No MgSO4, N = 76 babies | NEC | P = 0.042 | ✓ |
|  |  |  | ICH grade 3/4 | P = 0.009 | ✓ |
|  |  |  | cPVL in survivors ≥ 21 days | P = 0.088 | ~ |
|  |  |  | ICH grade 3/4 or cPVL | OR 4.1; 95% CI 2.0-8.5; P = 0.0001 | ✓ |
| Wutthigate 2017; PCS with CCS(N); high | N = 57 women, 63 babies; PIH/T | 1: Apnoeic episodes, N = 8 babies  2: No apnoeic episodes, N = 55 babies | MgSO4 dose (reported as mg/dL) (mean ± SD) | P = 0.06 | ~ |
| Yokoyama 2010; RCS; high | N = 117 babies; T | 1: MgSO4, N = 58 babies  2: No MgSO4, N = 59 babies | RDS | P = 0.709 | ~ |
|  |  |  | IVH | P = 0.496 | ~ |
|  |  |  | PDA | P = 0.829 | ~ |
|  |  |  | ROP | P = 0.053 | ~ |
|  |  |  | Neonatal death | P = 0.243 | ~ |
|  |  |  | NEC | No events | ~ |
|  |  |  | Bone change (abnormalities: osteopenic radiolucent bands at metaphyses of long bones; not suggestive of rickets) | P = 0.496 | ~ |
| Young 1977; NRT; high | N = 144 women and their babies; PE/E | 1: MgSO4 ‘push’ IV, N = 97 women and babies  2: MgSO4 continuous IV, N = 47 women and babies | Perinatal death | 1: 2.1% vs. 2: 2.1% | ~ |

^Indicates an outcome reported by a single study

Abbreviations: aOR: adjusted odds ratio; aRR: adjusted risk ratio; BMI: body mass index; BPD: bronchopulmonary dysplasia; CCS; case-control study; CCS(N): nested case-control study; CI: confidence interval; CMV: continuous mandatory ventilation; CPAP: continuous positive airway pressure; CPR: cardiopulmonary resuscitation; cPVL: cystic periventricular leucomalacia; CRIB: clinical risk index for babies; E: eclampsia; ETT: endotracheal tube; FN: fetal neuroprotection; g: grams; GA: gestational age; HD: hypertensive disorders; HFOV: high frequency oscillatory ventilation; HsPDA: haemodynamically significant patent ductus arteriosus; ICH: intracranial haemorrhage; IMV: intermittent mandatory ventilation; IV: intravenous; IVH: intraventricular haemorrhage; LD: loading dose; LOS: length of stay; LSV: lenticulostriate vasculopathy; MD: maintenance dose; MgSO4: magnesium sulphate; MRI: magnetic resonance imaging; N: number; MV: mechanical ventilation; N: number; NCCS: non-concurrent cohort study; NEC: necrotising enterocolitis; NICU: neonatal intensive care unit; NR: not reported; NRT: non-randomised trial; NS: non-significant; OR: odds ratio; P: p value; PCS: prospective cohort study; PDA: patent ductus arteriosus; PE: pre-eclampsia; PEA: parenchymal echo abnormality; PEL/VE: parenchymal lesions/ventricular enlargement; PIE: pulmonary interstitial emphysema; PIH: pregnancy-induced hypertension; ; PTL: preterm labour; PV-IVH: periventricular intraventricular haemorrhage; PVL: periventricular leucomalacia; PWML: punctate white matter lesions; RCS: retrospective cohort study; RCT: randomised controlled trial; RD: respiratory distress; RDS: respiratory distress syndrome; ROM: rupture of membranes; ROP: retinopathy of prematurity; RR: risk ratio; SCBU: special care baby unit; SD: standard deviation; SGA: small-for-gestational age; SH: systematic hypertension; SIP: spontaneous intestinal perforation; SNAP: Score For Neonatal Acute Physiology; SNI: severe neurological injury; sPDA: significant patent ductus arteriosus; T: tocolysis; TSV: thalamostriate or mineralising vasculopathy; VLBW: very low birthweight; WMI: white matter injury
