# Supplementary material for: Antenatal magnesium sulphate and adverse neonatal outcomes: A systematic review and meta-analysis
Source: PLoS Med. 2019 Dec 6;16(12):e1002988. doi: 10.1371/journal.pmed.1002988 (PMC6897495; doi:10.1371/journal.pmed.1002988)
Supplement: S4 Table — (DOCX) [file pmed.1002988.s007.docx]

**Detail of adverse outcomes from case reports**

| **Common adverse outcome** | **Authors’ conclusions** | **Setting** | **Neonatal characteristics** | **MgSO4 regimen** | **MgSO4** **indication** | **Study** |
| --- | --- | --- | --- | --- | --- | --- |
| **Neonatal death** | | | | | | |
| Twin A: Apgar scores 1/1/0; resuscitated (including intratracheal epinephrine receipt); death at 30 minutes of age  Twin B: Apgar scores 4/6/8; resuscitated (intubated); hypermagnesemic; elevated serum cardiac troponin T levels; non-specific ST-T wave abnormalities | “it is conceivable that the mechanism of death… may be directly related to the toxic effects of magnesium on the myocardium of twin A, whereas the myocardium of twin B, although compromised, was sufficiently functional to maintain life.” | USA  Year NR | 2 neonates; twins; preterm; LBW | 4 g IV LD; 2.5 g/hour MD for ~ 1 day; total dose: 51.4 g | T | Herschel 2001 |
| 42 neonates: diagnosis of poisoning; abnormal clinical signs (suck failure, cyanosis, hypoactivity, hypotonia, hyporeflexia); IV calcium gluconate receipt; death of 7/27 neonates exposed to MgSO_4_ as a single agent | “in fact only two (2.7%) patients died of the secondary effects of overdose of drugs (one of them due to magnesium sulfate.” | Turkey  1975 to 1997 | 42 neonates with a diagnosis of poisoning; exposed to MgSO_4_ (27 as a single agent) | 18 to 40 g IV for 12 to 48 hours prior to birth | PE/E | Kurtoglu 2000 |
| **Cardiopulmonary arrest after gentamicin exposure following hypermagnesemia at birth** | | | | | | |
| Respiratory arrest and cardiac arrest following IM gentamicin at 24 hours for suspected sepsis (in context of hypermagnesemia at birth with abnormal neuromuscular function tracings and clinical examination: peripheral motor weakness, rapid shallow respirations, poor suck, poor Moro reflex, decreased grasp reflexes) | “aminoglycosides may potentiate a magnesium-induced impairment of neuromuscular transmission and cause muscular weakness in neonates.” | USA  Year NR | 1 neonate; term | 24 g in 32 hours prior to birth | PE | L’Hommedieu 1983 |
| Respiratory and cardiac arrest following IV gentamicin at 48 hours for suspected sepsis (in context of hypermagnesemia at birth with neuromuscular compromise: ulnar nerve stimulation studies and neurologic examination) | “depressed NMF [neuromuscular function] in a hypermagnesemic infant may be further compromised by aminoglycoside therapy.” | USA  Year NR | 1 neonate; term | 28 g | PE | Rasch 1981 |
| **Clinical features of magnesium ‘toxicity’ or ‘intoxication’ at birth** | | | | | | |
| Flaccid, apnoeic, cyanotic at birth (muscular paralysis, absent reflexes, respiratory failure); Apgar score 2 at 1 minute; endotracheal intubation, ventilation and calcium gluconate receipt; hypermagnesemic | “Magnesium Intoxication… “in our patient, peak serum levels from the mother’s first intramuscular dose could have coincided with the peak serum level from her second intravenous dose… the toxic effects of motor and respiratory paralysis were immediately reversed when the serum magnesium level lowered during an exchange transfusion.” | USA  1966 | 1 neonate; very preterm; LBW | 3 g IV, 6 g IM, and 2 g IV, 3.5 hours, 2.5 hours, 0.5 hours prior to birth respectively | PE | Brady 1967 |
| Depressed, hypotonic, cyanotic, apnoeic, partially responsive to initial resuscitation; hypermagnesemic | “a newborn with a history of maternal use of magnesium sulfate, who presented hypermagnesemia.” | Chile  Year NR | 1 neonate; preterm | 17 hours IV prior to birth | PE | Cruz 2009 |
| “CNS disturbances” | “For appropriate treatment of neonates, it is necessary to take into consideration maternal medication use during pregnancy and delivery.” | USA  Year NR | ~25 neonates [225 neonates with RDS, 11% with symptoms of “Magnezium Sulfate intoxication”] | NR | NR | Jashi 2014 |
| “clinical and biochemical changes associated with hypermagnesemia in newborns”:  Neonate 1: low Apgar score at 1 minute (intubation, ventilation), flaccid, unresponsive, shallow respirations, respiratory arrest  Neonate 2: low Apgar score at 1 minute, bradycardia, minimal activity, weak cry  Neonate 3: low Apgar score at birth, hypotonia, hyporeflexia  Neonate 4: low Apgar score at 1 minute (intubation, ventilation), floppy, poor shallow respiratory movement, poor activity, weak cry  Neonates 5 and 6: low Apgar scores (intubation and ventilation), flaccid, death [not attributed to MgSO4; rather HMD]  Neonate 6: low Apgar at 1 minute (intubation and ventilation), flaccid, weak cry, poor reflexes, poor muscle tone | “clinical and biochemical changes associated with hypermagnesemia in newborns.” | USA  1965-1966 | 7 neonates [detailed case reports]; 3 term, 4 preterm (1 set of twins)  [Note: only detailed cases included; others presented as case series] | 1: 51.5 g in 33.5 hours  2: 21 g in 24 hours  3: 30 g in 19 hours  4: 31 g in 20 hours  5: 41.3 g in 30 hours  6: 60.4 g in 44 hours | PE/E | Lipsitz 1967 |
| Apgar scores 2/6, severe respiratory distress (intubated) at birth, hyporeflexic, hypotonic, lethargic; hypermagnesemic; hypocalcaemic; ventilated and IV calcium gluconate receipt | “Hypermagnesemia was judged to be the cause of the newborn’s clinical presentation.” | Taiwan  1989 | 1 neonate; preterm; LBW | 50 g IV in 46 hours prior to birth | PE | Teng 1989 |
| **Microcolon or ‘meconium-plug syndrome’** | | | | | | |
| Microcolon, with clinical signs and symptoms: failure to pass meconium within first 24 hours with progressive abdominal distention | “In summary, the microcolon seen in our very premature infants seems secondary to functional obstruction on the basis of ganglion cell dysfunction and depressive effects of magnesium sulfate.” | USA  1980 to 1984 | 4 neonates with non-organic intestinal obstruction; very preterm; VLBW | NR | PE | Amodio 1986 |
| No passage or delayed massage of meconium and abdominal distension; findings consistent with “meconium plug syndrome” | “The role of magnesium in depression smooth muscle cells and passing the placenta has been well documented and was contributory in some of our patients.” | USA  Year NR | 8 neonates; LBW/VLBW; preterm  [12 neonates not exposed to MgSO_4_ excluded] | NR | PE/E | Krasna 1996 |
| Abdominal distention and failure to pass meconium (“Meconium-plug syndrome”)  1 neonate: depressed, shallow respirations, hypotonic, hyporeflexic; hypermagnesemic; hypocalcaemic  1 neonate: lethargic, hypotonic, rapid and grunting respiration, lower extremities hypotonic, absent deep tendon reflexes; hypermagnesemic; hypocalcaemic; calcium gluconate IV receipt | “We believe that the hypermagnesemia depressed function of the intestinal smooth muscle as well as skeletal striated muscle in these babies.” | USA  Year NR | 2 neonates; 1 term, LBW; 1 preterm | 41 and 25 g in 24 hours prior to birth respectively | PE/E | Sokal 1972 |
| **Nonoliguric hyperkalaemia** | | | | | | |
| Neonatal non-oliguric hyperkalaemia (high risk for developing life-threatening cardiac arrhythmia) at 2 hours after birth; insufficient urinary K excretion; hypermagnesemic; not hypocalcaemic with calcium sulphate receipt; transiently hyponatraemic | “Maternal and fetal hypermagnesemia can induce rapidly progressive hyperkalemia in neonates. | Japan  Year NR | 1 neonate; preterm; VLBW | 0.1 g/hour IV on day 1; 0.5 g/hour IV on days 2 to 5; 1 g/hour IV days 6 to 12; 2 g/hour IV on day 12 | PE | Tanaka 2018 |
| **Bone abnormalities with prolonged MgSO_4_ for tocolysis** | | | | | | |
| Laboratory abnormalities (“especially hypermagnesemia and hypocalcemia”) and skeletal abnormalities including osteopenia and fractures | “The postmarket… data support an association between prolonged maternal administration of MgSO4 and neonatal hypermagnesemia, hypocalcemia, and skeletal abnormalities.” | USA  1986 to 2011 | 18 neonates; identified through the FDA Adverse Event Reporting System | Prolonged IV administration | Not clear (PE/T) | Ahmad 2013 |
| Radiographic changes in the metaphyses of long bones (discrete band of osteopenic metaphyseal bone); hypermagnesemic; not hypocalcaemic; elevated alkaline phosphatase | “we believe it likely the abnormal ossification was due to hypermagnesemia.” | USA  Year NR | 1 neonate; very preterm; LBW | 1 to 4 g/hour IV from ~18 weeks until birth at 28 weeks | T | Cumming 1989 |
| Diffuse osteopenia of long bones (diffuse metaphyseal osteopenia); probable rib fracture (2 neonates); hypermagnesemic; severely hypocalcemic; elevated alkaline phosphatase; relative hypoparathyroidism; calcium and calcitriol receipt (5 neonates) | “Premature infants who are exposed to large doses of MgSO4, especially those of multiple pregnancies, have an increased risk of developing hypocalcemia, osteopenia, and fractures.” | USA  Year NR | 10 neonates (twins or triplets); preterm; LBW or VLBW | Average mean parenteral dose: 3.66 (SD: 0.8) kg per pregnancy over a mean of 10.0 (SD: 0.5) weeks (1.75 to 3 g/hour for 9 to 11 weeks) | T | Kaplan 2006 |
| Triplets B and C: bone abnormalities “mimicking genetic bone disease”: at 3 weeks thin, demineralised bones with multiple fractures of the ribs, humeri and clavicle; skull bones were demineralised; very wide fontanelles, white sclera; not hypocalcaemic; mildly hypermagnesemic; elevated alkaline phosphatase; low bone density persisting at 3 months | “The bone abnormalities were presumed to be related to prolonged prenatal exposure to magnesium, with genetic bone disease unlikely… This case points out the necessity of including prolonged magnesium therapy in the differential diagnosis of the newborn infant with multiple fractures.” | NR | 2 neonates (from triplets); very preterm | 2.5 g/hour IV for 8.5 weeks | T | Kogan 2003 |
| Radiological, biochemical or clinical features of rickets  1 neonate: radiographic bony abnormalities: frank rachitic changes and dental enamel hypoplasia; hypotonic, respiratory distress at birth; hypermagnesemic; hypocalcaemic; elevated alkaline phosphatase; IV calcium gluconate receipt (dental enamel hypoplasia persisting at 3 years)  3 neonates: no bony abnormalities; mild respiratory distress; 1 hypotonic; hypermagnesemic; 1 hypocalcaemic | “We hypothesize that prolonged infusion of magnesium sulfate, especially when imitated during the second trimester, may lead to fetal parathyroid gland suppression with consequent abnormalities resembling rickets.” | USA  Year NR | 4 neonates; preterm  [1 neonate exposed to combination tocolysis excluded] | 1 neonate: 4 g IV LD; 2-3 g/hour IV MD for 13 weeks  3 neonates: doses similar to above, for 4 to 6 weeks | T | Lamm 1988 |
| Abnormal radiological findings: abnormal mineralisation of long-bone metaphyses  Twin 1A and 1B: hypotonic, poor sucking capacity; widening of anterior ends of ribs and widening of femora and humeri metaphyses with radiolucency; hypermagnesemic  Twin 2B: radiolucency of metaphyses, mainly humeri; hypermagnesemic | “abnormal radiological findings consisting of abnormal mineralisation of long-bone metaphyses owing to fetal hypermagnesaemia.”  Twin 2A not affected: “intrauterine growth restriction may be a protective factor against the development of bone abnormalities secondary to prolonged fetal hypermagnesaemia.” | Lebanon  Year NR | 3 neonates (from 2 sets of twins); preterm; LBW  [twin 2A VLBW, excluded] | Twins 1A and B: 3 to 3.5 g/hour IV for 12 weeks  Twins 2A and B: 3 to 4 g/hour IV for 8 weeks | T | Malaeb 2004 |

Abbreviations: CNS: central nervous system; E: eclampsia; g: grams; HMD: hyaline membrane disease; IM: intramuscular; IV: intravenous; LBW: low birthweight; LD: loading dose; MD: maintenance dose; MgSO_4_: magnesium sulphate; NR: not reported; PE: pre-eclampsia; RDS: respiratory distress syndrome; SD: standard deviation; T: tocolysis; USA: United States of America; VLBW: very low birthweight
