# Supplementary material for: Antenatal magnesium sulphate and adverse neonatal outcomes: A systematic review and meta-analysis
Source: PLoS Med. 2019 Dec 6;16(12):e1002988. doi: 10.1371/journal.pmed.1002988 (PMC6897495; doi:10.1371/journal.pmed.1002988)
Supplement: S1 Text — (PDF) [file pmed.1002988.s008.pdf]

**Project Title**
Neonatal and infant adverse effects of antenatal magnesium sulphate for improving outcomes for mothers and babies: a systematic review

**Chief Investigator**	
Name	Emily Bain
**Co-Investigator**	
Name	Philippa Middleton
**Co-Investigator**	
Name	Maria Makrides
**Co-Investigator**	
Name	Caroline Crowther

Research Plan

BACKGROUND

Antenatal magnesium sulphate for cerebral palsy prevention

Two landmark observational studies published in the 1990s provided the first descriptions of an association between in utero magnesium sulphate exposure and a reduced risk of cerebral palsy.[1,2] Due to the limitations and discrepancies of findings from observational studies, a need to establish reliable evidence, through the conduct of randomised trials, was realised. From 1995 to 2004, five placebo-controlled randomised trials (ACTOMgSO4;[3] BEAM;[4] MagNET;[5] PREMAG[6]; MAGPIE[7]) were conducted, testing the hypotheses that antenatal magnesium sulphate reduces brain injury, cerebral palsy, or mortality for preterm infants.

In the meta-analysis of the aforementioned trials in the 2009 Cochrane review, magnesium sulphate administered with neuroprotective intent reduced the risk of death or cerebral palsy (RR 0.85, 95% CI 0.74-0.98; 4 trials, 4,446 infants).[8] Overall magnesium sulphate reduced the risk of cerebral palsy (RR 0.68, 95% CI 0.54-0.87; 5 trials, 6,145 infants). This review confirmed the neuroprotective role for magnesium sulphate – 63 babies (95% CI 44-155) need to be exposed to magnesium sulphate in utero to benefit one preterm baby by avoiding cerebral palsy.[8]

Additional systematic reviews and meta-analyses have reached similar conclusions.[9,10] This has been regarded as a very important finding, as few interventions have been found to prevent the devastating consequences of cerebral palsy. Importantly, in a recent cost-effectiveness analysis, magnesium sulphate prior to very preterm birth was shown to be cost-effective from a societal and health system perspective.[11]

Following this compelling evidence from randomised trials and reviews, in many countries, including Australia, New Zealand, the United Kingdom, Canada and the United States, clinical practice guidelines[12,13] and opinion papers[14-17] have recommended use of this therapy. Australian and New Zealand clinical practice guidelines, *Antenatal magnesium sulphate prior to preterm birth for neuroprotection of the fetus, infant and child,* endorsed by the National Health and Medical Research Council (NHMRC) in 2010, provided guidance in the form of nine evidence-based recommendations and six practice points.[12]

With passive guideline dissemination alone, it was not anticipated that all health professionals would immediately begin using antenatal magnesium sulphate.[18] The WISH Project (*Working to Improve Survival and Health for babies born very preterm*) was thus designed, funded by the Cerebral Palsy Alliance Research Foundation, to improve and monitor the use of this therapy in Australia and New Zealand.[19] Through WISH, audit studies at lead hospitals have highlighted rapid translation – at the Women's and Children's Hospital, South Australia uptake increased from 30% in 2010, to 78% in 2011-13,[20,21] and in 2012, uptake at Auckland City Hospital, New Zealand, was 82%.[22] While encouragingly in 2011, 76%, and in 2013, almost all Australian and New Zealand tertiary maternity hospitals had local practices to implement this therapy, fewer were formally auditing use, and uptake estimates have varied.[23,24] The Australian and New Zealand Neonatal Network (ANZNN) data report increasing uptake bi-nationally (2012: 44%; 2013: 60%).[25,26]

Maternal adverse effects of antenatal magnesium sulphate: a systematic review

Given the extensive use of antenatal magnesium sulphate in obstetric care, to prevent or treat pre-eclampsia (beneficial),[27,28] for acute and maintenance tocolytic therapy for women during and following threatened preterm labour (not beneficial),[29,30] and most recently, for fetal neuroprotection and cerebral palsy prevention (beneficial),[8] the potential adverse effects for the mother are well known.

High quality evidence regarding maternal adverse effects can be drawn from a recent systematic review (led by CI Bain), *'Maternal adverse effects of different antenatal magnesium sulphate regimens for improving maternal and infant outcomes: a systematic review'*.[31] This review included 143 publications – 21 randomised controlled trials, 14 non-randomised comparative studies, 32 case series, and 74 reports of individual cases – reporting adverse effects of magnesium sulphate when given for treatment of pre-eclampsia, for cerebral palsy prevention, or for preterm labour tocolysis. Reassuringly, this review showed that antenatal magnesium sulphate was not associated with an increased risk of maternal death, cardiac arrest or respiratory arrest.[31] In this review, individual case reports, did, however support an association between iatrogenic overdose of magnesium sulphate and life-threatening consequences.[31,32] Appropriate administration of therapy was shown to increase

the risk of 'any adverse maternal effects' (RR 4.62, 95% CI 2.42 to 8.83; 4 trials, 13,322 women), and treatment cessation due to maternal adverse effects (RR 2.77, 95% CI 2.32 to 3.30; 5 trials, 13,666 women).[31] This systematic review concluded that for each antenatal indication for use, further trials designed to determine optimal regimens (aimed at achieving maximal effectiveness with minimal adverse effects) may be beneficial, and called for vigilance in the use of this therapy, in order to ensure women's safety.[31]

Potential neonatal or infant adverse effects of antenatal magnesium sulphate

With the increased, widespread use of antenatal magnesium sulphate for cerebral palsy prevention, concern has been raised about potential unintended neonatal or infant adverse effects. In a recent evaluation of barriers and enablers to implementing antenatal magnesium sulphate for cerebral palsy prevention (as part of The WISH Project), the uncertainty surrounding adverse effects for very preterm neonates was specifically raised by health professionals, particularly neonatologists, as a potential barrier to increased use.[33]

Magnesium has fundamental roles in many cellular process (such as gating of calcium channels; muscle contraction; neuronal activity; control of vasomotor tone; cardiac excitability; and neurotransmitter release)[34], and thus above normal magnesium concentrations (associated with magnesium sulphate therapy), can plausibly be associated with fetal, neonatal or infant adverse effects. Magnesium is known to cross the placenta readily, with fetal serum concentrations correlated with maternal serum magnesium concentrations,[35] and/or total maternal dose of magnesium sulphate given.[36] Where there is delayed fetal urinary excretion, fetal serum and amniotic fluid concentrations can exceed maternal concentrations.

In the Cochrane systematic reviews assessing magnesium sulphate for treating pre-eclampsia,[27,28] for cerebral palsy prevention,[8] and for preterm labour tocolysis,[29] no clear increased risks of adverse neonatal or infant outcomes were reported, however a borderline increased risk of total death (fetal, neonatal, infant) with prolonged use for tocolysis was observed (RR 4.56, 95% CI 1.00 to 20.86; 2 trials, 257 babies).[29] These reviews were restricted to assessing randomised controlled trial evidence, and a limited number of pre-specified outcomes.

Recently, a systematic review (including randomised trials and prospective observational studies) summarised the effects of magnesium sulphate given for treating pre-eclampsia, for cerebral palsy prevention, and for preterm labour tocolysis specifically on fetal heart rate.[37] This review suggested a small negative effect on fetal heart rate, variability and accelerative pattern, *"not sufficient clinically to warrant medical intervention".*[37] To date, a comprehensive systematic review of all available evidence surrounding potential unintended neonatal or infant adverse effects of antenatal magnesium sulphate has not been conducted.

While secondary analyses from the BEAM trial (one of the five randomised trials assessing magnesium sulphate for cerebral palsy prevention) have shown no clear effects of magnesium sulphate on neonatal resuscitation,[38,39] and other cardiovascular parameters in the first 24 hours of life,[40] numerous published observational studies have indicated higher risks of some adverse outcomes, necessitating further evaluation.

For example, a retrospective cohort study (of 6,654 women and their babies) has shown increasing maternal serum magnesium concentrations (when given for pre-eclampsia) to be associated with lower one and five-minute Apgar scores, and higher risks of intubation in the delivery room, admission to special care nursery and neonatal hypotonia.[41] Two retrospective cohort studies (of 160 neonates born at less than 28 weeks gestation; and 954 neonates born 500 to 1000 g), and one prospective case-control study (of 48 neonates born 30 to 34 weeks gestation), have shown antenatal magnesium sulphate exposure (when given for pre-eclampsia or for tocolysis) to be associated with an increased risk of neonatal patent ductus arteriosus.[42-44] A recent retrospective cohort study (of 155 neonates born less than 1000 g) has shown magnesium sulphate (when given for cerebral palsy prevention), to be associated with spontaneous intestinal perforation; with higher doses associated with spontaneous intestinal perforation and death among infants with the lowest birthweights.[45] Observational studies (including retrospective cohort studies and case reports) have shown prolonged magnesium sulphate exposure (when given for tocolysis) to be associated with abnormal bone metabolism for the neonate or infant,[46] and rarely, bone fracture at birth.[47]

Why it is important to conduct a systematic review of adverse effects for the neonate or infant

Strong evidence from randomised trials and systematic reviews supports the use of antenatal magnesium sulphate for cerebral palsy prevention.[3-6,8,48] Rapid translation into clinical practice across Australia and New

Zealand has been observed.[20,23,24] Concerns have however been raised surrounding potential neonatal and infant adverse effects of therapy, warranting further evaluation. A systematic review of all available evidence is thus now required in order to determine whether such adverse effects are associated with magnesium sulphate therapy, and if so, whether they vary according to factors such as different regimens for administration, or indications for use. Implementation of antenatal magnesium sulphate for cerebral palsy prevention can be strengthened, and safety improved, if clinical practice guidelines and their recommendations can be based on such knowledge.

AIMS

The primary aim of this systematic review is to assess whether antenatal exposure to magnesium sulphate, given to improve outcomes for mothers or their babies, including for cerebral palsy prevention, is associated with adverse effects for the neonate or infant.

Secondary aims are to assess whether there are variations in adverse effects according to pregnancy and birth characteristics for the women/neonate (such as: indication for use of antenatal magnesium sulphate, antenatal co-interventions, mode of birth, gestational age at birth, and birthweight) or characteristics of the magnesium sulphate regimen received by women (such as: dose, route of administration, timing before birth, and duration of treatment).

METHODS

Research design

Systematic review of adverse effects.

Criteria for considering studies for inclusion

Studies

We will include interventional (e.g. randomised, cluster-randomised, quasi-randomised and non-randomised comparative trials) and observational studies (e.g. cohort, case-control and cross-sectional studies, case series and case reports). We will include studies available as abstract only, along with full-text publications.

Participants

We will include neonates or infants who were exposed to antenatal magnesium sulphate, regardless of their gestational age at exposure or birth.

Interventions and comparators

We will include studies where antenatal magnesium sulphate was given for pre-eclampsia or eclampsia, for tocolysis to women in or following threatened preterm labour, or for neuroprotection of the fetus to women at risk of preterm birth (for cerebral palsy prevention). We will exclude studies where oral magnesium sulphate was given, and where magnesium sulphate was given as an adjuvant during anaesthesia. We will include studies in which magnesium sulphate was compared to no treatment, placebo, or a different magnesium sulphate regimen; and/or, for observational studies, we will include studies in which magnesium sulphate was assessed as an 'exposure.'

Outcomes

Primary: perinatal mortality (defined as stillbirth, neonatal or infant mortality up to the time of primary hospital discharge).

Secondary outcomes for the neonate or infant will be comprehensive, including anticipated or unanticipated adverse effects, such as:

- Stillbirth
- Neonatal or infant death
- Low Apgar scores at one and five minutes

- Need for active resuscitation at birth
- Neonatal respiratory depression
- Neonatal hypotension
- Need for neonatal nursery admission and duration of admission
- Lethargy, hypotonia, or hyporeflexia
- Patent ductus arteriosus
- Spontaneous intestinal perforation
- Osteopenia or bone fractures
- Other unanticipated adverse effects

Search methods

A comprehensive search of the following bibliographic databases will be performed: MEDLINE (PubMed/OVID which includes the Cochrane Library), EMBASE (OVID), CINAHL, Lilacs, Scopus, ISI Web of Science and Toxline, from their inceptions. We will use a combination of MeSH and free-text terms, and will not apply date or language restrictions.

We will search research registers of ongoing trials (clinicaltrials.gov, international clinical trials registry), and search engines Google Scholar and Google using key words. The reference lists of eligible articles or reviews identified will be checked for additional references.

Data collection and synthesis

The methodology for data collection and synthesis will be based on current recommendations of the Cochrane Adverse Effects Methods Group.[49] Where appropriate, the overview will be prepared using CAST[50] and Review Manager Software.[51]

Study selection

After screening all titles and abstracts, we will obtain the full-text article for any study which seems to meet the inclusion criteria based on the title and/or abstract, along with any reviews that may provide relevant references. Each stage will be carried out by two reviewers. We will resolve any discrepancies through discussion, or if required, we will consult a third reviewer.

Data extraction and management

Once a study is included, data will be extracted using a standardised form. Data extracted will include information regarding study design, participants, the magnesium sulphate regimen(s), the control or comparison if applicable, neonatal or infant adverse effects reported and results relevant to the review, the risk of bias, confounding and relevance. Extraction will be carried out by two reviewers independently. We will resolve any discrepancies through discussion, or if required, we will consult a third reviewer.

Quality assessment

The levels of evidence will be assessed using the Australian National Health and Medical Council Levels of Evidence, and the quality (risk of bias) of randomised trials assessed using established guidelines provided in the Cochrane Handbook.[52] The quality assessment of non-randomised studies will be based on recommendations from Cochrane (ACROBAT-NRSI).[53] As it has been suggested that the quality of adverse effect detection and reporting is not always adequately assessed, the methods used to detect adverse effects and how rigorous these methods were, will also be considered, along with incomplete reporting.[54,55]

Data synthesis

The analysis and presentation of results will be categorised by study design, and according to indication for use. Statistical analyses will be performed using Review Manager.[51]

For interventional studies we will present quantitative data from individual studies where possible as risk ratios (RR) with 95% confidence intervals (CI) for dichotomous outcomes. For all outcomes, we will carry out analyses as far as possible on an intention-to-treat basis. Pooled estimates (summary RR with 95% CI) will be calculated

using fixed-effect meta-analysis where there is a sufficient quantity of data, with clinical homogeneity. Where we consider that there is clinical heterogeneity sufficient to expect that the underlying effects differ between trials, or there is substantial statistical heterogeneity (where I^2 is greater than 30% and either Tau^2 is greater than zero, or there is a low P value (less than 0.10) in the Chi^2 test), summary estimates will be calculated using random-effects meta-analysis.

Separate comparisons will be performed for those studies assessing magnesium sulphate versus no treatment or placebo, and those comparing different magnesium sulphate regimens.

For observational studies (e.g. cohort, case-control and cross-sectional studies, and case series) we will present effect estimates where possible as percentages, RR or odds ratios (OR) with 95% CIs, or adjusted RR or OR if reported with 95% CIs, in tabular format based on study type; we will use narrative synthesis to summarise the results. Data from case reports will be tabulated, subsequently grouped according to common outcomes, and summarised narratively.

Analysis of subgroups

Subgroup analyses will be carried out if sufficient data are available based on pregnancy and birth characteristics, and characteristics of the magnesium sulphate regimens:

Characteristics of the pregnancy and birth

- Indication for magnesium sulphate administration (e.g. fetal neuroprotection and cerebral palsy prevention, pre-eclampsia/eclampsia treatment, preterm labour tocolysis, other)
- Gestational age at magnesium sulphate administration (e.g. < 28, 28 to < 32, 32 to < 37, ≥ 37 weeks gestation)
- Birthweight (e.g. < 500, 500 to < 1000, 1000 to < 1500, 1500 to < 2500, 2500 to < 4000, ≥ 4000 g)
- Mode of birth (vaginal birth, caesarean section)
- Concomitant maternal treatment including: antenatal or intrapartum drug therapy (e.g. corticosteroids, tocolytic agents, agents for pain relief).

Characteristics of the magnesium sulphate regimen

- Route of administration of magnesium sulphate (intravenous (IV), intramuscular (IM))
- Concentrated solution requiring dilution or pre-mixed solution
- Dose of magnesium sulphate
    - Loading dose only, loading and maintenance dose, maintenance dose only
    - Loading dose: none, ≤ 4 g, > 4 g
    - Maintenance dose: none, ≤ 1 g/hour IV, > 1 g/hour IV (OR none, ≤ 10 g IM every 4 hours, > 10 g IM every 4 hours)
    - Total dose prior to birth (< 4, 4 to < 14, 14 to < 28, ≥ 28 g)
    - Interval between treatment and birth (0 to < 1, 1 to < 4, 4 to < 8, 8 to < 12, 12 to < 24, ≥ 24 hours)

References

1. Nelson KB, Grether JK. Can magnesium sulfate reduce the risk of cerebral palsy in very low birthweight infants? *Pediatrics* 1995; **95**(2): 263-9.
2. Kuban KCK, Leviton A, Pagano M, Fenton T, Strassfeld R, Wolff M. Maternal toxemia is associated with reduced incidence of germinal matrix hemorrhage in premature babies. *J Child Neurol* 1992; **7**(1): 70-6.
3. Crowther CA, Hiller JE, Doyle LW, Haslam RR. Effect of magnesium sulfate given for neuroprotection before preterm birth: a randomized controlled trial. *JAMA* 2003; **290**(20): 2669-76.
4. Rouse DJ, Hirtz DG, Thom E, et al. A randomized, controlled trial of magnesium sulfate for the prevention of cerebral palsy. *N Engl J Med* 2008; **359**(9): 895-905.
5. Mittendorf R, Dambrosia J, Pryde PG, et al. Association between the use of antenatal magnesium sulfate in preterm labor and adverse health outcomes in infants. *Am J Obstet Gynecol* 2002; **186**(6): 1111-8.
6. Marret S, Marpeau L, Follet-Bouhamed C, et al. [Effect of magnesium sulphate on mortality and neurologic morbidity of the very-preterm newborn (of less than 33 weeks) with two-year neurological outcome: results of the prospective PREMAG trial]. *Gynecol Obstet Fertil* 2008; **36**(3): 278-88.
7. Altman D, Carroli G, Duley L, et al. Do women with pre-eclampsia, and their babies, benefit from magnesium sulphate? The Magpie Trial: a randomised placebo-controlled trial. *Lancet* 2002; **359**(9321): 1877-90.
8. Doyle LW, Crowther CA, Middleton P, Marret S, Rouse D. Magnesium sulphate for women at risk of preterm birth for neuroprotection of the fetus. *Cochrane Database Syst Rev* 2009; **1**: CD004661.
9. Costantine MM, Weiner SJ. Effects of antenatal exposure to magnesium sulfate on neuroprotection and mortality in preterm infants: a meta-analysis. *Obstet Gynecol* 2009; **114**(2 Pt 1): 354-64.
10. Conde-Agudelo A, Romero R. Antenatal magnesium sulfate for the prevention of cerebral palsy in preterm infants less than 34 weeks' gestation: a systematic review and metaanalysis. *Am J Obstet Gynecol* 2009; **200**(6): 595-609.
11. Bickford CD, Magee LA, Mitton C, et al. Magnesium sulphate for fetal neuroprotection: a cost-effectiveness analysis. *BMC Health Serv Res* 2013; **13**: 527.
12. The Antenatal Magnesium Sulphate for Neuroprotection Guideline Development Panel. Antenatal Magnesium Sulphate Prior to Preterm Birth for Neuroprotection of the Fetus, Infant and Child: National Clinical Practice Guidelines. Adelaide: The University of Adelaide, 2010.
13. Magee L, Sawchuck D, Synnes A, von Dadelszen P, Magnesium Sulphate for Fetal Neuroprotection Consensus Committee. SOGC Clinical Practice Guideline. Magnesium sulphate for fetal neuroprotection. *J Obstet Gynaecol Can* 2011; **33**(5): 516-29.
14. Mercer BM, Merlino AA, Society for Maternal-Fetal Medicine. Magnesium sulfate for preterm labor and preterm birth. *Obstet Gynecol* 2009; **114**(3): 650-68.
15. American College of Obstetricians and Gynecologists Committee on Obstetric Practice, Society for Maternal-Fetal Medicine. Committee Opinion No. 455: Magnesium sulfate before anticipated preterm birth for neuroprotection. *Obstet Gynecol* 2010; **115**(3): 669-71.
16. Reeves SA, Gibbs RS, Clark SL. Magnesium for fetal neuroprotection. *Am J Obstet Gynecol* 2011; **204**(3): 202.e1-4.
17. Royal College of Obstetricians & Gynaecologists. Magnesium sulphate to prevent cerebral palsy following preterm birth. RCOG Scientific Impact Paper 29. 2011. https://www.rcog.org.uk/ (accessed 19 February 2015).
18. Penney G, Foy R. Do clinical guidelines enhance safe practice in obstetrics and gynaecology? *Best Pract Res Clin Obstet Gynaecol* 2007; **21**(4): 657-73.
19. Crowther C, Middleton P, Bain E, et al. Working to improve survival and health for babies born very preterm: the WISH project protocol. *BMC Pregnancy Childbirth* 2013; **13**(1): 239.
20. Siwicki K, Bain E, Bubner T, Ashwood P, Middleton P, Crowther CA. Nonreceipt of antenatal magnesium sulphate for fetal neuroprotection at the Women's and Children's Hospital, Adelaide 2010-2013. *Aust N Z J Obstet Gynaecol* 2015; **55**(3): 233-8.
21. Bain E, Ashwood P, Middleton P, et al. Rapid implementation of antenatal magnesium sulphate for fetal neuroprotection at the WCH, Adelaide, Australia (2009-2012). *J Paediatr Child Health* 2013; **49**(Suppl 2): OP036.
22. Tan YH, Groom KM. A prospective audit of the adherence to a new magnesium sulphate guideline for the neuroprotection of infants born less than 30 weeks' gestation. *Aust N Z J Obstet Gynaecol* 2015; **55**(1): 90-3.
23. Bain E, Bubner T, Ashwood P, Crowther CA, Middleton P, The WPT. Implementation of a clinical practice guideline for antenatal magnesium sulphate for neuroprotection in Australia and New Zealand. *Aust N Z J Obstet Gynaecol* 2013; **53**(1): 86-9.

24. Middleton P, Bain E, Ashwood P, et al. Implementation progress of a clinical practice guideline for antenatal magnesium sulphate for neuroprotection in Australia and New Zealand. *J Paediatr Child Health* 2013; **49**(Suppl 2): A117.
25. Chow SSW. Report of the Australian and New Zealand Neonatal Network 2012. Sydney: ANZNN, 2014.
26. Chow SSW, Le Marsney R, S. H, Haslam R, Lui K. Report of the Australian and New Zealand Neonatal Network 2013. Sydney: ANZNN, 2015.
27. Duley L, Gulmezoglu AM, Henderson-Smart DJ, Chou D. Magnesium sulphate and other anticonvulsants for women with pre-eclampsia. *Cochrane Database Syst Rev* 2010; **11**: CD000025.
28. Duley L, Matar HE, Almerie MQ, Hall DR. Alternative magnesium sulphate regimens for women with pre-eclampsia and eclampsia. *Cochrane Database Syst Rev* 2010; **8**: CD007388.
29. Crowther CA, Brown J, McKinlay CJ, Middleton P. Magnesium sulphate for preventing preterm birth in threatened preterm labour. *Cochrane Database Syst Rev* 2014; **8**: CD001060.
30. Han S, Crowther CA, Moore V. Magnesium maintenance therapy for preventing preterm birth after threatened preterm labour. *Cochrane Database Syst Rev* 2013; **5**: CD000940.
31. Bain E, Middleton P, Crowther C. Maternal adverse effects of different antenatal magnesium sulphate regimens for improving maternal and infant outcomes: a systematic review. *BMC Pregnancy Childbirth* 2013; **13**(1): 195.
32. Simpson KR, Knox GE. Obstetrical accidents involving intravenous magnesium sulfate: recommendations to promote patient safety. *MCN Am J Matern Child Nurs* 2004; **29**(3): 161-9; quiz 70-1.
33. Bain E, Bubner T, Ashwood P, et al. Barriers and enablers to implementing antenatal magnesium sulphate for fetal neuroprotection guidelines: a study using the theoretical domains framework. *BMC Pregnancy Childbirth* 2015; **15**: 176.
34. Fawcett W, Haxby E, Male D. Magnesium: physiology and pharmacology. *Br J Anaesth* 1999; **83**(2): 302-20.
35. Sherwin CM, Balch A, Campbell SC, et al. Maternal magnesium sulphate exposure predicts neonatal magnesium blood concentrations. *Basic Clin Pharmacol Toxicol* 2014; **114**(4): 318-22.
36. Borja-Del-Rosario P, Basu SK, Haberman S, Bhutada A, Rastogi S. Neonatal serum magnesium concentrations are determined by total maternal dose of magnesium sulfate administered for neuroprotection. *J Perinat Med* 2014; **42**(2): 207-11.
37. Nensi A, De Silva DA, von Dadelszen P, et al. Effect of magnesium sulphate on fetal heart rate parameters: a systematic review. *J Obstet Gynaecol Can* 2014; **36**(12): 1055-64.
38. Drassinower D, Friedman AM, Levin H, Obican SG, Gyamfi-Bannerman C. Does magnesium exposure affect neonatal resuscitation? *Am J Obstet Gynecol* 2015.
39. Johnson LH, Mapp DC, Rouse DJ, et al. Association of cord blood magnesium concentration and neonatal resuscitation. *J Pediatr* 2012; **160**(4): 573-7 e1.
40. Paradisis M, Osborn DA, Evans N, Kluckow M. Randomized controlled trial of magnesium sulfate in women at risk of preterm delivery-neonatal cardiovascular effects. *J Perinatol* 2012; **32**(9): 665-70.
41. Abbassi-Ghanavati M, Alexander JM, McIntire DD, Savani RC, Leveno KJ. Neonatal effects of magnesium sulfate given to the mother. *Am J Perinatol* 2012; **29**(10): 795-9.
42. Shokry M, Elsedfy GO, Bassiouny MM, Anmin M, Abozid H. Effects of antenatal magnesium sulfate therapy on cerebral and systemic hemodynamics in preterm newborns. *Acta Obstet Gynecol Scand* 2010; **89**(6): 801-6.
43. Del Moral T, Gonzalez-Quintero V, Claure N, Vanbuskirk S, Bancalari E. Antenatal exposure to magnesium sulfate and the incidence of patent ductus arteriosus in extremely low birth weight infants. *J Perinatol* 2007; **27**(3): 154-7.
44. Katayama Y, Minami H, Enomoto M, Takano T, Hayashi S, Lee YK. Antenatal magnesium sulfate and the postnatal response of the ductus arteriosus to indomethacin in extremely preterm neonates. *J Perinatol* 2011; **31**(1): 21-4.
45. Rattray BN, Kraus DM, Drinker LR, Goldberg RN, Tanaka DT, Cotten CM. Antenatal magnesium sulfate and spontaneous intestinal perforation in infants less than 25 weeks gestation. *J Perinatol* 2014; **34**(11): 819-22.
46. Yokoyama K, Takahashi N, Yada Y, et al. Prolonged maternal magnesium administration and bone metabolism in neonates. *Early Hum Dev* 2010; **86**(3): 187-91.
47. Wedig KE, Kogan J, Schorry EK, Whitsett JA. Skeletal demineralization and fractures caused by fetal magnesium toxicity. *J Perinatol* 2006; **26**(6): 371-4.
48. Mittendorf R DJ, Pryde PG, Lee K-S, Gianopoulois JG, Besinger RE, Tomich PG. Association between the use of antenatal magnesium sulfate in preterm labor and adverse health outcomes in infants. *Am J Obstet Gynecol* 2002; **186**(6): 1111-8.

49. Cochrane Methods Adverse Effects. Cochrane Methods Adverse Effects, Trusted evidence. Informed decisions. Better Health. 2015. http://aem.cochrane.org/ (accessed 15 August 2015).
50. Cochrane Informatics & Knowledge Management Department. Cochrane Author Support Tool. 2015. http://tech.cochrane.org/our-work/cochrane-author-support-tool (accessed 15 August 2015).
51. The Nordic Cochrane Centre, The Cochrane Collaboration. Review Manager (RevMan). 5.1. Copenhagen: The Nordic Cochrane Centre, The Cochrane Collaboration; 2011.
52. Higgins J, Green S. Cochrane Handbook for Systematic Reviews of Interventions Version 5.1.0 [updated March 2011]. The Cochrane Collaboration; 2011.
53. Sterne J, Higgins J, Reeves B, on behalf of the development group for ACROBAT-NRSI. A Cochrane Risk Of Bias Assessment Tool: for Non-Randomized Studies of Interventions (ACROBAT-NRSI) Version 1.0.0, 24 September 2014. Available from http://www.riskofbias.info [accessed 15 August 2015].
54. Loke YK, Price D, Herxheimer A. Systematic reviews of adverse effects: framework for a structured approach. *BMC Med Res Methodol* 2007; **7**(1): 32.
55. Zorzela L, Golder S, Liu Y, et al. Quality of reporting in systematic reviews of adverse events: systematic review. *BMJ* 2014; **348**.
