# Supplementary material for: Antenatal magnesium sulphate and adverse neonatal outcomes: A systematic review and meta-analysis
Source: PLoS Med. 2019 Dec 6;16(12):e1002988. doi: 10.1371/journal.pmed.1002988 (PMC6897495; doi:10.1371/journal.pmed.1002988)
Supplement: S2 Text — (DOCX) [file pmed.1002988.s009.docx]

**Database search strategies**

**CINAHL**

Date searched: 30/05/2016; with top-up searches conducted on 10/08/2018 and 03/09/2019

Total records retrieved: **422**

1. (MH "magnesium sulfate+")
2. “magnesium sulfate” or “magnesium sulphate” or mgso4
3. S1 or S1
4. (MH “Prenatal Care”)
5. (MH “Pregnancy+”)
6. (MH “Perinatal Care”)
7. (MH “Maternal Exposure”)
8. (pregnan* or labor or laboring or labour* or antepart* or prenatal* or antenatal* or perinatal* or intranatal* or obstetric* or intrapart* or preterm or prematur* or tocoly* or “maintenance therapy” or preeclamp* or pre-eclamp* or “pre eclamp*” or eclamp* or neuroprotection* or “cerebral palsy”)
9. S4 OR S5 OR S6 OR S7 OR S8
10. (MH "Infant+")
11. (MH "Infant, Newborn, Diseases+")
12. (MH "Intensive Care, Neonatal+")
13. (fetus* or fetal* or foetus* or foetal* or baby or babies or neonat* or infan* or newborn* or “new born”)
14. S10 OR S11 OR S12 OR S13
15. S3 AND S9 AND S14

**Cochrane Library**

Date searched: 30/05/2016; with top-up search conducted on 10/08/2018 and 03/09/2019

Total records retrieved: **494**

1. MeSH descriptor: [Magnesium Sulfate] explode all trees
2. ((magnesium next sulfate) or (magnesium next sulphate) or MgSO4):ti,ab,kw
3. #1 or #2
4. MeSH descriptor: [Pregnancy] explode all trees
5. MeSH descriptor: [Pregnancy Complications] explode all trees
6. MeSH descriptor: [Prenatal Care] explode all trees
7. MeSH descriptor: [Perinatal Care] explode all trees
8. MeSH descriptor: [Maternal Exposure] explode all trees
9. (pregnan* or labor or laboring or labour* or antepart* or prenatal* or antenatal* or perinatal* or intranatal* or obstetric* or intrapart* or preterm or prematur* or tocoly* or (maintenance NEXT therapy) or preeclamp* or pre-eclamp* or (pre NEXT eclamp*) or eclamp* or neuroprotection* or (cerebral NEXT palsy)):ti,ab,kw
10. #4 or #5 or #6 or #7 or #8 or #9
11. MeSH descriptor: [Infant] explode all trees
12. MeSH descriptor: [Infant, Newborn, Diseases] explode all trees
13. MeSH descriptor: [Intensive Care, Neonatal] explode all trees
14. (fetus* or fetal* or foetus* or foetal* or baby or babies or neonat* or infan* or newborn* or (new NEXT born)):ti,ab,kw
15. #11 or #12 or #13 or #14
16. #3 and #10 and #15

**LILACS**

Date searched: 30/05/2016; with top-up search conducted on 10/08/2018 and 03/09/2019

Total records retrieved: **58**

("magnesium sulphate" or "magnesium sulfate" or mgso4) and (fetus$ or fetal$ or foetus$ or foetal$ or baby or babies or neonat$ or infan$ or newborn$ or "new born")

**MEDLINE and Embase (OVID)**

Date searched: 30/05/2016; with top-up search conducted on 10/08/2018 and 03/09/2019

Total records retrieved: **4044**

1. exp Magnesium Sulfate/
2. ((magnesium adj sulfate) or (magnesium adj sulphate) or MgSO4).mp.
3. or/1-2
4. exp Pregnancy/
5. exp Pregnancy Complications/
6. exp Prenatal Care/
7. exp Perinatal Care/
8. exp Maternal Exposure/
9. (pregnan$ or labor or laboring or labour$ or antepart$ or prenatal$ or antenatal$ or perinatal$ or intranatal$ or obstetric$ or intrapart$ or preterm or prematur$ or tocoly$ or (maintenance adj therapy) or preeclamp$ or pre-eclamp$ or (pre adj eclamp$) or eclamp$ or neuroprotection$ or (cerebral adj palsy)).mp.
10. or/4-9
11. exp Infant/
12. exp Infant, Newborn, Diseases/
13. Intensive Care, Neonatal/
14. (fetus$ or fetal$ or foetus$ or foetal$ or baby or babies or neonat$ or infan$ or newborn$ or (new adj born)).mp.
15. or/11-14
16. 3 and 10 and 15
17. exp Animals/
18. exp Humans/
19. 17 not 18
20. 16 not 19
21. Remove duplicates from 20

**TOXLINE**

Date searched: 30/05/2016; with top-up search conducted on 10/08/2018 and 03/09/2019

Total records retrieved: **319**

(“magnesium sulphate” OR “magnesium sulphate” OR MgSO4) AND (fetus* or fetal* or foetus* or foetal* or baby or babies or neonat* or infan* or newborn* or “new born”)

**Web of Science**

Date searched: 30/05/2016; with top-up search conducted on 10/08/2018 and 03/09/2019

Total records retrieved: **553**

1. TS=(“magnesium sulphate” OR “magnesium sulphate” OR MgSO4) *Timespan=All years*
2. TS=(fetus* or fetal* or foetus* or foetal* or baby or babies or neonat* or infan* or newborn* or “new born”) *Timespan=All years*
3. TS=(pregnan* OR labor OR laboring OR labour* OR antepart* OR prenatal* OR antenatal* OR perinatal* OR intranatal* OR obstetric* OR intrapart* OR preterm OR prematur* OR tocoly* OR “maintenance therapy” OR preeclamp* OR pre-eclamp* OR “pre eclamp*” OR eclamp* OR neuroprotection* OR “cerebral palsy*”) *Timespan=All years*
4. #3 AND #2 AND #1 *Timespan=All years*
5. #3 AND #2 AND #1 Refined by: Databases: ( BCI OR SCIELO OR WOS OR BIOSIS OR KJD OR CCC OR CABI OR RSCI )
