# Supplementary material for: Antenatal magnesium sulphate and adverse neonatal outcomes: A systematic review and meta-analysis
Source: PLoS Med. 2019 Dec 6;16(12):e1002988. doi: 10.1371/journal.pmed.1002988 (PMC6897495; doi:10.1371/journal.pmed.1002988)
Supplement: S3 Text — (DOCX) [file pmed.1002988.s010.docx]

**Records excluded at full-text screening due to absence of English translation**

1. Abalos E, Giordano D, Majic C, Morales EM, Peretti JI, Ramos S. Morbilidad severa materna y neonatal: vigilancia en servicios y capacidad de respuesta del sistema de salud. Rev Argent Salud Pública 2014; 5(18): 15-23.
2. Baraibar R, Krauel J, Molina V. Efectos de la administracion de sulfato de magnesio a la mujer embarazada sobre el feto y el neonate. Prog Obstet Ginecol. 1978; 21(4): 209-12.
3. Beliaev IT, Ishpakhtin I. [Effect of certain drug substances in late pregnancy toxicoses on the fetal cardiac activity]. Vopr Okhr Materin Det. 1973; 18(3): 71-4.
4. Bourret B, Compere V, Torre S, Azhougagh K, Provost D, Rachet B, Gillet R, et al. Évaluation de l’utilisation du sulfate de magnésium dans la prévention secondaire de l’éclampsie : étude rétrospective sur 39 cas. Ann Fr Anesth. 2012; 31(12): 933-6.
5. Bruhwiler H, Hafligher M, Luscher KP. Schwere akzidentelle Magnesiumintoxikation bei einer Zwillingsschwangerschaft in der 32. SSW. Geburtshilfe Frauenheilkd. 1994; 54(3): 184-6.
6. Civi S, Marakoglu K, Sahsivar S. Aile hekimliginde iki olgu sunumu ile preeklampsi ve eklampsinin incelenmesi. Turkiye Klinikleri J Med Sci. 2008; 28(3): 382-6.
7. Feitosa HN, Alencar Junior CA, Camano L, Bertini AM; Santos JFK. Repercussäo do sulfato de magnésio na frequência cardíaca fetal. Femina 1990; 18(4): 272-3.
8. Figueroa Calderón I, Saavedra Moredo D; de la Torres Sieres Y; Sánchez Lueiro M. Eficacia del sulfato de magnesio en el tratamiento de la preeclampsia. Rev Cuba Obstet Ginecol. 2012; 38(4): 458-66.
9. Freire S. Tratamento da eclâmpsia com o sulfato de magnésio, em um grupo de primigrávidas. J Bras Ginecol. 1986; 96(7): 323-33.
10. Freire S. Repercussöes do tratamento da pré-eclâmpsia com sulfato de magnésio com início no pré-parto ou no pós-parto. Thesis 1997. [<http://bases.bireme.br/cgi-bin/wxislind.exe/iah/online/?IsisScript=iah/iah.xis&src=google&base=LILACS&lang=p&nextAction=lnk&exprSearch=236732&indexSearch=ID>]
11. Ganzevoort JW, Hoogerwaard EM, Van Der Post JAM. Hypocalciemisch delier door magnesiumsulfaatbehandeling bij een zwangere met preeclampsie. Ned Tijdschr Geneeskd. 2002; 146(31): 1453-6.
12. Hiltmann WD, Wischnik A, Hettenbach A, Melchert F. Ie auswirkungen einer bolusgabe von mgso4 auf das fetale herz-kreislauf-system (dopsonographische untersuchungen). Arch Gynecol Obstet. 1989; 245(1-4): 101.
13. Kiriushchenkov AP, MetaksaIa V. [The effect of magnesium sulfate on fetal heart action]. Vopr Okhr Materin Det 1966; 11(1): 72-7.
14. Kyank H. [Magnesium-sulfate treatment of severe preeclampsia eclampsia]. Zentralblatt fur Gynakologie. 1990; 112(1): 5-10.
15. Lokossou A, Avode DG, Komongui DG, Takpara I, Sacca PC, Perrin RX. Prise en charge des manifestations neurologiques de la pre- eclampsie severe et de l'eclampsie par le sulfate de magnesium a Cotonou. Afr J Neuroll Sci. 2006; 25(1): 41-9.
16. Malek-Mellouli M, Atef Y, Ben Amara F, Nasr M, Khaled N, Bouchnack M, et al. [Sulfate de magnésium au cours de la préeclampsie sévère : innocuité d’utilisation?](http://www.latunisiemedicale.com/article-medicale-tunisie_2006_fr). Tunis Med. 2012; 90(7): 552-6.
17. Martinez Orgado J, Saez Perez E, Garcia Aparicio J. Efectos sobre el neonato del tratamiento antihipertensivo materno. Rev Esp Pediatr 1991; 47(280): 296-300.
18. Millochau JC, Marret S, Oden S, Verspyck E. État des lieux de l’utilisation du sulfate de magnésium à visée neuroprotectrice au CHU de Rouen. Gynecol Obstet Fertil. 2016; 44(7-8): 446-9.
19. Nitsche A, Kliemann R, Manfrim EB, Zeigelboin BS, Liberalesso P. Hemorragia cerebral em recém-nascidos de baixo peso e o uso de sulfato de magnésio pré-natal. Pediatr Mod. 2014; 50(2).
20. Presl J. [Effect of MgSO-4 on fetus, newborn infant and uterine activity]. Cesk Gynekol 1972; 37(2): 112-3.
21. Souza AS, Amorim MM, Coelho IC, Lima MM, Noronha NC, Figueroa JN. Doppler das artérias umbilicais e cerebral média fetal após sulfato de magnésio na pré-eclâmpsia. Rev Assoc Med Bras 2008; 54(3): 232-7.
22. Spatling L. [Magnesium medication in addition to tocolysis - chemical monitoring]. Geburtshilfe Frauenheilkd. 1984; 44(1): 19-24
23. Spuls PI, Offringa M. Magnesiumsulfaat bij dreigende vroeggeboorte: Minder risico op spasticiteit bij baby's. Ned Tijdsch Geneeskd 2009; 153(29): 1449.
24. Tejada R, Roig A, Tejada D, Halls A, Rodríguez V, Bencosme S. Disminución de respuesta relajante de la vena umbilical de recien nacidos de pacientes preeclampticas al sulfato de magnesio. Acta Méd Domin. 1990; 12(6): 226-30.
25. Unknown. ¿Se benefician las mujeres con pre-eclampsia y sus niños con el sulfato de magnesio? El estudio Magpie: una investigación clínica aleatorizada. Rev Hosp Matern Infant Ramon Sarda 2002; 21(4): 147-54.
26. Unknown. Sulfato de magnesio para la neuroprotección fetal. Prog Obstet Ginecol. 2012; 55(8): 416-21.
