# Supplementary material for: Antenatal magnesium sulphate and adverse neonatal outcomes: A systematic review and meta-analysis
Source: PLoS Med. 2019 Dec 6;16(12):e1002988. doi: 10.1371/journal.pmed.1002988 (PMC6897495; doi:10.1371/journal.pmed.1002988)
Supplement: S4 Text — (DOCX) [file pmed.1002988.s011.docx]

**References to included studies**

**Randomised controlled trials**

1. Abdul M, Nasir U, Khan N, Yusuf M. Low-dose magnesium sulphate in the control of eclamptic fits: a randomized controlled trial. Arch Gynecol Obstet. 2013;287(1):43-6.
2. Agrawal S, Das V, Verma V, Agarwal A, Pandey A, Jain V. Evaluation of medium dose versus standard Pritchard regime of magnesium sulfate in the management of eclampsia in developing nation. Int J Gynaecol Obstet. 2015;131(Suppl 5):E183.
3. Bain E, Middleton P, Yelland L, Ashwood P, Crowther C. Maternal adverse effects with different loading infusion rates of antenatal magnesium sulphate for preterm fetal neuroprotection: the IRIS randomised trial. Br J Obstet Gynaecol. 2014;121(5):595-603.
4. Begum M, Begum A, Quardir E. Loading dose versus standard regime of magnesium sulfate in the management of eclampsia: A randomized trial. J Obstet Gynaecol Res. 2002;28(3):154-9.
5. Behrad B, Moossavifar N, Motahedzadeh M, Esmaili H, Moghtadeii P. A prospective, randomized, controlled trial of high and low doses of magnesium sulfate for acute tocolysis. Acta Med Iran. 2003;41(2):126-31.
6. Bhattacharjee N, Saha S, Ganguly R, Patra K, Shali B, Das N, et al. A randomised comparative study between low-dose intravenous magnesium sulphate and standard intramuscular regimen for the treatment of eclampsia. J Obstet Gynaecol. 2011;31(4):298-303.
7. Blackwell S, Hallak M, Hassan S, Berry S, Russell E, Sorokin Y. The effects of intrapartum magnesium sulfate therapy on fetal serum interleukin-1β, interleukin-6, and tumor necrosis factor-α at delivery: a randomized, placebo-controlled trial. Am J Obstet Gynecol. 2001;184(7):1320-4.
8. Chama C, Geidam A, Bako B, Mairiga A, Atterwahmie A. A shortened versus standard matched postpartum magnesium sulphate regimen in the treatment of eclampsia: a randomised controlled trial. Afr J Reprod Health. 2013; 17(3):131-6.
9. Chen F-P, Chang S-D, Chu K-K. Expectant management in severe preeclampsia: does magnesium sulfate prevent the development of eclampsia? Acta Obstet Gynecol Scand. 1995;74(3):182-5.
10. Chissell S, Botha J, Moodley J, McFadyen L. Intravenous and intramuscular magnesium sulphate regimens in severe pre-eclampsia. S Afr Med J. 1994;84(9):607-10.
11. Coetzee E, Dommisse J, Anthony J. A randomised controlled trial of intravenous magnesium sulphate versus placebo in the management of women with severe pre-eclampsia. Br J Obstet Gynaecol. 1998;105(3):300-3.
12. Colon I, Berletti M, Garabedian M, Wilcox N, Williams K, Chueh J, et al. Randomized, double-blinded trial of magnesium sulfate tocolysis vs intravenous normal saline for nonsevere placental abruption. Am J Obstet Gynecol. 2015;212(1 Suppl):S388-9.
13. Cotton D, Strassner H, Hill L, Schifrin B, Paul R. Comparison of magnesium sulfate, terbutaline and a placebo for inhibition of preterm labor. A randomized study. J Reprod Med. 1984;29(2):92-7.
14. Cox S, Sherman L, Leveno K. Randomized investigation of magnesium sulfate for prevention of preterm birth. Am J Obstet Gynecol. 1990;163(3):767-72.
15. Crowther C, Hiller J, Doyle L, Haslam R, for the Australasian Collaborative Trial of Magnesium Sulphate (ACTOMgSO4) Collaborative Group. Effect of magnesium sulfate given for neuroprotection before preterm birth. A randomized controlled trial. JAMA. 2003;290(20):2669-76.
    - Paradisis M OD, Evans N, Kluckow M. Randomized controlled trial of magnesium sulfate in women at risk of preterm delivery – neonatal cardiovascular effects. J Perinatol. 2012;32(9):665-70.*
16. Easterling T, Hebert M, Bracken H, Darwish E, Ramadan MC, Shaarawy S, et al. A randomized trial comparing the pharmacology of magnesium sulfate when used to treat severe preeclampsia with serial intravenous boluses versus a continuous intravenous infusion. BMC Pregnancy Childbirth. 2018;18(1):290.
17. Fox M, Allbert J, McCaul J, Martin R, McLaughlin B, Morrison J. Neonatal morbidity between 34 and 37 weeks’ gestation. Obstet Gynecol Surv. 1993;49(4):242-3.
    - Fox M, Allbert J, McCaul J, Martin R, McLaughlin B, Morrison J. Neonatal morbidity between 34 and 37 weeks’ gestation. J Perinatol. 1993;13(5):349-53.*
18. Magpie Trial Collaborative Group. Do women with pre-eclampsia, and their babies, benefit from magnesium sulphate? The Magpie Trial: a randomised placebo-controlled trial. Lancet. 2002;359(1):1877-90.
19. How HY CC, Cook VD, Miles DE, Spinnato JA. Preterm premature rupture of membranes: aggressive tocolysis versus expectant management. J Matern Fetal Med. 1998;7(1):8-12.
20. Keepanasseril A, Maurya DK, Manikandan K, Suriya YJ, Habeebullah S, Raghavan SS. Prophylactic magnesium sulphate in prevention of eclampsia in women with severe preeclampsia: randomised controlled trial (PIPES trial). J Obstet Gynaecol. 2018;38(3):305-9.
21. Lewis DF, Bergstedt S, Edwards MS, Burlison S, Gallaspy JW, Brooks GG, Adair CD. Successful magnesium sulfate tocolysis: is “weaning” the drug necessary? Am J Obstet Gynecol. 1997;177(4):742-5.
22. Livingston J, Livingston L, Ramsey R, Mabie B, Sibai B. Magnesium sulfate in women with mild preeclampsia: a randomized controlled trial. Obstet Gynecol. 2003;101(2):217-20.
23. Malapaka S, Ballal P. Low-dose magnesium sulfate versus Pritchard regimen for the treatment of eclampsia and imminent eclampsia. Int J Gynaecol Obstet. 2011;115(1):70-2.
24. Marret S, Marpeau L, Zupan-Simunek V, Eurin D, Leveque C, Hellot M-F, et al. Magnesium sulphate given before very-preterm birth to protect infant brain: the randomised controlled PREMAG trial. Br J Obstet Gynaecol. 2007;114(3):310-8.
25. Mirzamoradi M, Behnam M, Jahed T, Saleh-Gargari S. Does magnesium sulfate delay the active phase of labor in women with premature rupture of membranes? A randomized controlled trial. Taiwan J Obstet Gynecol. 2014;53(3):309-12.
26. Mittendorf R, Dambrosia J, Pryde P, Lee K-S, Gianopoulois J, Besinger R, et al. Association between the use of antenatal magnesium sulfate in preterm labor and adverse health outcomes in infants. Am J Obstet Gynecol. 2002;186(6):1111-8.
27. Moodley J, Moodley V. Prophylactic anticonvulsant therapy in hypertensive crises of pregnancy – the need for a large, randomized trial. Hypertens Pregnancy. 1994;13(3):245-52.
28. Mundle S, Regi A, Easterling T, Biswas B, Bracken H, Khedekar V, et al. Treatement approaches for preeclampsia in low-resource settings: a randomized trial of the Spingfusor pump for delivery of magnesium sulfate. Pregnancy Hypertens. 2012;2(1):32-8.
29. Orji E, Ogoke G, Fasubaa O. Efficacy of a single loading dose of magnesium sulphate versus the standard Pritchard regimen in the management of severe preeclampsia in an African population. Int J Gynaecol Obstet. 2012;119(S3):S447.
30. Parashi S, Bordbar A, Mahmoodi Y, Jafari M. The survey of magnesium sulfate in prevention of intraventricular haemorrhage in premature infants: a randomized clinical trial. Shiraz E Med J. 2017;18(11):e55094.
31. Pascoal ACF, Katz L, Pinto MH, Santos CA, Braga LCO, Maia SB, et al. Serum magnesium levels during magnesium sulfate infusion at 1 gram/hour versus 2 grams/hour as a maintenance dose to prevent eclampsia in women with severe preeclampsia: A randomized clinical trial. Medicine (Baltimore). 2019;98(32):e16779.
32. Rimal S, Rijal P, Bhatt R, Thapa K. Loading dose only versus standard dose magnesium sulfate seizure prophylaxis in severe pre-eclamptic women. J Nepal Med Assoc. 2017;56(208):388-94.
33. Rouse D, Hirtz D, Thom E, Varner M, Spong C, Mercer B, et al. A randomized, controlled trial of magnesium sulfate for the prevention of cerebral palsy. N Engl J Med. 2008;359(9):895-905.
    - Hirtz DG WS, Bulas D, DiPietro M, Seibert J, Rouse DJ, Mercer BM, Varner MW, Reddy UM, Iams JD, Wapner RJ, Sorokin Y, Thorp JM, Tamin SM, Malone FD, Carpenter MW, O’Sullivan MJ, Peaceman AM, Hankins GDV, Dudley D, Caritis SN, on behalf on the Eunice Kennedy Shriver National Institute of Child Health and Human Development Maternal-Fetal Medicine Units Network. Antenatal magnesium and cerebral palsy in preterm infants. J Pediatr. 2015;167(4):834-9.*
    - Horton AL LY, Rouse DJ, Spong CY, Keveno KJ, Varner MW, Mercer BM, Iams JD, Wapner RJ, Sorokin Y, Thorp JM, Ramin SM, Malone FD, O’Sullivan MJ, Hankins GDV, Caritis SN, the Eunice Kennedy Shriver National Institute of Child Health and Human Development Maternal-Fetal Medicine Units Network. Effect of magnesium sulfate administration for neuroprotection on latency in women with preterm premature rupture of membranes. Am J Perinatol. 2015;32(4):387-92.*
    - Vilchez G, Dai J, Kumar K, Mundy D, Kontopoulos E, Sokol RJ. Racial/ethnic disparities in magnesium sulfate neuroprotection: a subgroup analysis of a multicenter randomized controlled trial. J Matern Fetal Neonatal Med. 2018;31(17):2304-11.*
    - Vilchez G, Dai J, Lagos M, Sokol RJ. Maternal side effects and fetal neuroprotection according to body mass index after magnesium sulfate in a multicenter randomized controlled trial. J Matern Fetal Neonatal Med. 2018b;31(2):178-83.*
34. Saha P, Kaur J, Goel P, Kataria S, Tandon R, Saha L. Safetly and efficacy of low dose intramuscular magnesium sulphate (MgSO4) compared to intravenous regimen for treatment of eclampsia J Obstet Gynaecol Res. 2017;4(10):1543-9.
35. Shilva., Saha S, Kalra J, Prasad R. Safety and efficacy of low-dose MgSO4 in the treatment of eclampsia. Int J Gynaecol Obstet. 2007;97(2):150-1.
36. Shreya M, Krishna L, Shailaja N, Bhat B. Evaluation of single dose magnesium sulphate and Pritchard regimen in the treatment of eclampsia – A comparative study. Biomedicine. 2014;34(2):252-6.
37. Singh S, Behera A. Eclampsia in Eastern India: incidence, demographic profile and response to three different anticonvulsant regimes of magnesium sulphate. Internet J Gynecol Obstet. 2011;15(2):1-7.
38. Tangmanowutthikul S, Champawong R, Songthamwat S, Songthamwat M. Comparison of magnesium sulphate protocols by weight-adjusted versus two grams per hour for preventing convulsion in preeclampsia: a randomised controlled trial. J Clin Diagn Res. 2019;13(2):QC01-4.
39. Terrone D, Rinehart B, Kimmel E, May W, Larmon J, Morrison J. A prospective, randomized, controlled trial of high and low maintenance doses of magnesium sulfate for acute tocolysis. Am J Obstet Gynecol. 2000;182(6):1477-82.
40. Wiltlin A, Friedman S, Sibai B. The effect of magnesium sulfate therapy on the duration of labor in women with mild preeclampsia at term: A randomized, double-blind, placebo-controlled trial. Am J Obstet Gynecol. 1997;176(3):623-7.

**Non-randomised studies**

1. Adama-Hondegla AB, Lawson-Evi K, Bassowa A, Modji S, Egbla KF, Akpadza K. Perinatal mortality risk factors of infants bom from eclamptic mothers at Tokoin Teaching Hospital of Lome. Pak J Med Sci. 2013;13(5):391-5.
2. Alexander JM, McIntire DD, Leveno KJ, Cunningham FG. Selective magnesium sulfate prophylaxis for the prevention of eclampsia in women with gestational hypertension. Obstet Gynecol. 2006;108(4):826-32.
3. Alston MJ, Alexandrovic K, Stiglich N, Metz TD. Discontinuation of tocolytics for preterm labor in an academic safety net hospital: Impact on the duration of betamethasone exposure. J Reprod Med. 2016;61(2):109-13.
4. Ambadkar A, Prasad M, Chauhan AR. Neonatal effects of maternal magnesium sulphate in late preterm and term pregnancies. J Obstet Gynaecol India. 2019;69(1):25-30.
5. Bajaj M, Natarajan G, Shankaran S, Wyckoff M, Laptook AR, Bell EF, et al. Delivery room resuscitation and short-term outcomes in moderately preterm infants. J Pediatr. 2018;195:33-8e2.
6. Basu SK, Chickajajur V, Lopez V, Bhutada A, Pagala M, Rastogi S. Immediate clinical outcomes in preterm neonates receiving antenatal magnesium for neuroprotection. J Perinat Med. 2012;40(2):185-9.
7. Belden MK, Gnadt S, Ebert A. Effects of maternal magnesium sulfate treatment on neonatal feeding tolerance. J Pediatri Pharmacol Ther. 2017;22(2):112-7.
8. Bertello Grecco M, Barrón B, Rigo D, McCormick Cook A, Pajón Scocco J, Novoa P, et al. Maternal and neonatal safety with the use of magnesium sulfate in preeclampsia. Kidney Int Rep. 2019;4(7):S146.
9. Black B, Holditch-Davis D, Schwartz T, Scher MS. Effects of antenatal magnesium sulfate and corticosteroid therapy on sleep states of preterm infants. Res Nurs Health. 2006;29(4):269-80.
10. Blackwell SC, Redman ME, Whitty JE, Refuerzo JS, Berry SM, Sorokin Y, et al. The effect of intrapartum magnesium sulfate therapy on fetal cardiac troponin I levels at delivery. J Matern Fetal Neonatal Med. 2002;12(5):327-31.
11. Bonta BW, Chin TK, DeVoe WM. Maternal intravenous MgSO4 administration and its effects on neonatal respiratory function and risk of development of hemodynamically significant patent ductus arteriosus shunts during the initial 72 hours of life. J Investig Med. 2000;48(1):107A.
12. Bozkurt O, Eras Z, Canpolat FE, Oguz SS, Uras N, Dilmen U. Antenatal magnesium sulfate and neurodevelopmental outcome of preterm infants born to preeclamptic mothers. J Matern Fetal Neonatal Med. 2016;29(7):1101-4.
13. Boyle A, Greer K, Caballero A, Norton T, Kate P, Ferguson J, et al. Neonatal outcomes in obese women undergoing cesarean delivery for fetal heart rate tracing abnormalities. Am J Obstet Gynecol. 2018;218(1):S335.
14. Brazy JE, Grimm JK, Little VA. Neonatal manifestations of severe maternal hypertension occurring before the thirty-sixth week of pregnancy. J Pediatr. 1982;100(2):265-71.
15. Brookfield K, Su F, Drover D, Adelus M, Lyell D, Carvalho B. Umbilical cord magnesium levels and neonatal resuscitation in infants exposed to magnesium sulfate. Am J Obstet Gynecol. 2015;212(1 Suppl):S395-6.
16. Brookfield K, O’Malley K, Yeaton-Massey A, Butwick A. Does magnesium sulfate exposure attenuate the effete of steroids administered for fetal lung maturation? Am J Obstet Gynecol. 2016;1(Suppl):S89.
17. Brown BE, Vincer M, Acott P, El-Naggar W, O’Connell C, Kajetanowicz A. Systemic hypertension in preterm infants - a population-based study. Paediatr Child Health. 2019;24(Suppl 2):e47-8.
18. Canterino JC, Verma UL, Visintainer PF, Figueroa R, Klein SA, Tejani NA. Maternal magnesium sulfate and the development of neonatal periventricular leucomalacia and intraventricular hemorrhage. Obstet Gynecol. 1999;93(3):396-402.
19. Cawyer CR. The association of magnesium sulfate with maternal morbidity when used for preeclampsia without severe features. Am J Obstet Gynecol. 2019;220(1):S292-3.
20. Cho GJ, Lee JE, Hong HR, Hong SC, Hong YS, Kim HJ, et al. Maternal magnesium sulfate treatment is not associated with serum calcium levels of preterm neonate. Am J Obstet Gynecol. 2014;210(1 Suppl):S356.
21. Chowdhury JR, Chaudhuri S, Bhattacharyya N, Biswas PK, Panpalia M. Comparison of intramuscular magnesium sulfate with low dose intravenous magnesium sulfate regimen for treatment of eclampsia. J Obstet Gynaecol Res. 2009;35(1):119-25.
22. Chun E-H, Do S-H, Shin H-J, Na H-S, Hwang J-W. Effects of magnesium sulfate on the labor duration and neonatal outcome in parturients with preeclampsia. Anesth Pain Med. 2014;9(2):128-33.
23. Cuff RD, Sullivan SA, Chang EY. Impact of dosing schedule on uptake of neuroprotective magnesium sulfate. J Matern Fetal Neonatal Med. 2018 Sep 19 doi: 10.1080/14767058.2018.1513482.
24. Das M, Chaudhuri PR, Mondal BC, Mitra S, Bandyopadhyay D, Pramanik S. Assessment of serum magnesium levels and its outcome in neonates of eclamptic mothers treated with low-dose magnesium sulfate regimen. Indian J Pharmacol. 2015;47(5):502-8.
25. De Jesus L, Sood B, Shankaran S, Kendrick D, Das A, Bell E, et al. Antenatal magnesium sulfate exposure and acute cardiorespiratory events in preterm infants. Am J Obstet Gynecol. 2015;212(1):94.e1-7.
26. De Silva D, Synnes A, von Dadelszen P, Lee T, Bone J, Mag-CP., et al. MAGnesium sulphate for fetal neuroprotection to prevent Cerebral Palsy (MAG-CP)-implementation of a national guideline in Canada. Implement Sci. 2018;13(1):8.
27. de Veciana M, Porto M, Major CA, Barke JI. Tocolysis in advanced preterm labor: impact on neonatal outcome. Am J Perinatol. 1995;12(4):294-8.
28. Deering SH, Stagg AR, Spong CY, Abubakar K, Pezzullo JC, Ghidini A. Antenatal magnesium treatment and neonatal illness severity as measured by the Score for Neonatal Acute Physiology (SNAP). J Matern Fetal Neonatal Med. 2005;17(2):151-5.
29. del moral T, Gonzalez-Quintero VH, Claure N, Vanbuskirk S, Bancalari E. Antenatal exposure to magnesium sulfate and the incidence of patent ductus arteriosus in extremely low birth weight infants. J Perinatol. 2007;27(3):154-7.
30. delValle GM, Bister GL, Lynch LA, Cummings JJ. Prenatal magnesium sulfate exposure and the incidence of cerebral palsy in very low birth weight infants. J Investig Med. 1998;46(1):175A.
31. Derks JB, Sol CM, Van Leeuwen J, Keunen K, Mulder EJ, De Vries LS, et al. Antenatal magnesiumsulphate for neuroprotection reduces punctate white matter laesions at 30 weeks MRI in the human neonate. Reprod Sci. 2016;23(Suppl 1):273A.
32. Downey LC, Cotten CM, Hornik CP, Laughon MM, Tolia VN, Clark RH, et al. Association of in utero magnesium exposure and spontaneous intestinal perforations in extremely low birth weight infants. J Perinatol. 2017;37(6):641-4.
33. Drassinower D, Obican S, Levin H, Gyamfi-Bannerman C. Immediate neonatal outcomes in infants exposed to magnesium sulfate at the time of delivery. Am J Obstet Gynecol. 2015;212(1 Suppl):S90.
34. Duffy CR, Odibo AO, Roehl KA, Macones GA, Cahill AG. Effect of magnesium sulfate on fetal heart rate patterns in the second stage of labor. Obstet Gynecol. 2012;119(6):1129-36.
35. Edwards J, Edwards L, Swamy G, Grotegut C. Magnesium sulfate for neuroprotection in the setting of chorioamnionitis. J Matern Fetal Neonatal Med. 2018;31(9):1156–60.
36. Elimian A, Verma R, Ogburn P, Wiencek V, Spitzer A, Quirk JG. Magnesium sulfate and neonatal outcomes of preterm neonates. J Matern Fetal Neonatal Med. 2002;12(2):118-22.
37. Elliott J, Garite T, Clark R, Combs A. Perinatal effect of magnesium sulfate administered for tocolysis. Am J Obstet Gynecol. 2003;189(6 Suppl):S63.
38. Farkouh LJ, Thorp JA, Jones PG, Clark RH, Knox GE. Antenatal magnesium exposure and neonatal demise. Am J Obstet Gynecol. 2001;185(4):869-72.
39. FineSmith RB, Roche K, Yellin PB, Walsh KK, Shen C, Zeglis M, et al. Effect of magnesium sulfate on the development of cystic periventricular leukomalacia in preterm infants. Am J Perinatol. 1997;14(5):303-7.
40. Gano D, Ho ML, Partridge JC, Glass HC, Xu D, Barkovich AJ, et al. Antenatal exposure to magnesium sulfate is associated with reduced cerebellar hemorrhage in preterm newborns. J Pediatr. 2016;178:68-74.
41. Garcia Alonso L, Pumarada Priet M, Gonzalez Colmenero E, Concheiro Guisan A, Suarez Albo M, Duran Fernandez-Feijoo C, et al. Prenatal therapy with magnesium sulfate and its correlation with neonatal serum magnesium concentration. Am J Perinatol. 2018;35(2):170-6.
42. Gasparyan A. [Neurosonographical characteristics of dysmature infants depending on conducted neuroprotection]. Georgian Med News. 2017;(268-9):72-5.
43. Ghidini A, Espada RA, Spong CY. Does exposure to magnesium sulfate in utero decrease the risk of necrotizing enterocolitis in premature infants? Acta Obstet Gynecol Scand. 2001;80(2):126-9.
44. Gibbins KJ, Browning KR, Lopes VV, Anderson BL, Rouse DJ. Evaluation of the clinical use of magnesium sulfate for cerebral palsy prevention. Obstet Gynecol. 2013;121(2 Pt 1):235-40.
45. Girsen AI, Greenberg MB, El-Sayed YY, Lee H, Carvalho B, Lyell DJ. Magnesium sulfate exposure and neonatal intensive care unit admission at term. J Perinatol. 2015;35(3):181-5.
46. Gonzalez-Quintero VH, Tolaymat L, Claure N, Vanbuskirk S, Siman D, del Moral T, et al. Survival rate in neonates exposed to magnesium sulfate. J Perinat Med. 2001;29(Suppl 1):20.
47. Greenberg MB, Penn AA, Thomas LJ, El-Sayed YY, Caughey AB, Lyell DJ. Neonatal medical admission in a term and late-preterm cohort exposed to magnesium sulfate. Am J Obstet Gynecol. 2011;204(6):515.e1-7.
48. Greenberg MB, Penn AA, Whitaker KR, Kogut EA, El-Sayed YY, Caughey AB, et al. Effect of magnesium sulfate exposure on term neonates. J Perinatol. 2013;33(3):188-93.
49. Grether JK, Hoogstrate J, Selvin S, Nelson KB. Magnesium sulfate tocolysis and risk of neonatal death. Am J Obstet Gynecol. 1998;178(1 Pt 1):1-6.
50. Grimbly C, Rosolowsky E, Aziz K, O'Reilly M, Cheung PY, Schmolzer G. New baby jitters: Novel characterization of the incidence and risk factors for neonatal hypoglycemia in the premature infant <33 weeks. Paediatr Child Health. 2015;20(5):e86.
51. Gulcan H, Gungor S, Tiker F, Kilicdag H. Effect of perinatal factors on time of first stool passage in preterm newborns: An open, prospective study. Curr Ther Res Clin Exp. 2006;67(3):214-25.
52. Gursoy T, Imamoglu EY, Ovali F, Karatekin G. Effects of antenatal magnesium exposure on intestinal blood flow and outcome in preterm neonates. Am J Perinatol. 2015;32(11):1064-9.
53. Havranek T, Ashmeade TL, Afanador M, Carver JD. Effects of maternal magnesium sulfate administration on intestinal blood flow velocity in preterm neonates. Neonatology. 2011;100(1):44-9.
54. Hechtman J, Blackwell S, Moldenhauer J, Refuerzo J, Hassan S, Berry S, et al. Lack of association of neonatal mortality and exposure to tocolytic magnesium. Am J Obstet Gynecol. 2002;187(6 Suppl 1):S124.
55. Holcomb WL, Shackelford GD, Petrie RH. Magnesium tocolysis and neonatal bone abnormalities: a controlled study. Obstet Gynecol. 1991;78(4):611-4.
56. Hom K, Brar B, Kennel P, Jackson D. Magnesium for fetal neuroprotection: Should it be started when delivery is not imminent in pprom? Obstet Gynecol. 2018;131 (Suppl 1):44S.
57. Hong JY, Kim Y-M, Hong JY, Seo M-r, Chae J, Sung J-H, et al. Does antenatal magnesium sulfate exposure increase the risk of necrotizing enterocolitis in preterm neonates? Am J Obstet Gynecol. 2019;220(1):S327.
58. Igarashi H, Honma Y, Suwa K, Momoi M, Yanagisawa M. The clinical effects of hypermagnesemia on preterm infants of mothers treated with magnesium sulfate for tocolysis. Acta Neonatol Japon. 1995;31(2):388-93.
59. Imamoglu EY, Gursoy T, Karatekin G, Ovali F. Effects of antenatal magnesium sulfate treatment on cerebral blood flow velocities in preterm neonates. J Perinatol. 2014;34(3):192-6.
60. James AT, Corcoran JD, Hayes B, Franklin O, El-Khuffash A. The effect of antenatal magnesium sulfate on left ventricular afterload and myocardial function measured using deformation and rotational mechanics imaging. J Perinatol. 2015;35(11):913-8.
61. Jazayeri A, Jazayeri MK, Sutkin G. Tocolysis does not improve neonatal outcome in patients with preterm rupture of membranes. Am J Perinatol. 2003;20(4):189-93.
62. Jeanneteau P, Bouet PE, Baisson AL, Courtay V, Gascoin-Lachambre G, Gillard P, et al. Evaluation of the clinical use of magnesium sulfate for cerebral palsy prevention. J Matern Fetal Neonatal Med. 2014;27(Suppl 1):377-8.
63. Jones CW, Petrashek K, Wenzlaff M, Simpson P, Pan AY. Prenatal magnesium sulfate and time to first stool in late preterm infants. Obstet Gynecol. 2018;131(Suppl 1):160S.
64. Jung EJ, Byun JM, Kim YN, Lee KB, Sung MS, Kim KT, et al. Antenatal magnesium sulfate for both tocolysis and fetal neuroprotection in premature rupture of the membranes before 32 weeks' gestation. J Matern Fetal Neonatal Med. 2018;31(11):1431-41.
65. Kamilya G, Bharracharyya SK, Mukherji J. Changing trends in the management of eclampsia from a teaching hospital. J Indian Med Assoc. 2005;103(3):132, 4-5.
66. Kamyar M, Bardsley T, Korgenski K, Clark E. Magnesium sulfate and the extremely low birth weight neonate. Am J Obstet Gynecol. 2015;212(1 Suppl):S362-3.
67. Kamyar M, Bardsley T, Korgenski K, Clark EAS. Association of antenatal magnesium sulfate with neonatal morbidity and mortality in very preterm infants. Reprod Sci. 2015;22(Suppl 1):144A.
68. Kamyar M, Clark EA, Yoder BA, Varner MW, Manuck TA. Antenatal magnesium sulfate, necrotizing enterocolitis, and death among neonates<28 weeks gestation. AJP Rep. 2016;6(1):e148-54.
69. Kamyar M, Manuck TA, Stoddard GJ, Varner MW, Clark EAS. Magnesium sulfate, chorioamnionitis, and neurodevelopment after preterm birth. Br J Obstet Gynaecol. 2016;123(7):1161-6.
70. Kamyar M, Varner M, Clark E. Magnesium sulfate neuroprophylaxis and the effect of infant sex. Am J Obstet Gynecol. 2015;212(1 Suppl):S144.
71. Katayama Y, Minami H, Enomoto M, Takano T, Hayashi S, Lee YK. Antenatal magnesium sulfate and the postnatal response of the ductus arteriosus to indomethacin in extremely preterm neonates. J Perinatol. 2011;31(1):21-4.
72. Kelly MJ, Viscardi RM. Effects of maternal magnesium sulfate on preterm newborns. Pediatr Res. 1992;31(4 Pt 2):207A.124.
73. Khodapanahandeh F, Khosravi N, Larijani T. Risk factors for intraventricular hemorrhage in very low birth weight infants in Tehran, Iran. Turk J Pediatr. 2008;50(3):247-52.
74. Kimberlin DF, Hauth JC, Goldenberg RL, Bottoms SF, Iams JD, Mercer B, et al. The effect of maternal magnesium sulfate treatment on neonatal morbidity in < or = 1000-gram infants. Am J Perinatol. 1998;15(11):635-41.
75. Koksal N, Baytan B, Bayram Y, Nacarkucuk E. Risk factors for intraventricular haemorrhage in very low birth weight infants. Indian J Pediatr. 2002;69(7):561-4.
76. Kuban KC, Leviton A, Pagano M, Fenton T, Strassfeld R, Wolff M. Maternal toxemia is associated with reduced incidence of germinal matrix hemorrhage in premature babies. J Child Neurol. 1992;7(1):70-6.
77. Lai TC, Liao CY. Maternal magnesium sulfate treatment and infant outcomes. J Obstet Gynaecol Res. 2017;43(Suppl 1):56-7.
78. Lee B, Cho GJ, Jin HM, Chung SH, Oh MJ, Kim HJ. Maternal magnesium sulfate treatment is not associated with serum calcium levels of preterm neonate. J Perinat Med. 2015;43:667.
79. Lee NY, Cho SJ, Park EA. Influence of antenatal magnesium sulfate exposure on perinatal outcomes in VLBW infants with maternal preeclampsia. Neonatal Med. 2013;20(1):28-34.
80. Leung JC, Cifra CL, Agthe AG, Sun CC, Viscardi RM. Antenatal factors modulate hearing screen failure risk in preterm infants. Arch Dis Child Fetal Neonatal Ed. 2016;101(1):F56-61.
81. Leviton A, Paneth N, Susser M, Reuss ML, Allred EN, Kuban K, et al. Maternal receipt of magnesium sulfate does not seem to reduce the risk of neonatal white matter damage. Pediatrics. 1997;99(4):E2.
82. Lipsitz PJ. The clinical and biochemical effects of excess magnesium in the newborn. Pediatrics. 1971;47(3):501-9.
83. Lloreda-Garcia JM, Lorente-Nicolás A, Bermejo-Costa F, Martínez-Uriarte J, López-Pérez R. Necesidad de reanimación en prematuros menores de 32 semanas expuestos a sulfato de magnesio para neuroprotección fetal. Rev Chil Pediatr. 2016;87(4):261-7.
84. Martin D, Gonzalez JL, Gardner MO, Izquierdo LA, Tobey K, Curet LB. Incidence of intraventricular hemorrhage in neonates under 32 weeks of gestation delivered to mothers with severe pre-eclampsia. Prenat Neonatal Med. 1998;3(2):250-4.
85. Matsuda Y, Maeda Y, Ito M, Sakamoto H, Masaoka N, Takada M, et al. Effect of magnesium sulfate treatment on neonatal bone abnormalities. Gynecol Obstet Invest. 1997;44(2):82-8.
86. McGuinness GA, Weinstein MM, Cruikshank DP, Pitkin RM. Effects of magnesium sulfate treatment on perinatal calcium metabolism. II. Neonatal responses. Obstet Gynecol. 1980;56(5):595-600.
87. McPherson JA, Rouse DJ, Grobman WA, Palatnik A, Stamilio DM. Association of duration of neuroprotective magnesium sulfate infusion with neonatal and maternal outcomes. Obstet Gynecol. 2014;124(4):749-55.
88. Mikhael M, Bronson C, Zhang L, Curran M, Rodriguez H, Bhakta KY. Lack of evidence for time or dose relationship between antenatal magnesium sulfate and intestinal injury in extremely preterm neonates. Neonatology. 2019;115(4):371-8.
89. Mitani M, Matsuda Y, Shimada E. Short- and long-term outcomes in babies born after antenatal magnesium treatment. J Obstet Gynaecol Res. 2011;37(11):1609-14.
90. Mittendorf R, Besinger R, Santillan M, Gianopoulos J. When used in the circumstance of preterm labor, is there a paradoxical effect of varying exposures to magnesium sulfate (MgSO4) on the developing human brain? Am J Obstet Gynecol. 2005;193(6):S65.
91. Mittendorf R, Pryde P, Gianopoulos J, Besinger R, Lee K-S. Thalamostriate vasculopathy in the neonate is associated with antenatal exposures to tocolytic MgSO4. Am J Obstet Gynecol. 2009;201(6):S79.
92. Morag I, Okrent AL, Strauss T, Staretz-Chacham O, Kuint J, Simchen MJ, et al. Early neonatal morbidities and associated modifiable and non-modifiable risk factors in a cohort of infants born at 34-35 weeks of gestation. J Matern Fetal Neonatal Med. 2015;28(8):876-82.
93. Morag I, Yakubovich D, Stern O, Siman-Tov M, Schushan-Eisen I, Strauss T, et al. Short-term morbidities and neurodevelopmental outcomes in preterm infants exposed to magnesium sulphate treatment. J Paediatr Child Health. 2016;52(4):397-401.
94. Moschos E, Magee K. Does magnesium sulfate exposure decrease the incidence of necrotizing enterocolitis? Am J Obstet Gynecol. 2001;185(6 Suppl):S148.
95. Murata Y, Itakura A, Matsuzawa K, Okumura A, Wakai K, Mizutani S. Possible antenatal and perinatal related factors in development of cystic periventricular leukomalacia. Brain Dev. 2005;27(1):17-21.
96. Nakamura Y, Ibara S, Ikenoue T. Effect of maternally administered magnesium sulfate on the neonate. J Perinat Med. 1991;19(Suppl 2):136.
97. Narasimhulu D, Brown A, Egbert NM, Rojas M, Haberman S, Bhutada A, et al. Maternal magnesium therapy, neonatal serum magnesium concentration and immediate neonatal outcomes. J Perinatol. 2017;37(12):1297-303.
98. Nassar AH, Sakhel K, Maarouf H, Naassan GR, Usta IM. Adverse maternal and neonatal outcome of prolonged course of magnesium sulfate tocolysis. Acta Obstet Gynecol Scand. 2006;85(9):1099-103.
99. Nelson KB, Grether JK. Can magnesium sulfate reduce the risk of cerebral palsy in very low birthweight infants? Pediatrics. 1995;95(2):263-9.
100. Nunes RD, Schutz FD, Traebert JL. Association between the use of magnesium sulfate as neuroprotector in prematurity and the neonatal hemodynamic effects. J Matern Fetal Neonatal Med. 2018;31(14):1900-5.
101. O Reilly E, Rogers EL, Hayes B. Effects of magnesium sulphate on respiratory function in the preterm infants who received magnesium sulphate prophylaxis at delivery. Ir J Med Sci. 2016;185:S277-8.
102. Okusanya BO, Garba KK, Ibrahim HM. The efficacy of 10gram intramuscular loading dose of MgSO(4) in severe preeclampsia/ eclampsia at a tertiary referral centre in Northwest Nigeria. Niger Postgrad Med J. 2012;19(3):143-8.
103. Özlü F, Hacıoğlu C, Büyükkurt S, Yapıcıoğlu H, Satar M. Changes on preterm morbidities with antenatal magnesium. Cukurova Med J. 2019;44(2):doi: 10.17826/cumj.444238.
104. Palatnik A, Liu LY, Lee A, Yee LM. Predictors of early-onset neonatal sepsis or death among newborns born at <32 weeks of gestation. J Perinatol. 2019;39(7):949-55.
105. Paneth N, Jetton J, Pinto-Martin J, Susser M. Magnesium sulfate in labor and risk of neonatal brain lesions and cerebral palsy in low birth weight infants. The Neonatal Brain Hemorrhage Study Analysis Group. Pediatrics. 1997;99(5):E1.
106. Perlman J, Fernandez C, Gee J, Leveno K, Risser R. Magnesium sulphate (Mg) administered to mothers with pregnancy-induced hypertension (PIH) is associated with a reduction in periventricular- intraventricular hemorrhage (PV-IVH). Pediatr Res. 1995;37(4 Pt 2):231A.
107. Petrov V, Lupascu A, Etsco L, Pavlenco A. Maternal and new born hemodynamics after antenatal administration of magnesium sulfate (MGSO4), as a neuroprotective drug in preterm birth. J Perinat Med. 2013;41:RU350.
108. Petrova A, Mehta R. Magnesium sulfate tocolysis and intraventricular hemorrhage in very preterm infants. Indian J Pediatr. 2012;79(1):43-7.
     - Lupascu A. The antenatal role administration of magnesium sulfate (MGSO4) as a neuroprotective drug in preterm birth. J Mater Fetal Neonatal Med. 2014;27(S1):388-9.*
109. Qasim A, Jain S, Dasgupta S. Does antenatal magnesium sulfate increase the likelihood of a hemodynamically significant patent ductus arteriosus in neonates? J Investig Med. 2017;65(2):547-8.
110. Rantonen T, Kaapa P, Gronlund J, Ekblad U, Helenius H, Kero P, et al. Maternal magnesium sulfate treatment is associated with reduced brain-blood flow perfusion in preterm infants. Crit Care Med. 2001;29(7):1460-5.
111. Rasch DK, Huber PA, Richardson CJ, L'Hommedieu CS, Nelson TE, Reddi R. Neurobehavioral effects of neonatal hypermagnesemia. J Pediatr. 1982;100(2):272-6.
112. Rattray BN, Kraus DM, Drinker LR, Goldberg RN, Tanaka DT, Cotten CM. Antenatal magnesium sulfate and spontaneous intestinal perforation in infants less than 25 weeks gestation. J Perinatol. 2014;34(11):819-22.
113. Rauf M, Sevil E, Ebru C, Yavuz S, Cemil C. Antenatal magnesium sulfate use for fetal neuroprotection: experience from a tertiary care hospital in Turkey. Biomed Res. 2017;28(4):1749-54.
114. Rhee E, Beiswenger T, Oguejiofor CE, James AH. The effects of magnesium sulfate on maternal and fetal platelet aggregation. J Matern Fetal Neonatal Med. 2012;25(5):478-83.
115. Riaz M, Porat R, Brodsky NL, Hurt H. The effects of maternal magnesium sulfate treatment on newborns: a prospective controlled study. J Perinatol. 1998;18(6 Pt 1):449-54.
116. Rizzolo A, Shah PS, Boucorian I, Lemyre B, Bertelle V, Pelausa E, et al. Cumulative effect of evidence-based practices on outcomes of preterm infants born at< 29 weeks gestational age. Am J Obstet Gynecol. 2019 Sept 6. doi: 10.1016/j.ajog.2019.08.058
117. Sahin H, Akay AF, Bircan MK, Gocmen A, Bircan Z. The first micturition times of the newborns whose mothers were treated with magnesium sulfate. Int Urol Nephrol. 2001;32(4):651-3.
118. Sakae C, Sato Y, Kanbayashi S, Taga A, Emoto I, Maruyama S, et al. Introduction of management protocol for early-onset severe pre-eclampsia. J Obstet Gynaecol Res. 2017;43(4):644-52.
119. Salafia CM, Minior VK, Rosenkrantz TS, Pezzullo JC, Popek EJ, Cusick W, et al. Maternal, placental, and neonatal associations with early germinal matrix/intraventricular hemorrhage in infants born before 32 weeks' gestation. Am J Perinatol. 1995;12(6):429-36.
120. Sarkar S, Bhagat I, Dechert R, Schumacher RE, Donn SM. Severe intraventricular hemorrhage in preterm infants: comparison of risk factors and short-term neonatal morbidities between grade 3 and grade 4 intraventricular hemorrhage. Am J Perinatol. 2009;26(6):419-24.
121. Schanler RJ, Smith LG, Burns PA. Effects of long-term maternal intravenous magnesium sulfate therapy on neonatal calcium metabolism and bone mineral content. Gynecol Obstet Invest. 1997;43(4):236-41.
122. Scudiero R, Khoshnood B, Pryde PG, Lee KS, Wall S, Mittendorf R. Perinatal death and tocolytic magnesium sulfate. Obstet Gynecol. 2000;96(2):178-82.
123. Shalabi M, Mohamed A, Lemyre B, Aziz K, Faucher D, Shah PS, et al. Antenatal exposure to magnesium sulfate and spontaneous intestinal perforation and necrotizing enterocolitis in extremely preterm neonates. Am J Perinatol. 2017;34(12):1227-33.
124. Shamsuddin L, Nahar K, Nasrin B, Nahar S, Tamanna S, Kabir RM, et al. Use of parenteral magnesium sulphate in eclampsia and severe pre-eclampsia cases in a rural set up of Bangladesh. Bangladesh Med Res Counc Bull. 2005;31(2):75-82.
125. Shokry M, Elsedfy GO, Bassiouny MM, Anmin M, Abozid H. Effects of antenatal magnesium sulfate therapy on cerebral and systemic hemodynamics in preterm newborns. Acta Obstet Gynecol Scand. 2010;89(6):801-6.
126. Stetson BT, Buhimschi CS, Kellert BA, Hay K, Buhimschi IA, Maitre NL. Comparison of cerebral palsy severity between 2 eras of antenatal magnesium use. JAMA Pediatr. 2019;173(2):188-90.
127. Stockley EL, Ting JY, Kingdom JC, McDonald SD, Barrett JF, Synnes AR, et al. Intrapartum magnesium sulfate is associated with neuroprotection in growth-restricted fetuses. Am J Obstet Gynecol. 2018;219(6):606e1-8.
128. Suh B, Ko K, Bang J, Oh Y, Lee Y, Lee J, et al. Neonatal outcomes of premature infants who were delivered from mother with hypertensive disorders of pregnancy and effects of antihypertensive drugs and MgSO4. Korean J Perinatol. 2015;26(3):190-9.
129. Teng RJ, Wu TJ, Sharma R, Garrison RD, Hudak ML. Early neonatal hypotension in premature infants born to preeclamptic mothers. J Perinatol. 2006;26(8):471-5.
130. Verma RP, Chandra S, Niwas R, Komaroff E. Risk factors and clinical outcomes of pulmonary interstitial emphysema in extremely low birth weight infants. J Perinatol. 2006;26(3):197-200.
131. Weintraub Z, Solovechick M, Reichman B, Rotschild A, Waisman D, Davkin O, et al. Effect of maternal tocolysis on the incidence of severe periventricular/intraventricular haemorrhage in very low birthweight infants. Arch Dis Child Fetal Neonatal Ed. 2001;85(1):F13-7.
132. Weisz D, Shivananda S, Asztalos E, Yee W, Synnes A, Lee S, et al. Intrapartum magnesium sulfate and need for intensive delivery room resuscitation. Arch Dis Child Fetal Neonatal Ed. 2015;100(1):F59-65.
133. Whitsel A, Insel A, Desilva H, Bernstein B. Association of maternal antepartum management with mortality and morbidity of the extremely low birthweight (ELBW) neonate. Am J Obstet Gynecol. 2004;191(6 Suppl):S75.
134. Whitten A, Ogunyemi D, Betcher K, Nowakowski A, Qu S. What factors predict prolonged neonatal length of stay in term babies? Int J Gynaecol Obstet. 2015;131:E462-3.
135. Wiswell TE, Caddell JL, Graziani LJ, Kornhauser MS, Spitzer AR. Maternally-administered magnesium sulfate (MgSO4) decreases the incidence of severe necrotizing enterocolitis (NEC) in preterm infants: A prospective study. Pediatr Res. 1996;39(4):1501.
     - Wiswell T, Graziani LC, JL., Vecchione N, Stanley C, Spitzer A. Maternally-administered magnesium sulfate (MgSO4) protects against early brain injury and long-term adverse neurodevelopmental outcomes in preterm infants: a prospective study. Pediatr Res. 1996;39:253.*
136. Wutthigate P, Yangthara B, Siripattanapipong P, Kitsommart R. Correlation between maternal cumulative dose of intrapartum magnesium sulfate and cord blood magnesium level. Southeast Asian J Trop Med Public Health. 2017;48(Suppl 2):256-63.
137. Yokoyama K, Takahashi N, Yada Y, Koike Y, Kawamata R, Uehara R, et al. Prolonged maternal magnesium administration and bone metabolism in neonates. Early Hum Dev. 2010;86(3):187-91.
138. Young BK, Weinstein HM. Effects of magnesium sulfate on toxemic patients in labor. Obstet Gynecol. 1977;49(6):681-5.

**Case reports**

1. Ahmad S, Miller M, Slaughter S. Is there any evidence for fetal harm with prolonged used of magnesium sulfate in pregnant women? Pharmacoepidemiol Drug Saf. 2013;22(1):141.
2. Amodio J, Berdon W, Abramson S, Stolar C. Microcolon of prematurity: a form of functional obstruction. AJR Am J Roentgenol. 1986;146(2):239-44.
3. Cruz M, Doren A, Fernandez B, Antonio Salinas J, Urzua S, Lui Tapia J. Intoxicación neonatal por sulfato de magnesio: caso clínico. Rev Chil Pediatr. 2009;80(3):261-6.
4. Cumming W, Thomas V. Hypermagnesemia: a cause of abnormal metaphyses in the neonate. AJR Am J Roentgenol. 1989;152(5):1071-2.
5. Herschel M, Mittendorf R. Tocolytic magnesium sulfate toxicity and unexpected neonatal death. J Perinatol. 2001;21(4):261-2.
6. Brady J. Magnesium intoxication in a premature infant. Pediatrics. 1967;40(1):100-3.
7. Jashi R, Gorgadze N. Maternal medication part of infant mortality. J Matern Fetal Neonatal Med. 2014;27:320-1.
8. Kaplan W, Haymond MW, McKay S, Karaviti LP. Osteopenic effects of MgSO4 in multiple pregnancies. J Pediatr Endocrinol Metab. 2006;19(10):1225-30.
9. Kogan JM, Wedig KE, Whitsett JA, Schorry EK. Prolonged prenatal exposure to magnesium sulfate associated with bone abnormalities mimicking genetic bone disease. Am J Hum Genet. 2003;73(5 Suppl):590.
10. Krasna IH, Rosenfeld D, Salerno P. Is it necrotizing enterocolitis, microcolon of prematurity, or delayed meconium plug? A dilemma in the tiny premature infant. J Pediatr Surg. 1996;31(6):855-8.
11. Kurtoglu S, Caksen H, Poyrazoglu MH. Neonatal poisonings in middle Anatolia of Turkey: an analysis of 72 cases. J Toxicol Sci. 2000;25(2):115-9.
12. L’Hommedieu CS, Huber P, Rasch DK. Potentiation of magnesium-induced neuromuscular weakness by gentamicin. Crit Care Med. 1983;11(1):55-6.
13. Lamm C, Norton K, Murphy R, Wilkins I, Rabinowitz J. Congenital rickets associated with magnesium sulfate infusion for tocolysis. J Pediatr. 1988;113(6):1078-82.
14. Lipsitz PJ EI. Hypermagnesemia in the newborn infant. Pediatrics. 1967;40(5):856-62.
15. Malaeb S, Rassi A, Haddad M, Seoud M, Yunis K. Bone mineralization in newborns whose mothers received magnesium sulphate for tocolysis of preterm labour. Pediatr Radiol. 2004;34(384-6).
16. Rasch D, Richardson C. Effect of gentamicin on neuromuscular function (NMF) of a hypermagnesemic neonate. Pediatr Res. 1981;15(4):499.
17. Sokal M, Koenigsberger M, Rose J, Berdon W, Santulli T. Neonatal hypermagnesemia and the meconium-plug syndrome. N Engl J Med. 1972;286(1):823-5.
18. Tanaka K, Mori H, Sakamoto R, Matsumoto S, Mitsubuchi H, Nakamura K, et al. Early-onset neonatal hyperkalemia associated with maternal hypermagnesemia: a case report. BMC Pediatr. 2018;15(1):55.
19. Teng R, Liu H, Tsou Yau K. Neonatal hypermagnesemia: report of one case. Acta Paediatr Sin. 1989;30(5):333-6.

*Indicates secondary reference providing additional relevant data for the the primary study reference listed immediately above
